# Burden of type 2 diabetes mellitus and its risk factors in North Africa and the Middle East, 1990–2019: findings from the Global Burden of Disease study 2019

**DOI:** 10.1186/s12889-023-16540-8

**Published:** 2024-01-05

**Authors:** Nazli Namazi, Sahar Saeedi Moghaddam, Shahnaz Esmaeili, Maryam Peimani, Yeganeh Sharifnejad Tehrani, Fatemeh Bandarian, Parnian Shobeiri, Ensieh Nasli-Esfahani, Mohammad-Reza Malekpour, Negar Rezaei, Nazila Rezaei, Babak Arjmand, Bagher Larijani, Farshad Farzadfar

**Affiliations:** 1 Diabetes Research Center, Endocrinology and Metabolism Clinical Sciences Institute, Tehran University of Medical Sciences, Tehran, Iran; 2 Endocrinology and Metabolism Population Sciences Institute, Non-Communicable Diseases Research Center, Tehran University of Medical Sciences, Tehran, Iran; 3 Metabolic Disorders Research Center, Endocrinology and Metabolism Molecular -Cellular Sciences Institute, Tehran University of Medical Sciences, Tehran, Iran; 4 Cell Therapy and Regenerative Medicine Research Center, Endocrinology and Metabolism, Molecular Cellular Sciences Institute, Tehran University of Medical Science, Tehran, Iran; 5 Metabolomics and Genomics Research Center Endocrinology and Metabolism Molecular- Cellular Sciences Institute, Tehran University of Medical Sciences, Tehran, Iran; 6 Endocrinology and Metabolism Research Center, Endocrinology and Metabolism Clinical Sciences Institute, Tehran University of Medical Sciences, Tehran, Iran; 7 Cell Therapy and Regenerative Medicine Research Center, Endocrinology and Metabolism Molecular-Cellular Sciences Institute, Tehran, Iran

**Keywords:** Burden, Risk factors, T2DM, GBD

## Abstract

**Background:**

The prevalence of Type 2 Diabetes Mellitus (T2DM) in the North Africa and Middle East region is alarmingly high, prompting us to investigate the burden and factors contributing to it through the GBD study. Additionally, there is a lack of knowledge about the epidemiological status of T2DM in this region, so our aim is to provide a comprehensive overview of the burden of T2DM and its associated risk factors.

**Methods:**

Using data from the 2019 Global Burden of Disease Study, we calculated the attributable burden of T2DM for each of the 21 countries in the region for the years 1990 and 2019. This included prevalence, mortality, disability-adjusted life years (DALYs), and risk factors.

**Results:**

Between 1990 and 2019, there was a significant increase in the age-standardized incidence (79.6%; 95% Uncertainty Interval: 75.0 to 84.5) and prevalence (85.5%; [80.8 to 90.3]) rates of T2DM per 100,000 populations. The age-standardized mortality rate (1.7%; [-10.4 to 14.9]), DALYs (31.2%; [18.3 to 42.2]), and years lived with disability (YLDs) (82.6%; [77.2 to 88.1]) also increased during this period. Modifiable risk factors, such as high body mass index (56.4%; [42.8 to 69.8]), low physical activity (15.5%; [9.0 to 22.8]), and ambient particulate matter pollution (20.9%; [15.2 to 26.2]), were the main contributors to the number of deaths.

**Conclusion:**

The burden of T2DM, in terms of mortality, DALYs, and YLDs, continues to rise in the region. The incidence rate of T2DM has increased in many areas. The burden of T2DM attributed to modifiable risk factors continues to grow in most countries. Targeting these modifiable risk factors could effectively reduce the growth and disease burden of T2DM in the region.

**Supplementary Information:**

The online version contains supplementary material available at 10.1186/s12889-023-16540-8.

## Background

Diabetes mellitus is a major global public health concern affecting populations worldwide. It encompasses different types, such as type 1, type 2 (T2D), and gestational diabetes. In T2D, the main factor contributing to abnormal blood sugar levels is the inability of β-cells to adequately compensate for peripheral insulin resistance. Additionally, disruptions in hormonal balance and organokines further contribute to the dysregulation of blood sugar in T2D. Metabolites from various tissues also play a role in the onset and progression of this health concern [1].

This disease places a significant socio-economic burden on governments and causes psychological distress for patients and their families [2]. According to the 9th edition of the International Diabetes Federation Diabetes report, the Middle East and North Africa had the highest age-standardized prevalence of diabetes worldwide in 2019, standing at 12.2%. The report also predicts that by 2045, the prevalence is projected to increase further to 13.9% in this region [3]. In addition, it is estimated that diabetes mellitus is a contributing factor in one out of nine deaths among individuals aged 20 to 79 years old. Approximately 11.5% of annual global deaths are attributed to diabetes, with percentages ranging from 6.8% in the Africa Region to 16.2% in the Middle East and North Africa [4]. The rising prevalence of type 2 diabetes mellitus (T2DM), which is the most common subtype of diabetes mellitus, is influenced by multiple factors. These include unhealthy lifestyle choices, the shift towards a more Westernized diet, obesity, industrialization, epigenetics, limited access to appropriate healthcare services, and other behavioral and environmental risk factors [5].

The prevalence of T2DM varies across countries in the region due to the differences in various societal dimensions mentioned earlier. Factors such as lifestyle, dietary patterns, cultural practices, socioeconomic conditions, healthcare infrastructure, and access to preventive healthcare services can all contribute to the variations in T2DM prevalence among different countries in the region [6–10]. Several studies have been conducted to examine the burden of diabetes mellitus and its associated metabolic disorders, focusing on specific risk factors in individual countries, regions, or globally. However, there is a need for comprehensive, multidimensional assessments that take into account the parameters mentioned earlier at both national and international levels. Such assessments could offer valuable insights into the effective management of T2DM.

The Global Burden of Diseases, Injuries, and Risk Factors (GBD) studies are examples of such studies that have provided suitable frameworks to quantify the comparative magnitude of health loss due to a specific disease and injury as well as risk factors of certain diseases.

In the present study, we tried to figure out the age and sex pattern of this disease in this region and across 21 countries. Considering 600 million people living in this region and the growing trend of T2DM across the world, encourages us to focus on the burden and attributed burden provided by the GBD study to explore what is going on here. In addition, there is a knowledge gap about the epidemiologic status of T2DM in this region, we conducted this study to reflect a comprehensive outlook of T2DM burden and attributed the burden to its risk factors. Accordingly, we aimed to provide the burden and attributable burden to metabolic, behavioral, and environmental exposures of T2DM at the regional and national levels for 21 countries in the region from 1990 to 2019.

## Methods

In the present study, data from GBD study 2019 were used to estimate the burden and attributable burden of T2DM in the region. The entry data were obtained through GBD results (http://ghdx.healthdata.org/gbd-results-tool) and compare (https://vizhub.healthdata.org/gbd-compare) tools which are prepared by the Institute for Health Metrics and Evaluation (IHME). Details of GBD study 2019 are provided elsewhere [11–13]. Briefly, GBD 2019 study can provide a ground to estimate the prevalence of exposure and attributable mortalities, Years Lived with Disability (YLDs), Years of Life Lost (YLLs), and Disability-Adjusted Life Years (DALYs) for a cluster of diseases in both genders (separately and combined) and 23 age groups from 1990 to 2019. It contains information of 204 countries and territories classified in 21 GBD regions and 7 super-regions.

For the current paper, we used the data of GBD 2019 study for T2DM in 21 countries in the region considering age group, sex, and location. According to the Institute for Health Metrics and Evaluation (IHME), countries in this region are as follows: Afghanistan, Algeria, Bahrain, Egypt, Iran (Islamic Republic of), Iraq, Jordan, Kuwait, Lebanon, Libya, Morocco, Oman, Palestine, Qatar, Saudi Arabia, Sudan, Syrian Arab Republic, Tunisia, Turkey, United Arab Emirates (UAE), and Yemen. To define T2DM, the International Classification of Diseases 10th Revision (ICD-10) codes of E11-E11.1, E11.3-E11.9 were used.

### Burden of T2DM

In this study, we aimed to estimate the mortality rate, years of life lost (YLLs), years lived with disability (YLDs), and disability-adjusted life years (DALYs) attributed to T2DM in both the region as a whole and in each of the 21 individual countries. We took into consideration age and sex when calculating these estimates.

To determine the number of deaths caused by T2DM, we first estimated all-cause mortality rates for each of the 21 countries in the region from 1990 to 2019. We used various sources of data, including vital registration systems, sample registration data, household reports of deaths, and sibling history surveys, to gather information and construct the mortality envelopes.

By utilizing these data sources, we were able to estimate the overall burden of T2DM on mortality and quantify the impact of the disease across different age groups and genders in the region and within each individual country (Supplementary Table 1). Furthermore, we collected cause of mortality data from the same sources mentioned earlier, as well as available verbal autopsies. Using a cause of death ensemble modeling approach, we utilized this data to estimate the number of deaths specifically attributed to T2DM. These estimates were calculated based on factors such as year, age group, sex, and location, allowing for a more detailed understanding of the impact of T2DM on mortality in the region and within each country [13].

### Attributable burden

The GBD 2019 study utilized a hierarchical list of risk factors, which included specific risk factors and related aggregates. The analysis involved six key steps:

1. Identification of Risk-Outcome Pairs: The study considered risk-outcome pairs that met the criteria of convincing or probable evidence from scientific research. Additionally, new risk-outcome pairs were included based on emerging evidence.
2. Estimation of Relative Risks: Published systematic reviews and meta-regressions were used to estimate the relative risks associated with each risk-outcome pair.
3. Estimation of Exposure Levels: Exposure levels for each age group, sex, country, and year were estimated using available data sources, employing techniques such as spatiotemporal Gaussian process regression, DisMod-MR 2.1, and Bayesian metaregression.
4. Determination of Minimum Theoretical Risk Exposure Levels: Minimum theoretical risk exposure levels were determined based on data from cohort studies or trials, representing the exposure level associated with the lowest risk.
5. Calculation of Attributable Burden: Attributable deaths, DALYs, YLDs, and YLLs were calculated by multiplying the population attributable fractions (PAFs) with the relevant outcome quantity for each year (1990–2019), age group, country, and sex.
6. Estimation of Burden for Risk Factor Combinations: The study also considered the mediation of various risk factors through other risk factors, estimating PAFs and attributable burden for combinations of risk factors.

These analytical steps enabled a comprehensive evaluation of the burden attributed to different risk factors, providing valuable insights into the impact of these factors on health outcomes [12].

### Socio-demographic index (SDI)

Socio-demographic index (SDI) calculated based on geometric mean of lag-distribution income per capita (ln LDI), total fertility rate < 2.5 (TFU25), and average educational attainment for those older than 15 years (EDU15). The highest value of the SDI (1) represents the theoretical maximum level of development relevant to health outcomes compared to the lowest value (0) which shows the theoretical minimum level.

$$SDI\;=\;\sqrt[3]{I_{LnLDI}\;\ast\;I_{TFU25}\;\ast\;I_{EDU15}}$$

### Mortality-to-incidence ratio (MIR)

Mortality-to-incidence ratio (MIR) is an indicator for the quality of care. To calculate MIR, available data of all-age crude death rate was divided by all-age crude incidence rate of T2DM [14].

### Decomposition analysis

To provide the proportion of population growth, age structure change and incidence rate change on the overall change of T2DM new cases, a decomposition analysis was applied between 1990 and 2019. In this regard, two scenarios were considered. For the first one, the age and sex structures and the age-specific rates from initial year (1990) were applied to the ultimate year (2019) total population. The difference between the total number of new cases in the initial year and the hypothetical population growth scenario was assigned. For the second one, the age-specific rates from initial year were applied to the age and sex structures and population size of the ultimate year and the differences between this hypothetical scenario and the first one were considered as population aging. To calculate the age-specific rate change ratio, the difference between the total number of T2DM cases in 2019 and the second hypothetical scenario was considered [15].

GBD 2019 documented each step of the estimation processes and data sources on the basis on the Guidelines for Accurate and Transparent Health Estimates Reporting (GATHER) statement [16]. In this paper, 95% uncertainty interval (UI) for every measure and metric of the estimates and percent changes were reported. UIs were estimated by ordering 1000 samples of the posterior distribution of each quantity and applied the 2.5^th^and 97.5^th^ ordered draw of the uncertainty distribution. Further to report the percent changes reported in the GBD study, we calculated the annual percent changes (APC) from the joinpoint regression model. Age-standardization was done using the direct method, with a GBD world population age standard. The non-weighted mean of 2019 age-specific proportional distributions for all national locations with a population greater than 5 million people in 2019 was used to generate a standard population age structure.

## Results

### Burden of T2DM

Incremental trends for the changes of age-standardized incidence (79.6%; 95% UI: 75.0 to 84.5) and prevalence (85.5%; [80.8 to 90.3]) rates of T2DM were observed between 1990 and 2019. Percentage changes of age-standardized mortality rate (1.7%; [-10.4 to 14.9]), DALYs (31.2%; [18.3 to 42.2]), and YLDs (82.6%; [77.2 to 88.1]) for T2DM have been experienced rising patterns from 1990 to 2019 in this region. However, YLLs were decreased during this period (-0.6%; [-13.1 to 13.3]) (Table 1).

**Table 1:** All age numbers and age-standardized rate of T2DM burden by sex in 1990 and 2019 with percent changes between 1990 and 2019 in the region

Measure	Age, Metric	Both			Female			Male		
		Year		% Change (1990 to 2019)	Year		% Change (1990 to 2019)	Year		% Change (1990 to 2019)
		1990	2019		1990	2019		1990	2019	
Incidence	All ages (number)	441,642 (405,349 to 481,563)	2,007,270 (1,842,502 to 2,191,938)	354.5 (341.1 to 367.5)	219,001 (201,430 to 238,224)	952,255 (877,008 to 1,039,978)	334.8 (321 to 347.7)	222,641 (203,805 to 243,612)	1,055,015 (967,902 to 1,151,473)	373.9 (359.1 to 389.3)
	Age-standardized rate (per 100,000)	196.6 (180.7 to 213.7)	353.2 (326.1 to 383.4)	79.6 (75 to 84.5)	200.7 (185.2 to 217.3)	351.5 (325.3 to 381.9)	75.2 (70.3 to 80.3)	192.4 (177 to 209.4)	354.4 (326.3 to 385)	84.2 (78.7 to 89.9)
Prevalence	All ages (number)	6,959,611 (6,320,252 to 7,662,966)	32,927,679 (29,948,035 to 36,227,971)	373.1 (361.4 to 385)	3,475,754 (3,152,916 to 3,820,990)	15,787,738 (14,371,677 to 17,303,627)	354.2 (340.9 to 366.8)	3,483,857 (3,146,895 to 3,846,127)	17,139,941 (15,563,041 to 18,973,186)	392 (378.5 to 406.8)
	Age-standardized rate (per 100,000)	3640.4 (3313.8 to 3996)	6753.3 (6170.2 to 7394.2)	85.5 (80.8 to 90.3)	3700.2 (3368.8 to 4057.8)	6706.2 (6129.1 to 7328.4)	81.2 (75.8 to 86.8)	3580.9 (3245.7 to 3940.9)	6794.8 (6201.7 to 7431.4)	89.8 (84.1 to 95.6)
Deaths	All ages (number)	36,365 (33,087 to 40,381)	95,375 (84,916 to 107,421)	162.3 (130.3 to 198.2)	20,954 (18,267 to 23,850)	50,900 (44,649 to 59,988)	142.9 (112.3 to 190.5)	15,411 (13,836 to 17,359)	44,475 (39,036 to 50,851)	188.6 (137.9 to 241.3)
	Age-standardized rate (per 100,000)	24.8 (22.5 to 27.7)	25.2 (22.4 to 28.2)	1.7 (-10.4 to 14.9)	28.6 (24.7 to 33.1)	27.6 (24.1 to 32.2)	-3.6 (-15.5 to 14.6)	20.7 (18.6 to 23.3)	22.8 (20 to 25.9)	10.3 (-8.4 to 29.3)
DALYs	All ages (number)	1,429,598 (1,229,348 to 1,675,907)	4,806,918 (3,927,069 to 5,857,180)	236.2 (202.8 to 264.6)	771,460 (657,893 to 916,400)	2,406,794 (1,958,949 to 2,941,423)	212 (180.1 to 246.6)	658,139 (555,366 to 777,299)	2,400,124 (1,946,053 to 2,933,251)	264.7 (221.7 to 300.7)
	Age-standardized rate (per 100,000)	808.3 (696.9 to 944.3)	1060.8 (872.1 to 1279.1)	31.2 (18.3 to 42.2)	887.1 (763.2 to 1050.1)	1096.2 (902.4 to 1324.9)	23.6 (11.2 to 37.9)	728.8 (617.7 to 855.2)	1025.5 (840.8 to 1243.2)	40.7 (24.1 to 54.2)
YLLs	All ages (number)	857,761 (785,019 to 953,813)	2,139,801 (1,875,264 to 2,453,045)	149.5 (116.6 to 186.2)	481,904 (421,959 to 543,101)	1,104,840 (962,742 to 1,308,934)	129.3 (98.5 to 175.2)	375,857 (335,420 to 426,788)	1,034,961 (892,583 to 1,198,623)	175.4 (124.8 to 230.4)
	Age-standardized rate (per 100,000)	499 (455.6 to 551.1)	496.2 (439.1 to 562.3)	-0.6 (-13.1 to 13.3)	569.6 (499.1 to 642.2)	528.7 (462.3 to 622.7)	-7.2 (-19 to 11.2)	427.5 (383.7 to 481.8)	463.6 (401.6 to 532.5)	8.4 (-10.9 to 28.6)
YLDs	All ages (number)	571,838 (382,036 to 792,767)	2,667,118 (1,775,783 to 3,687,122)	366.4 (353.5 to 379.2)	289,556 (194,029 to 400,176)	1,301,954 (870,737 to 1,798,142)	349.6 (335 to 364.2)	282,282 (188,718 to 392,427)	1,365,164 (903,123 to 1,892,498)	383.6 (368.2 to 399.1)
	Age-standardized rate (per 100,000)	309.3 (208.1 to 426.4)	564.6 (380.7 to 777.9)	82.6 (77.2 to 88.1)	317.5 (213 to 439.1)	567.5 (381.1 to 781.3)	78.8 (72.9 to 84.8)	301.3 (203.7 to 417.1)	561.9 (375.5 to 774)	86.5 (80.4 to 92.7)

Data in parentheses are 95% uncertainty interval (UI); *DALYs* Disability-Adjusted Life Years, *YLLs* Years of Life Lost, *YLDs* Years Lived with Disability

The percentage changes in the burden of T2DM in 12 out of 21 countries have also been positive in these 30 years However, downward changes in age-standardized death rate per 100,000 populations were observed in 8 countries from -7.0% to 51.7%. In these countries, upward changes in both incidence and prevalence of patients with T2DM were also observed (Tables 2, and Table 3, Supplementary Tables 2 and 3). Evaluating the APC of the age-standardized rate by each burden measures in the region and its 21 countries for incidence, prevalence and YLDs were positive while for the death, DALYs and YLLs different pattern was observed (Supplementary Table 4).

**Table 2:** Burden and a ranking of region countries based on the age-standardized incidence and prevalence rates in 1990 and 2019 with percent change

**Country**	**Both**					**Female**					**Male**				
	**1990**		**2019**		**% Change (1990 to 2019)**	**1990**		**2019**		**% Change (1990 to 2019)**	**1990**		**2019**		**% Change (1990 to 2019)**
	**ASR (per 100,000)**	**Ranking**	**ASR (per 100,000)**	**Ranking**		**ASR (per 100,000)**	**Ranking**	**ASR (per 100,000)**	**Ranking**		**ASR (per 100,000)**	**Ranking**	**ASR (per 100,000)**	**Ranking**	
**Incidence**															
**Afghanistan**	226.88 (204.75 to 251.76)	12	412.01 (371.66 to 458.7)	9	81.6 (73.3 to 89.4)	245.1 (219.54 to 271.85)	9	451.7 (404.09 to 502.25)	4	84.3 (74.9 to 94.5)	206.34 (184.3 to 230.7)	15	373.92 (335.46 to 419.3)	13	81.2 (69.9 to 93.4)
**Algeria**	211.89 (191.35 to 234.05)	15	386.38 (350.31 to 428.79)	13	82.4 (72 to 92.9)	215.64 (193.48 to 237.97)	14	400.66 (362.81 to 442.74)	10	85.8 (72.7 to 100.1)	208.43 (188.36 to 232.78)	14	372.31 (333.53 to 415.36)	14	78.6 (65.9 to 93.5)
**Bahrain**	442.46 (409.23 to 476.74)	2	757.62 (721.3 to 795.85)	2	71.2 (58.9 to 83.3)	410.29 (378.81 to 444.26)	3	727.56 (691.77 to 763.31)	2	77.3 (64.9 to 90)	464.52 (426.01 to 505.79)	2	771.02 (731.79 to 813.2)	2	66 (51.8 to 79.1)
**Egypt**	140.81 (130.44 to 151.68)	21	295.24 (268.25 to 325.18)	19	109.7 (97.1 to 123.8)	167.29 (154.25 to 182.04)	19	311.79 (285.25 to 340.8)	19	86.4 (72.8 to 100.2)	114.36 (104.77 to 124.21)	21	281.06 (251.37 to 313.02)	19	145.8 (128.3 to 166.7)
**Iran (Islamic Republic of)**	170.51 (154.95 to 188.36)	19	323.31 (295.73 to 354.45)	18	89.6 (82.5 to 97.3)	166.15 (150.04 to 183.6)	20	338.09 (307.89 to 370.83)	18	103.5 (94.6 to 113.7)	173.83 (158.21 to 192.35)	19	308.53 (282.46 to 338.07)	18	77.5 (69.9 to 85.8)
**Iraq**	286.87 (262.83 to 312.74)	6	424.91 (392.39 to 460.2)	8	48.1 (37.1 to 59.8)	292.2 (266.47 to 318.66)	6	439.64 (399.87 to 480.44)	8	50.5 (36.8 to 64.4)	281.84 (255.11 to 309.75)	7	410 (370.72 to 450)	12	45.5 (32.1 to 59.7)
**Jordan**	300.4 (277.93 to 323.26)	5	395.59 (363.36 to 428.41)	12	31.7 (22.9 to 41.3)	305.47 (283.07 to 328.65)	5	355.16 (322.18 to 390.42)	14	16.3 (6.4 to 26.3)	294.03 (268.92 to 322.79)	6	427.58 (386.65 to 467.44)	10	45.4 (32.7 to 59.3)
**Kuwait**	346.89 (317.74 to 378.89)	4	495.49 (451.9 to 542.72)	4	42.8 (33.1 to 53.1)	328.22 (297.05 to 358.17)	4	449.77 (402.63 to 498.82)	5	37 (25.6 to 49.6)	356.43 (322.29 to 390.8)	4	531.85 (481.91 to 585.5)	4	49.2 (36.9 to 62.8)
**Lebanon**	231.3 (211.83 to 255.18)	11	383.5 (347.67 to 424.07)	14	65.8 (56.2 to 74.9)	218.7 (199.26 to 240.52)	13	349.96 (316.13 to 387.2)	16	60 (49 to 71.8)	243.23 (220.89 to 270.3)	12	423.3 (377.46 to 472.86)	11	74 (60.7 to 88.8)
**Libya**	244.75 (221.33 to 271.05)	9	454.79 (409.66 to 506.5)	6	85.8 (75.1 to 95.7)	235 (210.38 to 260.3)	11	443.94 (398.4 to 494.06)	6	88.9 (75.5 to 103.7)	252.23 (227.66 to 281.24)	10	465.07 (416.29 to 520.15)	7	84.4 (70.8 to 97.4)
**Morocco**	178.73 (161.37 to 198.14)	18	345.08 (311.46 to 378.66)	17	93.1 (83 to 103.3)	177.24 (158.21 to 198.05)	18	345.36 (308.9 to 380.83)	17	94.9 (80.6 to 109.3)	180.06 (162.21 to 200.96)	18	344.58 (310.27 to 384.2)	16	91.4 (78.4 to 104.6)
**Oman**	241.99 (222.13 to 264.43)	10	410.33 (374.42 to 447.78)	10	69.6 (60.5 to 79)	238.65 (218.26 to 261.26)	10	370.08 (336.56 to 409.8)	11	55.1 (44.9 to 66.3)	244.21 (221.18 to 267.82)	11	433.41 (393.48 to 474.56)	9	77.5 (66.5 to 90.9)
**Palestine**	258.18 (237.51 to 281.69)	8	452.68 (414.55 to 485.39)	7	75.3 (62 to 89)	250.46 (229.58 to 273.73)	8	415.86 (380.11 to 452.48)	9	66 (52.5 to 82.6)	267.64 (244.05 to 296.44)	8	486.72 (443.15 to 525.54)	5	81.9 (64.6 to 100)
**Qatar**	493.12 (453.74 to 534.08)	1	818.03 (773.89 to 868.7)	1	65.9 (53.3 to 79.1)	475.71 (435.23 to 517.66)	1	821.66 (770.07 to 874.31)	1	72.7 (57.4 to 87.1)	497.63 (456.97 to 541.98)	1	816.78 (771.41 to 872.95)	1	64.1 (51.4 to 78.1)
**Saudi Arabia**	282.68 (260.43 to 306.36)	7	462.07 (420.77 to 506.93)	5	63.5 (53.8 to 73.5)	263.99 (241.85 to 289.58)	7	441.81 (399.45 to 491.13)	7	67.4 (56 to 80)	294.06 (270.18 to 320.4)	5	475.63 (431.03 to 526.32)	6	61.7 (50 to 74.7)
**Sudan**	186.55 (168.72 to 206.62)	17	359.63 (323.43 to 399.64)	15	92.8 (84.1 to 101.6)	186.38 (167.72 to 206.98)	17	355.25 (319.17 to 396.54)	13	90.6 (79.1 to 102.7)	186.44 (166.46 to 208.09)	17	363.98 (325.53 to 407.6)	15	95.2 (82.5 to 109.7)
**Syrian Arab Republic**	224.53 (204.77 to 245.98)	14	345.74 (312.53 to 378.21)	16	54 (45.5 to 63.7)	231.4 (208.47 to 255.33)	12	351.13 (315.62 to 386)	15	51.7 (40.6 to 64)	218.26 (198.21 to 240.98)	13	341.16 (305.91 to 374.4)	17	56.3 (43.7 to 70.6)
**Tunisia**	226.12 (205.28 to 249.65)	13	408.64 (369.92 to 451.9)	11	80.7 (72.5 to 89.5)	197.34 (176.58 to 219.71)	16	360.14 (322.92 to 400.67)	12	82.5 (70.2 to 94.7)	254.51 (230.19 to 282.11)	9	458.43 (411.83 to 508.7)	8	80.1 (67.6 to 93.2)
**Turkey**	202.48 (188.56 to 219.42)	16	273.83 (248.82 to 298.61)	20	35.2 (23.7 to 47.2)	211.57 (197.44 to 228.77)	15	269.1 (243.42 to 296.39)	20	27.2 (15 to 41.1)	192.66 (175.93 to 211.27)	16	277.91 (249.26 to 307.86)	20	44.2 (28.8 to 60.1)
**United Arab Emirates**	397.24 (364.66 to 431.78)	3	589.9 (545.98 to 641.76)	3	48.5 (40.5 to 57.7)	419.13 (380.53 to 456.47)	2	601.88 (556.14 to 653.68)	3	43.6 (33.4 to 54.7)	383.84 (352.54 to 421.14)	3	583.36 (537.03 to 638.6)	3	52 (41.9 to 63.1)
**Yemen**	145.9 (131.49 to 161.79)	20	241.73 (217.44 to 268.59)	21	65.7 (57.3 to 75.3)	145.02 (129.16 to 160.51)	21	246.74 (221.49 to 274.51)	21	70.1 (59 to 82.5)	145.99 (130.85 to 163.37)	20	236.65 (210.36 to 266.69)	21	62.1 (50.8 to 75.1)
**Prevalence**															
**Afghanistan**	4458.65 (4001.18 to 4972.69)	10	8537.38 (7658.31 to 9562.53)	8	91.5 (82.3 to 100.1)	4893.3 (4371.37 to 5478.21)	8	9499.6 (8456.34 to 10688.04)	4	94.1 (83 to 105.4)	3991.07 (3544.93 to 4497.15)	14	7554.56 (6722.6 to 8503.12)	13	89.3 (76.5 to 102)
**Algeria**	4047.97 (3641.25 to 4517.59)	15	7675.14 (6860.5 to 8599.14)	11	89.6 (78.4 to 101.2)	4113.33 (3649.88 to 4587.26)	14	7963.42 (7102.72 to 8915.81)	9	93.6 (78.9 to 109.5)	3983.27 (3538.87 to 4479.77)	15	7396.28 (6590.24 to 8334.63)	14	85.7 (72.9 to 100.2)
**Bahrain**	7546.04 (6822.36 to 8259.72)	2	14234.85 (13261.86 to 15287.17)	2	88.6 (73.6 to 103.5)	6989.22 (6293.42 to 7705.71)	3	13458.47 (12533.43 to 14496.73)	2	92.6 (77.1 to 108.9)	8010.27 (7153.16 to 8811.55)	2	14673.74 (13497.95 to 15950.67)	2	83.2 (64.5 to 102.2)
**Egypt**	2392.2 (2172.59 to 2629.73)	21	5657.59 (5105.82 to 6302.28)	19	136.5 (119.8 to 154.2)	2783.87 (2500.73 to 3085.8)	20	5823.77 (5293.81 to 6463.25)	19	109.2 (92 to 127.3)	1996.27 (1791.31 to 2208.78)	21	5541.82 (4935.32 to 6229.41)	19	177.6 (155.4 to 204.1)
**Iran (Islamic Republic of)**	3285.57 (2940.82 to 3652.42)	19	6312.86 (5690.22 to 6959.49)	18	92.1 (85.4 to 99.6)	3239.89 (2898.09 to 3595.61)	19	6555.23 (5898.76 to 7251.32)	18	102.3 (94.3 to 111.6)	3326.95 (2979.36 to 3711.08)	19	6066.88 (5456.28 to 6663.1)	18	82.4 (74.5 to 91.2)
**Iraq**	5595.54 (5041.53 to 6185.75)	5	8564.58 (7852.92 to 9298.92)	7	53.1 (41.1 to 65.7)	5777.67 (5199.83 to 6414.74)	5	8860.28 (7984.86 to 9708.97)	8	53.4 (38.9 to 69.2)	5421.17 (4864.17 to 6054.87)	6	8259.41 (7399.86 to 9105.93)	11	52.4 (37.6 to 67.6)
**Jordan**	5243.64 (4824.32 to 5714.45)	7	7628.82 (6944.1 to 8317.96)	13	45.5 (35 to 55.9)	5063.47 (4624.39 to 5533.31)	6	6631.38 (5961.46 to 7367.58)	17	31 (20 to 43.8)	5393.85 (4885.58 to 5971.69)	7	8493.1 (7575.37 to 9405.44)	9	57.5 (43.8 to 72.9)
**Kuwait**	6685.25 (6107.33 to 7339.88)	4	10250.15 (9240.18 to 11339.5)	4	53.3 (42.1 to 63.9)	6270.75 (5603.16 to 6879.15)	4	9154.55 (8175.14 to 10231.55)	5	46 (32.9 to 59.7)	6941.46 (6271.12 to 7647.16)	3	11041.41 (9941.81 to 12281.38)	3	59.1 (46.3 to 73.3)
**Lebanon**	4447.09 (4035.03 to 4924.77)	11	7653.3 (6870.97 to 8524.27)	12	72.1 (61.2 to 82)	4259.42 (3854.25 to 4726.7)	12	7022.55 (6269.32 to 7837.39)	13	64.9 (52.6 to 77.3)	4638.28 (4172.23 to 5164.29)	11	8414.41 (7497.83 to 9458.38)	10	81.4 (66.9 to 96.8)
**Libya**	4770.42 (4282.05 to 5283.8)	8	9291.96 (8328.27 to 10384.67)	6	94.8 (83 to 105.6)	4573.76 (4072.45 to 5122.4)	9	9029.12 (8011.96 to 10112.86)	7	97.4 (83.3 to 113.1)	4942.56 (4433.3 to 5516.01)	8	9543.78 (8488.53 to 10713.8)	6	93.1 (78 to 107.8)
**Morocco**	3419.56 (3061.95 to 3803.46)	18	6918.03 (6211.82 to 7684.05)	16	102.3 (91.7 to 113.5)	3398.02 (3024.55 to 3807.52)	18	6952.09 (6205.42 to 7793.77)	14	104.6 (89.3 to 121.3)	3440.44 (3069.9 to 3843.15)	18	6883.7 (6137.36 to 7694.85)	16	100.1 (86.2 to 113.7)
**Oman**	4248.79 (3825.28 to 4693.36)	14	7423.26 (6666.41 to 8199.82)	14	74.7 (64.5 to 84.7)	4237.37 (3785.31 to 4715.79)	13	6823.77 (6125.42 to 7666.97)	16	61 (49.2 to 74.5)	4303.81 (3831.27 to 4795.66)	12	7906.67 (7072.4 to 8756.05)	12	83.7 (69.5 to 98.3)
**Palestine**	4569.68 (4130.77 to 5052.97)	9	8319.6 (7549.86 to 9012.96)	9	82.1 (67.8 to 97.1)	4366.11 (3931.35 to 4847.3)	11	7566.61 (6813.85 to 8323.87)	10	73.3 (57.7 to 90.8)	4819.07 (4316.24 to 5387.49)	10	9104.65 (8189.87 to 9907.89)	8	88.9 (70.2 to 108.1)
**Qatar**	8552.63 (7728.45 to 9442.19)	1	16312.38 (15050.01 to 17723.24)	1	90.7 (75.2 to 107.8)	8308.73 (7432.39 to 9176.11)	1	16294.85 (14866.9 to 17796.65)	1	96.1 (76.2 to 116.4)	8738.41 (7854.85 to 9738.42)	1	16309.75 (15082.79 to 17843.78)	1	86.6 (70.8 to 105.6)
**Saudi Arabia**	5380.74 (4914.32 to 5869.38)	6	9453.1 (8563.11 to 10498.61)	5	75.7 (65.4 to 87.1)	5018.12 (4534.37 to 5539.92)	7	9037.04 (8145.53 to 10160.64)	6	80.1 (67.1 to 94.2)	5638.31 (5129.71 to 6194.53)	5	9728.54 (8777.61 to 10881.72)	5	72.5 (59.9 to 87.2)
**Sudan**	3621.67 (3234.61 to 4040.03)	16	7272 (6509.85 to 8134.3)	15	100.8 (91.6 to 110.8)	3603.11 (3211.94 to 4040.01)	17	7155.51 (6396.4 to 8021.97)	11	98.6 (86.1 to 112)	3636.53 (3219.15 to 4088.49)	16	7371.91 (6548.48 to 8260.8)	15	102.7 (89.8 to 117.8)
**Syrian Arab Republic**	4341.77 (3942.21 to 4797.34)	13	6832.55 (6133.83 to 7554.81)	17	57.4 (47.8 to 67.7)	4419.06 (3952.24 to 4904.8)	10	6852.48 (6071.44 to 7632.53)	15	55.1 (43.2 to 68.8)	4271.15 (3826.59 to 4746.72)	13	6824 (6058.81 to 7597.9)	17	59.8 (47.3 to 74.3)
**Tunisia**	4352.58 (3907.53 to 4816.59)	12	8162.22 (7324.55 to 9059.79)	10	87.5 (78.5 to 97.1)	3758.32 (3334.82 to 4202.3)	15	7132 (6318.78 to 7951.1)	12	89.8 (76.7 to 102.8)	4919.71 (4434.75 to 5506.76)	9	9239.46 (8228.74 to 10271.12)	7	87.8 (74.2 to 101.8)
**Turkey**	3582.84 (3284.41 to 3912.64)	17	5081.97 (4584.64 to 5564.4)	20	41.8 (29.5 to 54.5)	3712.39 (3411.34 to 4047.73)	16	4963.87 (4432.35 to 5496.96)	20	33.7 (20.2 to 47.7)	3443.51 (3091.38 to 3797.16)	17	5204.34 (4648.12 to 5783.56)	20	51.1 (34.7 to 68.8)
**United Arab Emirates**	6855.76 (6171.62 to 7585.2)	3	11098.17 (10089.01 to 12249.41)	3	61.9 (52.2 to 72.8)	7156.57 (6328.59 to 7956.75)	2	11175.67 (10171.77 to 12325.5)	3	56.2 (43.7 to 70.1)	6650.38 (5979.02 to 7413.97)	4	11028.11 (9980.49 to 12236.78)	4	65.8 (53.4 to 78.8)
**Yemen**	2720.16 (2418.55 to 3042.05)	20	4686.94 (4150.59 to 5245.5)	21	72.3 (63.6 to 82)	2690.75 (2378.52 to 3028.32)	21	4760.69 (4223.42 to 5376.85)	21	76.9 (64.9 to 90.2)	2754.74 (2432.77 to 3103.29)	20	4611.5 (4068.95 to 5223.07)	21	67.4 (55.6 to 81.2)

*ASR* Age-standardized rate; Data in parentheses are 95% Uncertainty Intervals (95% UIs)

Change in the ranking of countries (range from 1 (the highest estimate) to 21 (the lowest estimate)) in 2019 vs 1990 were classified by three groups:

Upward

Monotone

Downward

The greatest age-standardized rate of incidence (493.1; [453.7 to 534.1]) and prevalence (8552.6; [7728.4 to 9442.2]) per 100,000 in 1990 were estimated for Qatar. In 2019, the first rank for both the incidence (818; [773.9 to 868.7]) and prevalence (16,312.4; [15050 to 17,723.2]) rate per 100,000 of T2DM was also dedicated to this country. In 2019, the incidence in Qatar was 3.39 times greater than Yemen as a country with the least age-standardized incidence rate. A significant downward trend was observed in the age-standardized incidence and prevalence of T2DM in Jordan between 1990 and 2019 (Table 2). This country had stood at 5^th^ rank of the age-standardized incidence in 1990 and its rank fall into 12^th^ country in 2019. The similar pattern was observed for this country in age-standardized death and DALYs trend. The rank of Turkey in age-standardized DALYs rate from 8 among the countries in 1990 failed into 18 in 2019 (Table 3). Other burden parameters (incidence, prevalence, and death) were also decreased through these 30 years. Ranking of countries in the age-standardized burden rates of T2DM were different between males and females (Tables 2, and Table 3 Supplementary Tables 2 and 3).

**Table 3:** Burden and a ranking of region countries based on the age-standardized deaths and DALYs rates in 1990 and 2019 with percent change

**Country**	**Both**					**Female**					**Male**				
	**1990**		**2019**		**% Change (1990 to 2019)**	**1990**		**2019**		**% Change (1990 to 2019)**	**1990**		**2019**		**% Change (1990 to 2019)**
	**ASR (per 100,000)**	**Ranking**	**ASR (per 100,000)**	**Ranking**		**ASR (per 100,000)**	**Ranking**	**ASR (per 100,000)**	**Ranking**		**ASR (per 100,000)**	**Ranking**	**ASR (per 100,000)**	**Ranking**	
**Deaths**															
**Afghanistan**	27.82 (18.48 to 40.5)	10	39.88 (23.39 to 58)	8	43.4 (3.7 to 85.1)	38.16 (22.16 to 60.59)	9	57.1 (28.93 to 88.76)	4	49.6 (5.2 to 102)	18.31 (13.1 to 26.01)	13	21.09 (14.91 to 29.41)	10	15.2 (-15.1 to 50.3)
**Algeria**	17.01 (12.64 to 23.51)	15	18.18 (13.91 to 23.39)	16	6.9 (-19 to 41.8)	20.23 (13.34 to 31.07)	14	22.91 (16.9 to 33.43)	13	13.3 (-18 to 62.5)	14.44 (10.6 to 19.66)	15	14.6 (10.85 to 18.64)	19	1.1 (-28 to 41.5)
**Bahrain**	77.76 (66.23 to 91.01)	2	126.95 (102.53 to 154.58)	1	63.3 (23 to 103.2)	73.37 (60.74 to 93.82)	4	129.32 (100.85 to 155.58)	2	76.2 (8.4 to 126.5)	82.23 (67.21 to 101.12)	2	124.53 (99.58 to 156.5)	1	51.4 (17 to 92.3)
**Egypt**	23.3 (21.45 to 25.35)	12	33.75 (26.03 to 43.78)	9	44.8 (10.5 to 89.1)	26.84 (24.06 to 30.54)	11	42.01 (31.52 to 59.63)	9	56.5 (17.4 to 119.5)	19.76 (17.14 to 22.04)	12	29.15 (21.41 to 38.75)	8	47.5 (9.5 to 98.1)
**Iran (Islamic Republic of)**	12.46 (10.69 to 14.44)	18	21.98 (18.39 to 23.79)	12	76.4 (38.3 to 110.8)	13.84 (10.86 to 16.86)	18	24.23 (17.22 to 27.04)	11	75 (10.6 to 140.1)	10.93 (9.27 to 13.11)	18	19.84 (17.77 to 21.94)	13	81.5 (46.3 to 117.9)
**Iraq**	49.22 (39.45 to 58.61)	6	45.78 (37.07 to 54.43)	6	-7 (-28.6 to 19.8)	52.22 (40.44 to 64.76)	6	43.56 (34.55 to 54.21)	7	-16.6 (-37.6 to 12.9)	45.87 (35.77 to 57.07)	6	47.92 (38.44 to 56.39)	6	4.5 (-21.3 to 40.2)
**Jordan**	63.93 (53.77 to 74.93)	4	40.22 (34.1 to 47.54)	7	-37.1 (-50.1 to -21)	81.36 (64.14 to 99.16)	3	42.28 (34.16 to 51.74)	8	-48 (-60.4 to -29.2)	46.26 (36.74 to 57.63)	5	38.65 (30.57 to 48.41)	7	-16.4 (-39.3 to 16.4)
**Kuwait**	28.75 (25.44 to 32.25)	9	18.47 (15.26 to 22.2)	14	-35.8 (-45.5 to -24.7)	33.68 (28.96 to 38.02)	10	16.71 (13 to 20.85)	17	-50.4 (-60.1 to -38.8)	24.49 (21.28 to 28.95)	10	19.56 (15.34 to 24.82)	14	-20.1 (-36.3 to -2.1)
**Lebanon**	20.39 (17.53 to 24.43)	13	15.92 (11.38 to 20.38)	17	-21.9 (-44.4 to 2.8)	17.65 (14.5 to 21.52)	15	11.59 (8.66 to 16.07)	21	-34.3 (-52.4 to -6.1)	23.33 (19.39 to 28.36)	11	21.23 (13.25 to 29.63)	9	-9 (-44.3 to 30.8)
**Libya**	14.22 (10.45 to 17.97)	16	18.33 (13.35 to 24.07)	15	28.9 (-10 to 82.3)	15.65 (11.5 to 22.34)	16	19.72 (14.17 to 26.31)	14	26 (-13.3 to 81.6)	12.92 (9.14 to 17)	16	16.97 (10.79 to 24.72)	16	31.3 (-17.7 to 96.9)
**Morocco**	13.09 (10.39 to 18.56)	17	22.36 (17.15 to 28.34)	11	70.8 (31.5 to 112.6)	14.85 (11.03 to 24.8)	17	25.22 (19.3 to 35.99)	10	69.8 (27.9 to 125.1)	11.28 (8.77 to 14.13)	17	19.43 (14.32 to 24.06)	15	72.3 (26.1 to 122.8)
**Oman**	44.74 (34.25 to 57.2)	7	58.33 (50.42 to 66.51)	4	30.4 (-1.9 to 74)	48.45 (35.13 to 64.34)	7	54.1 (44.46 to 66)	5	11.6 (-18.7 to 65)	41.85 (32.21 to 53.18)	7	64.24 (51.47 to 76.74)	4	53.5 (6 to 114.8)
**Palestine**	53.89 (42.96 to 66.43)	5	68.84 (59.52 to 78.69)	3	27.7 (0.8 to 65.3)	56.55 (44.45 to 70.91)	5	67.01 (56.64 to 77.8)	3	18.5 (-8.7 to 56.4)	50.97 (38.72 to 65.48)	4	71.5 (60.98 to 83)	3	40.3 (5 to 88.4)
**Qatar**	111.16 (94.36 to 130.89)	1	122.07 (98.85 to 151.52)	2	9.8 (-15.1 to 41.1)	111.86 (88.74 to 138.66)	1	173.19 (134.57 to 213.1)	1	54.8 (18.9 to 100.1)	112.67 (91.11 to 137.9)	1	107.38 (83.77 to 136.35)	2	-4.7 (-33 to 33.7)
**Saudi Arabia**	25.17 (19.16 to 33.07)	11	19.51 (16.12 to 23.69)	13	-22.5 (-43.8 to 7)	26.15 (18.98 to 34.02)	12	17.62 (13.83 to 23.19)	16	-32.6 (-53.6 to 0.7)	24.66 (18.11 to 33.88)	9	20.78 (16.82 to 24.74)	11	-15.7 (-43.6 to 22.9)
**Sudan**	11.51 (8.44 to 16.41)	20	15.65 (10.59 to 21.73)	19	36 (-0.3 to 81.3)	13.01 (8.69 to 21.1)	19	16.59 (11 to 24.44)	18	27.5 (-5.5 to 72.3)	10.14 (7.6 to 14.13)	21	14.89 (9.08 to 22.53)	18	46.8 (-5.3 to 116)
**Syrian Arab Republic**	18.67 (14.4 to 23.23)	14	15.89 (12.31 to 20.76)	18	-14.9 (-36.6 to 20.4)	23.01 (17.11 to 29.4)	13	19.66 (15.19 to 26.36)	15	-14.5 (-36.6 to 26.8)	14.81 (11.38 to 18.85)	14	13.68 (10.17 to 18.05)	20	-7.6 (-36.7 to 34.6)
**Tunisia**	10.9 (8.73 to 15.01)	21	14.84 (10.71 to 19.94)	20	36.1 (-0.8 to 85.4)	11.6 (8.88 to 20.14)	21	14.13 (10.13 to 20.21)	20	21.7 (-13.1 to 73.4)	10.27 (8.01 to 12.83)	19	15.65 (10.9 to 22.08)	17	52.3 (5.8 to 117.6)
**Turkey**	40.35 (34.56 to 47.01)	8	22.73 (18.27 to 27.61)	10	-43.7 (-56.3 to -26.7)	46.19 (35.35 to 55.2)	8	24.05 (19.05 to 29.9)	12	-47.9 (-60.9 to -28.2)	32.99 (26.1 to 41.64)	8	20.77 (16.46 to 25.88)	12	-37 (-55.5 to -12.5)
**United Arab Emirates**	75.28 (58.3 to 92.75)	3	55.19 (41.41 to 70.71)	5	-26.7 (-51.7 to 3.3)	85.68 (58.34 to 114.38)	2	51.24 (37.98 to 67.48)	6	-40.2 (-60.2 to -10.2)	66.9 (50.61 to 86.36)	3	57.07 (40.37 to 75.28)	5	-14.7 (-50.4 to 27.5)
**Yemen**	11.66 (8.14 to 17.85)	19	14.18 (10.2 to 19.83)	21	21.6 (-9.7 to 62.6)	12.82 (8.2 to 21.61)	20	16.54 (10.79 to 24.42)	19	29.1 (-7.3 to 77.8)	10.26 (7.45 to 14.51)	20	11.69 (8.26 to 16.23)	21	13.9 (-16.9 to 57.2)
**DALYs**															
**Afghanistan**	997.07 (713.44 to 1373.48)	10	1567.9 (1136.29 to 2073)	7	57.3 (26.8 to 88.1)	1280.24 (841.26 to 1907.17)	8	2042.23 (1331.65 to 2841.15)	3	59.5 (24.2 to 97.1)	722.51 (547.46 to 971.76)	12	1053.36 (805.06 to 1376.27)	13	45.8 (20.8 to 69.8)
**Algeria**	641.35 (485.37 to 828.73)	16	951.02 (727.94 to 1234.63)	16	48.3 (26.4 to 71.6)	700.39 (520.52 to 932.9)	16	1046.02 (799.18 to 1365.35)	12	49.3 (23.5 to 78.8)	586.68 (438.47 to 753.82)	17	866.04 (648.73 to 1123.72)	17	47.6 (24 to 71)
**Bahrain**	2067.56 (1751.71 to 2415.78)	2	3232.51 (2622.36 to 3929.33)	1	56.3 (28.4 to 83.8)	1952.21 (1640.07 to 2383.6)	3	3200.82 (2597.54 to 3885.57)	2	64 (21.7 to 97.5)	2175.55 (1807.53 to 2616)	2	3246.6 (2649.6 to 3978.11)	1	49.2 (23.8 to 79)
**Egypt**	718.24 (637.23 to 811.6)	14	1224.68 (973.01 to 1492.76)	9	70.5 (43.2 to 102.9)	817.27 (711.54 to 942.81)	13	1343.94 (1057.53 to 1709.72)	8	64.4 (35.2 to 106)	618.6 (537.49 to 706.18)	15	1146.52 (882.87 to 1410.27)	10	85.3 (51.2 to 124.2)
**Iran (Islamic Republic of)**	516.51 (420.71 to 628.24)	19	958.11 (776.6 to 1170.16)	15	85.5 (67 to 99.8)	534 (438.41 to 648.37)	19	1002.62 (803.18 to 1235.04)	14	87.8 (59.7 to 111.8)	498.12 (398.87 to 614.42)	19	913.79 (740.01 to 1115.3)	16	83.4 (66.1 to 100.3)
**Iraq**	1544.01 (1292.48 to 1824.71)	5	1624.99 (1314.76 to 1979.27)	5	5.2 (-13.9 to 25.2)	1632.88 (1322.43 to 1978.59)	5	1598.57 (1285.3 to 1960.93)	6	-2.1 (-22.3 to 21)	1454.09 (1177.35 to 1767.01)	4	1652.18 (1339.43 to 1995.44)	6	13.6 (-8 to 39.6)
**Jordan**	1624.67 (1380.1 to 1899.1)	4	1292.88 (1069.38 to 1569.32)	8	-20.4 (-33.5 to -5.5)	1916.01 (1572.95 to 2281.48)	4	1227.99 (985.11 to 1475.6)	9	-35.9 (-48.6 to -18.9)	1332.43 (1099.03 to 1603.34)	6	1349.17 (1081.72 to 1674.91)	7	1.3 (-19.1 to 26.6)
**Kuwait**	1072.42 (877.31 to 1310.96)	9	1136.27 (841.6 to 1480.01)	11	6 (-5.9 to 17.2)	1136.18 (942.73 to 1382.58)	10	1020.52 (737.57 to 1336.31)	13	-10.2 (-23 to 1.7)	1025.76 (820.82 to 1284.76)	8	1215.68 (917.37 to 1595.55)	8	18.5 (4.9 to 31.4)
**Lebanon**	801.76 (649.51 to 990.43)	12	991.24 (737.79 to 1295.03)	14	23.6 (5.2 to 39.6)	740.76 (590.89 to 919.76)	14	860.87 (643.72 to 1142.27)	18	16.2 (-0.4 to 33.1)	864.6 (699.79 to 1074.25)	11	1149.75 (838.66 to 1510.46)	9	33 (7.8 to 57.3)
**Libya**	686.76 (531.84 to 866.67)	15	1138.79 (859.69 to 1475.17)	10	65.8 (43.5 to 92.1)	706.74 (544.9 to 899.04)	15	1154.01 (864.16 to 1481.41)	10	63.3 (37.5 to 92.4)	670.21 (509.24 to 855.89)	13	1123.99 (810.65 to 1491.76)	11	67.7 (41.2 to 96)
**Morocco**	546.75 (436.72 to 695.74)	18	1008.63 (786.89 to 1261.78)	13	84.5 (60.9 to 106.9)	587.09 (463.16 to 799.75)	17	1080.38 (835.06 to 1372.98)	11	84 (51.9 to 115.6)	505.61 (393.25 to 628.48)	18	936.05 (701.84 to 1175.73)	15	85.1 (59.8 to 110.5)
**Oman**	1248.34 (991.95 to 1505.85)	7	1618.46 (1363.58 to 1912.06)	6	29.6 (4.1 to 58.3)	1351.13 (1035.2 to 1685.78)	7	1544 (1284.1 to 1844.97)	7	14.3 (-11 to 49.2)	1176.89 (929.17 to 1453.25)	7	1713.32 (1403.51 to 2051.3)	5	45.6 (11.1 to 83.9)
**Palestine**	1398.92 (1131.05 to 1700.57)	6	1907.93 (1642.58 to 2233.13)	3	36.4 (13.4 to 65.9)	1422.55 (1150.7 to 1739.63)	6	1790.08 (1515.61 to 2122.53)	4	25.8 (3 to 55.2)	1375.92 (1078.3 to 1729.58)	5	2031.4 (1725.9 to 2395.25)	3	47.6 (17.5 to 83.4)
**Qatar**	2528.14 (2147.48 to 2948.9)	1	2975.32 (2401.18 to 3673.13)	2	17.7 (-2.4 to 40.8)	2575.2 (2107.63 to 3102.36)	1	3628.58 (2952.61 to 4381.58)	1	40.9 (15.3 to 70)	2545.47 (2071.49 to 3057.78)	1	2769.75 (2202.23 to 3468.86)	2	8.8 (-15.4 to 36.8)
**Saudi Arabia**	902.32 (710.14 to 1117)	11	1064.47 (829.68 to 1348.61)	12	18 (-5.1 to 42.3)	914.07 (722.85 to 1127.89)	11	994.63 (771.07 to 1275.59)	15	8.8 (-14.6 to 36.3)	898.95 (691.03 to 1144.79)	10	1110.36 (853.98 to 1406.95)	12	23.5 (-4.8 to 52.7)
**Sudan**	510.28 (386.47 to 656.18)	20	855.62 (643.55 to 1090.18)	19	67.7 (43.6 to 92.6)	534.22 (396.52 to 718.61)	18	852.79 (641.34 to 1101.74)	19	59.6 (30.1 to 87.6)	488.62 (372.21 to 627.52)	20	857.85 (619.19 to 1124.79)	19	75.6 (46.6 to 107.2)
**Syrian Arab Republic**	740.13 (594.84 to 903.01)	13	853.15 (646.49 to 1103.18)	20	15.3 (-3.1 to 34.8)	824.28 (662.92 to 1013.65)	12	898.17 (684.09 to 1169.51)	16	9 (-10.4 to 32.1)	663.92 (525.38 to 825.18)	14	820.15 (615.48 to 1057.33)	20	23.5 (2.5 to 46.6)
**Tunisia**	563.98 (434.18 to 720.31)	17	943.84 (713.81 to 1233.84)	17	67.4 (48 to 86.7)	533 (411.25 to 703.99)	20	851.52 (635.85 to 1121.66)	20	59.8 (37.7 to 82.8)	593.76 (446.58 to 761.73)	16	1040.95 (771.29 to 1355.65)	14	75.3 (54.9 to 98.9)
**Turkey**	1141.59 (976.14 to 1333.62)	8	869.94 (702.29 to 1073.51)	18	-23.8 (-36.5 to -9.4)	1253.44 (1016.84 to 1487.14)	9	873.17 (700.61 to 1082.39)	17	-30.3 (-43.2 to -13.1)	1011.05 (831 to 1239.86)	9	860.19 (678.27 to 1066.49)	18	-14.9 (-33.5 to 5.8)
**United Arab Emirates**	1884.22 (1558.66 to 2246.72)	3	1865.19 (1467.97 to 2332.36)	4	-1 (-23.8 to 23.8)	2032.82 (1571.38 to 2527.7)	2	1741.29 (1374.73 to 2210.97)	5	-14.3 (-35.2 to 12.1)	1765.17 (1406.61 to 2187.74)	3	1909.75 (1484.31 to 2404)	4	8.2 (-22.2 to 41.5)
**Yemen**	467.3 (354.49 to 626.37)	21**/**	676.5 (512.32 to 878.75)	21	44.8 (21.8 to 68.9)	494.8 (357.21 to 695.47)	21	736.4 (535.37 to 978.65)	21	48.8 (19.4 to 77.3)	439.11 (330.6 to 584.07)	21	614.95 (453.83 to 807.47)	21	40 (17 to 64.7)

*ASR* Age-standardized rate; Data in parentheses are 95% Uncertainty Intervals (95% UIs)

Change in the ranking of countries (range from 1 (the highest estimate) to 21 (the lowest estimate)) in 2019 vs 1990 were classified by three groups:

Upward

Monotone

Downward

Between 1990 and 2019, total new cases of T2DM increased from 441,642 (405,349 to 481,563) to 2,007,270 (1,842,502 to 2,191,938) in the region. Population growth change, age structure change, and incidence rate change regardless of sex accounted for 76.4%, 70.8%, and 207.3% increase in the total number of new cases, respectively (Table 4). The most changes in new cases were observed in Qatar (1713%). The proportions of incidence rate change, population growth, and age-structure change in the diagnosed new cases were 909.8%, 543.5%, and 259.5%, respectively.

**Table 4:** Decomposition analysis of new cases between 1990 and 2019 at regional and country levels

Location		New cases		Expected new cases in 2019		% 1990—2019 new cases change cause			% 1990—2019 new cases overall change
		**1990**	**2019**	**Population growth**	**Population growth + Aging**	**Population growth**	**Age structure change**	**Incidence rate change**	
**North Africa and Middle East**		441,642	2,007,270	779,166	1,091,862	76.4%	70.8%	207.3%	354.5%
**Country**	Afghanistan	18,398	96,531	61,672	51,290	235.2%	-56.4%	245.9%	424.7%
	Algeria	33,340	159,510	55,183	86,108	65.5%	92.8%	220.2%	378.4%
	Bahrain	1313	14,376	3729	7776	184%	308.2%	502.6%	994.7%
	Egypt	53,140	251,707	94,520	114,302	77.9%	37.2%	258.6%	373.7%
	Iran (Islamic Republic of)	61,449	291,482	88,487	152,041	44%	103.4%	226.9%	374.3%
	Iraq	30,355	145,024	72,659	97,396	139.4%	81.5%	156.9%	377.8%
	Jordan	5950	38,421	18,352	27,848	208.4%	159.6%	177.7%	545.7%
	Kuwait	4253	24,117	10,701	16,513	151.6%	136.7%	178.8%	467%
	Lebanon	6092	20,237	9630	12,243	58.1%	42.9%	131.2%	232.2%
	Libya	6200	33,010	9856	17,236	59%	119%	254.4%	432.5%
	Morocco	31,322	129,305	44,516	65,595	42.1%	67.3%	203.4%	312.8%
	Oman	2707	14,921	6385	8948	135.9%	94.7%	220.7%	451.3%
	Palestine	2769	15,518	6630	8699	139.4%	74.7%	246.3%	460.5%
	Qatar	1319	23,920	8491	11,915	543.5%	259.5%	909.8%	1712.9%
	Saudi Arabia	27,124	169,200	60,405	98,705	122.7%	141.2%	259.9%	523.8%
	Sudan	22,706	100,586	45,874	50,833	102%	21.8%	219.1%	343%
	Syrian Arab Republic	16,251	51,642	18,262	32,941	12.4%	90.3%	115.1%	217.8%
	Tunisia	13,747	55,022	18,849	30,323	37.1%	83.5%	179.7%	300.2%
	Turkey	88,857	258,309	120,947	190,403	36.1%	78.2%	76.4%	190.7%
	United Arab Emirates	4008	63,860	19,786	38,567	393.7%	468.6%	631%	1493.3%
	Yemen	10,045	48,534	23,049	28,513	129.5%	54.4%	199.3%	383.2%

**Fig. 1:**
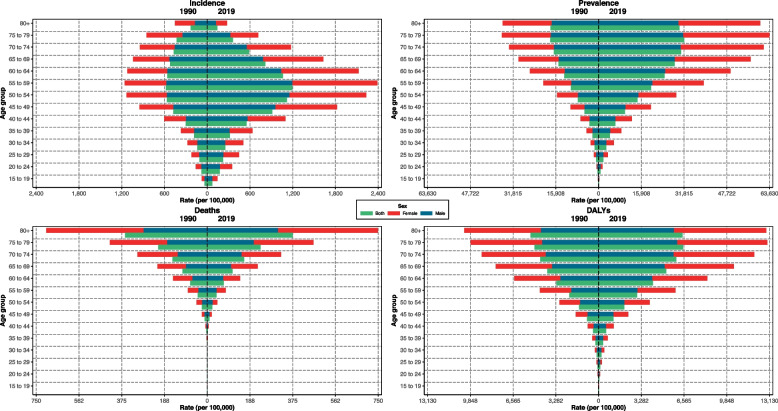
Burden of T2DM by age groups and sex in 1990 compared to 2019

The incidence and prevalence of T2DM was increased in 2019 for all age groups. There were no considerable differences between both sexes in the region. The most incidence rate per 100,000 people was observed in patients aged 55 to 59 years old while the most prevalence was dedicated to whom with 75–79 years old in both sexes. Although the incidence and prevalence of T2DM in the countries did not vary between two genders, the number of deaths and DALYs in females was greater than males in both 1990 and 2019 (Fig. 1). Totally, the most estimated death rate in patients with T2DM was observed in those with 80 years of age and older.

Regarding SDI, generally this index was improved in all countries through 30 years. Oman and Afghanistan experienced the highest and lowest changes in SDI in 2019 compared to 1990 (Supplementary Table 5). Comparison of age-standardized mortality rate in four years (1990, 2000, 2010, and 2019) based on the SDI of 21 countries revealed that countries with higher SDI seem to have higher age-standardized mortality rates (Supplementary Fig. 1).

Age-standardized MIR rate showed downward trend in the region and approximately all countries from 1990 to 2019. Age-standardized MIR rate in ten countries regardless of sex were upper than the region. However, after considering sex, we found different trends between 1990 and 2019. In 2019, this rate in Qatari female was the greatest (0.21) compare to other countries. Regarding men, we found six countries with age-standardized MIR rate more than 0.1 and the greatest one was related to Bahrain in 2019 (Supplementary Fig. 2).

### Attributable burden to risk factors

Three attributed risk factors (Metabolic, Behavioral, and environmental/ occupational risk factors) to deaths and DALYs had substantially different geographical patterns. In comparison to 1990, different patterns in most countries were observed in DALYs and death attributed to the leading risk factors in 2019 (Supplementary Fig. 3).

Percent of attributed age-standardized rate to the leading risk factors did not show considerable changes from 1990 to 2019. In 2019, the highest percentage of deaths were attributed to metabolic risk factors (100 for high fasting blood glucose, 56.4%; [42.8 to 69.8] for high BMI). Behavioral risk factors (44.3%; [37.2 to 51.0] and environmental/occupational (30.2%; [24.7 to 36.0] were in the next ranks. Among attributed risk factors to death (age-standardized rate) of T2DM, high body mass index (BMI) (56.4%; [42.8 to 69.8]), low physical activity (15.5%; [9.0 to 22.8], and ambient particulate matter pollution (20.9%; [15.2 to 26.2]) were prominent attributable deaths rate to the T2DM in the region. Among attributed death rate of dietary risk factors, diet high in processed meat (21.4%; [11.0 to 35.6]), and diet high in sugar-sweetened beverages (15.6%; [2.3 to 32.2]) showed the greatest percentage changes during these 30 years. The greatest changes among 17 age-standardized attributable burden to mortality rate of T2DM was related to high temperature (50.0%; [-70.0 to 258.2]) (Table 5).

**Table 5:** Percent of attributed age-standardized rate (%) to risk factors with percent changes between 1990 and 2019 for both sexes in the region

Risk factor	Percent of attributed age-standardized rate per 100,000 (%)					
	**Deaths**			**DALYs**		
	**1990**	**2019**	**% Change (1990 to 2019)**	**1990**	**2019**	**% Change (1990 to 2019)**
**Metabolic risks**	100	100	0	100	100	0
High fasting plasma glucose	100	100	0	100	100	0
High body-mass index	46.3 (32.4 to 60.4)	56.4 (42.8 to 69.8)	21.7 (12.7 to 37)	54.9 (40.9 to 68)	66.9 (53.7 to 77.9)	21.8 (13.3 to 35.8)
**Behavioral risks**	45.8 (38.6 to 52.7)	44.3 (37.2 to 51)	-3.3 (-5.7 to -0.9)	46.3 (39.2 to 53.1)	45.2 (38.4 to 51.9)	-2.4 (-4.6 to -0.3)
Diet high in processed meat	2.1 (0.9 to 2.6)	2.5 (1.2 to 3.1)	21.4 (11 to 35.6)	2.2 (1 to 2.7)	2.8 (1.3 to 3.3)	23.7 (14.2 to 38)
Diet high in red meat	3.4 (0.9 to 5.5)	3.4 (0.8 to 5.5)	-0.5 (-4.7 to 5.5)	3.7 (1 to 5.9)	3.7 (0.9 to 5.8)	-0.3 (-3.0 to 3.9)
Diet high in sugar-sweetened beverages	2.5 (1.3 to 3.5)	2.9 (1.5 to 4)	15.6 (2.3 to 32.2)	2.6 (1.4 to 3.6)	3.2 (1.6 to 4.3)	20.6 (8.5 to 36)
Diet low in fiber	1.5 (0.5 to 2.4)	1.5 (0.6 to 2.4)	-0.1 (-8.4 to 11.3)	1.5 (0.5 to 2.5)	1.4 (0.6 to 2.4)	-3.7 (-9.1 to 4.9)
Diet low in fruits	2.7 (1.1 to 5.1)	2.1 (0.9 to 4)	-20.3 (-28.4 to -8.4)	2.9 (1.2 to 5.3)	2.3 (1 to 4.3)	-20.9 (-26.8 to -13.3)
Diet low in nuts and seeds	2 (0.3 to 4.2)	1.8 (0.3 to 3.7)	-12.0 (-19.4 to 6.6)	2.1 (0.3 to 4.3)	1.7 (0.3 to 3.7)	-15.0 (-22.2 to -3)
Diet low in whole grains	8.2 (4.6 to 10.9)	8 (4.4 to 10.6)	-3.0 (-6.6 to -0.6)	8.2 (4.6 to 10.8)	7.9 (4.3 to 10.5)	-3.4 (-6.5 to -1.8)
Low physical activity	14.5 (8.1 to 21.6)	15.5 (9 to 22.8)	7.4 (3.5 to 13.6)	13.2 (7.1 to 20.4)	14.5 (8.1 to 22)	10.2 (5.9 to 17)
Smoking	9.7 (8 to 11.8)	8.1 (6.6 to 9.7)	-16.7 (-28.1 to -5.9)	11.4 (9.6 to 13.7)	9.7 (8.1 to 11.5)	-15.2 (-23.2 to -7.4)
Secondhand smoke	11.7 (4.6 to 17.7)	10 (3.9 to 15.2)	-14.9 (-18 to -11.9)	11.6 (4.5 to 17.5)	10.3 (4 to 15.7)	-10.6 (-13.1 to -8.2)
Alcohol use	-0.3 (-0.6 to -0.1)	-0.2 (-0.5 to 0)	-29.2 (-75.8 to 9.5)	-0.3 (-0.6 to 0.1)	-0.2 (-0.5 to 0.1)	-26.4 (-145.0 to 35.8)
**Environmental/occupational risks**	30.4 (24.4 to 36.4)	30.2 (24.7 to 36)	-0.5 (-7.8 to 5.2)	27.4 (21.3 to 33.5)	26 (19.9 to 31.7)	-5.3 (-13.8 to -0.2)
Ambient particulate matter pollution	15.3 (10.7 to 20.1)	20.9 (15.2 to 26.2)	36.8 (21.9 to 62.7)	15.1 (10.6 to 19.9)	20.8 (15 to 26.1)	37.9 (23.0 to 62.5)
Household air pollution from solid fuels	7.3 (4.4 to 11.7)	1.4 (0.8 to 2.2)	-81.0 (-86 to -74.9)	7.5 (4.6 to 12.1)	1.5 (0.9 to 2.3)	-80 (-83.8 to -75.2)
High temperature	1.9 (0 to 3.4)	2.8 (1 to 4.5)	50.0 (-70.0 to 258.2)	1.2 (0 to 2.3)	1.4 (0.5 to 2.4)	18.2 (-53.1 to 188.1)
Low temperature	8.3 (5.4 to 11.3)	7.5 (4.4 to 10.6)	-9.7 (-25.1 to 3)	5 (3.1 to 7.2)	3.4 (1.8 to 5.1)	-32.9 (-47.8 to -20.9)

Data in parentheses are 95% uncertainty interval (UI); *DALYs* Disability-Adjusted Life Years

**Fig. 2:**
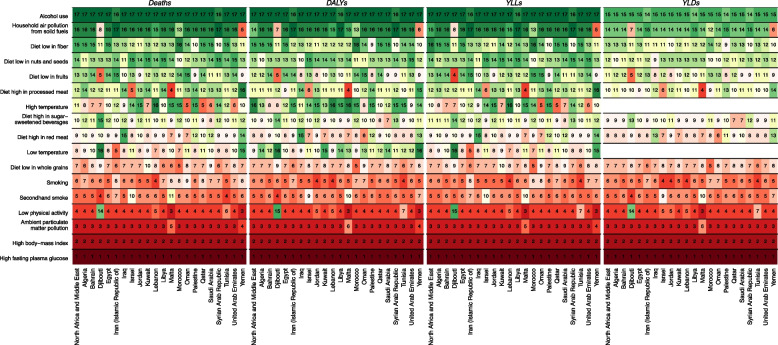
The ranking of age-standardized attributed burden to 17 risk factors of T2DM at region and country levels, 2019

We observed that high FPG ranked first and high BMI ranked second among 17 risk factors for age-standardized deaths and DALYs rate for region and all countries in 2019 (Fig. 2). The third rank in all countries except Malta and Yemen was attributed to ambient particulate matter pollution.

## Discussion

Our findings indicated that the prevalence and incidence of T2DM increased from 1990 to 2019. No considerable differences were found between two genders in the prevalence and incidence of T2DM; however, deaths and DALYs in females were greater than men. Qatar ranked the first in both incidence and prevalence of T2DM among the countries in the region. However, the report by the International Diabetes Federation (IDF) in 2019 showed that Pakistan ranked the first among the countries in the region regarding the numbers of people with diabetes [17]. During these years in some countries such as Qatar, Kuwait, and UAE rapid economic growth and urbanization have occurred that affected life expectancy, while in some countries such as Palestine, Iraq, Lebanon, and Afghanistan due to some dramatic political changes, war, and sanction controlling non-communicable diseases (NCDs) including T2DM was faced some problems. Although Qatar ranked first in the incidence and prevalence of T2DM, reported deaths and DALYs in this country were relatively low compared to the numbers of countries. Relatively low deaths and DALYs for T2DM may be partly explained by Qatar national response to diabetes. Action plans for obesity, diabetes, and/or physical activity are also provided in some countries such as Iran, Bahrain, Qatar, Kuwait, UAE. These programs can take part in the prevention and the management of diabetes There is also national registries for diabetes and national surveys for identification of risk factors in several countries including Bahrain, Qatar, Iran, Jordan, and Kuwait [18]. However, due to the remarkable number of individuals suffered from T2DM in some countries controlling and monitoring the status with high quality services are difficult.

Our findings regarding greater deaths in diabetic females were similar to those have reached so far [19, 20]. A systematic review and meta-analysis by Xu et al., demonstrated that all-cause mortality in females with diabetes was higher than men [20]. Although it is not fully understood why the number of deaths in women was higher than men, several possible mechanisms can explain a part of this. Based on evidence, sex differences can play a pivotal role in the management of diabetes and mortality rates between males and females. Diabetic men vs. women may be diagnosed earlier and men with diabetes are likely to receive more suitable therapeutic interventions and greater comprehensive care [20]. This factor along with more attention of men to self-care and adherence to therapies vs. women can affect the status of disease and complications [21]. It has been also indicated that diabetic men more than women received recommended care processes [22]. Importantly, there is compelling evidence suggesting that men, when adhering to the same treatment plan, tend to attain a higher percentage of treatment goals for managing diabetes and mitigating other mortality risk factors. This difference in outcomes can be attributed to a range of factors, including variances in biological characteristics, hormonal influences, and behavioral tendencies [19].

In the Present study, we also found that the increase in the incidence of T2DM cannot be fully explained by population growth and aging. It was affected mostly by age-structure change. The most prevalence and incidence during these years were observed in those aged 75 to 80 and 50 to 60 years old, respectively. Mortality, DALY, and YLD rates increased following this growth in the region. However, the number of deaths decreased in less than one-third countries in the region.

The increased incidence and prevalence of T2DM and mortality can be attributed to several factors, mainly a combination of metabolic and environmental factors. Urbanization, modernization, and industrialization in the countries of the region can result in changes in lifestyle such as physical activity and dietary habits. These factors can induce overweight/obesity and metabolic disorders, and provide a ground for developing T2DM [23].

Our findings confirmed the previous evidence on the roles of metabolic, behavioral, and environmental factors in the mortality from T2DM. Based on our findings, the most important risk factor for T2DM mortality is due to high Fasting Blood Glucose [FPG]. High BMI, air pollution and low physical activity were ranked next.

Although the proportion of about all risk factors in the deaths of T2DM did not change considerably during these years, high intake of processed meat and sugar sweetened beverages participated in the most portions of deaths from T2DM. Thus, primary prevention must be paid specific attention by policy makers and clinicians. National preventive programs with the aims of promoting healthy lifestyle and considering various dimensions including social, economic, cultural, and regional characteristics of each country in the region must be given high priority in the health care agenda.

Public educational programs and tax policy interventions for unhealthy foods such as processed meat and sugar-sweetened beverages (SSB) can be helpful to control diabetes and mortality related to these chronic diseases. Based on estimation in Germany, for instance, combined tax interventions containing a 50% price increase for sugar-sweetened beverages, red meat and its products, and tobacco in 2020 can lead to a 0.95% decrease in the prevalence of T2DM in 2040 that corresponds to 640,000 fewer cases of T2DM [24]. However, such interventions alone are not sufficient to attenuate the incidence and prevalence of T2DM in years ahead [24]. In a systematic review study, Zhou et al., indicated that lifestyle interventions in high-risk populations were cost-effective to prevent diabetes from a health care system or a societal perspective. Among population-based interventions, taxing SSB was cost saving from both governmental and health care system perspectives. However, it revealed that findings for community-based education programs and modifications to build environment are inconsistent [25]. One of WHO's program called “best buys” contained some strategies such as raising taxes on unhealthy foods that are implemented in some countries to tackle sharp increase in the cases with diabetes and other NCDs [18]. In the present study, we shed light on the ranking of risk factors attributed to mortality from T2DM for each country in the region. Findings can be helpful for policy makers to set priorities and allocate resources for the prevention of this NCD in real-world settings.

Although our findings showed that high BMI was one of the main factors for mortality in both men and women with T2DM in the region, a systematic review and meta-analysis showed an association between high BMI and all-cause mortality in T2DM in women only [26]. In this systematic review and meta-analysis, no subgroup analysis was performed based on location. Thus, it may be due to different patterns in each region across the world.

Changes in the proportion of high temperature in mortality rate of T2DM increased by 50% in 2019 compared to 1990. Temperature is a new environmental risk factor introduced in GBD 2019. Recent studies have shown a link between high temperature and the risk of developing diabetes and gestational diabetes.

Temperature is a new environmental risk factor provided in GBD 2019. Recent studies revealed the association between high temperature and the risk of diabetes and gestational diabetes mellitus [27–29]. This link can originate through a multifactorial pathway containing social, biological, geophysical, environmental, and economic factors [30].

Apart from multi-dimensional transition (e.g., a shift from rural to urban, socioeconomic development, adherence to unhealthy and sedentary lifestyle) in the region, several countries in the Middle East encounter into war, political instability, forced migration, climate change, and social instability. These issues can lead to a significant challenge in health care and social services [18]. Although countries in this region in most cases have similarities including religion, development level, ethnicity, urbanization, and level of health care, there are differences in culture, income, and level of healthcare that involve in the quality of care and the burden of diseases including T2DM. In addition, many countries have developed national diabetes treatment guidelines (specific strategy and standard protocol). But some countries such as Libya and Yemen have not yet provided a standard protocol.

In addition, based on the last accessible report, action plans for diabetes (e.g. in Egypt, Libya, Oman, Sudan, Yemen), weight management (in most countries), and physical activity have not been existed in several countries. National diabetes registry and national risk factor survey in which glycemic status have been considered are other examples of implementations to collect data on diabetes status that have not been reported in several countries [18].

Other issues are related to knowledge gaps and poor attitude in diabetes self-management, poor compliance with healthy life style recommendations and medications, lack of social support and no access to healthy environment that can involve in increasing the burden of T2DM in the region [18]. Therefore, health care providers prepare appropriate education and other intervention strategies to help prevent and control diabetes, taking into account local sociocultural practices, gender, and age.

Based on the current status of primary care in the management of diabetes in the region more focus should be done to strengthen and improvement the delivery of health servicesaa [31].

## Conclusion and recommendations

The burden of T2DM, as measured by the number of deaths, DALYs, and YLDs, continues to increase in the region from 1990 to 2019. The age-standardized incidence rate of T2DM has also shown an upward trend in many areas. The burden of T2DM attributed to modifiable risk factors remains high, highlighting the need for targeted public health programs focused on prevention, screening, and monitoring of patients with type 2 diabetes.

To reduce the growth and disease burden of T2DM in the region, it is important to address modifiable risk factors, promote healthy aging throughout life, improve healthcare services, and aim to decrease disability and premature death associated with T2DM. The study's strength lies in considering the burden and risk factors across all 21 countries in the region. However, the study has some limitations, such as not accounting for psychological factors like stress, which play a significant role in diabetes management. Additionally, it does not include epidemiological information for gestational diabetes mellitus and its associated risk factors. It is recommended that future GBD protocols consider the effects of the COVID-19 pandemic.

### Supplementary Information

**Additional file 1:** **Supplementary Table 1.** Data input of the North Africa and Middle East countries.

**Additional file 2:** **Supplementary Table 2.** T2DM burden by all ages (number and rate) and age-standardized rate in 1990 and 2019 with percent changes between 1990 and 2019 in the region countries.

**Additional file 3:** **Supplementary Table 3.** Burden and ranking of region countries based on the age-standardized YLLs and YLDs rates in 1990 and 2019 with percent change.

**Additional file 4:** **Supplementary Table 4.** Annual percent change of the age-standardized rates in the region and its countries by the measures and sex.

**Additional file 5:** **Supplementary Table 5.** Socio-demographic index of the region countries during 1990 to 2019.

**Additional file 6:** **Supplementary Figure 1.** Ordering of the region countries based on the age-standardized mortality rate and colored based on the socio-demographic index in 1990, 2000, 2010, and 2019.

**Additional file 7:** **Supplementary Figure 2.** Time trend of mortality-to-incidence ratio in the region and its countries by sex, 1990 to 2019.

**Additional file 8:** **Supplementary Figure 3.** Geographical distribution of attributed burden to T2DM risk factors by measures in 1990 and 2019.

## Data Availability

The dataset used in this study is available in the GBD compare tool (https://vizhub.healthdata.org/gbd-compare). We inform consent from all subjects and/or their legal guardian(s) for publication of identifying information/images in an online open-access publication. It is available and can be shared, if requested.

## References

[1] Sanches JM, Zhao LN, Salehi A, Wollheim CB, Kaldis P (2023). Pathophysiology of type 2 diabetes and the impact of altered metabolic interorgan crosstalk. FEBS J.

[2] Walker RJ, Gebregziabher M, Martin-Harris B, Egede LE (2015). Understanding the influence of psychological and socioeconomic factors on diabetes self-care using structured equation modeling. Patient Educ Couns.

[3] Saeedi P, Petersohn I, Salpea P, Malanda B, Karuranga S, Unwin N (2019). Global and regional diabetes prevalence estimates for 2019 and projections for 2030 and 2045: results from the International Diabetes Federation Diabetes Atlas. Diabetes Res Clin Pract.

[4] Saeedi P, Salpea P, Karuranga S, Petersohn I, Malanda B, Gregg EW (2020). Mortality attributable to diabetes in 20–79 years old adults, et al 2019 estimates: results from the International Diabetes Federation Diabetes Atlas. Diabetes Res Clin Pract.

[5] Moradi-Lakeh M, Forouzanfar MH, El Bcheraoui C, Daoud F, Afshin A, Hanson SW (2017). High fasting plasma glucose, diabetes, and its risk factors in the eastern mediterranean region, 1990–2013: findings From the Global Burden of Disease Study 2013. Diabetes Care.

[6] Esteghamati A, Larijani B, Aghajani MH, Ghaemi F, Kermanchi J, Shahrami A (2017). Diabetes in Iran: prospective analysis from first nationwide diabetes report of National Program for Prevention and Control of Diabetes (NPPCD-2016). Sci Rep.

[7] Ali NSM, Allela OQ, Salih HM, Ahmed IH (2019). Prevalence of Type 2 Diabetes Associated Complications in Kurdistan Region Iraq. J Basic Clin Pharmacol.

[8] Al Mansour MA (2020). The prevalence and risk factors of type 2 diabetes mellitus (DMT2) in a semi-urban Saudi population. Int J Environ Res Public Health.

[9] Mirahmadizadeh A, Fathalipour M, Mokhtari AM, Zeighami S, Hassanipour S, Heiran A (2020). The prevalence of undiagnosed type 2 diabetes and prediabetes in Eastern Mediterranean region (EMRO): a systematic review and meta-analysis. Diabetes Res Clin Pract.

[10] Khan Y, Hamdy O. Type 2 diabetes in the middle east and north africa (MENA). Diabetes Mellitus in Developing Countries and Underserved Communities: Springer; 2017. p. 49–61.

[11] Institute for Health Metrics and Evaluation. Protocol for the global burden of diseases, injuries, and risk factors study (GBD). Version 4.0.Available at: http://www.healthdata.org/gbd/about/protocol. 2020.

[12] Murray CJ, Aravkin AY, Zheng P, Abbafati C, Abbas KM, Abbasi-Kangevari M (2020). Global burden of 87 risk factors in 204 countries and territories, 1990–2019: a systematic analysis for the Global Burden of Disease Study 2019. The lancet.

[13] Vos T, Lim SS, Abbafati C, Abbas KM, Abbasi M, Abbasifard M (2020). Global burden of 369 diseases and injuries in 204 countries and territories, 1990–2019: a systematic analysis for the Global Burden of Disease Study 2019. The Lancet.

[14] Keykhaei M, Masinaei M, Mohammadi E, Azadnajafabad S, Rezaei N, Moghaddam SS (2021). A global, regional, and national survey on burden and Quality of Care Index (QCI) of hematologic malignancies; global burden of disease systematic analysis 1990–2017. Exp Hematol Oncol.

[15] Fitzmaurice C, Dicker D, Pain A, Hamavid H, Moradi-Lakeh M, MacIntyre MF (2015). The global burden of cancer 2013. JAMA Oncol.

[16] Stevens GA, Alkema L, Black RE, Boerma JT, Collins GS, Ezzati M (2016). Guidelines for accurate and transparent health estimates reporting: the GATHER statement. PLoS Med.

[17] International Diabetes Federation. Diabetes in MENA. Available at: https://www.idf.org/our-network/regions-members/middle-east-and-north-africa/diabetes-in-mena.html. 2019.

[18] Al Busaidi N, Shanmugam P, Manoharan D (2019). Diabetes in the Middle East: government health care policies and strategies that address the growing diabetes prevalence in the Middle East. Curr Diab Rep.

[19] Peters SA, Woodward M (2018). Sex differences in the burden and complications of diabetes. Curr DiabRep.

[20] Xu G, You D, Wong L, Duan D, Kong F, Zhang X (2019). Risk of all-cause and CHD mortality in women versus men with type 2 diabetes: a systematic review and meta-analysis. Eur J Endocrinol.

[21] Mansyur CL, Rustveld LO, Nash SG, Jibaja-Weiss ML (2015). Social factors and barriers to self-care adherence in Hispanic men and women with diabetes. Patient Educ Couns.

[22] Kalyani RR, Lazo M, Ouyang P, Turkbey E, Chevalier K, Brancati F (2014). Sex differences in diabetes and risk of incident coronary artery disease in healthy young and middle-aged adults. Diabetes Care.

[23] Ramachandran A, Snehalatha C, Shetty AS, Nanditha A (2012). Trends in prevalence of diabetes in Asian countries. World J Diabetes.

[24] Tönnies T, Heidemann C, Paprott R, Seidel-Jacobs E, Scheidt-Nave C, Brinks R (2021). Estimating the impact of tax policy interventions on the projected number and prevalence of adults with type 2 diabetes in Germany between 2020 and 2040. BMJ Open Diabetes Res Care.

[25] Zhou X, Siegel KR, Ng BP, Jawanda S, Proia KK, Zhang X (2020). Cost-effectiveness of diabetes prevention interventions targeting high-risk individuals and whole populations: a systematic review. Diabetes Care.

[26] Zaccardi F, Dhalwani NN, Papamargaritis D, Webb DR, Murphy GJ, Davies MJ (2017). Nonlinear association of BMI with all-cause and cardiovascular mortality in type 2 diabetes mellitus: a systematic review and meta-analysis of 414,587 participants in prospective studies. Diabetologia.

[27] Booth GL, Luo J, Park AL, Feig DS, Moineddin R, Ray JG (2017). Influence of environmental temperature on risk of gestational diabetes. CMAJ.

[28] Blauw LL, Aziz NA, Tannemaat MR, Blauw CA, de Craen AJ, Pijl H (2017). Diabetes incidence and glucose intolerance prevalence increase with higher outdoor temperature. BMJ Open Diabetes Res Care.

[29] Vasileiou V, Kyratzoglou E, Paschou SA, Kyprianou M, Anastasiou E (2018). The impact of environmental temperature on the diagnosis of gestational diabetes mellitus. Eur J Endocrinol.

[30] Cuschieri S, Agius JC. The interaction between diabetes and climate change–A review on the dual global phenomena. Early Hum Dev. 2020:105220. 10.1016/j.earlhumdev.2020.105220 33039261

[31] Alhyas L, Nielsen JDJ, Dawoud D, Majeed A (2013). Factors affecting the motivation of healthcare professionals providing care to Emiratis with type 2 diabetes. JRSM short reports.

