# Supplementary material for: Burden of type 2 diabetes mellitus and its risk factors in North Africa and the Middle East, 1990–2019: findings from the Global Burden of Disease study 2019
Source: BMC Public Health. 2024 Jan 5;24:98. doi: 10.1186/s12889-023-16540-8 (PMC10768242; doi:10.1186/s12889-023-16540-8)
Supplement: Supplementary file 1 — Additional file 1: Supplementary Table 1. Data input of the North Africa and Middle East countries. [file 12889_2023_16540_MOESM1_ESM.pdf]

Data type	Secondary data type	Coverage type	Geography	Time period covered	Suggested citation
Survey	Cross-sectional, Household, Individual, Interview, Nationally representative, Subnationally representative, Urban-rural representative	Country	Afghanistan	08/2012 to 12/2012	Indian Institute of Health Management Research (IIHMR), Johns Hopkins University, Ministry of Public Health (Afghanistan). Afghanistan Health Survey 2012.
Survey	Cross-sectional, Household, Interview, Subnationally representative, Urban-rural representative, Verbal autopsy	Country	Afghanistan	07/2015 to 12/2015	Ministry of Public Health (Afghanistan), Royal Tropical Institute. Afghanistan Health Survey 2015.
Survey	Community, Cross-sectional, Household, Individual, Interview, Nationally representative, Urban-rural representative, Verbal autopsy	Country	Afghanistan	04/2010 to 12/2010	Central Statistics Organization (Afghanistan), ICF Macro, Indian Institute of Health Management Research (IIHMR), Ministry of Public Health (Afghanistan), World Health Organization Regional Office for the Eastern Mediterranean (EMRO-WHO). Afghanistan Special Demographic and Health Survey 2010. Fairfax, United States of America: ICF International.
Survey	Household, Individual, Nationally representative	Country	Afghanistan	02/2018 to 10/2018	Ministry of Public Health (Afghanistan), World Health Organization (WHO). Afghanistan STEPS Noncommunicable Disease Risk Factors Survey 2018. Geneva, Switzerland: World Health Organization (WHO).
Survey	Household	Subnational	Algeria, Mostaganem, S√©tif	01/2003 to 12/2003	Ministry of Health, Population and Hospital Reform (Algeria), World Health Organization (WHO). Algeria - S√©tif and Mostaganem STEPS Noncommunicable Disease Risk Factors Survey 2003.
Report	Verbal autopsy	Country	Algeria	01/2002 to 12/2002	Epidemiological Transition and Health Impact in North Africa (TAHINA), Ministry of Health and Population (Algeria), National Institute of Public Health (Algeria). Algeria - Study of Causes of Death, TAHINA 2002. Alger, Algeria: National Institute of Public Health (Algeria), 2008.
Survey	Household	Country	Algeria	09/2002 to 01/2003	National Office of Statistics (Algeria), Ministry of Health, Population and Hospital Reform (Algeria), League of Arab States. Algeria Family Health Survey 2002-2003.
Survey	Cross-sectional, Household, Individual, Interview, Nationally representative, Urban-rural representative	Country	Algeria	03/2006 to 06/2006	Joint United Nations Program on HIV/AIDS (UNAIDS), Ministry of Health, Population and Hospital Reform (Algeria), National Office of Statistics (Algeria), United Nations Children's Fund (UNICEF), United Nations Population Fund (UNFPA). Algeria Multiple Indicator Cluster Survey 2006.
Survey	Cross-sectional, Household, Individual, Interview, Nationally representative, Subnationally representative, Urban-rural representative	Country	Algeria	10/2012 to 01/2013	Ministry of Health and Population (Algeria), United Nations Children's Fund (UNICEF). Algeria Multiple Indicator Cluster Survey 2012-2013. New York, United States of America: United Nations Children's Fund (UNICEF), 2018.
Survey	Cross-sectional, Household, Individual, Interview, Urban-rural representative	Country	Algeria	06/2005 to 07/2005	National Institute of Public Health, Ministry of Health, Population, and Hospital Reform (Algeria). Algeria National Health Survey 2005.
Survey	Household	Subnational	Algeria	11/2016 to 06/2017	Ministry of Health, Population and Hospital Reform (Algeria), World Health Organization (WHO). Algeria STEPS Noncommunicable Disease Risk Factors Survey 2016-2017.
Scientific literature	NA	NA	Algeria	01/1973 to 12/2017	Touhami M, Zennaki A, Bouchetara A, Naceur M, Aoui A, Gharnouti M, Latroch C, Bouziane-Nedjadi K, Boudraa G. Epidemiological Evolution of Type 1 Diabetes in Children: Data from the Register of the Department of Oran, Algeria, 1973-2017. Rev Epidemiol Sante Publique. 2019; 67(6): 369-374.
Scientific literature	NA	NA	Algeria, Argentina, Australia, Austria, Barbados, Belgium, Brazil, Bulgaria, Canada, Chile, China, Colombia, Cuba, Denmark, Dominica, Estonia, Finland, France, Germany, Greece, Hungary, Israel, Italy, Japan, Kuwait, Latvia, Lithuania, Luxembourg, Mauritius, Mexico, Netherlands, New Zealand, Norway, Pakistan, Paraguay, Peru, Poland, Portugal, Romania, Russian Federation, Slovakia, Slovenia, Spain, Sudan, Tunisia, United Kingdom, United States of America, Puerto Rico, United States Virgin Islands, Uruguay, Venezuela (Bolivarian Republic of)	01/2000 to 12/2000	Karvonen M, Viik-Kajander M, Moltchanova E, Libman I, LaPorte R, Tuomilehto J. Incidence of childhood type 1 diabetes worldwide. Diabetes Mondiale (DiaMond) Project Group. Diabetes Care. 2000; 23(10): 1516,Äì26.

Data type	Secondary data type	Coverage type	Geography	Time period covered	Suggested citation
Scientific literature	NA	NA	Algeria	01/2001 to 12/2001	Temmar M, Labat C, Benkhedda S, Charifi M, Thomas F, Bouafia MT, Bean K, Darne B, Safar ME, Benetos A. Prevalence and determinants of hypertension in the Algerian Sahara. J Hypertens. 2007; 25(11): 2218-26.
Scientific literature	NA	Country	Algeria, Argentina, Bahrain, Bangladesh, China, Egypt, India, Indonesia, Iran (Islamic Republic of), Jordan, Kuwait, Libya, Malaysia, Mexico, Morocco, Pakistan, Philippines, Qatar, Russian Federation, Saudi Arabia, Singapore, Republic of Korea, Taiwan (Province of China), Tunisia, Turkiye, United Arab Emirates, Yemen	01/2009 to 12/2010	Litwak L, Goh SY, Hussein Z, Malek R, Prusty V, Khamseh ME. Prevalence of diabetes complications in people in type 2 diabetes mellitus and its association with baseline characteristics in the multinational A1chieve study . Diabetol Metab Syndr. 2013; 5(1): 57.
Survey	Household, Individual, Interview	Country	Bahrain	10/1995 to 12/1995	Ministry of Health (Bahrain), Council of Health Ministers of GCC States. Bahrain Family Health Survey 1995. Manama, Bahrain: Ministry of Health (Bahrain).
Report	Discharge, Inpatient, Outpatient	Country	Bahrain	01/1996 to 12/2000	Ministry of Health (Bahrain). Bahrain Health Statistics 2000. Juffair, Bahrain: Ministry of Health (Bahrain).
Report	Epi surveillance, Subnationally representative	Country	Bahrain	01/2009 to 12/2013	Ministry of Health (Bahrain). Bahrain Health Statistics 2013. Juffair, Bahrain: Ministry of Health (Bahrain).
Report	Epi surveillance	Country	Bahrain	01/2011 to 12/2015	Ministry of Health (Bahrain). Bahrain Health Statistics 2015. Juffair, Bahrain: Ministry of Health (Bahrain).
Report	Epi surveillance	Country	Bahrain	01/2012 to 12/2016	Ministry of Health (Bahrain). Bahrain Health Statistics 2016. Juffair, Bahrain: Ministry of Health (Bahrain).
Report	Discharge, Epi surveillance, Inpatient, Outpatient	Country	Bahrain	01/2007 to 12/2017	Ministry of Health (Bahrain). Bahrain Health Statistics 2017. Juffair, Bahrain: Ministry of Health (Bahrain).
Survey	NA	Country	Bahrain	01/1998 to 12/1999	Ministry of Health (Bahrain). Bahrain National Nutrition Survey 1998-1999.
Survey	Household	Country	Bahrain	01/2007 to 12/2007	Ministry of Health (Bahrain), World Health Organization (WHO). Bahrain STEPS Noncommunicable Disease Risk Factors Survey 2007.
Scientific literature	NA	NA	Bahrain	01/2005 to 12/2005	Al-Mahroos F,¬†Al-Roomi K. Diabetic neuropathy, foot ulceration, peripheral vascular disease and potential risk factors among patients with diabetes in Bahrain: a nationwide primary care diabetes clinic-based study. Ann Saudi Med. 2007; 27(1): 25-31.
Scientific literature	NA	NA	Bahrain	01/1996 to 12/1996	al-Mahroos F, McKeigue PM. High prevalence of diabetes in Bahrainis. Associations with ethnicity and raised plasma cholesterol. Diabetes Care. 1998; 21(6): 936-42.
Scientific literature	NA	Country	Algeria, Argentina, Bahrain, Bangladesh, China, Egypt, India, Indonesia, Iran (Islamic Republic of), Jordan, Kuwait, Libya, Malaysia, Mexico, Morocco, Pakistan, Philippines, Qatar, Russian Federation, Saudi Arabia, Singapore, Republic of Korea, Taiwan (Province of China), Tunisia, Turkiye, United Arab Emirates, Yemen	01/2009 to 12/2010	Litwak L, Goh SY, Hussein Z, Malek R, Prusty V, Khamseh ME. Prevalence of diabetes complications in people in type 2 diabetes mellitus and its association with baseline characteristics in the multinational A1chieve study . Diabetol Metab Syndr. 2013; 5(1): 57.
Scientific literature	NA	NA	Bahrain	01/2003 to 12/2009	Al Alawi E, Ahmed AA. Screening for diabetic retinopathy: the first telemedicine approach in a primary care setting in Bahrain. Middle East Afr J Ophthalmol. 2012; 19(3): 295-8.
Scientific literature	NA	NA	Canada, Egypt, Finland, Japan, Netherlands, Republic of Korea, Sweden, Taiwan (Province of China), United Kingdom, United States of America, Hawaii	01/1996 to 05/2016	Zhang J, Chen C, Hua S, Liao H, Wang M, Xiong Y, Cao F. An updated meta-analysis of cohort studies: diabetes and risk of Alzheimer's disease. Diabetes Res Clin Pract. 2017; Feb (124): 41-47.

Data type	Secondary data type	Coverage type	Geography	Time period covered	Suggested citation
Scientific literature	NA	NA	Egypt	01/1985 to 12/1985	Arab M. Diabetes mellitus in Egypt. World Health Stat Q. 1992; 45(4): 334-7.
Survey	Cross-sectional, GPS coordinates (GIS), Household, Individual, Interview, Nationally representative	Country	Egypt	03/2008 to 06/2008	El-Zanaty and Associates, Macro International, Inc, Ministry of Health and Population (Egypt). Egypt Demographic and Health Survey 2008. Fairfax, United States of America: ICF International.
Survey	Household, Individual, Longitudinal	Country	Egypt	10/1996 to 11/1997	Macro International, Inc, Population Council (Egypt). Egypt In Depth Demographic and Health Survey 1996-1997. Fairfax, United States of America: ICF International.
Survey	Cross-sectional, Household, Individual, Interview	Country	Egypt	02/2015 to 05/2015	El-Zanaty and Associates, ICF International, Ministry of Health and Population (Egypt), Population Council (Egypt). Egypt Special Demographic and Health Survey 2015. Fairfax, United States of America: ICF International.
Report	NA	Country	Egypt	01/1960 to 12/2005	Central Agency for Public Mobilization and Statistics (CAPMAS) (Egypt). Egypt Statistical Yearbook 2005. Cairo, Egypt: Central Agency for Public Mobilization and Statistics (CAPMAS) (Egypt), 2006.
Report	NA	Country	Egypt	01/1960 to 12/2008	Central Agency for Public Mobilization and Statistics (CAPMAS) (Egypt). Egypt Statistical Yearbook 2009. Cairo, Egypt: Central Agency for Public Mobilization and Statistics (CAPMAS) (Egypt), 2009.
Report	NA	Country	Egypt	01/1960 to 12/2009	Central Agency for Public Mobilization and Statistics (CAPMAS) (Egypt). Egypt Statistical Yearbook 2010. Cairo, Egypt: Central Agency for Public Mobilization and Statistics (CAPMAS) (Egypt), 2010.
Report	NA	Country	Egypt	01/1960 to 12/2010	Central Agency for Public Mobilization and Statistics (CAPMAS) (Egypt). Egypt Statistical Yearbook 2011. Cairo, Egypt: Central Agency for Public Mobilization and Statistics (CAPMAS) (Egypt), 2011.
Report	NA	Country	Egypt	01/1960 to 12/2011	Central Agency for Public Mobilization and Statistics (CAPMAS) (Egypt). Egypt Statistical Yearbook 2012. Cairo, Egypt: Central Agency for Public Mobilization and Statistics (CAPMAS) (Egypt), 2012.
Report	NA	Country	Egypt	01/1960 to 12/2012	Central Agency for Public Mobilization and Statistics (CAPMAS) (Egypt). Egypt Statistical Yearbook 2013. Cairo, Egypt: Central Agency for Public Mobilization and Statistics (CAPMAS) (Egypt), 2013.
Report	NA	Country	Egypt	01/1960 to 12/2016	Central Agency for Public Mobilization and Statistics (CAPMAS) (Egypt). Egypt Statistical Yearbook 2017. Cairo, Egypt: Central Agency for Public Mobilization and Statistics (CAPMAS) (Egypt), 2017.
Survey	Household	Country	Egypt	01/2005 to 12/2005	Ministry of Health and Population (Egypt), United States Agency for International Development (USAID), World Health Organization (WHO). Egypt STEPS Noncommunicable Disease Risk Factors Survey 2005.
Survey	Household, Interview	Country	Egypt	11/2011 to 01/2012	Ministry of Health and Population (Egypt), United States Agency for International Development (USAID), World Health Organization (WHO). Egypt STEPS Noncommunicable Disease Risk Factors Survey 2011-2012.
Survey	Cross-sectional, Household, Individual, Interview, Nationally representative	Country	Egypt	07/2017 to 10/2017	Central Agency for Public Mobilization and Statistics (CAPMAS) (Egypt), Ministry of Health and Population (Egypt), World Health Organization (WHO). Egypt STEPS Noncommunicable Disease Risk Factors Survey 2017.
Survey	Community, Household, Nationally representative	Country	Egypt	01/2009 to 12/2009	Information and Decision Support Center (IDSC) (Egypt), Population Council. Egypt Survey of Young People 2009. New York City , United States of America: Population Council.
Scientific literature	NA	NA	Egypt	01/1996 to 12/2011	El-Ziny MA, Salem NA, El-Hawary AK, Chalaby NM, Elsharkawy AA. Epidemiology of childhood type 1 diabetes mellitus in Nile Delta, northern Egypt - a retrospective study. J Clin Res Pediatr Endocrinol. 2014; 6(1): 9-15.
Scientific literature	NA	NA	Egypt	01/2007 to 12/2008	Macky TA, Khater N, Al-Zamil MA, El Fishawy H, Soliman MM. Epidemiology of diabetic retinopathy in Egypt: a hospital-based study. Ophthalmic Res. 2011; 45(2): 73-8.
Scientific literature	NA	NA	Egypt	01/2004 to 12/2004	Marzouk D, Sass J, Bakr I, El Hosseiny M, Abdel-Hamid M, Rekacewicz C, Chaturvedi N, Mohamed MK, Fontanet A. Metabolic and cardiovascular risk profiles and hepatitis C virus infection in rural Egypt. Gut. 2007; 56(8): 1105-10.
Scientific literature	NA	NA	Egypt	01/2013 to 12/2015	Khedr EM, Fawi G, Allah Abbas MA, El-Fetoh NA, Al Attar G, Zaki AF, Gamea A. Prevalence of Diabetes and Diabetic Neuropathy in Qena Governorate: Population-Based Survey. Neuroepidemiology. 2016; 46(3): 173,Äì81.
Scientific literature	NA	Country	Algeria, Argentina, Bahrain, Bangladesh, China, Egypt, India, Indonesia, Iran (Islamic Republic of), Jordan, Kuwait, Libya, Malaysia, Mexico, Morocco, Pakistan, Philippines, Qatar, Russian Federation, Saudi Arabia, Singapore, Republic of Korea, Taiwan (Province of China), Tunisia, Turkiye, United Arab Emirates, Yemen	01/2009 to 12/2010	Litwak L, Goh SY, Hussein Z, Malek R, Prusty V, Khamseh ME. Prevalence of diabetes complications in people in type 2 diabetes mellitus and its association with baseline characteristics in the multinational A1chieve study . Diabetol Metab Syndr. 2013; 5(1): 57.

Data type	Secondary data type	Coverage type	Geography	Time period covered	Suggested citation
Scientific literature	NA	NA	Egypt, Jordan, Kuwait, Lebanon	01/2009 to 12/2009	Jambart S, Ammache Z, Haddad F, Younes A, Hassoun A, Abdalla K, Selwan CA, Sunna N, Wajsbrot D, Youseif E. Prevalence of painful diabetic peripheral neuropathy among patients with diabetes mellitus in the Middle East region. J Int Med Res. 2011; 39(2): 366-77.
Scientific literature	NA	NA	Egypt	01/2006 to 12/2008	Al-Emam A, Elhaddad AA, Ramadan E. The risk of clinically diagnosed alzheimer disease in patients with non insulin dependent diabetes mellitus. Egypt J Neurol Psychiatr Neurosurg. 2010; 47: 419,Äì24.
Scientific literature	NA	NA	Iran (Islamic Republic of)	01/2006 to 12/2014	Bahadoran Z, Mirmiran P, Momenan AA, Azizi F. Allium vegetable intakes and the incidence of cardiovascular disease, hypertension, chronic kidney disease, and type 2 diabetes in adults: a longitudinal follow-up study. J Hypertens. 2017; 35(9): 1909-1916.
Scientific literature	NA	Subnational	Iran (Islamic Republic of), Qazvƒ´n	01/2010 to 12/2011	Ghorbani A, Ziaee A, Esmailzadehha N, Javadi H. Association between health-related quality of life and impaired glucose metabolism in Iran: the Qazvin Metabolic Diseases Study. Diabet Med. 2014; 31(6): 754,Äì8.
Scientific literature	NA	Subnational	Iran (Islamic Republic of), TehrƒÅn	01/2006 to 12/2007	Ghasemi A, Zahediasl S, Syedmoradi L, Azizi F. Association between serum nitric oxide metabolites and hypertension in a general population. Int Angiol. 2011; 30(4): 380,Äì7.
Scientific literature	NA	Subnational	Iran (Islamic Republic of), E≈üfahƒÅn	01/2007 to 12/2012	Najafian J, Mohamadifard N, Siadat ZD, Sadri G, Rahmati MR. Association between sleep duration and diabetes mellitus: Isfahan Healthy Heart Program. Niger J Clin Pract. 2013; 16(1): 59,Äì62.
Scientific literature	NA	Subnational	Iran (Islamic Republic of), E≈üfahƒÅn	01/2002 to 12/2010	Janghorbani M, Amini M. Associations of hip circumference and height with incidence of type 2 diabetes: the Isfahan diabetes prevention study. Acta Diabetol. 2012; 49 Suppl 1: S107,Äì114.
Scientific literature	NA	Subnational	Iran (Islamic Republic of), E≈üfahƒÅn	01/2000 to 12/2001	Shirani S, Kelishadi R, Sarrafzadegan N, Khosravi A, Sadri G, Amani A, Heidari S, Ramezani MA. Awareness, treatment and control of hypertension, dyslipidaemia and diabetes mellitus in an Iranian population: the IHHP study. East Mediterr Health J. 2009; 15(6): 1455,Äì63.
Scientific literature	NA	Subnational	Iran (Islamic Republic of), B≈´shehr	01/2013 to 12/2014	Ostovar A, Nabipour I, Larijani B, Heshmat R, Darabi H, Vahdat K, Ravanipour M, Mehrdad N, Raeisi A, Heidari G, Shafiee G, Haeri M, Pourbehi M, Sharifi F, Noroozi A, Tahmasebi R, Aghaei Meybodi H, Assadi M, Farrokhi S, Nemati R, Amini MR, Barekat M, Amini A, Salimipour H, Dobaradaran S, Moshtaghi D. Bushehr Elderly Health (BEH) Programme, phase I (cardiovascular system). BMJ Open. 2015; 5(12): e009597.
Scientific literature	NA	NA	Iran (Islamic Republic of)	01/2005 to 12/2005	Alavi A, Sanjari M, Haghdoost A, Sibbald RG. Common foot examination features of 247 Iranian patients with diabetes. Int Wound J. 2009; 6(2): 117-22.
Scientific literature	NA	Subnational	Iran (Islamic Republic of), E≈üfahƒÅn	01/2003 to 12/2005	Janghorbani M, Amini M. Comparison of fasting glucose with post-load glucose values and glycated hemoglobin for prediction of type 2 diabetes: the Isfahan diabetes prevention study. Rev Diabet Stud. 2009; 6(2): 117-23.
Scientific literature	NA	Subnational	Iran (Islamic Republic of), E≈üfahƒÅn	01/2001 to 12/2001	Sadeghi M, Roohafza H, Shirani S, Poormoghadas M, Kelishadi R, Baghaii A, Sarraf-Zadegan N. Diabetes and associated cardiovascular risk factors in Iran: the Isfahan Healthy Heart Programme. Ann Acad Med Singapore. 2007; 36(3): 175-80.
Scientific literature	NA	NA	Iran (Islamic Republic of)	01/2004 to 12/2004	Golozar A, Khademi H, Kamangar F, Poutschi H, Islami F, Abnet CC, Freedman ND, Taylor PR, Pharoah P, Boffetta P, Brennan PJ, Dawsey SM, Malekzadeh R, Etemadi A. Diabetes mellitus and its correlates in an Iranian adult population. PLoS One. 2011; 6(10): e26725.
Scientific literature	NA	Subnational	Iran (Islamic Republic of), TehrƒÅn	01/2006 to 12/2008	Mirmiran P, Hajifaraji M, Bahadoran Z, Sarvghadi F, Azizi F. Dietary protein intake is associated with favorable cardiometabolic risk factors in adults: Tehran Lipid and Glucose Study. Nutr Res. 2012; 32(3): 169,Äì76.
Scientific literature	NA	NA	Iran (Islamic Republic of)	01/2005 to 12/2005	Farzadfar F, Murray CJL, Gakidou E, Bossert T, Namdaritabar H, Alikhani S, Moradi G, Delavari A, Jamshidi H, Ezzati M. Effectiveness of diabetes and hypertension management by rural primary health-care workers (Behvarz workers) in Iran: a nationally representative observational study. Lancet. 2012; 379(9810): 47,Äì54.
Scientific literature	NA	NA	Iran (Islamic Republic of)	01/2004 to 12/2007	Manaviat MR, Rashidi M, Afkhami-Ardekani M. Four years incidence of diabetic retinopathy and effective factors on its progression in type II diabetes. Eur J Ophthalmol. 2008; 18(4): 572-7.
Scientific literature	NA	NA	Iran (Islamic Republic of)	01/2007 to 12/2007	Haghighatdoost F, Sarrafzadegan N, Mohammadifard N, Sajjadi F, Maghroon M, Boshtam M, Alikhasi H, Azadbakht L. Healthy eating index and cardiovascular risk factors among Iranians. J Am Coll Nutr. 2013; 32(2): 111,Äì21.
Scientific literature	NA	NA	Iran (Islamic Republic of)	01/2009 to 12/2014	Ebrahimi H, Emamian MH, Hashemi H, Fotouhi A. High Incidence of Diabetes Mellitus Among a Middle-Aged Population in Iran: A Longitudinal Study. Can J Diabetes. 2016; 40(6): 570,Äì5.
Scientific literature	NA	NA	France, Iran (Islamic Republic of)	01/1999 to 12/2003	Azimi-Nezhad M, Herbeth B, Siest G, Dad√© S, Ndiaye NC, Esmaily H, Hosseini SJ, Ghayour-Mobarhan M, Visvikis-Siest S. High prevalence of metabolic syndrome in Iran in comparison with France: what are the components that explain this?. Metab Syndr Relat Disord. 2012; 10(3): 181,Äì8.
Scientific literature	NA	Subnational	Iran (Islamic Republic of), TehrƒÅn	01/1999 to 12/2001	Hadaegh F, Shafiee G, Ghasemi A, Sarbakhsh P, Azizi F. Impact of metabolic syndrome, diabetes and prediabetes on cardiovascular events: Tehran lipid and glucose study. Diabetes Res Clin Pract. 2010; 87(3): 342,Äì7.
Scientific literature	NA	Subnational	Iran (Islamic Republic of), E≈üfahƒÅn	01/1992 to 12/2001	Janghorbani M, Amini M, Ghanbari H, Safaiee H. Incidence of and risk factors for diabetic retinopathy in Isfahan, Iran. Ophthalmic Epidemiol. 2003; 10(2): 81-95.
Survey	Household, Individual, Interview, Nationally representative	Subnational	Iran (Islamic Republic of), GolestƒÅn	01/2004 to 12/2008	International Agency for Research on Cancer (IARC), National Cancer Institute (NCI) (United States), Tehran University of Medical Sciences. Iran - Golestan Cohort Study 2004-2008 - Teheran University of Medical Sciences.
Survey	Cross-sectional, Individual, Interview, Subnationally representative	Subnational	Iran (Islamic Republic of), FƒÅrs	Jan-13	Digestive Diseases Research Institute (DDRI) (Iran), Shiraz University of Medical Sciences. Iran - Pars Cohort Study.

Data type	Secondary data type	Coverage type	Geography	Time period covered	Suggested citation
Survey	Household	Country	Iran (Islamic Republic of)	01/2010 to 12/2010	Ministry of Health and Medical Education (Iran), Statistical Centre of Iran. Iran Multiple Indicator Demographic and Health Survey 2010.
Survey	Longitudinal	Country	Iran (Islamic Republic of)	Jan-02	Hamilton Health Sciences, McMaster University (Canada), Population Health Research Institute (PHRI). Iran Prospective Urban and Rural Epidemiological Study.
Survey	Cross-sectional, Household, Individual, Interview, Nationally representative	Country	Iran (Islamic Republic of)	01/2005 to 02/2005	World Health Organization (WHO), Ministry of Health and Medical Education (Iran), Center for Non-Communicable Diseases Control (Iran). Iran STEPS Noncommunicable Disease Risk Factors Survey 2005.
Survey	Cross-sectional	Country	Iran (Islamic Republic of)	05/2006 to 06/2006	Ministry of Health and Medical Education (Iran), World Health Organization (WHO). Iran STEPS Noncommunicable Disease Risk Factors Survey 2006.
Survey	Household	Country	Iran (Islamic Republic of)	05/2007 to 06/2007	World Health Organization (WHO), Ministry of Health and Medical Education (Iran), Center for Non-Communicable Diseases Control (Iran). Iran STEPS Noncommunicable Disease Risk Factors Survey 2007.
Survey	Cross-sectional, Household	Country	Iran (Islamic Republic of)	May-08	Ministry of Health and Medical Education (Iran), World Health Organization (WHO). Iran STEPS Noncommunicable Disease Risk Factors Survey 2008.
Survey	Household, Interview	Country	Iran (Islamic Republic of)	01/2009 to 12/2009	Ministry of Health and Medical Education (Iran), World Health Organization (WHO). Iran STEPS Noncommunicable Disease Risk Factors Survey 2009.
Survey	Individual, Nationally representative	Country	Iran (Islamic Republic of)	04/2016 to 11/2016	Ministry of Health and Medical Education (Iran), National Institute of Health Research, Ministry of Health and Medical Education (Iran), Tehran University of Medical Sciences. Iran STEPS Noncommunicable Disease Risk Factors Survey 2016.
Scientific literature	NA	NA	Iran (Islamic Republic of)	01/1991 to 12/1996	Pishdad GR. Low incidence of type 1 diabetes in Iran. Diabetes Care. 2005; 28(4): 927-8.
Scientific literature	NA	Country	Colombia, Iran (Islamic Republic of), Mexico, Thailand, United Kingdom, England, Scotland, United States of America	01/2003 to 12/2007	Gakidou E,¬†Mallinger L,¬†Abbott-Klafter J,¬†Guerrero R,¬†Villalpando S,¬†Ridaura RL,¬†Aekplakorn W,¬†Naghavi M,¬†Lim S,¬†Lozano R,¬†Murray CJ. Management of diabetes and associated cardiovascular risk factors in seven countries: a comparison of data from national health examination surveys. Bull World Health Organ. 2011; 89(3): 172-83.
Scientific literature	NA	Subnational	Iran (Islamic Republic of), TehrƒÅn	01/2006 to 12/2008	Shab-Bidar S, Hosseini-Esfahani F, Mirmiran P, Hosseinpour-Niazi S, Azizi F. Metabolic syndrome profiles, obesity measures and intake of dietary fatty acids in adults: Tehran Lipid and Glucose Study. J Hum Nutr Diet. 2014; 27 Suppl 2: 98,Äì108.
Scientific literature	NA	Subnational	Iran (Islamic Republic of), E≈üfahƒÅn	01/2003 to 12/2005	Janghorbani M, Amini M. Normal fasting plasma glucose and risk of prediabetes and type 2 diabetes: the Isfahan Diabetes Prevention Study. Rev Diabet Stud. 2011; 8(4): 490-8.
Scientific literature	NA	Subnational	Iran (Islamic Republic of), E≈üfahƒÅn	01/2000 to 12/2003	Janghorbani M, Rezvanian H, Kachooei A, Ghorbani A, Chitsaz A, Izadi F, Amini M. Peripheral neuropathy in type 2 diabetes mellitus in Isfahan, Iran: prevalence and risk factors. Acta Neurol Scand. 2006; 114(6): 384,Äì91.
Scientific literature	NA	NA	Iran (Islamic Republic of)	01/1997 to 12/2004	Hosseinpanah F, Rambod M, Azizi F. Population attributable risk for diabetes associated with excess weight in Tehranian adults: a population-based cohort study. BMC Public Health. 2007; 7: 328.
Scientific literature	NA	Subnational	Iran (Islamic Republic of), TehrƒÅn	01/1999 to 12/2001	Harati H, Hadaegh F, Saadat N, Azizi F. Population-based incidence of Type 2 diabetes and its associated risk factors: results from a six-year cohort study in Iran. BMC Public Health. 2009; 186.
Scientific literature	NA	NA	Iran (Islamic Republic of)	01/2008 to 12/2009	Hasani N, Khosrawi S, Hashemipour M, Haghighatiyan M, Javdan Z, Taheri MH, Kelishadi R, Amini M, Barekatein R. Prevalence and related risk-factors of peripheral neuropathy in children with insulin-dependent diabetes mellitus. J Res Med Sci. 2013; 18(2): 132-6.
Scientific literature	NA	Subnational	Iran (Islamic Republic of), Yazd	01/2010 to 12/2011	Dehghan MH, Katibeh M, Ahmadieh H, Nourinia R, Yaseri M. Prevalence and risk factors for diabetic retinopathy in the 40 to 80 year-old population in Yazd, Iran: the Yazd Eye Study. J Diabetes. 2015; 7(1): 139-41.
Scientific literature	NA	Subnational	Iran (Islamic Republic of), E≈üfahƒÅn	01/1993 to 12/1993	Amini M, Afshin-Nia F, Bashardoost N, Aminorroaya A, Shahparian M, Kazemi M. Prevalence and risk factors of diabetes mellitus in the Isfahan city population (aged 40 or over) in 1993. Diabetes Res Clin Pract. 1997; 38(3): 185-90.
Scientific literature	NA	Country	Algeria, Argentina, Bahrain, Bangladesh, China, Egypt, India, Indonesia, Iran (Islamic Republic of), Jordan, Kuwait, Libya, Malaysia, Mexico, Morocco, Pakistan, Philippines, Qatar, Russian Federation, Saudi Arabia, Singapore, Republic of Korea, Taiwan (Province of China), Tunisia, Turkiye, United Arab Emirates, Yemen	01/2009 to 12/2010	Litwak L, Goh SY, Hussein Z, Malek R, Prusty V, Khamseh ME. Prevalence of diabetes complications in people in type 2 diabetes mellitus and its association with baseline characteristics in the multinational A1chieve study . Diabetol Metab Syndr. 2013; 5(1): 57.
Scientific literature	NA	NA	Iran (Islamic Republic of)	01/2005 to 12/2005	Abdollahi A, Malekmadani MH, Mansoori MR, Bostak A, Abbaszadeh MR, Mirshahi A. Prevalence of diabetic retinopathy in patients with newly diagnosed type II diabetes mellitus. Acta Med Iran. 2006; 44(6): 415-19.

Data type	Secondary data type	Coverage type	Geography	Time period covered	Suggested citation
Scientific literature	NA	Subnational	Iran (Islamic Republic of), TehrƒÅn	01/2007 to 12/2007	Javadi MA,¬†Katibeh M,¬†Rafati N,¬†Dehghan MH,¬†Zayeri F,¬†Yaseri M,¬†Sehat M,¬†Ahmadieh H. Prevalence of diabetic retinopathy in Tehran province: a population-based study. BMC Ophthalmol. 2009; 9(1): 12.
Scientific literature	NA	Subnational	Iran (Islamic Republic of), MƒÅzandarƒÅn, Sƒ´stƒÅn va Bal≈´chestƒÅn	01/2012 to 12/2013	Ostovaneh MR, Zamani F, Sharafkhah M, Ansari-Moghaddam A, Akhavan Khaleghi N, Saeedian FS, Rohani Z, Motamed N, Maadi M, Malekzadeh R, Poustchi H. Prevalence of metabolic syndrome in Amol and Zahedan, Iran: a population based study. Arch Iran Med. 2014; 17(7): 477‚Äì82.
Scientific literature	NA	NA	Iran (Islamic Republic of)	01/2011 to 12/2011	Noshad S, Abbasi M, Etemad K, Meysamie A, Afarideh M, Khajeh E, Asgari F, Mousavizadeh M, Rafei A, Neishaboury M, Ghajar A, Nakhjavani M, Koohpayehzadeh J, Esteghamati A. Prevalence of metabolic syndrome in Iran: A 2011 update. J Diabetes. 2017; 9(5): 518-525.
Scientific literature	NA	NA	Iran (Islamic Republic of)	01/2005 to 12/2007	Azimi-Nezhad M, Ghayour-Mobarhan M, Parizadeh MR, Safarian M, Esmaeili H, Parizadeh SM, Khodaee G, Hosseini J, Abasalti Z, Hassankhani B, Ferns G. Prevalence of type 2 diabetes mellitus in Iran and its relationship with gender, urbanisation, education, marital status and occupation. Singapore Med J. 2008; 49(7): 571-6.
Scientific literature	NA	NA	Iran (Islamic Republic of)	01/2009 to 12/2009	Rajavi Z, Katibeh M, Ziaei H, Fardesmaeilpour N, Sehat M, Ahmadieh H, Javadi MA. Rapid assessment of avoidable blindness in Iran. Ophthalmology. 2011; 118(9): 1812-8.
Scientific literature	NA	NA	Iran (Islamic Republic of)	01/1999 to 12/2009	Fahimfar N, Khalili D, Mohebi R, Azizi F, Hadaegh F. Risk factors for ischemic stroke; results from 9 years of follow-up in a population based cohort of Iran. BMC Neurol. 2012; 12: 117.
Scientific literature	NA	NA	Iran (Islamic Republic of)	01/2010 to 12/2010	Vatankhah N, Noudeh YJ, Khamseh ME, Baradaran HR. Screening people with type 2 diabetes at risk for foot ulceration in Iran. Diabetes Technol Ther. 2010; 12(9): 731-6.
Scientific literature	NA	Subnational	Iran (Islamic Republic of), TehrƒÅn	01/2002 to 12/2011	Kheirandish M, Asgari S, Lotfaliany M, Bozorgmanesh M, Saadat N, Tohidi M, Azizi F, Hadaegh F. Secular trends in serum lipid levels of a Middle Eastern adult population; 10 years follow up in Tehran lipid and glucose study. Lipids Health Dis. 2014; 13: 20.
Scientific literature	NA	Subnational	Iran (Islamic Republic of), TehrƒÅn	01/1999 to 12/2011	Derakhshan A, Sardarinia M, Khalili D, Momenan AA, Azizi F, Hadaegh F. Sex specific incidence rates of type 2 diabetes and its risk factors over 9 years of follow-up: Tehran Lipid and Glucose Study. PLoS One. 2014; 9(7): e102563.
Scientific literature	NA	Subnational	Iran (Islamic Republic of), TehrƒÅn	01/1998 to 12/2008	Hadaegh F, Shafiee G, Hatami M, Azizi F. Systolic and diastolic blood pressure, mean arterial pressure and pulse pressure for prediction of cardiovascular events and mortality in a Middle Eastern population. Blood Press. 2012; 21(1): 12‚Äì8.
Scientific literature	NA	NA	Iran (Islamic Republic of)	01/2007 to 12/2007	Shirani S, Heidari K, Sabzghabaee AM, Mirmoghtadaee P, Hoseini L, Aalifar H, Fadaei H, Esnaashari H, Soltani R. The modifiable noncommunicable risk factors among an Iranian population. Southeast Asian J Trop Med Public Health. 2012; 43(5): 1227-32.
Scientific literature	NA	NA	Iran (Islamic Republic of)	01/2014 to 12/2017	Mansour-Ghanaei F, Joukar F, Naghipour MR, Sepanlou SG, Poustchi H, Mojtahedi K, Balou HA, Heidarzadeh A, Malekzadeh R. The PERSIAN Guilan Cohort Study (PGCS). Arch Iran Med. 2019; 22(1): 39-45.
Scientific literature	NA	Subnational	Iran (Islamic Republic of), HamadƒÅn	01/2011 to 12/2011	Kiani J, Moghimbeigi A, Azizkhani H, Kosarifard S. The prevalence and associated risk factors of peripheral diabetic neuropathy in Hamedan, Iran. Arch Iran Med. 2013; 16(1): 17‚Äì9.
Scientific literature	NA	Subnational	Iran (Islamic Republic of), E≈üfahƒÅn	01/1980 to 12/1995	Sarraf-Zadegan N, Sayed-Tabatabaei FA, Bashardoost N, Maleki A, Totonchi M, Habibi HR, Sotodehmaram E, Tafazoli F, Karimi A. The prevalence of coronary artery disease in an urban population in Isfahan, Iran. Acta Cardiol. 1999; 54(5): 257-63.
Scientific literature	NA	Subnational	Iran (Islamic Republic of), KhorƒÅsƒÅn-e Jon≈´bƒ´	01/2012 to 12/2012	Chahkandi T, Taheri F, Kazemi T, Bijari B. The Prevalence of Diabetes and Prediabetes Among Elementary School Children in Birjand. Iran J Pediatr. 2015; 25(1): e183.
Scientific literature	NA	NA	Iran (Islamic Republic of)	01/2004 to 12/2004	Tabatabaei-Malazy O, Mohajeri-Tehrani M, Madani S, Heshmat R, Larijani B. The prevalence of diabetic peripheral neuropathy and related factors. Iran J Public Health. 2011; 40(3): 55‚Äì62.
Scientific literature	NA	NA	Iran (Islamic Republic of)	01/2004 to 12/2013	Golozar A, Khalili D, Etemadi A, Poustchi H, Fazeltabar A, Hosseini F, Kamangar F, Khoshnia M, Islami F, Hadaegh F, Brennan P, Boffetta P, Abnet CC, Dawsey SM, Azizi F, Malekzadeh R, Danaei G. White rice intake and incidence of type-2 diabetes: analysis of two prospective cohort studies from Iran. BMC Public Health. 2017; 17(1): 133.
Scientific literature	NA	NA	Iraq	01/2012 to 12/2016	Almahfoodh D, Alabbood M, Alali A, Mansour A. Epidemiology of type 1 diabetes mellitus in Basrah, Southern Iraq: A retrospective study. Diabetes Res Clin Pract. 2017; 133: 104-108.
Survey	Household	Subnational	Iraq, Dah≈´k	12/2003 to 01/2004	Directorate General of Health-Duhok (Iraq), Kurdistan Regional Government (Iraq), Ministry of Health (Iraq), World Health Organization (WHO). Iraq - Dah≈´k STEPS Noncommunicable Disease Risk Factors Survey 2003-2004.
Survey	Household, Nationally representative, Urban-rural representative	Country	Iraq	08/2006 to 03/2007	Ministry of Health (Iraq), Central Organization for Statistics and Information Technology (Iraq), Kurdistan Regional Statistics Office, World Health Organization (WHO), Ministry of Health (Kurdistan). Iraq Family Health Survey 2006-2007.
Survey	Household, Individual, Interview, Nationally representative, Subnationally representative, Urban-rural representative	Country	Iraq	11/2006 to 11/2007	Central Organization for Statistics and Information Technology (Iraq), Kurdistan Regional Statistics Office, World Bank. Iraq Household Socioeconomic Survey 2006-2007. Washington DC, United States of America: World Bank.
Survey	Household, Individual, Interview, Nationally representative, Subnationally representative, Urban-rural representative	Country	Iraq	02/2012 to 01/2013	OAMDI, 2016. Harmonized Household Income and Expenditure Surveys (HHIES), http://erf.org.eg/data-portal/. Version 2.0 of Licensed Data Files; IHSES 2012- Central Organization for Statistics (COS). Egypt: Economic Research Forum (ERF).¬†

Data type	Secondary data type	Coverage type	Geography	Time period covered	Suggested citation
Survey	Household	Country	Iraq	01/2006 to 02/2006	Central Organization for Statistics and Information Technology (Iraq), Ministry of Health (Iraq), World Health Organization (WHO). Iraq STEPS Noncommunicable Disease Risk Factors Survey 2006.
Survey	Cross-sectional, Household, Individual, Interview, Nationally representative	Country	Iraq	08/2015 to 12/2015	Ministry of Health (Iraq), Ministry of Planning (Iraq), World Health Organization (WHO). Iraq STEPS Noncommunicable Disease Risk Factors Survey 2015.
Scientific literature	NA	NA	Iraq	01/2011 to 12/2012	Mansour AA, Al-Maliky AA, Kasem B, Jabar A, Mosbeh KA. Prevalence of diagnosed and undiagnosed diabetes mellitus in adults aged 19 years and older in Basrah, Iraq. Diabetes Metab Syndr. 2014; 139,Äì44.
Scientific literature	NA	NA	Jordan	01/1997 to 12/1997	Ajlouni K, Jaddou H, Batieha A. Diabetes and impaired glucose tolerance in Jordan: prevalence and associated risk factors. J Intern Med. 1998; 244(4): 317-23.
Scientific literature	NA	NA	Jordan	01/2009 to 12/2011	Jammal H, Khader Y, Alkhatib S, Abujbara M, Alomari M, Ajlouni K. Diabetic retinopathy in patients with newly diagnosed type 2 diabetes mellitus in Jordan: prevalence and associated factors. J Diabetes. 2013; 5(2): 172-9.
Scientific literature	NA	NA	Jordan	01/1992 to 12/1996	Ajlouni K, Qusous Y, Khawaldeh AK, Jaddou H, Batiehah A, Ammari F, Zaheri M, Mashal A. Incidence of insulin-dependent diabetes mellitus in Jordanian children aged 0-14 y during 1992-1996. Acta Paediatr Suppl. 1999; 88(427): 11-3.
Survey	Cross-sectional, GPS coordinates (GIS), Household, Individual, Interview, Nationally representative, Subnationally representative, Urban-rural representative	Country	Jordan	10/2017 to 01/2018	Department of Statistics (Jordan), ICF International. Jordan Demographic and Health Survey 2017-2018. Fairfax, United States of America: ICF International, 2019.
Survey	Household	Country	Jordan	01/2004 to 12/2004	Ministry of Health (Jordan), World Health Organization (WHO). Jordan STEPS Noncommunicable Disease Risk Factors Survey 2004.
Survey	Household, Individual, Interview, Nationally representative	Country	Jordan	01/2007 to 12/2007	Centers for Disease Control and Prevention (CDC), Ministry of Health (Jordan), World Health Organization (WHO). Jordan STEPS Noncommunicable Disease Risk Factors Survey 2007.
Scientific literature	NA	NA	Jordan	01/1996 to 12/1996	Ajlouni K, Jaddou H, Batieha A. Obesity in Jordan. Int J Obes Relat Metab Disord. 1998; 22(7): 624-8.
Scientific literature	NA	NA	Jordan	01/1994 to 12/2004	Khader Y, Batieha A, Ajlouni H, El-Khateeb M, Ajlouni K. Obesity in Jordan: prevalence, associated factors, comorbidities, and change in prevalence over ten years. Metab Syndr Relat Disord. 2008; 6(2): 113-20.
Scientific literature	NA	NA	Jordan	01/2001 to 12/2001	Jbour AS, Jarrah NS, Radaideh AM, Shegem NS, Bader IM, Batieha AM, Ajlouni KM. Prevalence and predictors of diabetic foot syndrome in type 2 diabetes mellitus in Jordan. Saudi Med J. 2003; 24(7): 761-4.
Scientific literature	NA	NA	Jordan	01/2012 to 12/2012	Rabiu MM, Al Bdour MD, Abu Ameerh MA, Jadoon MZ. Prevalence of blindness and diabetic retinopathy in northern Jordan. Eur J Ophthalmol. 2015; 25(4): 320-7.
Scientific literature	NA	Country	Algeria, Argentina, Bahrain, Bangladesh, China, Egypt, India, Indonesia, Iran (Islamic Republic of), Jordan, Kuwait, Libya, Malaysia, Mexico, Morocco, Pakistan, Philippines, Qatar, Russian Federation, Saudi Arabia, Singapore, Republic of Korea, Taiwan (Province of China), Tunisia, Turkiye, United Arab Emirates, Yemen	01/2009 to 12/2010	Litwak L, Goh SY, Hussein Z, Malek R, Prusty V, Khamseh ME. Prevalence of diabetes complications in people in type 2 diabetes mellitus and its association with baseline characteristics in the multinational A1chieve study . Diabetol Metab Syndr. 2013; 5(1): 57.
Scientific literature	NA	NA	Egypt, Jordan, Kuwait, Lebanon	01/2009 to 12/2009	Jambart S, Ammache Z, Haddad F, Younes A, Hassoun A, Abdalla K, Selwan CA, Sunna N, Wajsbrot D, Youseif E. Prevalence of painful diabetic peripheral neuropathy among patients with diabetes mellitus in the Middle East region. J Int Med Res. 2011; 39(2): 366-77.
Scientific literature	NA	NA	Jordan	01/2009 to 12/2009	Khader YS, Batieha A, Jaddou H, Batieha Z, El-Khateeb M, Ajlouni K. Relationship between 25-hydroxyvitamin D and metabolic syndrome among Jordanian adults. Nutr Res Pract. 2011; 5(2): 132-9.
Scientific literature	NA	NA	Jordan	01/2015 to 12/2017	Abujbara M, Batieha A, Khader Y, Jaddou H, El-Khateeb M, Ajlouni K. The Prevalence of Dyslipidemia among Jordanians. J Lipids. 2018; 2018.
Scientific literature	NA	NA	Jordan	01/1994 to 12/2017	Ajlouni K, Batieha A, Jaddou H, Khader Y, Abdo N, El-Khateeb M, Hyassat D, Al-Louzi D. Time trends in diabetes mellitus in Jordan between 1994 and 2017. Diabet Med. 2019; 36(9): 1176-82.
Scientific literature	NA	NA	Kuwait	01/2012 to 12/2013	Awad AI, Alsaleh FM. 10-year risk estimation for type 2 diabetes mellitus and coronary heart disease in Kuwait: a cross-sectional population-based study. PLoS One. 2015; 10(1): e0116742.
Scientific literature	NA	NA	Kuwait	01/2010 to 12/2012	Alarouj M, Bennakhi A, Alnesef Y, Sharifi M, Elkum N. Diabetes and associated cardiovascular risk factors in the State of Kuwait: the first national survey. Int J Clin Pract. 2013; 67(1): 89-96.

Data type	Secondary data type	Coverage type	Geography	Time period covered	Suggested citation
Scientific literature	NA	NA	Kuwait	01/1980 to 12/1981	Taha TH, Moussa MA, Rashid AR, Fenech FF. Diabetes mellitus in Kuwait. Incidence in the first 29 years of life. Diabetologia. 1983; 25(4): 306-8.
Scientific literature	NA	NA	Kuwait	01/1992 to 12/1997	Shaltout AA, Moussa MAA, Qabazard M, Abdella N, Karvonen M, Al-Khawari M, Al-Arouj M, Al-Nakhi A, Tuomilehto J, El-Gammal A, Kuwait Diabetes Study Group. Further evidence for the rising incidence of childhood Type 1 diabetes in Kuwait. Diabet Med. 2002; 19(6): 522-5.
Scientific literature	NA	NA	Kuwait	01/1992 to 12/1993	Shaltout AA, Qabazard MA, Abdella NA, LaPorte RE, al Arouj M, Ben Nekhi A, Moussa MA, al Khawari MA. High incidence of childhood-onset IDDM in Kuwait. Kuwait Study Group of Diabetes in Childhood. Diabetes Care. 1995; 18(7): 923-7.
Scientific literature	NA	NA	Kuwait	01/2008 to 12/2008	Al Zenki S, Al Omirah H, Al Hooti S, Al Hamad N, Jackson RT, Rao A, Al Jahmah N, Al Obaid I, Al Ghanim J, Al Somaie M, Zaghloul S, Al Othman A. High prevalence of metabolic syndrome among Kuwaiti adults--a wake-up call for public health intervention. Int J Environ Res Public Health. 2012; 9(5): 1984-96.
Scientific literature	NA	NA	Kuwait	01/1995 to 12/1999	Abdul-Rasoul M, Al-Qattan H, Al-Haj A, Habib H, Ismael A. Incidence and seasonal variation of Type 1 diabetes in children in Farwania area, Kuwait (1995-1999). Diabetes Res Clin Pract. 2002; 56(2): 153-7.
Scientific literature	NA	NA	Algeria, Argentina, Australia, Austria, Barbados, Belgium, Brazil, Bulgaria, Canada, Chile, China, Colombia, Cuba, Denmark, Dominica, Estonia, Finland, France, Germany, Greece, Hungary, Israel, Italy, Japan, Kuwait, Latvia, Lithuania, Luxembourg, Mauritius, Mexico, Netherlands, New Zealand, Norway, Pakistan, Paraguay, Peru, Poland, Portugal, Romania, Russian Federation, Slovakia, Slovenia, Spain, Sudan, Tunisia, United Kingdom, United States of America, Puerto Rico, United States Virgin Islands, Uruguay, Venezuela (Bolivarian Republic of)	01/2000 to 12/2000	Karvonen M, Viik-Kajander M, Moltchanova E, Libman I, LaPorte R, Tuomilehto J. Incidence of childhood type 1 diabetes worldwide. Diabetes Mondiale (DiaMond) Project Group. Diabetes Care. 2000; 23(10): 1516,Äì26.
Scientific literature	NA	NA	Kuwait	01/1992 to 12/2013	Shaltout AA, Wake D, Thanaraj TA, Omar DM, Al-AbdulRazzaq D, Channanath A, AlKandari H, Abdulrasoul M, Miller S, Conway N, Tuomilehto J, Davidsson L, on behalf of the Steering Group for the Study of Childhood Diabetes in Kuwait. Incidence of type 1 diabetes has doubled in Kuwaiti children 0-14 years over the last 20 years. Pediatr Diabetes. 2017; 18(8): 761,Äì6.
Report	Discharge, Financial, Inpatient, National vital registration, Outpatient	Country	Kuwait	01/1957 to 12/2012	Central Statistical Bureau (Kuwait). Kuwait Annual Statistical Abstract 2012. Kuwait City, Kuwait: Central Statistical Bureau (Kuwait), 2013.
Report	Discharge, Epi surveillance, Financial, Inpatient, National vital registration, Outpatient	Country	Kuwait	01/1957 to 12/2013	Central Statistical Bureau (Kuwait). Kuwait Annual Statistical Abstract 2013. Kuwait City, Kuwait: Central Statistical Bureau (Kuwait), 2014.
Survey	Cross-sectional, Household, Individual, Interview, Nationally representative	Country	Kuwait	11/1996 to 12/1996	Health Ministers,Äô Council for GCC States, Ministry of Health (Kuwait), United Nations Statistics Division (UNSD). Kuwait Family Health Survey 1996.
Report	NA	Country	Kuwait	01/2003 to 12/2012	Ministry of Health (Kuwait). Kuwait Health Indicators 2003-2012. Kuwait City, Kuwait: Ministry of Health (Kuwait), 2014.
Survey	Household	Country	Kuwait	01/2006 to 12/2006	Ministry of Health (Kuwait), World Health Organization (WHO). Kuwait STEPS Noncommunicable Disease Risk Factors Survey 2006.
Survey	Cross-sectional, Individual, Interview, Nationally representative	Country	Kuwait	03/2014 to 09/2014	Ministry of Health (Kuwait), World Health Organization (WHO). Kuwait STEPS Noncommunicable Disease Risk Factors Survey 2014.
Survey	Cross-sectional, Household, Individual, Interview, Nationally representative, Subnationally representative	Country	Kuwait	01/2008 to 12/2010	Health Ministers,Äô Council for GCC States, Ministry of Health (Kuwait), World Health Organization (WHO). Kuwait World Health Survey 2008-2010.
Scientific literature	NA	NA	Kuwait	01/1996 to 12/1996	Abdella N, Al Arouj M, Al Nakhi A, Al Assoussi A, Moussa M. Non-insulin-dependent diabetes in Kuwait: prevalence rates and associated risk factors. Diabetes Res Clin Pract. 1998; 42(3): 187-96.

Data type	Secondary data type	Coverage type	Geography	Time period covered	Suggested citation
Scientific literature	NA	Country	Algeria, Argentina, Bahrain, Bangladesh, China, Egypt, India, Indonesia, Iran (Islamic Republic of), Jordan, Kuwait, Libya, Malaysia, Mexico, Morocco, Pakistan, Philippines, Qatar, Russian Federation, Saudi Arabia, Singapore, Republic of Korea, Taiwan (Province of China), Tunisia, Turkiye, United Arab Emirates, Yemen	01/2009 to 12/2010	Litwak L, Goh SY, Hussein Z, Malek R, Prusty V, Khamseh ME. Prevalence of diabetes complications in people in type 2 diabetes mellitus and its association with baseline characteristics in the multinational A1chieve study . Diabetol Metab Syndr. 2013; 5(1): 57.
Scientific literature	NA	NA	Egypt, Jordan, Kuwait, Lebanon	01/2009 to 12/2009	Jambart S, Ammache Z, Haddad F, Younes A, Hassoun A, Abdalla K, Selwan CA, Sunna N, Wajsbrot D, Youseif E. Prevalence of painful diabetic peripheral neuropathy among patients with diabetes mellitus in the Middle East region. J Int Med Res. 2011; 39(2): 366-77.
Scientific literature	NA	NA	Kuwait	01/2002 to 12/2002	Moussa MA, Alsaeid M, Abdella N, Refai TM, Al-Sheikh N, Gomez JE. Prevalence of type 1 diabetes among 6- to 18-year-old Kuwaiti children. Med Princ Pract. 2005; 14(2): 87-91.
Scientific literature	NA	NA	Kuwait	01/2002 to 12/2004	Al-Zuabi H, Al-Tammar Y, Al-Moataz R, Al-Sabti K, Wani VB, Hamama F, Mohammad H, Al-Suwayan MH. Retinopathy in newly diagnosed type 2 diabetes mellitus. Med Princ Pract. 2005; 14(5): 293-6.
Scientific literature	NA	NA	Kuwait	01/2000 to 12/2005	Al-Adsani AM. Risk factors for diabetic retinopathy in Kuwaiti type 2 diabetic patients. Saudi Med J. 2007; 28(4): 579-83.
Scientific literature	NA	NA	Kuwait	01/2002 to 12/2009	Ahmed F, Waslien C, Al-Sumaie MA, Prakash P, Allafi A. Trends and risk factors of hyperglycemia and diabetes among Kuwaiti adults: National Nutrition Surveillance Data from 2002 to 2009. BMC Public Health. 2013; 103.
Scientific literature	NA	NA	Libya	01/2008 to 12/2008	Elhwuegi AS, Darez AA, Langa AM, Bashaga NA. Cross-sectional pilot study about the health status of diabetic patients in city of Misurata, Libya. Afr Health Sci. 2012; 12(1): 81-6.
Scientific literature	NA	NA	Libya	01/1981 to 12/1990	Kadiki OA, Roaed RB. Epidemiological and clinical patterns of diabetes mellitus in Benghazi, Libyan Arab Jamahiriya. East Mediterr Health J. 1999; 5(1): 6-13.
Scientific literature	NA	NA	Libya	01/1981 to 12/1990	Kadiki OA, Moawad SE. Incidence and prevalence of type 1 diabetes in children and adolescents in Benghazi, Libya. Diabet Med. 1993; 10(9): 866-9.
Scientific literature	NA	NA	Libya	01/1981 to 12/1990	Kadiki OA, Reddy MR, Marzouk AA. Incidence of insulin-dependent diabetes (IDDM) and non-insulin-dependent diabetes (NIDDM) (0-34 years at onset) in Benghazi, Libya. Diabetes Res Clin Pract. 1996; 32(3): 165-73.
Scientific literature	NA	NA	Libya	01/1991 to 12/1995	Kadiki OA, Roaeid RB, Bhairi AM, Elamari IM. Incidence of insulin-dependent diabetes mellitus in Benghazi, Libya (1991-1995). Diabetes Metab. 1998; 24(5): 424-7.
Scientific literature	NA	NA	Libya	01/1991 to 12/2000	Kadiki OA, Roaeid RBM. Incidence of type 1 diabetes in children (0-14 years) in Benghazi Libya (1991-2000). Diabetes Metab. 2002; 28(6 Pt 1): 463-7.
Survey	Health facility, Household, Individual, Interview	Country	Libya	05/2007 to 10/2007	League of Arab States, National Center for Disease Control (Libya), Pan Arab Project for Family Health (PAPFAM). Libya Family Health Survey 2007.
Survey	Household, Interview	Country	Libya	02/2009 to 11/2009	Secretariat of Health and Environment (Libya), World Health Organization (WHO). Libya STEPS Noncommunicable Disease Risk Factors Survey 2009.
Scientific literature	NA	NA	Libya	01/2010 to 12/2010	Rabiu MM, Jenf M, Fituri S, Choudhury A, Agbabiaka I, Mousa A. Prevalence and causes of visual impairment and blindness, cataract surgical coverage and outcomes of cataract surgery in Libya. Ophthalmic Epidemiol. 2013; 20(1): 26-32.
Scientific literature	NA	Country	Algeria, Argentina, Bahrain, Bangladesh, China, Egypt, India, Indonesia, Iran (Islamic Republic of), Jordan, Kuwait, Libya, Malaysia, Mexico, Morocco, Pakistan, Philippines, Qatar, Russian Federation, Saudi Arabia, Singapore, Republic of Korea, Taiwan (Province of China), Tunisia, Turkiye, United Arab Emirates, Yemen	01/2009 to 12/2010	Litwak L, Goh SY, Hussein Z, Malek R, Prusty V, Khamseh ME. Prevalence of diabetes complications in people in type 2 diabetes mellitus and its association with baseline characteristics in the multinational A1chieve study . Diabetol Metab Syndr. 2013; 5(1): 57.
Scientific literature	NA	NA	Libya	01/1998 to 12/1999	Kadiki OA, Roaeid RB. Prevalence of diabetes mellitus and impaired glucose tolerance in Benghazi Libya. Diabetes Metab. 2001; 27(6): 647-54.

Data type	Secondary data type	Coverage type	Geography	Time period covered	Suggested citation
Scientific literature	NA	NA	Libya	01/1981 to 12/1990	Kadiki OA, Moawad SE. Ten-year incidence (1981-90) of insulin-dependent diabetes in the 0-29-year-old age group in Benghazi, Libya. Diabetes Res Clin Pract. 1994; 26(3): 223-8.
Survey	GPS coordinates (GIS), Household, Individual, Interview, Nationally representative	Country	Morocco	10/2003 to 02/2004	League of Arab States, Macro International, Inc, Ministry of Health (Morocco). Morocco Demographic and Health Survey 2003-2004. Fairfax, United States of America: ICF International.
Report	NA	Country	Morocco	01/1994 to 12/2003	Ministry of Health (Morocco). Morocco Health in Figures 2004.
Report	NA	Country	Morocco	01/1995 to 12/2004	Ministry of Health (Morocco). Morocco Health in Figures 2005.
Report	Subnationally representative	Country	Morocco	01/1995 to 12/2005	Ministry of Health (Morocco). Morocco Health in Figures 2006.
Report	Subnationally representative	Country	Morocco	01/1996 to 06/2007	Ministry of Health (Morocco). Morocco Health in Figures 2006 Edition 2007.
Report	Subnationally representative	Country	Morocco	01/1996 to 12/2007	Ministry of Health (Morocco). Morocco Health in Figures 2007 Edition 2008.
Report	Subnationally representative	Country	Morocco	01/1997 to 06/2009	Ministry of Health (Morocco). Morocco Health in Figures 2008.
Report	Subnationally representative	Country	Morocco	01/2000 to 12/2009	Ministry of Health (Morocco). Morocco Health in Figures 2009.
Report	Subnationally representative	Country	Morocco	01/2001 to 12/2011	Ministry of Health (Morocco). Morocco Health in Figures 2010.
Report	Subnationally representative	Country	Morocco	01/2002 to 12/2011	Ministry of Health (Morocco). Morocco Health in Figures 2011.
Report	Subnationally representative	Country	Morocco	01/2004 to 12/2013	Ministry of Health (Morocco). Morocco Health in Figures 2012. Rabat, Morocco: Ministry of Health (Morocco).
Report	Epi surveillance, Subnationally representative, Urban-rural representative	Country	Morocco	01/1998 to 12/2014	Ministry of Health (Morocco). Morocco Health in Figures 2014. Rabat, Morocco: Ministry of Health (Morocco).
Report	Subnationally representative, Urban-rural representative, Epi surveillance, Nationally representative	Country	Morocco	01/2004 to 12/2015	Ministry of Health (Morocco). Morocco Health in Figures 2015. Rabat, Morocco: Ministry of Health (Morocco), 2016.
Report	Epi surveillance, Nationally representative, Subnationally representative, Urban-rural representative	Country	Morocco	01/1998 to 12/2016	Ministry of Health (Morocco). Morocco Health in Figures 2016. Rabat, Morocco: Ministry of Health (Morocco).
Report	Epi surveillance, Nationally representative, Subnationally representative, Urban-rural representative	Country	Morocco	01/2010 to 12/2017	Ministry of Health (Morocco). Morocco Health in Figures 2017. Rabat, Morocco: Ministry of Health (Morocco).
Survey	Cross-sectional, Household, Individual, Nationally representative, Urban-rural representative	Country	Morocco	11/2010 to 03/2011	Ministry of Health (Morocco), Pan Arab Project for Family Health (PAPFAM), United Nations Children's Fund (UNICEF), United Nations Population Fund (UNFPA), World Health Organization (WHO). Morocco National Survey on Population and Family Health 2010-2011.
Survey	Cross-sectional, Household, Individual, Nationally representative, Urban-rural representative	Country	Morocco	10/2017 to 01/2018	Ministry of Health (Morocco), Pan Arab Project for Family Health (PAPFAM), United Nations Children's Fund (UNICEF), United Nations Population Fund (UNFPA), World Health Organization (WHO). Morocco National Survey on Population and Family Health 2017-2018.
Survey	Household	Country	Morocco	03/2017 to 05/2017	Ministry of Health (Morocco), World Health Organization (WHO). Morocco STEPS Noncommunicable Disease Risk Factors Survey 2017.
Survey	Cross-sectional, GPS coordinates (GIS), Household, Individual, Interview, Nationally representative, Verbal autopsy	Country	Morocco	04/2003 to 07/2003	World Health Organization (WHO). Morocco World Health Survey 2003. Geneva, Switzerland: World Health Organization (WHO), 2005.

Data type	Secondary data type	Coverage type	Geography	Time period covered	Suggested citation
Scientific literature	NA	Country	Algeria, Argentina, Bahrain, Bangladesh, China, Egypt, India, Indonesia, Iran (Islamic Republic of), Jordan, Kuwait, Libya, Malaysia, Mexico, Morocco, Pakistan, Philippines, Qatar, Russian Federation, Saudi Arabia, Singapore, Republic of Korea, Taiwan (Province of China), Tunisia, Turkiye, United Arab Emirates, Yemen	01/2009 to 12/2010	Litwak L, Goh SY, Hussein Z, Malek R, Prusty V, Khamseh ME. Prevalence of diabetes complications in people in type 2 diabetes mellitus and its association with baseline characteristics in the multinational A1chieve study . Diabetol Metab Syndr. 2013; 5(1): 57.
Scientific literature	NA	NA	Oman	01/2006 to 12/2006	Khandekar RB, Tirumurthy S, Al-Harby S, Moorthy NS, Amir I. Diabetic retinopathy and ocular co-morbidities among persons with diabetes at Sumail Hospital of Oman. Diabetes Technol Ther. 2009; 11(10): 675-9.
Scientific literature	NA	NA	Oman	01/2000 to 12/2001	Khandekar R, Al Lawatii J, Mohammed AJ, Al Raisi A. Diabetic retinopathy in Oman: a hospital based study. Br J Ophthalmol. 2003; 87(9): 1061-4.
Scientific literature	NA	NA	Oman	01/1991 to 12/1991	Asfour MG, Lambourne A, Soliman A, Al-Behlani S, Al-Asfoor D, Bold A, Mahtab H, King H. High prevalence of diabetes mellitus and impaired glucose tolerance in the Sultanate of Oman: results of the 1991 national survey. Diabet Med. 1995; 12(12): 1122-5.
Survey	Household	Subnational	Oman, Ash Sharqf´yah	03/2006 to 06/2006	Ministry of Health (Oman), World Health Organization (WHO). Oman - Ash Sharqf´yah STEPS Noncommunicable Disease Risk Factors Survey 2006.
Report	NA	Country	Oman	01/2009 to 12/2009	Ministry of Health (Oman). Oman Annual Health Report 2009. Muscat, Oman: Ministry of Health (Oman)
Report	Subnationally representative	Country	Oman	01/2012 to 12/2012	Ministry of Health (Oman). Oman Annual Health Report 2012. Muscat, Oman: Ministry of Health (Oman).
Report	Discharge, Epi surveillance, Inpatient, Nationally representative, Outpatient, Subnationally representative	Country	Oman	01/1970 to 12/2016	Ministry of Health (Oman). Oman Annual Health Report 2016. Muscat, Oman: Ministry of Health (Oman).
Report	Discharge, Epi surveillance, Inpatient, Outpatient, Subnationally representative	Country	Oman	01/1985 to 12/2018	Ministry of Health (Oman). Oman Annual Health Report 2018. Muscat, Oman: Ministry of Health (Oman).
Report	Discharge, Epi surveillance, Inpatient, Nationally representative, Outpatient, Subnationally representative	Country	Oman	01/1980 to 12/1989	Ministry of Health (Oman). Oman Annual Statistical Report 1989. Muscat, Oman: Ministry of Health (Oman).
Report	Discharge, Epi surveillance, Inpatient, Nationally representative, Outpatient, Subnationally representative	Country	Oman	01/1980 to 12/1990	Ministry of Health (Oman). Oman Annual Statistical Report 1990. Muscat, Oman: Ministry of Health (Oman).
Report	Discharge, Epi surveillance, Inpatient, Nationally representative, Outpatient, Subnationally representative	Country	Oman	01/1980 to 12/1991	Ministry of Health (Oman). Oman Annual Statistical Report 1991. Muscat, Oman: Ministry of Health (Oman).
Report	Discharge, Epi surveillance, Inpatient, Nationally representative, Outpatient, Subnationally representative	Country	Oman	01/1980 to 12/1992	Ministry of Health (Oman). Oman Annual Statistical Report 1992. Muscat, Oman: Ministry of Health (Oman).
Report	Discharge, Epi surveillance, Inpatient, Nationally representative, Outpatient, Subnationally representative	Country	Oman	01/1980 to 12/1993	Ministry of Health (Oman). Oman Annual Statistical Report 1993. Muscat, Oman: Ministry of Health (Oman).
Report	Discharge, Epi surveillance, Inpatient, Outpatient, Subnationally representative	Country	Oman	01/1994 to 12/1994	Ministry of Health (Oman). Oman Annual Statistical Report 1994. Muscat, Oman: Ministry of Health (Oman), 1995.
Survey	Household, Individual, Interview	Country	Oman	01/1995 to 12/1995	Health Ministers,Äô Council for GCC States, Ministry of Health (Oman). Oman Family Health Survey 1995.
Survey	Household	Country	Oman	01/2000 to 03/2000	Ministry of Health (Oman). Oman National Health Survey 2000. Muscat, Oman: Ministry of Health (Oman).
Survey	Cross-sectional, Individual, Interview, Nationally representative	Country	Oman	01/2017 to 04/2017	Ministry of Health (Oman), World Health Organization (WHO). Oman STEPS Noncommunicable Disease Risk Factors Survey 2017.

Data type	Secondary data type	Coverage type	Geography	Time period covered	Suggested citation
Survey	Cross-sectional, Household, Individual, Interview, Nationally representative	Country	Oman	12/2007 to 05/2008	Ministry of Health (Oman), World Health Organization (WHO). Oman World Health Survey 2007-2008.
Scientific literature	NA	NA	Oman, Ash Sharqí´yah, Saudi Arabia	01/2004 to 12/2005	Almajwal AM, Al-Baghli NA, Batterham MJ, Williams PG, Al-Turki KA, Al-Ghamdi AJ. Performance of body mass index in predicting diabetes and hypertension in the Eastern Province of Saudi Arabia. Ann Saudi Med. 2009; 29(6): 437,Äì45.
Scientific literature	NA	NA	Oman	01/1996 to 12/1997	el Haddad OA, Saad MK. Prevalence and risk factors for diabetic retinopathy among Omani diabetics. Br J Ophthalmol. 1998; 82(8): 901-6.
Scientific literature	NA	NA	Oman	01/2001 to 12/2001	Al-Lawati JA, Mohammed AJ, Al-Hinai HQ, Jousilahti P. Prevalence of the metabolic syndrome among Omani adults. Diabetes Care. 2003; 26(6): 1781-5.
Scientific literature	NA	NA	Palestine	01/1998 to 12/1998	Abdul-Rahim HF, Husseini A, Giacaman R, Jervell J, Bjertness E. Diabetes mellitus in an urban Palestinian population: prevalence and associated factors. East Mediterr Health J. 2001; 7(1-2): 67-78.
Scientific literature	NA	NA	Palestine	01/2005 to 12/2005	Abu Sham,Äôa R a. H, Darwazah AK, Kufri FH, Yassin IH, Torok NI. MetS and cardiovascular risk factors among Palestinians of East Jerusalem. East Mediterr Health J. 2009; 15(6): 1464,Äì73.
Survey	Cross-sectional, Household, Individual, Interview, Subnationally representative, Urban-rural representative	Country	Palestine, Gaza Strip, West Bank	04/2000 to 05/2000	Ministry of Health (Palestine), Palestinian Central Bureau of Statistics, United Nations Children's Fund (UNICEF), United Nations Population Fund (UNFPA). Palestine - West Bank and Gaza Strip Multiple Indicator Cluster Survey 2000. Ramallah, Palestine: Palestinian Central Bureau of Statistics.
Survey	Cross-sectional, Household, Individual, Interview, Subnationally representative, Urban-rural representative	Country	Palestine, Gaza Strip, West Bank	04/2000 to 05/2000	Ministry of Health (Palestine), Palestinian Central Bureau of Statistics, United Nations Children's Fund (UNICEF), United Nations Population Fund (UNFPA). Palestine - West Bank and Gaza Strip Multiple Indicator Cluster Survey 2000 - UNICEF. Ramallah, Palestine: Palestinian Central Bureau of Statistics.
Survey	Household, Nationally representative	Country	Palestine	05/2004 to 07/2004	Palestinian Central Bureau of Statistics. Palestine Demographic and Health Survey 2004.
Survey	Household	Country	Palestine	01/2005 to 01/2006	Palestinian Central Bureau of Statistics. Palestine Domestic Violence Survey 2005-2006.
Survey	Cross-sectional, Household, Individual, Interview	Country	Palestine	11/2006 to 03/2007	League of Arab States, Palestinian Central Bureau of Statistics, United Nations Children's Fund (UNICEF). Palestine Family Health Survey 2006-2007.
Report	Epi surveillance, Nationally representative, Subnationally representative	Country	Palestine	01/2000 to 12/2018	Ministry of Health (Palestine). Palestine Health Annual Report 2018. Nablus, Palestine: Ministry of Health (Palestine), 2019.
Report	Subnationally representative	Country	Palestine	01/1996 to 12/1999	Ministry of Health (Palestine). Palestine Health Status Annual Report 1999. Nablus, Palestine: Ministry of Health (Palestine).
Report	Subnationally representative	Country	Palestine	01/2000 to 12/2000	Ministry of Health (Palestine). Palestine Health Status Annual Report 2000. Nablus, Palestine: Ministry of Health (Palestine), 2001.
Report	Epi surveillance, Subnationally representative	Country	Palestine	01/1997 to 12/2001	Ministry of Health (Palestine). Palestine Health Status Annual Report 2001. Nablus, Palestine: Ministry of Health (Palestine), 2002.
Report	Epi surveillance, Subnationally representative	Country	Palestine	01/2002 to 12/2011	Ministry of Health (Palestine). Palestine Health Status Annual Report 2011. Nablus, Palestine: Ministry of Health (Palestine), 2012.
Report	Epi surveillance, Subnationally representative	Country	Palestine	01/2002 to 12/2012	Ministry of Health (Palestine). Palestine Health Status Annual Report 2012. Nablus, Palestine: Ministry of Health (Palestine), 2013.
Report	Epi surveillance, Subnationally representative	Country	Palestine	01/2007 to 12/2013	Ministry of Health (Palestine). Palestine Health Status Annual Report 2013. Nablus, Palestine: Ministry of Health (Palestine), 2014.
Report	Epi surveillance, Subnationally representative	Country	Palestine	01/2008 to 12/2014	Ministry of Health (Palestine). Palestine Health Status Annual Report 2014. Nablus, Palestine: Ministry of Health (Palestine), 2015.
Report	Epi surveillance, Subnationally representative	Country	Palestine	01/2009 to 12/2015	Ministry of Health (Palestine). Palestine Health Status Annual Report 2015. Nablus, Palestine: Ministry of Health (Palestine), 2016.
Report	Epi surveillance, Subnationally representative	Country	Palestine	01/2009 to 12/2016	Ministry of Health (Palestine). Palestine Health Status Annual Report 2016. Nablus, Palestine: Ministry of Health (Palestine), 2016.
Report	Epi surveillance, Subnationally representative	Country	Palestine	01/1988 to 12/2017	Ministry of Health (Palestine). Palestine Health Status Annual Report 2017. Nablus, Palestine: Ministry of Health (Palestine), 2018.
Survey	Cross-sectional, Individual, Interview, Nationally representative, Subnationally representative, Urban-rural representative	Country	Palestine	05/2010 to 09/2010	Ministry of Health (Palestine), Palestinian Central Bureau of Statistics, United Nations Children's Fund (UNICEF), United Nations Population Fund (UNFPA). Palestine Multiple Indicator Cluster Survey 2010. New York, United States of America: United Nations Children's Fund (UNICEF), 2014.

Data type	Secondary data type	Coverage type	Geography	Time period covered	Suggested citation
Survey	Cross-sectional, Household, Individual, Interview, Nationally representative	Country	Palestine	10/1999 to 10/2000	Al-Quds Nutrition and Health Research Institute, Al-Quds University, Ministry of Health (Palestine), Operational Research Laboratory for Health and Nutrition, Al-Quds University, Palestinian Central Bureau of Statistics. Palestine National Health And Nutrition Survey 1999-2000.
Survey	Cross-sectional, Household, Individual, Interview, Nationally representative, Subnationally representative, Urban-rural representative	Country	Palestine	03/2002 to 06/2002	Birzeit University, Palestinian Central Bureau of Statistics. Palestine Nutrition Survey 2002. Ramallah, Palestine: Palestinian Central Bureau of Statistics.
Survey	Household, Interview	Country	Palestine	04/2010 to 03/2011	Ministry of Health (Palestine), World Health Organization (WHO). Palestine STEPS Noncommunicable Disease Risk Factors Survey 2010-2011. Geneva, Switzerland: World Health Organization (WHO).
Scientific literature	NA	NA	Palestine	01/1996 to 12/1996	Husseini A, Abdul-Rahim H, Awartani F, Jervell J, Bjertness E. Prevalence of diabetes mellitus and impaired glucose tolerance in a rural Palestinian population. East Mediterr Health J. 2000; 6(5-6): 1039-45.
Scientific literature	NA	NA	Palestine	01/2015 to 12/2015	Abu Al-Halaweh A, Davidovitch N, Almdal TP, Cowan A, Khatib S, Nasser-Eddin L, Baradia Z. Prevalence of type 2 diabetes mellitus complications among palestinians with T2DM. 2017; 11 Suppl 2: S783-S787.
Scientific literature	NA	NA	Palestine	01/2008 to 12/2008	Chiang F, Kuper H, Lindfield R, Keenan T, Seyam N, Magauran D, Khalilia N, Batta H, Abdeen Z, Sargent N. Rapid assessment of avoidable blindness in the Occupied Palestinian Territories. PLoS One. 2010; 5(7): e11854.
Scientific literature	NA	NA	Palestine	01/1999 to 12/2000	Husseini A, Abdul-Rahim H, Awartani F, Giacaman R, Jervell J, Bjertness E. Type 2 diabetes mellitus, impaired glucose tolerance and associated factors in a rural Palestinian village. Diabet Med. 2000; 17(10): 746-8.
Scientific literature	NA	NA	Qatar	01/2009 to 12/2009	Elshafei M, Gamra H, Khandekar R, Al Hashimi M, Pai A, Ahmed MF. Prevalence and determinants of diabetic retinopathy among persons ,â• 40 years of age with diabetes in Qatar: a community-based survey. Eur J Ophthalmol. 2011; 21(1): 39-47.
Scientific literature	NA	Country	Algeria, Argentina, Bahrain, Bangladesh, China, Egypt, India, Indonesia, Iran (Islamic Republic of), Jordan, Kuwait, Libya, Malaysia, Mexico, Morocco, Pakistan, Philippines, Qatar, Russian Federation, Saudi Arabia, Singapore, Republic of Korea, Taiwan (Province of China), Tunisia, Turkiye, United Arab Emirates, Yemen	01/2009 to 12/2010	Litwak L, Goh SY, Hussein Z, Malek R, Prusty V, Khamseh ME. Prevalence of diabetes complications in people in type 2 diabetes mellitus and its association with baseline characteristics in the multinational A1chieve study . Diabetol Metab Syndr. 2013; 5(1): 57.
Scientific literature	NA	NA	Qatar	01/2008 to 12/2008	Bener A, Zirie M, Janahi IM, Al-Hamaq AO, Musallam M, Wareham NJ. Prevalence of diagnosed and undiagnosed diabetes mellitus and its risk factors in a population-based study of Qatar. Diabetes Res Clin Pract. 2009; 84(1): 99-106.
Administrative data	Discharge, Inpatient	Country	Qatar	01/2002 to 12/2002	Hamad Medical Corporation (Qatar). Qatar - Annual Inpatients Discharge Abstract: Hamad General Hospital 2002. Doha, Qatar: Hamad Medical Corporation (Qatar).
Administrative data	Discharge, Inpatient	Country	Qatar	01/2003 to 12/2003	Hamad Medical Corporation (Qatar). Qatar - Annual Inpatients Discharge Abstract: Hamad General Hospital and Women's Hospital 2003. Doha, Qatar: Hamad Medical Corporation (Qatar).
Survey	Cross-sectional, Household, Individual, Interview, Nationally representative	Country	Qatar	03/1998 to 05/1998	Arab Fund for Economic and Social Development (AFESD), Arab Gulf Program for Development (AGFUND), Central Statistical Organization (Qatar), Health Ministers,Äô Council for GCC States, Ministry of Public Health (Qatar), United Nations Children's Fund (UNICEF), United Nations Population Fund (UNFPA), United Nations Statistics Division (UNSD), World Health Organization (WHO). Qatar Family Health Survey 1998.
Survey	Household, Interview, Nationally representative	Country	Qatar	03/2012 to 05/2012	Qatar Statistics Authority, Supreme Council of Health (Qatar), World Health Organization (WHO). Qatar STEPS Noncommunicable Disease Risk Factors Survey 2012. Geneva, Switzerland: World Health Organization (WHO).
Vital registration	Subnationally representative	Country	Qatar	01/2005 to 12/2014	Ministry of Development Planning and Statistics (Qatar), Supreme Council of Health (Qatar). Qatar Vital Statistics Annual Bulletin 2014. Doha, Qatar: Ministry of Development Planning and Statistics (Qatar), 2016.
Vital registration	Subnationally representative	Country	Qatar	01/2006 to 12/2015	Ministry of Development Planning and Statistics (Qatar), Supreme Council of Health (Qatar). Qatar Vital Statistics Annual Bulletin 2015. Doha, Qatar: Ministry of Development Planning and Statistics (Qatar), 2016.
Vital registration	Subnationally representative	Country	Qatar	01/2007 to 12/2016	Ministry of Development Planning and Statistics (Qatar), Supreme Council of Health (Qatar). Qatar Vital Statistics Annual Bulletin 2016. Doha, Qatar: Ministry of Development Planning and Statistics (Qatar), 2017.

Data type	Secondary data type	Coverage type	Geography	Time period covered	Suggested citation
Vital registration	Subnationally representative	Country	Qatar	01/2007 to 12/2017	Ministry of Development Planning and Statistics (Qatar), Supreme Council of Health (Qatar). Qatar Vital Statistics Annual Bulletin 2017. Doha, Qatar: Ministry of Development Planning and Statistics (Qatar), 2018.
Vital registration	Subnationally representative	Country	Qatar	01/2009 to 12/2018	Planning and Statistics Authority (PSA) (Qatar). Qatar Vital Statistics Annual Bulletin 2018. Planning and Statistics Authority (PSA) (Qatar), 2019.
Survey	NA	Country	Qatar	04/2006 to 05/2006	National Health Authority (Qatar), Qatar Statistics Authority, World Health Organization (WHO). Qatar World Health Survey 2006.
Scientific literature	NA	NA	Syrian Arab Republic	01/2006 to 12/2006	Albache N, Al Ali R, Rastam S, Fouad FM, Mzayek F, Maziak W. Epidemiology of Type 2 diabetes mellitus in Aleppo, Syria. J Diabetes. 2010; 2(2): 85-91.
Survey	Household, Subnationally representative	Country	Syrian Arab Republic	03/2006 to 05/2006	General Administration for Palestine Arab Refugees (GAPAR), Palestinian Central Bureau of Statistics, Pan Arab Project for Family Health (PAPFAM), United Nations Children's Fund (UNICEF). Palestinians in Syria Multiple Indicator Cluster Survey 2006.
Survey	Household	Subnational	Syrian Arab Republic, ·∏®alab	05/2004 to 08/2004	Syrian Center for Tobacco Studies. Syria - Aleppo Household Survey 2004.
Scientific literature	NA	NA	Saudi Arabia	01/1989 to 12/2004	Alwakeel JS, Al-Suwaida A, Isnani AC, Al-Harbi A, Alam A. Concomitant macro and microvascular complications in diabetic nephropathy. Saudi J Kidney Dis Transpl. 2009; 20(3): 402-9.
Scientific literature	NA	NA	Saudi Arabia, Ar RiyƒÅ·∏ë	01/2009 to 12/2010	Al-Daghri NM, Al-Attas OS, Alokail MS, Alkharfy KM, Sabico SLB, Chrousos GP. Decreasing prevalence of the full metabolic syndrome but a persistently high prevalence of dyslipidemia among adult Arabs. PLoS One. 2010; 5(8): e12159.
Scientific literature	NA	NA	Saudi Arabia	01/1994 to 12/1995	El-Hazmi MA, Warsy AS, Al-Swailem AR, Al-Swailem AM, Sulaimani R, Al-Meshari AA. Diabetes mellitus and impaired glucose tolerance in Saudi Arabia. Ann Saudi Med. 1996; 16(4): 381-5.
Scientific literature	NA	NA	Saudi Arabia	01/1998 to 12/1998	Al-Nozha MM, Al-Maatouq MA, Al-Mazrou YY, Al-Harthi SS, Arafah MR, Khalil MZ, Khan NB, Al-Khadra A, Al-Marzouki K, Nouh MS, Abdullah M, Attas O, Al-Shahid MS, Al-Mobeireek A. Diabetes mellitus in Saudi Arabia. Saudi Med J. 2004; 25(11): 1603-10.
Scientific literature	NA	NA	Saudi Arabia	01/2009 to 12/2010	Al-Daghri NM, Al-Attas OS, Alokail MS, Alkharfy KM, Yousef M, Sabico SL, Chrousos GP. Diabetes mellitus type 2 and other chronic non-communicable diseases in the central region, Saudi Arabia (Riyadh cohort 2): a decade of an epidemic. BMC Med. 2011; 76.
Scientific literature	NA	NA	Saudi Arabia	01/1994 to 12/1994	Warsy AS, el-Hazmi MA. Diabetes mellitus, hypertension and obesity--common multifactorial disorders in Saudis. East Mediterr Health J. 1999; 5(6): 1236-42.
Scientific literature	NA	NA	Saudi Arabia	01/2000 to 12/2012	Al-Rubeaan K, Al Derwish M, Ouizi S, Youssef AM, Subhani SN, Ibrahim HM, Alamri BN. Diabetic foot complications and their risk factors from a large retrospective cohort study. PLoS One. 2015; 10(5): e0124446.
Scientific literature	NA	NA	Saudi Arabia	01/1985 to 12/1987	Anokute CC. Epidemiologic studies of diabetes mellitus in Saudi Arabia--Part I--Screening of 3158 males in King Saud University. J R Soc Health. 1990; 110(6): 201-3.
Scientific literature	NA	NA	Saudi Arabia	01/2004 to 12/2009	Habeb AM, Al-Magamsi MS, Halabi S, Eid IM, Shalaby S, Bakoush O. High incidence of childhood type 1 diabetes in Al-Madinah, North West Saudi Arabia (2004-2009). Pediatr Diabetes. 2011; 12(8): 676-81.
Scientific literature	NA	NA	Saudi Arabia	01/1990 to 12/2007	Al-Mendalawi MD, Abduljabbar MA, Aljubeh JM, Amalraj A, Cherian MP. Incidence trends of childhood type 1 diabetes in eastern Saudi Arabia. Saudi Med J. 2010; 31(9): 1074-1075.
Scientific literature	NA	NA	Saudi Arabia	01/2001 to 12/2010	Habeb AM, Al-Magamsi MSF, Eid IM, Ali MI, Hattersley AT, Hussain K, Ellard S. Incidence, genetics, and clinical phenotype of permanent neonatal diabetes mellitus in northwest Saudi Arabia. Pediatr Diabetes. 2012; 13(6): 499-505.
Scientific literature	NA	NA	Saudi Arabia	01/2007 to 12/2009	Al-Rubeaan K. National surveillance for type 1, type 2 diabetes and prediabetes among children and adolescents: a population-based study (SAUDI-DM). J Epidemiol Community Health. 2015; nan.
Scientific literature	NA	NA	Oman, Ash Sharqƒ´yah, Saudi Arabia	01/2004 to 12/2005	Almajwal AM, Al-Baghli NA, Batterham MJ, Williams PG, Al-Turki KA, Al-Ghamdi AJ. Performance of body mass index in predicting diabetes and hypertension in the Eastern Province of Saudi Arabia. Ann Saudi Med. 2009; 29(6): 437‚Äì45.
Scientific literature	NA	NA	Saudi Arabia	01/1995 to 12/1996	Nielsen JV. Peripheral neuropathy, hypertension, foot ulcers and amputations among Saudi Arabian patients with type 2 diabetes. Diabetes Res Clin Pract. 1998; 41(1): 63-9.
Scientific literature	NA	NA	Saudi Arabia	01/2011 to 12/2012	Hajar S, Al Hazmi A, Wasli M, Mousa A, Rabiu M. Prevalence and causes of blindness and diabetic retinopathy in Southern Saudi Arabia. Saudi Med J. 2015; 36(4): 449-55.
Scientific literature	NA	NA	Saudi Arabia	01/2009 to 12/2010	Wang DD, Bakhotmah BA, Hu FB, Alzahrani HA. Prevalence and correlates of diabetic peripheral neuropathy in a Saudi Arabic population: a cross-sectional study. PLoS One. 2014; 9(9): e106935.
Scientific literature	NA	NA	Saudi Arabia	01/2007 to 12/2009	Khan AR, Wiseberg JA, Lateef ZA, Khan SA. Prevalence and determinants of diabetic retinopathy in Al hasa region of Saudi Arabia: primary health care centre based cross-sectional survey, 2007-2009. Middle East Afr J Ophthalmol. 2010; 17(3): 257-63.

Data type	Secondary data type	Coverage type	Geography	Time period covered	Suggested citation
Scientific literature	NA	Country	Algeria, Argentina, Bahrain, Bangladesh, China, Egypt, India, Indonesia, Iran (Islamic Republic of), Jordan, Kuwait, Libya, Malaysia, Mexico, Morocco, Pakistan, Philippines, Qatar, Russian Federation, Saudi Arabia, Singapore, Republic of Korea, Taiwan (Province of China), Tunisia, Turkiye, United Arab Emirates, Yemen	01/2009 to 12/2010	Litwak L, Goh SY, Hussein Z, Malek R, Prusty V, Khamseh ME. Prevalence of diabetes complications in people in type 2 diabetes mellitus and its association with baseline characteristics in the multinational A1chieve study . Diabetol Metab Syndr. 2013; 5(1): 57.
Scientific literature	NA	NA	Saudi Arabia	01/2004 to 12/2005	Al-Baghli NA, Al-Ghamdi AJ, Al-Turki KA, Al Elq AH, El-Zubaier AG, Bahnassy A. Prevalence of diabetes mellitus and impaired fasting glucose levels in the Eastern Province of Saudi Arabia: results of a screening campaign. Singapore Med J. 2010; 51(12): 923-30.
Scientific literature	NA	NA	Saudi Arabia	01/2009 to 12/2009	Alqurashi KA, Aljabri KS, Bokhari SA. Prevalence of diabetes mellitus in a Saudi community. Ann Saudi Med. 2011; 31(1): 19-23.
Scientific literature	NA	NA	Saudi Arabia	01/1990 to 12/2006	Al-Herbish AS, El-Mouzan MI, Al-Salloum AA, Al-Qurachi MM, Al-Omar AA. Prevalence of type 1 diabetes mellitus in Saudi Arabian children and adolescents. Saudi Med J. 2008; 29(9): 1285-8.
Scientific literature	NA	NA	Saudi Arabia	01/2006 to 12/2007	Halawa MR,¬†Karawagh A,¬†Zeidan A,¬†Mahmoud AE,¬†Sakr M,¬†Hegazy A. Prevalence¬†of¬†painful¬†diabetic¬†peripheral¬†neuropathy¬†among¬†patients¬†suffering¬†from¬†diabetes¬†mellitus¬†in¬†Saudi Arabia. Curr Med Res Opin. 2010; 26(2): 337-43.
Scientific literature	NA	Subnational	Saudi Arabia	01/2009 to 12/2011	Al Ghamdi AH, Rabiu M, Hajar S, Yorston D, Kuper H, Polack S. Rapid assessment of avoidable blindness and diabetic retinopathy in Taif, Saudi Arabia. Br J Ophthalmol. 2012; 96(9): 1168-72.
Scientific literature	NA	NA	Saudi Arabia	01/1996 to 12/1997	El-Asrar AM, Al-Rubeaan KA, Al-Amro SA, Kangave D, Moharram OA. Risk factors for diabetic retinopathy among Saudi diabetics. Int Ophthalmol. 1998; 22(3): 155-61.
Report	NA	Country	Saudi Arabia	01/1993 to 12/2012	Ministry of Health (Saudi Arabia), World Health Organization (WHO). Saudi Arabia Country Cooperation Strategy for WHO 2014-2015.
Survey	Cross-sectional, Household, Individual, Interview, Nationally representative, Urban-rural representative	Country	Saudi Arabia	11/1996 to 01/1997	Arab Fund for Economic and Social Development (AFESD), Arab Gulf Program for Development (AGFUND), Gulf-Co-operation Council (GCC), Ministry of Health (Saudi Arabia), United Nations Children's Fund (UNICEF), United Nations Population Fund (UNFPA), United Nations Statistics Division (UNSD), World Health Organization (WHO). Saudi Arabia Family Health Survey 1996-1997.
Survey	Household, Interview, Nationally representative, Subnationally representative	Country	Saudi Arabia	01/2013 to 12/2013	Institute for Health Metrics and Evaluation (IHME), Ministry of Health (Saudi Arabia). Saudi Arabia Health Interview Survey 2013.
Report	NA	Country	Saudi Arabia	01/1994 to 12/1999	Ministry of Health (Saudi Arabia). Saudi Arabia Health Statistical Yearbook 1999. Riyadh, Saudi Arabia: Ministry of Health (Saudi Arabia).
Report	NA	Country	Saudi Arabia	01/1991 to 12/2000	Ministry of Health (Saudi Arabia). Saudi Arabia Health Statistical Yearbook 2000. Riyadh, Saudi Arabia: Ministry of Health (Saudi Arabia).
Report	NA	Country	Saudi Arabia	01/1996 to 12/2001	Ministry of Health (Saudi Arabia). Saudi Arabia Health Statistical Yearbook 2001. Riyadh, Saudi Arabia: Ministry of Health (Saudi Arabia).
Report	NA	Country	Saudi Arabia	Jan-02	Ministry of Health (Saudi Arabia). Saudi Arabia Health Statistical Yearbook 2002. Riyadh, Saudi Arabia: Ministry of Health (Saudi Arabia).
Report	NA	Country	Saudi Arabia	Jan-06	Ministry of Health (Saudi Arabia). Saudi Arabia Health Statistical Yearbook 2006. Riyadh, Saudi Arabia: Ministry of Health (Saudi Arabia).
Report	NA	Country	Saudi Arabia	Jan-07	Ministry of Health (Saudi Arabia). Saudi Arabia Health Statistical Yearbook 2007. Riyadh, Saudi Arabia: Ministry of Health (Saudi Arabia).
Report	NA	Country	Saudi Arabia	Jan-08	Ministry of Health (Saudi Arabia). Saudi Arabia Health Statistical Yearbook 2008. Riyadh, Saudi Arabia: Ministry of Health (Saudi Arabia).
Report	Discharge, Emergency, Inpatient, Outpatient	Country	Saudi Arabia	01/2006 to 12/2010	Ministry of Health (Saudi Arabia). Saudi Arabia Health Statistical Yearbook 2010. Riyadh, Saudi Arabia: Ministry of Health (Saudi Arabia).
Report	Discharge, Emergency, Inpatient, Outpatient	Country	Saudi Arabia	01/2007 to 12/2011	Ministry of Health (Saudi Arabia). Saudi Arabia Health Statistical Yearbook 2011. Riyadh, Saudi Arabia: Ministry of Health (Saudi Arabia).
Report	Discharge, Emergency, Inpatient, Outpatient	Country	Saudi Arabia	01/2008 to 12/2012	Ministry of Health (Saudi Arabia). Saudi Arabia Health Statistical Yearbook 2012. Riyadh, Saudi Arabia: Ministry of Health (Saudi Arabia).
Report	NA	Country	Saudi Arabia	01/2007 to 12/2013	Ministry of Health (Saudi Arabia). Saudi Arabia Health Statistical Yearbook 2013. Riyadh, Saudi Arabia: Ministry of Health (Saudi Arabia).
Report	NA	Country	Saudi Arabia	01/2005 to 12/2014	Ministry of Health (Saudi Arabia). Saudi Arabia Health Statistical Yearbook 2014. Riyadh, Saudi Arabia: Ministry of Health (Saudi Arabia).

Data type	Secondary data type	Coverage type	Geography	Time period covered	Suggested citation
Report	Epi surveillance, Subnationally representative	Country	Saudi Arabia	01/2006 to 12/2015	Ministry of Health (Saudi Arabia). Saudi Arabia Health Statistical Yearbook 2015. Riyadh, Saudi Arabia: Ministry of Health (Saudi Arabia).
Report	Epi surveillance, Subnationally representative	Country	Saudi Arabia	01/2007 to 12/2016	Ministry of Health (Saudi Arabia). Saudi Arabia Health Statistical Yearbook 2016. Riyadh, Saudi Arabia: Ministry of Health (Saudi Arabia).
Report	Epi surveillance, Subnationally representative	Country	Saudi Arabia	01/2013 to 12/2017	Ministry of Health (Saudi Arabia). Saudi Arabia Health Statistical Yearbook 2017. Riyadh, Saudi Arabia: Ministry of Health (Saudi Arabia).
Report	Epi surveillance, Subnationally representative	Country	Saudi Arabia	01/2014 to 12/2018	Ministry of Health (Saudi Arabia). Saudi Arabia Health Statistical Yearbook 2018. Riyadh, Saudi Arabia: Ministry of Health (Saudi Arabia).
Survey	NA	Country	Saudi Arabia	01/2011 to 12/2012	King Abdulaziz City for Science and Technology (KACST), King Abdullah International Medical Research Center, Ministry of Education (Saudi Arabia). Saudi Arabia National Assessment of the Health Needs of Adolescents 2011-2012.
Survey	Health facility, Longitudinal	Subnational	Saudi Arabia	01/2012 to 01/2015	Hamilton Health Sciences, McMaster University (Canada), Population Health Research Institute (PHRI). Saudi Arabia Prospective Urban and Rural Epidemiological Study.
Survey	Cross-sectional, Household, Individual, Interview, Nationally representative	Country	Saudi Arabia	08/2004 to 12/2005	Ministry of Health (Saudi Arabia), World Health Organization (WHO). Saudi Arabia STEPS Noncommunicable Disease Risk Factors Survey 2004-2005.
Scientific literature	NA	NA	Saudi Arabia	01/2011 to 12/2011	Elsharawy MA, Hassan K, Alawad N, Kredees A, Almulhim A. Screening of diabetic foot in surgical inpatients: a hospital-based study in saudi arabia. Int J Angiol. 2012; 21(4): 213-6.
Scientific literature	NA	NA	Saudi Arabia	01/1991 to 12/1993	El-Hazmi MA, Al-Swailem A, Warsy AS, Al-Sudairy F, Sulaimani R, Al-Swailem A, Al-Meshari A. The prevalence of diabetes mellitus and impaired glucose tolerance in the population of Riyadh. Ann Saudi Med. 1995; 15(6): 598-601.
Scientific literature	NA	NA	Saudi Arabia	01/2007 to 12/2009	Al-Rubeaan K, Al-Manaa H, Khoja T, Ahmad N, Al-Sharqawi A, Siddiqui K, AlNaqeb D, Aburisheh K, Youssef A, Al-Batil A, Al-Otaibi M, Ghamdi AA. The Saudi Abnormal Glucose Metabolism and Diabetes Impact Study (SAUDI-DM). Ann Saudi Med. 2014; 34(6): 465,Äì75.
Scientific literature	NA	NA	Sudan	01/1986 to 12/1986	Elmahdi EM, Kaballo AM, Mukhtar EA. Features of non-insulin-dependent diabetes mellitus (NIDDM) in the Sudan. Diabetes Res Clin Pract. 1991; 11(1): 59-63.
Scientific literature	NA	NA	Sudan	01/1991 to 12/1995	Elamin A, Ghalib M, Eltayeb B, Tuvemo T. High incidence of type 1 diabetes mellitus in Sudanese children, 1991-1995. Ann Saudi Med. 1997; 17(4): 478-80.
Scientific literature	NA	NA	Algeria, Argentina, Australia, Austria, Barbados, Belgium, Brazil, Bulgaria, Canada, Chile, China, Colombia, Cuba, Denmark, Dominica, Estonia, Finland, France, Germany, Greece, Hungary, Israel, Italy, Japan, Kuwait, Latvia, Lithuania, Luxembourg, Mauritius, Mexico, Netherlands, New Zealand, Norway, Pakistan, Paraguay, Peru, Poland, Portugal, Romania, Russian Federation, Slovakia, Slovenia, Spain, Sudan, Tunisia, United Kingdom, United States of America, Puerto Rico, United States Virgin Islands, Uruguay, Venezuela (Bolivarian Republic of)	01/2000 to 12/2000	Karvonen M, Viik-Kajander M, Moltchanova E, Libman I, LaPorte R, Tuomilehto J. Incidence of childhood type 1 diabetes worldwide. Diabetes Mondiale (DiaMond) Project Group. Diabetes Care. 2000; 23(10): 1516,Äì26.
Scientific literature	NA	NA	Sudan	01/1986 to 12/1986	el Mahdi EM, Abdel Rahman Iel M, Mukhtar Sel D. Pattern of diabetes mellitus in the Sudan. Trop Geogr Med. 1989; 41(4): 353-7.
Scientific literature	NA	NA	Sudan	01/1992 to 12/1992	Elbagir MN, Eltom MA, Mahadi EO, Berne C. Pattern of long-term complications in Sudanese insulin-treated diabetic patients. Diabetes Res Clin Pract. 1995; 30(1): 59-67.
Survey	Household	Subnational	Sudan, Khartoum	12/2005 to 01/2006	Federal Ministry of Health (Sudan), World Health Organization (WHO). Sudan - Khartoum STEPS Noncommunicable Disease Risk Factors Survey 2005-2006.
Report	Discharge, Epi surveillance, Inpatient, Nationally representative, Outpatient, Subnationally representative	Country	Sudan, Sudan [Historical]	01/2007 to 12/2011	Federal Ministry of Health (Sudan). Sudan Annual Health Statistics Report 2011. Khartoum, Sudan: Federal Ministry of Health (Sudan), 2012.

Data type	Secondary data type	Coverage type	Geography	Time period covered	Suggested citation
Scientific literature	NA	NA	Tunisia	01/1990 to 12/1994	Ben Khalifa F, Mekaouar A, Taktak S, Hamhoum M, Jebara H, Kodia A, Zouari B, Chakroun M. A five-year study of the incidence of insulin-dependent diabetes mellitus in young Tunisians (preliminary results). Diabetes Metab. 1997; 23(5): 395-401.
Scientific literature	NA	NA	Tunisia	01/1981 to 12/1981	Papoz L, Ben Khalifa F, Eschwege E, Ben Ayed H. Diabetes mellitus in Tunisia: description in urban and rural populations. Int J Epidemiol. 1988; 17(2): 419-22.
Disease registry	NA	Country	Albania, Austria, Belgium, Bosnia and Herzegovina, Bulgaria, Croatia, Cyprus, Czechia, Denmark, Estonia, Finland, France, Georgia, Greece, Iceland, Israel, Latvia, Lithuania, Macedonia, Montenegro, Netherlands, Norway, Poland, Portugal, Romania, Serbia, Slovakia, Slovenia, Spain, Sweden, Tunisia, Sfax, Turkiye, Ukraine, United Kingdom, England, Northern Ireland, Scotland, Wales	01/2014 to 12/2014	European Renal Association-European Dialysis and Transplant Association (ERA-EDTA). ERA-EDTA Registry Annual Report 2014. Amsterdam, Netherlands: Department of Medical Informatics, Academic Medical Center (The Netherlands), 2016.
Disease registry	NA	Country	Albania, Austria, Belarus, Belgium, Bosnia and Herzegovina, Bulgaria, Croatia, Cyprus, Czechia, Denmark, Estonia, Finland, France, Georgia, Greece, Iceland, Israel, Latvia, Lithuania, Macedonia, Netherlands, Norway, Poland, Portugal, Romania, Russian Federation, Serbia, Slovakia, Slovenia, Spain, Sweden, Switzerland, Tunisia, Sfax, Turkiye, Ukraine, United Kingdom, England, Northern Ireland, Scotland, Wales	01/2015 to 12/2015	European Renal Association-European Dialysis and Transplant Association (ERA-EDTA). ERA-EDTA Registry Annual Report 2015. Amsterdam, Netherlands: Department of Medical Informatics, Academic Medical Center (The Netherlands), 2016.
Disease registry	NA	Country	Albania, Austria, Belarus, Belgium, Bosnia and Herzegovina, Bulgaria, Croatia, Cyprus, Czechia, Denmark, Estonia, Finland, France, Georgia, Greece, Iceland, Israel, Italy, Latvia, Lithuania, Macedonia, Norway, Poland, Portugal, Romania, Russian Federation, Serbia, Slovakia, Spain, Sweden, Switzerland, Tunisia, Sfax, Turkiye, Ukraine, United Kingdom, England, Northern Ireland, Scotland, Wales	01/2016 to 12/2016	European Renal Association-European Dialysis and Transplant Association (ERA-EDTA). ERA-EDTA Registry Annual Report 2016. Amsterdam, Netherlands: Department of Medical Informatics, Academic Medical Center (The Netherlands), 2016.

Data type	Secondary data type	Coverage type	Geography	Time period covered	Suggested citation
Disease registry	NA	Country	Albania, Austria, Belarus, Belgium, Bosnia and Herzegovina, Bulgaria, Croatia, Cyprus, Czechia, Denmark, Estonia, Finland, France, Georgia, Greece, Iceland, Israel, Italy, Latvia, Lithuania, North Macedonia, Norway, Poland, Portugal, Romania, Russian Federation, Serbia, Slovakia, Spain, Sweden, Switzerland, Tunisia, Sfax, Turkiye, Ukraine, United Kingdom, England, Northern Ireland, Scotland, Wales	01/2017 to 12/2017	European Renal Association-European Dialysis and Transplant Association (ERA-EDTA). ERA-EDTA Registry Annual Report 2017. Amsterdam, Netherlands: Department of Medical Informatics, Academic Medical Center (The Netherlands), 2019.
Scientific literature	NA	NA	Tunisia	01/2001 to 12/2001	Ben Romdhane H, Skhiri H, Bougatef S, Ennigrou S, Gharbi D, Chahed MK, Achour N. Hypertension prevalence, awareness, treatment and control: results from a community based survey. Tunis Med. 2005; 83(Suppl 5): 41-6.
Scientific literature	NA	NA	Algeria, Argentina, Australia, Austria, Barbados, Belgium, Brazil, Bulgaria, Canada, Chile, China, Colombia, Cuba, Denmark, Dominica, Estonia, Finland, France, Germany, Greece, Hungary, Israel, Italy, Japan, Kuwait, Latvia, Lithuania, Luxembourg, Mauritius, Mexico, Netherlands, New Zealand, Norway, Pakistan, Paraguay, Peru, Poland, Portugal, Romania, Russian Federation, Slovakia, Slovenia, Spain, Sudan, Tunisia, United Kingdom, United States of America, Puerto Rico, United States Virgin Islands, Uruguay, Venezuela (Bolivarian Republic of)	01/2000 to 12/2000	Karvonen M, Viik-Kajander M, Moltchanova E, Libman I, LaPorte R, Tuomilehto J. Incidence of childhood type 1 diabetes worldwide. Diabetes Mondiale (DiaMond) Project Group. Diabetes Care. 2000; 23(10): 1516,Äì26.
Scientific literature	NA	NA	Tunisia	01/2004 to 12/2005	Allal-Elasmi M, Feki M, Zayani Y, Hsairi M, Haj Taieb S, Jemaa R, Sanhaji H, Omar S, Mebazaa A, Kaabachi N. Prehypertension among adults in Great Tunis region (Tunisia): A population-based study. Pathol Biol. 2012; 60(3): 174,Äì9.
Scientific literature	NA	NA	Tunisia	01/2007 to 12/2011	Kahloun R, Jelliti B, Zaouali S, Attia S, Ben Yahia S, Resnikoff S, Khairallah M. Prevalence and causes of visual impairment in diabetic patients in Tunisia, North Africa. Eye (Lond). 2014; 28(8): 986-91.
Scientific literature	NA	NA	Tunisia	01/2004 to 12/2005	Belfki H, Ben Ali S, Aounallah-Skhiri H, Traissac P, Bougatef S, Maire B, Delpeuch F, Achour N, Ben Romdhane H. Prevalence and determinants of the metabolic syndrome among Tunisian adults: results of the Transition and Health Impact in North Africa (TAHINA) project. Public Health Nutr. 2013; 16(4): 582,Äì90.
Scientific literature	NA	NA	Tunisia	01/1990 to 12/1990	Gharbi M, Akrout M, Zouari B. Prevalence and risk factors of non-insulin-dependent diabetes mellitus in the rural and urban population of Tunisia. Rev Epidemiol Sante Publique. 2002; 50(4): 349-55.
Scientific literature	NA	Country	Algeria, Argentina, Bahrain, Bangladesh, China, Egypt, India, Indonesia, Iran (Islamic Republic of), Jordan, Kuwait, Libya, Malaysia, Mexico, Morocco, Pakistan, Philippines, Qatar, Russian Federation, Saudi Arabia, Singapore, Republic of Korea, Taiwan (Province of China), Tunisia, Turkiye, United Arab Emirates, Yemen	01/2009 to 12/2010	Litwak L, Goh SY, Hussein Z, Malek R, Prusty V, Khamseh ME. Prevalence of diabetes complications in people in type 2 diabetes mellitus and its association with baseline characteristics in the multinational A1chieve study . Diabetol Metab Syndr. 2013; 5(1): 57.

Data type	Secondary data type	Coverage type	Geography	Time period covered	Suggested citation
Scientific literature	NA	NA	Tunisia	01/2005 to 12/2005	Ben Romdhane H, Ben Ali S, Aissi W, Traissac P, Aounallah-Skhiri H, Bougatef S, Maire B, Delpeuch F, Achour N. Prevalence of diabetes in Northern African countries: the case of Tunisia. BMC Public Health. 2014; 86.
Scientific literature	NA	NA	Tunisia	01/2008 to 12/2009	Hammami S, Mehri S, Hajem S, Koubaa N, Souid H, Hammami M. Prevalence of diabetes mellitus among non institutionalized elderly in Monastir City. BMC Endocr Disord. 2012; 15.
Scientific literature	NA	NA	Tunisia	01/1997 to 12/1997	Bouguerra R, Alberti H, Salem LB, Rayana CB, Atti JE, Gaigi S, Slama CB, Zouari B, Alberti K. The global diabetes pandemic: the Tunisian experience. Eur J Clin Nutr. 2007; 61(2): 160-5.
Scientific literature	NA	NA	Tunisia	01/2005 to 12/2005	Allal-Elasmi M, Haj Taieb S, Hsairi M, Zayani Y, Omar S, Sanhaji H, Jemaa R, Feki M, Elati J, Mebazaa A, Kaabachi N. The metabolic syndrome: prevalence, main characteristics and association with socio-economic status in adults living in Great Tunis. Diabetes Metab. 2010; 36(3): 204,Äì8.
Survey	Health facility, Household, Individual, Interview, Nationally representative, Urban-rural representative	Country	Tunisia	06/2001 to 08/2001	League of Arab States, National Office for Family and Population, Ministry of Public Health (Tunisia), Pan Arab Project for Family Health (PAPFAM). Tunisia Family Health Survey 2001.
Disease registry	NA	Country	Tunisia	01/2014 to 12/2014	Tunisia Renal Replacement Therapy Data 2014 - ERA-EDTA
Survey	Cross-sectional, GPS coordinates (GIS), Household, Individual, Interview, Nationally representative, Verbal autopsy	Country	Tunisia	03/2003 to 09/2003	World Health Organization (WHO). Tunisia World Health Survey 2003. Geneva, Switzerland: World Health Organization (WHO), 2005.
Scientific literature	NA	NA	Turkiye	01/2015 to 12/2017	Emral R, Tetiker T, Sahin I, Sari R, Kaya A, Yetkin ƒ∞, Cil SU, Tv̆ºtv̆ºncv̆º NB, IO HAT investigator group. An international survey on hypoglycemia among insulin-treated type I and type II diabetes patients: Turkey cohort of the non-interventional IO HAT study. BMC Endocr Disord. 2018; 18(1): 9.
Scientific literature	NA	NA	Argentina, Australia, Austria, Belarus, Bermuda, Brazil, Canada, Chile, China, Colombia, Croatia, Democratic People's Republic of Korea, Denmark, Estonia, Finland, France, Germany, Hungary, India, Ireland, Israel, Italy, Latvia, Lithuania, Mexico, Netherlands, Norway, Philippines, Poland, Romania, Russian Federation, Slovakia, South Africa, Spain, Sweden, Switzerland, Turkiye, United Kingdom, United States of America, Venezuela (Bolivarian Republic of)	01/2000 to 12/2010	ORIGIN Trial Investigators, Gerstein HC, Bosch J, Dagenais GR, D√≠az R, Jung H, Maggioni AP, Pogue J, Probstfield J, Ramachandran A, Riddle MC, Ryd√©n LE, Yusuf S. Basal insulin and cardiovascular and other outcomes in dysglycemia. N Engl J Med. 2012; 367(4): 319-28.
Scientific literature	NA	NA	Turkiye	01/2001 to 12/2001	Sekuri C, Eser E, Akpinar G, Cakir H, Sitti I, Gulomur O, Ozcan C. Cardiovascular disease risk factors in post-menopausal women in West Anatolia. Jpn Heart J. 2004; 45(1): 119-31.
Scientific literature	NA	Country	Austria, Canada, Chile, Denmark, Germany, Italy, Japan, Netherlands, Republic of Korea, Sweden, Taiwan (Province of China), Thailand, Turkiye, United States of America	01/1974 to 12/2011	Boyle P, Boniol M, Koechlin A, Robertson C, Valentini F, Coppens K, Fairley LL, Boniol M, Zheng T, Zhang Y, Pasterk M, Smans M, Curado MP, Mullie P, Gandini S, Bota M, Bolli GB, Rosenstock J, Autier P. Diabetes and breast cancer risk: a meta-analysis. Br J Cancer. 2012; 107(9): 1608-17.
Scientific literature	NA	NA	Austria, Canada, Israel, Italy, Japan, Norway, Sweden, Switzerland, Taiwan (Province of China), Turkiye, United Kingdom, United States of America	01/1985 to 12/2012	Lee JY1, Jeon I, Kim JW, Song YS, Yoon JM, Park SM. Diabetes mellitus and ovarian cancer risk: a systematic review and meta-analysis of observational studies. Int J Gynaecol Obstet. 2013; 23(3): 402-12.

Data type	Secondary data type	Coverage type	Geography	Time period covered	Suggested citation
Disease registry	NA	Country	Albania, Austria, Belgium, Bosnia and Herzegovina, Bulgaria, Croatia, Cyprus, Czechia, Denmark, Estonia, Finland, France, Georgia, Greece, Iceland, Israel, Latvia, Lithuania, Montenegro, Netherlands, Norway, Poland, Portugal, Romania, Russian Federation, Serbia, Slovakia, Slovenia, Spain, Sweden, Switzerland, Turkiye, Ukraine, United Kingdom, England, Northern Ireland, Scotland, Wales	01/2013 to 12/2013	European Renal Association-European Dialysis and Transplant Association (ERA-EDTA). ERA-EDTA Registry Annual Report 2013. Amsterdam, Netherlands: Department of Medical Informatics, Academic Medical Center (The Netherlands), 2015.
Disease registry	NA	Country	Albania, Austria, Belgium, Bosnia and Herzegovina, Bulgaria, Croatia, Cyprus, Czechia, Denmark, Estonia, Finland, France, Georgia, Greece, Iceland, Israel, Latvia, Lithuania, Macedonia, Montenegro, Netherlands, Norway, Poland, Portugal, Romania, Serbia, Slovakia, Slovenia, Spain, Sweden, Tunisia, Sfax, Turkiye, Ukraine, United Kingdom, England, Northern Ireland, Scotland, Wales	01/2014 to 12/2014	European Renal Association-European Dialysis and Transplant Association (ERA-EDTA). ERA-EDTA Registry Annual Report 2014. Amsterdam, Netherlands: Department of Medical Informatics, Academic Medical Center (The Netherlands), 2016.
Disease registry	NA	Country	Albania, Austria, Belarus, Belgium, Bosnia and Herzegovina, Bulgaria, Croatia, Cyprus, Czechia, Denmark, Estonia, Finland, France, Georgia, Greece, Iceland, Israel, Latvia, Lithuania, Macedonia, Netherlands, Norway, Poland, Portugal, Romania, Russian Federation, Serbia, Slovakia, Slovenia, Spain, Sweden, Switzerland, Tunisia, Sfax, Turkiye, Ukraine, United Kingdom, England, Northern Ireland, Scotland, Wales	01/2015 to 12/2015	European Renal Association-European Dialysis and Transplant Association (ERA-EDTA). ERA-EDTA Registry Annual Report 2015. Amsterdam, Netherlands: Department of Medical Informatics, Academic Medical Center (The Netherlands), 2016.
Disease registry	NA	Country	Albania, Austria, Belarus, Belgium, Bosnia and Herzegovina, Bulgaria, Croatia, Cyprus, Czechia, Denmark, Estonia, Finland, France, Georgia, Greece, Iceland, Israel, Italy, Latvia, Lithuania, Macedonia, Norway, Poland, Portugal, Romania, Russian Federation, Serbia, Slovakia, Spain, Sweden, Switzerland, Tunisia, Sfax, Turkiye, Ukraine, United Kingdom, England, Northern Ireland, Scotland, Wales	01/2016 to 12/2016	European Renal Association-European Dialysis and Transplant Association (ERA-EDTA). ERA-EDTA Registry Annual Report 2016. Amsterdam, Netherlands: Department of Medical Informatics, Academic Medical Center (The Netherlands), 2016.

Data type	Secondary data type	Coverage type	Geography	Time period covered	Suggested citation
Disease registry	NA	Country	Albania, Austria, Belarus, Belgium, Bosnia and Herzegovina, Bulgaria, Croatia, Cyprus, Czechia, Denmark, Estonia, Finland, France, Georgia, Greece, Iceland, Israel, Italy, Latvia, Lithuania, North Macedonia, Norway, Poland, Portugal, Romania, Russian Federation, Serbia, Slovakia, Spain, Sweden, Switzerland, Tunisia, Sfax, Turkiye, Ukraine, United Kingdom, England, Northern Ireland, Scotland, Wales	01/2017 to 12/2017	European Renal Association-European Dialysis and Transplant Association (ERA-EDTA). ERA-EDTA Registry Annual Report 2017. Amsterdam, Netherlands: Department of Medical Informatics, Academic Medical Center (The Netherlands), 2019.
Scientific literature	NA	NA	Turkiye	01/1997 to 12/2010	Onat A, Can G, √á√ißek G, Ayhan E, Doƒüan Y, Kaya H. Fasting, non-fasting glucose and HDL dysfunction in risk of pre-diabetes, diabetes, and coronary disease in non-diabetic adults. Acta Diabetol. 2013; 50(4): 519-28.
Scientific literature	NA	NA	Turkiye	01/2007 to 12/2009	Unal B, Sozmen K, Ucku R, Ergor G, Soysal A, Baydur H, Meseri R, Simsek H, Gerceklioglu G, Doganay S, Budak R, Kilic B, Gunay T, Ergor A, Demiral Y, Aslan O, Cimrin D, Akvardar Y, Tuncel P. High prevalence of cardiovascular risk factors in a Western urban Turkish population: a community-based study. Anadolu Kardiyol Derg. 2013; 13(1): 9,Äì17.
Scientific literature	NA	NA	Turkiye	01/2003 to 12/2003	Gokcel A, Ozsahin AK, Sezgin N, Karakose H, Ertorer ME, Akbaba M, Baklaci N, Sengul A, Guvener N. High prevalence of diabetes in Adana, a southern province of Turkey. Diabetes Care. 2003; 26(11): 3031-4.
Scientific literature	NA	Country	Turkiye	01/2001 to 12/2001	Yumuk VD, Hatemi H, Tarakci T, Uyar N, Turan N, Bagriacik N, Ipbuker A. High prevalence of obesity and diabetes mellitus in Konya, a central Anatolian city in Turkey. Diabetes Res Clin Pract. 2005; 70(2): 151-8.
Scientific literature	NA	NA	Turkiye	01/2005 to 12/2006	Dogan N, Toprak D, Demir S. Hypertension prevalence and risk factors among adult population in Afyonkarahisar region: a cross-sectional research. Anadolu Kardiyol Derg. 2012; 12(1): 47,Äì52.
Scientific literature	NA	NA	Turkiye	01/2006 to 12/2006	Toprak O, Cirit M, Yesil M, Bayata S, Tanrisev M, Varol U, Ersoy R, Esi E. Impact of diabetic and pre-diabetic state on development of contrast-induced nephropathy in patients with chronic kidney disease. Nephrol Dial Transplant. 2007; 22(3): 819-26.
Scientific literature	NA	NA	Turkiye	01/2010 to 12/2011	Demirbilek H, √ñzbek MN, Baran RT. Incidence of type 1 diabetes mellitus in Turkish children from the southeastern region of the country: a regional report. J Clin Res Pediatr Endocrinol. 2013; 5(2): 98-103.
Scientific literature	NA	NA	Turkiye	01/2002 to 12/2012	Inal A, Kaplan MA, Kucukoner M, Urakcƒ± Z, kƒ±lƒ±nc F, Isƒ±kdogan A. Is diabetes mellitus a negative prognostic factor for the treatment of advanced non-small-cell lung cancer?. Rev Port Pneumol. 2014; 20(2): 62,Äì8.
Scientific literature	NA	NA	Turkiye	01/1990 to 12/2007	Onat A, Hergenc G, Kucukdurmaz Z, Ugur M, Kaya Z, Can G, Yuksel H. Moderate and heavy alcohol consumption among Turks: long-term impact on mortality and cardiometabolic risk. Turk Kardiyol Dern Ars. 2009; 37(2): 83-90.
Scientific literature	NA	NA	Turkiye	01/1998 to 12/1998	Satman I, Yilmaz T, Seng√ºl A, Salman S, Salman F, Uygur S, Bastar I, T√ºt√ºnc√º Y, Sargin M, Din√ß√ßag N, Karsidag K, Kala√ßa S, Ozcan C, King H. Population-based study of diabetes and risk characteristics in Turkey: results of the Turkish Diabetes Epidemiology Study (TURDEP). Diabetes Care. 2002; 25(9): 1551,Äì6.
Scientific literature	NA	NA	Turkiye	01/2013 to 12/2014	Bayindir Cevik A, Metin Karaaslan M, Kocan S, Pekmezci H, Baydur Sahin S, Kirbas A, Ayaz T. Prevalence and screening for risk factors of type 2 diabetes in Rize, Nourtheast Turkey: findings from a population-based study. Prim Care Diabetes. 2015; 10(1): nan.
Scientific literature	NA	Country	Algeria, Argentina, Bahrain, Bangladesh, China, Egypt, India, Indonesia, Iran (Islamic Republic of), Jordan, Kuwait, Libya, Malaysia, Mexico, Morocco, Pakistan, Philippines, Qatar, Russian Federation, Saudi Arabia, Singapore, Republic of Korea, Taiwan (Province of China), Tunisia, Turkiye, United Arab Emirates, Yemen	01/2009 to 12/2010	Litwak L, Goh SY, Hussein Z, Malek R, Prusty V, Khamseh ME. Prevalence of diabetes complications in people in type 2 diabetes mellitus and its association with baseline characteristics in the multinational A1chieve study . Diabetol Metab Syndr. 2013; 5(1): 57.
Scientific literature	NA	NA	Turkiye	01/1999 to 12/1999	Erem C, Yildiz R, Kavgaci H, Karahan C, Deger O, Can G, Telatar M. Prevalence of diabetes, obesity and hypertension in a Turkish population (Trabzon city). Diabetes Res Clin Pract. 2001; 54(3): 203-8.
Scientific literature	NA	NA	Turkiye	01/2012 to 12/2013	Bayram F, Kocer D, Gundogan K, Kaya A, Demir O, Coskun R, Sabuncu T, Karaman A, Cesur M, Rizzo M, Toth PP, Gedik V. Prevalence of dyslipidemia and associated risk factors in Turkish adults. J Clin Lipidol. 2014; 8(2): 206,Äì16.

Data type	Secondary data type	Coverage type	Geography	Time period covered	Suggested citation
Scientific literature	NA	NA	Turkiye	01/2003 to 12/2005	Gundogan K, Bayram F, Capak M, Tanriverdi F, Karaman A, Ozturk A, Altunbas H, Gokce C, Kalkan A, Yazici C. Prevalence of metabolic syndrome in the Mediterranean region of Turkey: evaluation of hypertension, diabetes mellitus, obesity, and dyslipidemia. Metab Syndr Relat Disord. 2009; 7(5): 427,Äì34.
Scientific literature	NA	NA	Turkiye	01/2000 to 12/2002	Sanisoglu SY, Oktenli C, Hasimi A, Yokusoglu M, Ugurlu M. Prevalence of metabolic syndrome-related disorders in a large adult population in Turkey. BMC Public Health. 2006; 92.
Scientific literature	NA	NA	Turkiye	01/2008 to 12/2010	Erbas T, Ertas M, Yucel A, Keskinaslan A, Senocak M, TURNEP Study Group. Prevalence of peripheral neuropathy and painful peripheral neuropathy in Turkish diabetic patients. J Clin Neurophysiol. 2011; 28(1): 51,Äì5.
Scientific literature	NA	NA	Turkiye	01/2009 to 12/2009	Akesen E, Turan S, G√ºran T, Atay Z, Save D, Bereket A. Prevalence of type 1 diabetes mellitus in 6-18-yr-old school children living in Istanbul, Turkey. Pediatr Diabetes. 2011; 12(6): 567-71.
Scientific literature	NA	NA	Turkiye	01/2002 to 12/2003	Beji NK, Reis N. Risk factors for breast cancer in Turkish women: a hospital-based case-control study. Eur J Cancer Care (Engl). 2007; 16(2): 178-84.
Scientific literature	NA	NA	Turkiye	01/2002 to 12/2003	ReiÃás N, BejiÃá NK. Risk Factors for Ovarian Cancer: Results from a Hospital-Based Case-Control Study. Turkiye Klinikleri J Med Sci. 2010; 30(1): 79,Äì87.
Scientific literature	NA	NA	Turkiye	01/1998 to 12/2003	Maral I, T√ºt√ºnc√º NB, Bakar C, Durukan E, Budakofülu II, Ozkan S, Aycan S, Ayg√ºn R, Bumin MA. The 5-year incidence of type 2 diabetes mellitus in women older than 15 years in Ankara, Turkey: a population-based study. J Investig Med. 2010; 58(6): 796-800.
Scientific literature	NA	Country	Germany, Japan, Netherlands, Norway, Serbia, Republic of Korea, Taiwan (Province of China), Turkiye, United States of America	01/1984 to 12/2012	Zhu L, Cao H, Zhang T, Shen H, Dong W, Wang L, Du J. The effect of diabetes mellitus on lung cancer prognosis: a PRISMA-compliant meta-analysis of cohort studies. Medicine (Baltimore). 2016; 95(17): :e3528.
Scientific literature	NA	NA	Turkiye	01/1990 to 12/1990	Onat A, Hergenc G, Bulur S, Ugur M, Kucukdurmaz Z, Can G. The paradox of high apolipoprotein A-I levels independently predicting incident type-2 diabetes among Turks. Int J Cardiol. 2010; 142(1): 72,Äì9.
Scientific literature	NA	NA	Turkiye	01/1994 to 12/1994	Kelestimur F, Cetin M, Pa≈üaofülu H, Coksevim B, Cetinkaya F, Unl√ºhizarci K, Unal S, K√∂ker AH. The prevalence and identification of risk factors for type 2 diabetes mellitus and impaired glucose tolerance in Kayseri, central Anatolia, Turkey. Acta Diabetol. 1999; 36(1-2): 85-91.
Scientific literature	NA	NA	Turkiye	01/2001 to 12/2002	Soysal A, Demiral Y, Soysal D, U√ßku R, K√∂seoglu M, Aksakoglu G. The prevalence of metabolic syndrome among young adults in Izmir, Turkey. Anadolu Kardiyol Derg. 2005; 5(3): 196-201.
Scientific literature	NA	NA	Turkiye	01/2003 to 12/2005	Tamer A, Yildiz S, Yildiz N, Kanat M, Gunduz H, Tahtaci M, Celebi H. The prevalence of neuropathy and relationship with risk factors in diabetic patients: a single-center experience. Med Princ Pract. 2006; 15(3): 190,Äì4.
Survey	Exam, Individual, Nationally representative	Country	Turkiye	07/2011 to 09/2011	Ministry of Health (Turkey). Turkey Chronic Diseases and Risk Factors Study 2011.
Report	NA	Country	Turkiye	01/1970 to 12/2007	Ministry of Health (Turkey). Turkey Health at a Glance 2007. Ankara, Turkey: Ministry of Health (Turkey), 2008.
Survey	Cross-sectional, Household, Individual, Interview, Urban-rural representative	Country	Turkiye	Apr-08	Turkish Statistical Institute. Turkey Health Interview Survey 2008. Ankara, Turkey: Turkish Statistical Institute.
Survey	Cross-sectional, Household, Individual, Interview, Urban-rural representative	Country	Turkiye	May-10	Turkish Statistical Institute. Turkey Health Interview Survey 2010. Ankara, Turkey: Turkish Statistical Institute.
Report	NA	Country	Turkiye	01/2009 to 12/2009	Ministry of Health of Turkey, Turkish Statistical Institute. Turkey Health Statistics Yearbook 2009. Ankara, Turkey
Report	NA	Country	Turkiye	01/2010 to 12/2010	Ministry of Health of Turkey, Turkish Statistical Institute. Turkey Health Statistics Yearbook 2010. Ankara, Turkey
Report	NA	Country	Turkiye	01/1990 to 12/2011	Ministry of Health (Turkey). Turkey Health Statistics Yearbook 2011. Ankara, Turkey: Ministry of Health (Turkey), 2012.
Report	Discharge, Epi surveillance, Inpatient, Outpatient	Country	Turkiye	01/2002 to 12/2014	Ministry of Health (Turkey). Turkey Health Statistics Yearbook 2014. Ankara, Turkey: General Directorate for Health Research (SAGEM) (Turkey), 2015.
Report	Discharge, Epi surveillance, Inpatient, Outpatient	Country	Turkiye	01/2002 to 12/2015	Ministry of Health (Turkey). Turkey Health Statistics Yearbook 2015. Ankara, Turkey: General Directorate for Health Research (SAGEM) (Turkey), 2015.
Report	Discharge, Epi surveillance, Inpatient, Outpatient	Country	Turkiye	01/2002 to 12/2016	Ministry of Health (Turkey). Turkey Health Statistics Yearbook 2016. Ankara, Turkey: General Directorate for Health Research (SAGEM) (Turkey), 2017.
Report	Discharge, Epi surveillance, Inpatient, Outpatient	Country	Turkiye	01/2002 to 12/2017	Ministry of Health (Turkey). Turkey Health Statistics Yearbook 2017. Ankara, Turkey: Ministry of Health (Turkey), 2018.

Data type	Secondary data type	Coverage type	Geography	Time period covered	Suggested citation
Survey	Cross-sectional, Household, Mortuary, Nationally representative, Urban-rural representative, Verbal autopsy	Country	Turkiye	06/2005 to 05/2006	BNB Consulting (Turkey), ICON-INSTITUTE Consulting Group, Institute of Population Studies, Hacettepe University, Ministry of Health (Turkey). Turkey National Maternal Mortality Study 2005-2006.
Survey	Cross-sectional, Household, Individual, Interview, Nationally representative, Urban-rural representative	Country	Turkiye	06/2003 to 11/2003	Omega Contract Research Organization, Turkish Society of Hypertension and Renal Diseases. Turkey Prevalence, Awareness, Treatment and Control of Hypertension Study 2003. Ankara, Turkey: Turkish Society of Hypertension and Renal Diseases.
Survey	Longitudinal	Country	Turkiye	Jan-02	Hamilton Health Sciences, McMaster University (Canada), Population Health Research Institute (PHRI). Turkey Prospective Urban and Rural Epidemiological Study.
Disease registry	NA	Country	Turkiye	01/2014 to 12/2014	Turkey Registry of Nephrology, Dialysis and Transplantation¬†Renal Replacement Therapy Data 2014 - ERA-EDTA
Disease registry	NA	Country	Turkiye	01/2015 to 12/2015	Turkey Registry of Nephrology, Dialysis and Transplantation Renal Replacement Therapy Data 2015 - ERA-EDTA
Disease registry	NA	Country	Turkiye	01/2016 to 12/2016	Turkey Registry of Nephrology, Dialysis and Transplantation Renal Replacement Therapy Data 2016 - ERA-EDTA.
Disease registry	NA	Country	Turkiye	01/2017 to 12/2017	Turkey Registry of Nephrology, Dialysis and Transplantation Renal Replacement Therapy Data 2017 - ERA-EDTA.
Survey	Interview, Nationally representative, Urban-rural representative	Country	Turkiye	01/2007 to 04/2007	Omega Contract Research Organization, Turkish Society of Hypertension and Renal Diseases. Turkey Salt Consumption and Blood Pressure Study 2007.
Report	NA	Country	Turkiye	01/1988 to 12/2010	Turkish Statistical Institute. Turkey Statistical Yearbook 2010. Ankara, Turkey: Turkish Statistical Institute, 2011.
Report	NA	Country	Turkiye	01/1998 to 12/2011	Turkish Statistical Institute. Turkey Statistical Yearbook 2011. Ankara, Turkey: Turkish Statistical Institute, 2012.
Survey	Cross-sectional, Exam, Household, Individual, Interview, Nationally representative	Country	Turkiye	04/2017 to 09/2017	Ministry of Health (Turkey), World Health Organization (WHO). Turkey STEPS Noncommunicable Disease Risk Factors Survey 2017.
Survey	Cross-sectional, GPS coordinates (GIS), Household, Individual, Interview, Nationally representative, Verbal autopsy	Country	Turkiye	02/2003 to 05/2003	World Health Organization (WHO). Turkey World Health Survey 2003. Geneva, Switzerland: World Health Organization (WHO), 2005.
Disease registry	NA	Country	Turkiye	01/2010 to 12/2010	Turkish Society of Nephrology (TSN) Registry Renal Replacement Therapy Data 2010 - ERA-EDTA
Disease registry	NA	Country	Turkiye	01/2011 to 12/2011	Turkish Society of Nephrology (TSN) Registry Renal Replacement Therapy Data 2011 - ERA-EDTA
Scientific literature	NA	NA	Turkiye	01/2010 to 12/2010	Satman I, Omer B, Tutuncu Y, Kalaca S, Gedik S, Dinccag N, Karsidag K, Genc S, Telci A, Canbaz B, Turker F, Yilmaz T, Cakir B, Tuomilehto J, TURDEP-II Study Group. Twelve-year trends in the prevalence and risk factors of diabetes and prediabetes in Turkish adults. Eur J Epidemiol. 2013; 28(2): 169-80.
Scientific literature	NA	NA	Turkiye	01/1955 to 12/1980	Bagria√ßik N, Ipb√ºker A. [Incidence of juvenile diabetes and its characteristics in Turkey]. Journ Annu Diabetol Hotel Dieu. 1981; 97-100.
Scientific literature	NA	NA	United Arab Emirates	01/2009 to 12/2010	Hajat C, Harrison O, Al Siksek Z. Diagnostic testing for diabetes using HbA(1c) in the Abu Dhabi population: Weqaya: the Abu Dhabi cardiovascular screening program. Diabetes Care. 2011; 34(11): 2400,Äì2.
Scientific literature	NA	NA	United Arab Emirates	01/1999 to 12/2000	Malik M, Bakir A, Saab BA, Roglic G, King H. Glucose intolerance and associated factors in the multi-ethnic population of the United Arab Emirates results of a national survey. Diabetes Res Clin Pract. 2005; 69(2): 188-95.
Scientific literature	NA	NA	United Arab Emirates	01/2012 to 12/2013	Hamoudi R, Saheb Sharif-Askari N, Saheb Sharif-Askari F, Abusnana S, Aljaibeji H, Taneera J, Sulaiman N. Prediabetes and diabetes prevalence and risk factors comparison between ethnic groups in the United Arab Emirates. Sci Rep. 2019; 9(1): 17437.

Data type	Secondary data type	Coverage type	Geography	Time period covered	Suggested citation
Scientific literature	NA	Country	Algeria, Argentina, Bahrain, Bangladesh, China, Egypt, India, Indonesia, Iran (Islamic Republic of), Jordan, Kuwait, Libya, Malaysia, Mexico, Morocco, Pakistan, Philippines, Qatar, Russian Federation, Saudi Arabia, Singapore, Republic of Korea, Taiwan (Province of China), Tunisia, Turkiye, United Arab Emirates, Yemen	01/2009 to 12/2010	Litwak L, Goh SY, Hussein Z, Malek R, Prusty V, Khamseh ME. Prevalence of diabetes complications in people in type 2 diabetes mellitus and its association with baseline characteristics in the multinational A1chieve study . Diabetol Metab Syndr. 2013; 5(1): 57.
Scientific literature	NA	NA	United Arab Emirates	01/2006 to 12/2006	Saadi H, Carruthers SG, Nagelkerke N, Al-Maskari F, Afandi B, Reed R, Lukic M, Nicholls MG, Kazam E, Algawi K, Al-Kaabi J, Leduc C, Sabri S, El-Sadig M, Elkhumaidi S, Agarwal M, Benedict S. Prevalence of diabetes mellitus and its complications in a population-based sample in Al Ain, United Arab Emirates. Diabetes Res Clin Pract. 2007; 78(3): 369-77.
Scientific literature	NA	NA	United Arab Emirates	01/2003 to 12/2004	Al-Maskari F,¬†El-Sadig M. Prevalence of diabetic retinopathy in the¬†United Arab Emirates: a cross-sectional survey. BMC Ophthalmol. 2007; 7(1): 11.
Scientific literature	NA	NA	United Arab Emirates	01/2003 to 12/2004	Al-Maskari F, El-Sadig M. Prevalence of risk factors for diabetic foot complications. BMC Fam Pract. 2007; 8(1): 59.
Report	Epi surveillance, Inpatient, Nationally representative, Outpatient	Subnational	United Arab Emirates, Ab≈´ ZÃßaby	01/1985 to 12/2010	Abu Dhabi General Authority for Health Services. United Arab Emirates - Abu Dhabi Health Statistics 2010. Abu Dhabi, United Arab Emirates: Abu Dhabi General Authority for Health Services, 2011.
Report	Epi surveillance, Inpatient, Nationally representative, Outpatient	Subnational	United Arab Emirates, Ab≈´ ZÃßaby	01/1985 to 12/2011	Abu Dhabi General Authority for Health Services. United Arab Emirates - Abu Dhabi Health Statistics 2011. Abu Dhabi, United Arab Emirates: Abu Dhabi General Authority for Health Services, 2012.
Report	Epi surveillance, Inpatient, Nationally representative, Outpatient	Subnational	United Arab Emirates, Ab≈´ ZÃßaby	01/1985 to 12/2012	Abu Dhabi General Authority for Health Services. United Arab Emirates - Abu Dhabi Health Statistics 2012. Abu Dhabi, United Arab Emirates: Abu Dhabi General Authority for Health Services, 2013.
Report	Discharge, Epi surveillance, Inpatient, Outpatient	Subnational	United Arab Emirates, Dubayy	01/1995 to 12/2005	Dubai Department of Health and Medical Services. United Arab Emirates - Dubai Health Statistical Yearbook 2005. Dubai: Dubai Department of Health and Medical Services.
Report	Discharge, Epi surveillance, Inpatient, Outpatient	Subnational	United Arab Emirates, Dubayy	01/1996 to 12/2006	Dubai Department of Health and Medical Services. United Arab Emirates - Dubai Health Statistical Yearbook 2006. Dubai: Dubai Department of Health and Medical Services.
Report	Discharge, Epi surveillance, Inpatient, Outpatient	Subnational	United Arab Emirates, Dubayy	01/1997 to 12/2007	Dubai Department of Health and Medical Services. United Arab Emirates - Dubai Health Statistical Yearbook 2007. Dubai: Dubai Department of Health and Medical Services.
Survey	Household, Individual, Interview	Country	United Arab Emirates	04/1995 to 06/1995	Health Ministers‚Äô Council for GCC States, Ministry of Health and Prevention (United Arab Emirates). United Arab Emirates Family Health Survey 1995.
Census	De jure, Household, Nationally representative, Subnationally representative	Country	United Arab Emirates	Dec-05	Ministry of Economy (United Arab Emirates). United Arab Emirates Population, Housing, and Establishments Census 2005.
Survey	Longitudinal	Country	United Arab Emirates	Jan-02	Hamilton Health Sciences, McMaster University (Canada), Population Health Research Institute (PHRI). United Arab Emirates Prospective Urban and Rural Epidemiological Study.
Survey	Cross-sectional, GPS coordinates (GIS), Household, Individual, Interview, Nationally representative, Verbal autopsy	Country	United Arab Emirates	05/2003 to 12/2003	World Health Organization (WHO). United Arab Emirates World Health Survey 2003. Geneva, Switzerland: World Health Organization (WHO), 2005.
Scientific literature	NA	NA	Yemen	01/2004 to 12/2004	Bamashmus MA, Gunaid AA, Khandekar RB. Diabetic retinopathy, visual impairment and ocular status among patients with diabetes mellitus in Yemen: a hospital-based study. Indian J Ophthalmol. 2009; 57(4): 293-8.
Scientific literature	NA	NA	Yemen	01/2008 to 12/2008	Modesti PA, Bamoshmoosh M, Rapi S, Massetti L, Al-Hidabi D, Al Goshae H. Epidemiology of hypertension in Yemen: effects of urbanization and geographical area. Hypertens Res. 2013; 36(8): 711‚Äì7.

Data type	Secondary data type	Coverage type	Geography	Time period covered	Suggested citation
Scientific literature	NA	Country	Algeria, Argentina, Bahrain, Bangladesh, China, Egypt, India, Indonesia, Iran (Islamic Republic of), Jordan, Kuwait, Libya, Malaysia, Mexico, Morocco, Pakistan, Philippines, Qatar, Russian Federation, Saudi Arabia, Singapore, Republic of Korea, Taiwan (Province of China), Tunisia, Turkiye, United Arab Emirates, Yemen	01/2009 to 12/2010	Litwak L, Goh SY, Hussein Z, Malek R, Prusty V, Khamseh ME. Prevalence of diabetes complications in people in type 2 diabetes mellitus and its association with baseline characteristics in the multinational A1chieve study . Diabetol Metab Syndr. 2013; 5(1): 57.
Scientific literature	NA	NA	Yemen	01/2000 to 12/2000	Gunaid AA, Assabri AM. Prevalence of type 2 diabetes and other cardiovascular risk factors in a semirural area in Yemen. East Mediterr Health J. 2008; 14(1): 42-56.
Scientific literature	NA	NA	Sweden, Yemen, 'Adan	01/2001 to 12/2005	Lindblad U, Ek J, Eckner J, Larsson CA, Shan G, Rastam L. Prevalence, awareness, treatment, and control of hypertension: rule of thirds in the Skaraborg project. Scand J Prim Health Care. 2012; 30(2): 88‚Äì94.
Scientific literature	NA	NA	Yemen	01/2007 to 12/2008	Modesti PA, Bamoshmoosh M, Rapi S, Massetti L, Bianchi S, Al-Hidabi D, Al Goshae H. Relationship between hypertension, diabetes and proteinuria in rural and urban households in Yemen. J Hum Hypertens. 2013; 27(9): 572-9.
Scientific literature	NA	NA	Yemen	01/2016 to 12/2016	Dahnan M, Assabri AM, Khader YS. Risk Factors for End-Stage Renal Failure Among Patients on Hemodialysis in Aljomhory Hospital, Sa'adah Governorate, Yemen: Hospital-Based Case-Control Study. JMIR Public Health Surveill. 2019; 5(3): e14215.
Scientific literature	NA	NA	Yemen	01/2000 to 12/2000	Al-Habori M, Al-Mamari M, Al-Meeri A. Type II diabetes mellitus and impaired glucose tolerance in Yemen: prevalence, associated metabolic changes and risk factors. Diabetes Res Clin Pract. 2004; 65(3): 275-81.
Report	Discharge, Epi surveillance, Inpatient, Outpatient, Subnationally representative	Country	Yemen	01/2012 to 12/2012	Ministry of Public Health and Population (Yemen). Yemen Annual Statistical Health Report 2012. Sana'a, Yemen: Ministry of Public Health and Population (Yemen).
Report	Discharge, Epi surveillance, Inpatient, Nationally representative, Outpatient, Urban-rural representative	Country	Yemen	01/2013 to 12/2013	Ministry of Public Health and Population (Yemen). Yemen Annual Statistical Health Report 2013. Sana'a, Yemen: Ministry of Public Health and Population (Yemen).
Report	Discharge, Epi surveillance, Inpatient, Nationally representative, Outpatient, Urban-rural representative	Country	Yemen	01/2014 to 12/2014	Ministry of Public Health and Population (Yemen). Yemen Annual Statistical Health Report 2014.
Survey	Cross-sectional, GPS coordinates (GIS), Household, Individual, Interview, Nationally representative, Subnationally representative, Urban-rural representative, Verbal autopsy	Country	Yemen	09/2013 to 11/2013	Central Statistical Organization (Yemen), ICF International, Ministry of Public Health and Population (Yemen). Yemen Demographic and Health Survey 2013. Fairfax, United States of America: ICF International.
Survey	Household, Individual, Interview, Urban-rural representative, Verbal autopsy	Country	Yemen	01/2003 to 03/2003	Central Statistical Organization (Yemen), League of Arab States, Ministry of Public Health and Population (Yemen), Pan Arab Project for Family Health (PAPFAM). Yemen Family Health Survey 2003.
