# Supplementary material for: Burden of type 2 diabetes mellitus and its risk factors in North Africa and the Middle East, 1990–2019: findings from the Global Burden of Disease study 2019
Source: BMC Public Health. 2024 Jan 5;24:98. doi: 10.1186/s12889-023-16540-8 (PMC10768242; doi:10.1186/s12889-023-16540-8)
Supplement: Supplementary file 2 — Additional file 2: Supplementary Table 2. T2DM burden by all ages (number and rate) and age-standardized rate in 1990 and 2019 with percent changes between 1990 and 2019 in the region countries. [file 12889_2023_16540_MOESM2_ESM.docx]

| **Country** | **Measure** | **Age (Metric)** | **Both** | | | **Female** | | | **Male** | | |
| --- | --- | --- | --- | --- | --- | --- | --- | --- | --- | --- | --- |
|  |  |  | **Year** | | **% Change (1990 to 2019)** | **Year** | | **% Change (1990 to 2019)** | **Year** | | **% Change (1990 to 2019)** |
|  |  |  | **1990** | **2019** |  | **1990** | **2019** |  | **1990** | **2019** |  |
| **Afghanistan** | **Incidence** | All ages  (number) | 18398 (16506 to 20384) | 96531 (85595 to 109548) | 424.7 (386.2 to 460) | 10293 (9185 to 11453) | 53457 (47591 to 60798) | 419.4 (383.4 to 459.7) | 8105 (7217 to 9085) | 43074 (37717 to 49029) | 431.5 (376.8 to 483.5) |
|  |  | All ages  (rate per 100,000) | 161.1 (144.5 to 178.5) | 252.2 (223.6 to 286.2) | 56.5 (45 to 67.1) | 178.4 (159.2 to 198.5) | 286.8 (255.3 to 326.2) | 60.8 (49.6 to 73.3) | 143.5 (127.8 to 160.8) | 219.3 (192.1 to 249.7) | 52.9 (37.1 to 67.8) |
|  |  | Age-standardized (rate per 100,000) | 226.9 (204.8 to 251.8) | 412 (371.7 to 458.7) | 81.6 (73.3 to 89.4) | 245.1 (219.5 to 271.8) | 451.7 (404.1 to 502.3) | 84.3 (74.9 to 94.5) | 206.3 (184.3 to 230.7) | 373.9 (335.5 to 419.3) | 81.2 (69.9 to 93.4) |
|  | **Prevalence** | All ages  (number) | 330512 (295972 to 368753) | 1389719 (1229482 to 1569720) | 320.5 (296.3 to 345.2) | 181625 (161649 to 203773) | 800028 (712003 to 908696) | 340.5 (311.6 to 370) | 148887 (131720 to 167951) | 589691 (517260 to 673182) | 296.1 (263.1 to 329) |
|  |  | All ages  (rate per 100,000) | 2894.4 (2592 to 3229.3) | 3630.6 (3212 to 4100.9) | 25.4 (18.2 to 32.8) | 3147.5 (2801.3 to 3531.3) | 4292 (3819.8 to 4875) | 36.4 (27.4 to 45.5) | 2635.9 (2332 to 2973.4) | 3002.9 (2634 to 3428) | 13.9 (4.4 to 23.4) |
|  |  | Age-standardized (rate per 100,000) | 4458.7 (4001.2 to 4972.7) | 8537.4 (7658.3 to 9562.5) | 91.5 (82.3 to 100.1) | 4893.3 (4371.4 to 5478.2) | 9499.6 (8456.3 to 10688) | 94.1 (83 to 105.4) | 3991.1 (3544.9 to 4497.2) | 7554.6 (6722.6 to 8503.1) | 89.3 (76.5 to 102) |
|  | **Deaths** | All ages  (number) | 1855 (1206 to 2762) | 4331 (2506 to 6392) | 133.5 (65 to 208.6) | 1225 (691 to 1943) | 3268 (1661 to 5108) | 166.8 (81.7 to 269.9) | 630 (437 to 909) | 1063 (730 to 1525) | 68.7 (20.6 to 125.1) |
|  |  | All ages  (rate per 100,000) | 16.2 (10.6 to 24.2) | 11.3 (6.5 to 16.7) | -30.4 (-50.8 to -7.9) | 21.2 (12 to 33.7) | 17.5 (8.9 to 27.4) | -17.4 (-43.7 to 14.5) | 11.2 (7.7 to 16.1) | 5.4 (3.7 to 7.8) | -51.5 (-65.3 to -35.2) |
|  |  | Age-standardized (rate per 100,000) | 27.8 (18.5 to 40.5) | 39.9 (23.4 to 58) | 43.4 (3.7 to 85.1) | 38.2 (22.2 to 60.6) | 57.1 (28.9 to 88.8) | 49.6 (5.2 to 102) | 18.3 (13.1 to 26) | 21.1 (14.9 to 29.4) | 15.2 (-15.1 to 50.3) |
|  | **DALYs** | All ages  (number) | 73967 (52412 to 102293) | 222032 (160451 to 295439) | 200.2 (137.9 to 262.6) | 46709 (30450 to 70318) | 150540 (99017 to 208491) | 222.3 (147.7 to 303.7) | 27258 (20417 to 36764) | 71492 (53409 to 94871) | 162.3 (112.6 to 212.3) |
|  |  | All ages  (rate per 100,000) | 647.8 (459 to 895.8) | 580.1 (419.2 to 771.8) | -10.5 (-29 to 8.2) | 809.4 (527.7 to 1218.6) | 807.6 (531.2 to 1118.5) | -0.2 (-23.3 to 25) | 482.6 (361.5 to 650.9) | 364.1 (272 to 483.1) | -24.6 (-38.9 to -10.2) |
|  |  | Age-standardized (rate per 100,000) | 997.1 (713.4 to 1373.5) | 1567.9 (1136.3 to 2073) | 57.3 (26.8 to 88.1) | 1280.2 (841.3 to 1907.2) | 2042.2 (1331.7 to 2841.2) | 59.5 (24.2 to 97.1) | 722.5 (547.5 to 971.8) | 1053.4 (805.1 to 1376.3) | 45.8 (20.8 to 69.8) |
|  | **YLLs** | All ages  (number) | 47583 (29814 to 71520) | 118561 (67570 to 180593) | 149.2 (71.2 to 240.3) | 32399 (17645 to 53458) | 90517 (42873 to 143508) | 179.4 (86.6 to 295.7) | 15184 (10214 to 22303) | 28044 (18576 to 41398) | 84.7 (27.8 to 155.6) |
|  |  | All ages  (rate per 100,000) | 416.7 (261.1 to 626.3) | 309.7 (176.5 to 471.8) | -25.7 (-48.9 to 1.5) | 561.5 (305.8 to 926.4) | 485.6 (230 to 769.9) | -13.5 (-42.2 to 22.5) | 268.8 (180.8 to 394.9) | 142.8 (94.6 to 210.8) | -46.9 (-63.3 to -26.5) |
|  |  | Age-standardized (rate per 100,000) | 638 (406.5 to 955.7) | 887.5 (512.1 to 1308.9) | 39.1 (-1.5 to 86.4) | 888.5 (495.4 to 1430.3) | 1286.8 (651.1 to 2001.9) | 44.8 (-1.7 to 99.4) | 398.8 (273.1 to 580.7) | 449.9 (306.6 to 641.5) | 12.8 (-20.1 to 51.7) |
|  | **YLDs** | All ages  (number) | 26384 (17449 to 37810) | 103471 (68433 to 146039) | 292.2 (262.9 to 319) | 14310 (9377 to 20737) | 60023 (40020 to 85211) | 319.4 (290.1 to 352.1) | 12074 (7905 to 17270) | 43448 (28681 to 61314) | 259.9 (220.9 to 296.4) |
|  |  | All ages  (rate per 100,000) | 231.1 (152.8 to 331.1) | 270.3 (178.8 to 381.5) | 17 (8.3 to 25) | 248 (162.5 to 359.4) | 322 (214.7 to 457.1) | 29.9 (20.8 to 39.9) | 213.8 (140 to 305.8) | 221.2 (146.1 to 312.2) | 3.5 (-7.7 to 14) |
|  |  | Age-standardized (rate per 100,000) | 359 (237.8 to 510.3) | 680.4 (457.4 to 959) | 89.5 (80.7 to 98.9) | 391.7 (256.6 to 557.1) | 755.4 (498.1 to 1053.6) | 92.9 (81.5 to 105.4) | 323.7 (212.2 to 460.6) | 603.5 (399.8 to 845.5) | 86.4 (73.9 to 99.4) |
| **Algeria** | **Incidence** | All ages  (number) | 33340 (30192 to 36817) | 159510 (144388 to 177237) | 378.4 (350 to 409.6) | 17146 (15289 to 18945) | 82119 (73978 to 91388) | 378.9 (343.6 to 418.7) | 16193 (14594 to 18021) | 77391 (69249 to 86928) | 377.9 (344.5 to 417.8) |
|  |  | All ages  (rate per 100,000) | 131.9 (119.4 to 145.6) | 381.2 (345 to 423.5) | 189.1 (171.9 to 207.9) | 137.2 (122.3 to 151.6) | 397.7 (358.3 to 442.6) | 189.9 (168.5 to 214) | 126.7 (114.2 to 141) | 365 (326.6 to 410) | 188.2 (168.1 to 212.2) |
|  |  | Age-standardized (rate per 100,000) | 211.9 (191.4 to 234.1) | 386.4 (350.3 to 428.8) | 82.4 (72 to 92.9) | 215.6 (193.5 to 238) | 400.7 (362.8 to 442.7) | 85.8 (72.7 to 100.1) | 208.4 (188.4 to 232.8) | 372.3 (333.5 to 415.4) | 78.6 (65.9 to 93.5) |
|  | **Prevalence** | All ages  (number) | 546325 (488835 to 610518) | 2810041 (2505864 to 3150617) | 414.4 (384.9 to 445.7) | 282243 (249462 to 317135) | 1440020 (1279215 to 1620998) | 410.2 (372.3 to 453.9) | 264082 (234334 to 297903) | 1370020 (1215769 to 1550433) | 418.8 (383.9 to 458.7) |
|  |  | All ages  (rate per 100,000) | 2160.9 (1933.5 to 2414.8) | 6715 (5988.1 to 7528.8) | 210.8 (193 to 229.7) | 2258.3 (1996 to 2537.5) | 6974.8 (6195.9 to 7851.4) | 208.8 (185.9 to 235.3) | 2065.6 (1832.9 to 2330.1) | 6462 (5734.4 to 7312.9) | 212.8 (191.8 to 236.9) |
|  |  | Age-standardized (rate per 100,000) | 4048 (3641.2 to 4517.6) | 7675.1 (6860.5 to 8599.1) | 89.6 (78.4 to 101.2) | 4113.3 (3649.9 to 4587.3) | 7963.4 (7102.7 to 8915.8) | 93.6 (78.9 to 109.5) | 3983.3 (3538.9 to 4479.8) | 7396.3 (6590.2 to 8334.6) | 85.7 (72.9 to 100.2) |
|  | **Deaths** | All ages  (number) | 1531 (1099 to 2117) | 4995 (3849 to 6447) | 226.3 (141.8 to 341.8) | 877 (577 to 1321) | 2904 (2159 to 4089) | 231.2 (137.6 to 386.5) | 654 (481 to 902) | 2091 (1530 to 2710) | 219.7 (120.4 to 357.6) |
|  |  | All ages  (rate per 100,000) | 6.1 (4.3 to 8.4) | 11.9 (9.2 to 15.4) | 97.1 (46.1 to 166.9) | 7 (4.6 to 10.6) | 14.1 (10.5 to 19.8) | 100.5 (43.8 to 194.5) | 5.1 (3.8 to 7.1) | 9.9 (7.2 to 12.8) | 92.8 (32.9 to 175.9) |
|  |  | Age-standardized (rate per 100,000) | 17 (12.6 to 23.5) | 18.2 (13.9 to 23.4) | 6.9 (-19 to 41.8) | 20.2 (13.3 to 31.1) | 22.9 (16.9 to 33.4) | 13.3 (-18 to 62.5) | 14.4 (10.6 to 19.7) | 14.6 (10.8 to 18.6) | 1.1 (-28 to 41.5) |
|  | **DALYs** | All ages  (number) | 79090 (58771 to 102412) | 329574 (249708 to 430895) | 316.7 (256.3 to 379.9) | 43428 (32178 to 57657) | 176919 (134469 to 233003) | 307.4 (236.4 to 383.9) | 35663 (26576 to 46117) | 152655 (112592 to 199621) | 328.1 (262.1 to 392.2) |
|  |  | All ages  (rate per 100,000) | 312.8 (232.5 to 405.1) | 787.6 (596.7 to 1029.7) | 151.8 (115.3 to 189.9) | 347.5 (257.5 to 461.3) | 856.9 (651.3 to 1128.6) | 146.6 (103.6 to 192.9) | 278.9 (207.9 to 360.7) | 720 (531.1 to 941.6) | 158.1 (118.4 to 196.8) |
|  |  | Age-standardized (rate per 100,000) | 641.4 (485.4 to 828.7) | 951 (727.9 to 1234.6) | 48.3 (26.4 to 71.6) | 700.4 (520.5 to 932.9) | 1046 (799.2 to 1365.4) | 49.3 (23.5 to 78.8) | 586.7 (438.5 to 753.8) | 866 (648.7 to 1123.7) | 47.6 (24 to 71) |
|  | **YLLs** | All ages  (number) | 34978 (24898 to 48532) | 103033 (79278 to 133177) | 194.6 (114.8 to 308.7) | 20519 (13417 to 30468) | 60207 (44811 to 81518) | 193.4 (106 to 328.9) | 14459 (10443 to 20146) | 42826 (30726 to 55960) | 196.2 (99.8 to 327.3) |
|  |  | All ages  (rate per 100,000) | 138.3 (98.5 to 192) | 246.2 (189.4 to 318.2) | 78 (29.8 to 146.9) | 164.2 (107.4 to 243.8) | 291.6 (217 to 394.8) | 77.6 (24.7 to 159.6) | 113.1 (81.7 to 157.6) | 202 (144.9 to 263.9) | 78.6 (20.5 to 157.7) |
|  |  | Age-standardized (rate per 100,000) | 305.1 (220.8 to 420.4) | 318.9 (244.8 to 411.2) | 4.5 (-22.2 to 42.4) | 358.1 (235.7 to 535) | 387.7 (288.6 to 545.5) | 8.3 (-21.8 to 55.8) | 256.6 (186.9 to 354.9) | 259.4 (187.6 to 336.7) | 1.1 (-30.7 to 44.2) |
|  | **YLDs** | All ages  (number) | 44113 (29419 to 62957) | 226542 (150489 to 318427) | 413.6 (382.1 to 446.7) | 22909 (15222 to 32663) | 116712 (76835 to 166886) | 409.5 (370 to 454.7) | 21204 (14087 to 30558) | 109830 (72401 to 153460) | 418 (381.4 to 461) |
|  |  | All ages  (rate per 100,000) | 174.5 (116.4 to 249) | 541.4 (359.6 to 760.9) | 210.3 (191.3 to 230.3) | 183.3 (121.8 to 261.4) | 565.3 (372.2 to 808.3) | 208.4 (184.5 to 235.8) | 165.9 (110.2 to 239) | 518 (341.5 to 723.8) | 212.3 (190.3 to 238.3) |
|  |  | Age-standardized (rate per 100,000) | 336.2 (224.8 to 476.3) | 632.1 (420 to 888.8) | 88 (77.1 to 99.8) | 342.3 (228.4 to 484.9) | 658.3 (437.1 to 930.1) | 92.3 (77 to 108.8) | 330.1 (219.2 to 468.4) | 606.6 (399.5 to 846.5) | 83.8 (71 to 99.1) |
| **Bahrain** | **Incidence** | All ages  (number) | 1313 (1192 to 1434) | 14376 (13281 to 15588) | 994.7 (906.8 to 1087.5) | 480 (435 to 527) | 4575 (4257 to 4914) | 852.4 (778.4 to 935.2) | 833 (749 to 919) | 9801 (8921 to 10809) | 1076.8 (963.6 to 1197.6) |
|  |  | All ages  (rate per 100,000) | 258.5 (234.6 to 282.3) | 996.4 (920.6 to 1080.5) | 285.5 (254.5 to 318.2) | 224.7 (203.3 to 246.5) | 838.6 (780.4 to 900.8) | 273.2 (244.2 to 305.6) | 283 (254.4 to 312.2) | 1092.4 (994.4 to 1204.8) | 286 (248.9 to 325.7) |
|  |  | Age-standardized (rate per 100,000) | 442.5 (409.2 to 476.7) | 757.6 (721.3 to 795.9) | 71.2 (58.9 to 83.3) | 410.3 (378.8 to 444.3) | 727.6 (691.8 to 763.3) | 77.3 (64.9 to 90) | 464.5 (426 to 505.8) | 771 (731.8 to 813.2) | 66 (51.8 to 79.1) |
|  | **Prevalence** | All ages  (number) | 16561 (14839 to 18328) | 187048 (169696 to 207117) | 1029.5 (938.2 to 1123.6) | 6379 (5705 to 7110) | 61020 (55616 to 67142) | 856.5 (777.3 to 941.2) | 10181 (9048 to 11335) | 126028 (112076 to 141665) | 1137.9 (1012.3 to 1269.7) |
|  |  | All ages  (rate per 100,000) | 3259.6 (2920.7 to 3607.5) | 12965.2 (11762.5 to 14356.3) | 297.8 (265.6 to 330.9) | 2984.7 (2668.9 to 3326.3) | 11185.7 (10195.1 to 12307.9) | 274.8 (243.7 to 307.9) | 3459.3 (3074.5 to 3851.6) | 14047.2 (12492.2 to 15790.2) | 306.1 (264.9 to 349.3) |
|  |  | Age-standardized (rate per 100,000) | 7546 (6822.4 to 8259.7) | 14234.9 (13261.9 to 15287.2) | 88.6 (73.6 to 103.5) | 6989.2 (6293.4 to 7705.7) | 13458.5 (12533.4 to 14496.7) | 92.6 (77.1 to 108.9) | 8010.3 (7153.2 to 8811.5) | 14673.7 (13498 to 15950.7) | 83.2 (64.5 to 102.2) |
|  | **Deaths** | All ages  (number) | 96 (81 to 113) | 700 (554 to 878) | 632.4 (449.4 to 845.4) | 42 (35 to 54) | 293 (227 to 356) | 594 (329.8 to 814.9) | 53 (43 to 66) | 407 (317 to 527) | 662.8 (470.3 to 934.6) |
|  |  | All ages  (rate per 100,000) | 18.8 (15.9 to 22.2) | 48.5 (38.4 to 60.9) | 157.9 (93.5 to 232.9) | 19.7 (16.2 to 25.1) | 53.6 (41.6 to 65.2) | 171.9 (68.4 to 258.5) | 18.2 (14.7 to 22.5) | 45.4 (35.3 to 58.8) | 150.2 (87.1 to 239.4) |
|  |  | Age-standardized (rate per 100,000) | 77.8 (66.2 to 91) | 127 (102.5 to 154.6) | 63.3 (23 to 103.2) | 73.4 (60.7 to 93.8) | 129.3 (100.8 to 155.6) | 76.2 (8.4 to 126.5) | 82.2 (67.2 to 101.1) | 124.5 (99.6 to 156.5) | 51.4 (17 to 92.3) |
|  | **DALYs** | All ages  (number) | 3654 (3058 to 4311) | 31385 (25064 to 39006) | 758.8 (608.3 to 909.4) | 1525 (1271 to 1868) | 11310 (9075 to 13782) | 641.8 (478.6 to 790.6) | 2130 (1765 to 2565) | 20075 (15846 to 25165) | 842.6 (676.9 to 1026.5) |
|  |  | All ages  (rate per 100,000) | 719.3 (602 to 848.6) | 2175.5 (1737.3 to 2703.7) | 202.4 (149.4 to 255.4) | 713.3 (594.6 to 874.1) | 2073.2 (1663.5 to 2526.5) | 190.7 (126.7 to 248.9) | 723.7 (599.8 to 871.6) | 2237.6 (1766.2 to 2804.9) | 209.2 (154.9 to 269.5) |
|  |  | Age-standardized (rate per 100,000) | 2067.6 (1751.7 to 2415.8) | 3232.5 (2622.4 to 3929.3) | 56.3 (28.4 to 83.8) | 1952.2 (1640.1 to 2383.6) | 3200.8 (2597.5 to 3885.6) | 64 (21.7 to 97.5) | 2175.5 (1807.5 to 2616) | 3246.6 (2649.6 to 3978.1) | 49.2 (23.8 to 79) |
|  | **YLLs** | All ages  (number) | 2329 (1954 to 2756) | 16348 (12725 to 20714) | 601.9 (424.6 to 826.6) | 1001 (823 to 1259) | 6279 (4914 to 7696) | 527.3 (304.1 to 743.8) | 1328 (1075 to 1661) | 10070 (7711 to 13165) | 658.2 (459.5 to 937.7) |
|  |  | All ages  (rate per 100,000) | 458.4 (384.6 to 542.4) | 1133.2 (882 to 1435.8) | 147.2 (84.7 to 226.3) | 468.3 (384.9 to 589.2) | 1151 (900.7 to 1410.8) | 145.8 (58.3 to 230.6) | 451.3 (365.2 to 564.4) | 1122.4 (859.5 to 1467.4) | 148.7 (83.5 to 240.4) |
|  |  | Age-standardized (rate per 100,000) | 1421.3 (1202 to 1671.7) | 2032.3 (1632.4 to 2519.8) | 43 (8.4 to 81.3) | 1350.6 (1109.7 to 1708) | 2052.8 (1594.2 to 2487.8) | 52 (-5.2 to 99.1) | 1490.3 (1210.6 to 1842.8) | 2015.3 (1586 to 2578) | 35.2 (3 to 78.4) |
|  | **YLDs** | All ages  (number) | 1325 (878 to 1842) | 15037 (9807 to 21040) | 1034.7 (941.2 to 1131) | 524 (341 to 738) | 5031 (3283 to 7031) | 860.9 (777.4 to 948.3) | 802 (528 to 1128) | 10006 (6532 to 14079) | 1148.2 (1021.1 to 1287.5) |
|  |  | All ages  (rate per 100,000) | 260.8 (172.9 to 362.6) | 1042.3 (679.7 to 1458.4) | 299.6 (266.6 to 333.5) | 245 (159.8 to 345.3) | 922.3 (601.9 to 1288.9) | 276.5 (243.8 to 310.7) | 272.4 (179.4 to 383.2) | 1115.2 (728 to 1569.2) | 309.4 (267.8 to 355.1) |
|  |  | Age-standardized (rate per 100,000) | 646.2 (428 to 900.2) | 1200.2 (798.7 to 1666) | 85.7 (70.9 to 100.9) | 601.6 (396.7 to 850) | 1148 (751.4 to 1594.7) | 90.8 (75 to 107.4) | 685.2 (455.1 to 966.5) | 1231.3 (817.8 to 1719.7) | 79.7 (61.8 to 98.3) |
| **Egypt** | **Incidence** | All ages  (number) | 53140 (48970 to 57588) | 251707 (229320 to 278450) | 373.7 (344.8 to 406.6) | 30567 (27890 to 33377) | 125299 (114039 to 138188) | 309.9 (279.3 to 341.9) | 22573 (20568 to 24609) | 126408 (113544 to 140844) | 460 (419 to 507.8) |
|  |  | All ages  (rate per 100,000) | 95.4 (87.9 to 103.4) | 254.1 (231.5 to 281.1) | 166.3 (150 to 184.8) | 112.4 (102.6 to 122.7) | 262.6 (239 to 289.7) | 133.6 (116.2 to 151.9) | 79.2 (72.2 to 86.3) | 246.1 (221.1 to 274.2) | 210.8 (188 to 237.3) |
|  |  | Age-standardized (rate per 100,000) | 140.8 (130.4 to 151.7) | 295.2 (268.3 to 325.2) | 109.7 (97.1 to 123.8) | 167.3 (154.2 to 182) | 311.8 (285.3 to 340.8) | 86.4 (72.8 to 100.2) | 114.4 (104.8 to 124.2) | 281.1 (251.4 to 313) | 145.8 (128.3 to 166.7) |
|  | **Prevalence** | All ages  (number) | 775872 (699913 to 860055) | 4082798 (3672577 to 4558285) | 426.2 (390.2 to 466.5) | 441570 (393946 to 491954) | 1928998 (1745603 to 2155496) | 336.8 (301.8 to 375.1) | 334301 (298395 to 373817) | 2153800 (1906357 to 2432362) | 544.3 (491.4 to 604.2) |
|  |  | All ages  (rate per 100,000) | 1393 (1256.6 to 1544.1) | 4121.1 (3707.1 to 4601.1) | 195.8 (175.6 to 218.5) | 1623.9 (1448.8 to 1809.2) | 4043.3 (3658.9 to 4518.1) | 149 (129 to 170.8) | 1172.7 (1046.8 to 1311.3) | 4193.4 (3711.6 to 4735.8) | 257.6 (228.2 to 290.8) |
|  |  | Age-standardized (rate per 100,000) | 2392.2 (2172.6 to 2629.7) | 5657.6 (5105.8 to 6302.3) | 136.5 (119.8 to 154.2) | 2783.9 (2500.7 to 3085.8) | 5823.8 (5293.8 to 6463.2) | 109.2 (92 to 127.3) | 1996.3 (1791.3 to 2208.8) | 5541.8 (4935.3 to 6229.4) | 177.6 (155.4 to 204.1) |
|  | **Deaths** | All ages  (number) | 6010 (5569 to 6529) | 19233 (14457 to 25267) | 220 (140.2 to 319.7) | 3401 (3052 to 3824) | 9725 (7120 to 13699) | 186 (109.8 to 309.2) | 2609 (2277 to 2909) | 9508 (6893 to 12723) | 264.5 (167.1 to 393.3) |
|  |  | All ages  (rate per 100,000) | 10.8 (10 to 11.7) | 19.4 (14.6 to 25.5) | 79.9 (35.1 to 136) | 12.5 (11.2 to 14.1) | 20.4 (14.9 to 28.7) | 63 (19.5 to 133.2) | 9.2 (8 to 10.2) | 18.5 (13.4 to 24.8) | 102.3 (48.3 to 173.8) |
|  |  | Age-standardized (rate per 100,000) | 23.3 (21.4 to 25.4) | 33.7 (26 to 43.8) | 44.8 (10.5 to 89.1) | 26.8 (24.1 to 30.5) | 42 (31.5 to 59.6) | 56.5 (17.4 to 119.5) | 19.8 (17.1 to 22) | 29.2 (21.4 to 38.7) | 47.5 (9.5 to 98.1) |
|  | **DALYs** | All ages  (number) | 219146 (193502 to 248721) | 835076 (661795 to 1022770) | 281.1 (219.5 to 351.1) | 122624 (106580 to 141598) | 406683 (319732 to 519031) | 231.7 (172.2 to 318.5) | 96523 (83487 to 110999) | 428393 (330030 to 527528) | 343.8 (262.5 to 438.3) |
|  |  | All ages  (rate per 100,000) | 393.5 (347.4 to 446.6) | 842.9 (668 to 1032.4) | 114.2 (79.6 to 153.6) | 451 (392 to 520.7) | 852.4 (670.2 to 1087.9) | 89 (55.2 to 138.5) | 338.6 (292.9 to 389.4) | 834.1 (642.6 to 1027.1) | 146.3 (101.2 to 198.8) |
|  |  | Age-standardized (rate per 100,000) | 718.2 (637.2 to 811.6) | 1224.7 (973 to 1492.8) | 70.5 (43.2 to 102.9) | 817.3 (711.5 to 942.8) | 1343.9 (1057.5 to 1709.7) | 64.4 (35.2 to 106) | 618.6 (537.5 to 706.2) | 1146.5 (882.9 to 1410.3) | 85.3 (51.2 to 124.2) |
|  | **YLLs** | All ages  (number) | 152128 (141131 to 163511) | 488495 (364915 to 644701) | 221.1 (139.1 to 324.9) | 83798 (75369 to 93450) | 240191 (172769 to 338612) | 186.6 (109.5 to 312.4) | 68330 (59508 to 76511) | 248304 (179055 to 337546) | 263.4 (163.5 to 397.4) |
|  |  | All ages  (rate per 100,000) | 273.1 (253.4 to 293.6) | 493.1 (368.3 to 650.8) | 80.5 (34.4 to 138.9) | 308.2 (277.2 to 343.7) | 503.5 (362.1 to 709.8) | 63.4 (19.4 to 135.1) | 239.7 (208.8 to 268.4) | 483.4 (348.6 to 657.2) | 101.7 (46.3 to 176) |
|  |  | Age-standardized (rate per 100,000) | 504.6 (467 to 546.4) | 729.9 (547.4 to 956.1) | 44.6 (8.3 to 89.7) | 565.8 (511.4 to 630.5) | 827.3 (609.7 to 1159.6) | 46.2 (8.2 to 107.8) | 443.2 (385 to 493.5) | 668.9 (484 to 897.8) | 50.9 (10.2 to 104.6) |
|  | **YLDs** | All ages  (number) | 67018 (44393 to 93536) | 346581 (225743 to 487042) | 417.1 (378 to 457.4) | 38826 (25590 to 54036) | 166492 (108464 to 235944) | 328.8 (289.8 to 369.5) | 28193 (18334 to 39806) | 180089 (116813 to 253676) | 538.8 (485.3 to 600) |
|  |  | All ages  (rate per 100,000) | 120.3 (79.7 to 167.9) | 349.8 (227.9 to 491.6) | 190.7 (168.7 to 213.4) | 142.8 (94.1 to 198.7) | 349 (227.4 to 494.6) | 144.4 (122.2 to 167.6) | 98.9 (64.3 to 139.6) | 350.6 (227.4 to 493.9) | 254.5 (224.8 to 288.5) |
|  |  | Age-standardized (rate per 100,000) | 213.6 (142.3 to 296.9) | 494.8 (325.8 to 691.9) | 131.7 (114.9 to 149.5) | 251.4 (165.5 to 350.8) | 516.7 (339.6 to 727) | 105.5 (87.6 to 125.1) | 175.4 (116 to 247.7) | 477.7 (312.3 to 667.2) | 172.3 (150.2 to 198.2) |
| **Iran (Islamic Republic of)** | **Incidence** | All ages  (number) | 61449 (55484 to 68016) | 291482 (265077 to 320393) | 374.3 (353.6 to 399.2) | 28876 (25972 to 31974) | 150871 (137027 to 166558) | 422.5 (396.6 to 452) | 32573 (29443 to 36266) | 140611 (127862 to 154214) | 331.7 (310.6 to 355.2) |
|  |  | All ages  (rate per 100,000) | 105 (94.8 to 116.2) | 345.8 (314.5 to 380.1) | 229.4 (215 to 246.7) | 100.9 (90.8 to 111.7) | 363.7 (330.3 to 401.5) | 260.5 (242.6 to 280.8) | 108.9 (98.4 to 121.2) | 328.4 (298.6 to 360.2) | 201.7 (187 to 218.1) |
|  |  | Age-standardized (rate per 100,000) | 170.5 (154.9 to 188.4) | 323.3 (295.7 to 354.4) | 89.6 (82.5 to 97.3) | 166.1 (150 to 183.6) | 338.1 (307.9 to 370.8) | 103.5 (94.6 to 113.7) | 173.8 (158.2 to 192.4) | 308.5 (282.5 to 338.1) | 77.5 (69.9 to 85.8) |
|  | **Prevalence** | All ages  (number) | 974556 (867290 to 1092764) | 5035012 (4514277 to 5574025) | 416.6 (398.7 to 435.8) | 460399 (410050 to 516014) | 2600441 (2322667 to 2892889) | 464.8 (442.5 to 491.5) | 514157 (456505 to 577492) | 2434570 (2173837 to 2690109) | 373.5 (352.7 to 395.9) |
|  |  | All ages  (rate per 100,000) | 1664.8 (1481.5 to 1866.7) | 5972.9 (5355.1 to 6612.3) | 258.8 (246.3 to 272.1) | 1608.8 (1432.9 to 1803.1) | 6269.1 (5599.4 to 6974.1) | 289.7 (274.3 to 308) | 1718.3 (1525.6 to 1930) | 5685.9 (5077 to 6282.7) | 230.9 (216.3 to 246.6) |
|  |  | Age-standardized (rate per 100,000) | 3285.6 (2940.8 to 3652.4) | 6312.9 (5690.2 to 6959.5) | 92.1 (85.4 to 99.6) | 3239.9 (2898.1 to 3595.6) | 6555.2 (5898.8 to 7251.3) | 102.3 (94.3 to 111.6) | 3327 (2979.4 to 3711.1) | 6066.9 (5456.3 to 6663.1) | 82.4 (74.5 to 91.2) |
|  | **Deaths** | All ages  (number) | 2414 (2110 to 2761) | 14191 (12014 to 15320) | 488 (361.6 to 594.8) | 1300 (1045 to 1568) | 7667 (5321 to 8527) | 489.8 (271 to 684.7) | 1114 (945 to 1328) | 6524 (5882 to 7191) | 485.8 (374 to 611.2) |
|  |  | All ages  (rate per 100,000) | 4.1 (3.6 to 4.7) | 16.8 (14.3 to 18.2) | 308.3 (220.5 to 382.5) | 4.5 (3.7 to 5.5) | 18.5 (12.8 to 20.6) | 306.9 (155.9 to 441.4) | 3.7 (3.2 to 4.4) | 15.2 (13.7 to 16.8) | 309.4 (231.2 to 397) |
|  |  | Age-standardized (rate per 100,000) | 12.5 (10.7 to 14.4) | 22 (18.4 to 23.8) | 76.4 (38.3 to 110.8) | 13.8 (10.9 to 16.9) | 24.2 (17.2 to 27) | 75 (10.6 to 140.1) | 10.9 (9.3 to 13.1) | 19.8 (17.8 to 21.9) | 81.5 (46.3 to 117.9) |
|  | **DALYs** | All ages  (number) | 138646 (111692 to 170125) | 716457 (578136 to 882592) | 416.8 (370.9 to 454.1) | 67899 (55243 to 83145) | 372689 (295914 to 461409) | 448.9 (371.7 to 510.2) | 70747 (55886 to 87857) | 343768 (276158 to 422870) | 385.9 (343.3 to 430.1) |
|  |  | All ages  (rate per 100,000) | 236.8 (190.8 to 290.6) | 849.9 (685.8 to 1047) | 258.9 (227 to 284.8) | 237.3 (193 to 290.5) | 898.5 (713.4 to 1112.4) | 278.7 (225.5 to 321) | 236.4 (186.8 to 293.6) | 802.9 (645 to 987.6) | 239.6 (209.8 to 270.5) |
|  |  | Age-standardized (rate per 100,000) | 516.5 (420.7 to 628.2) | 958.1 (776.6 to 1170.2) | 85.5 (67 to 99.8) | 534 (438.4 to 648.4) | 1002.6 (803.2 to 1235) | 87.8 (59.7 to 111.8) | 498.1 (398.9 to 614.4) | 913.8 (740 to 1115.3) | 83.4 (66.1 to 100.3) |
|  | **YLLs** | All ages  (number) | 56108 (49399 to 63966) | 284263 (239197 to 305971) | 406.6 (295.4 to 492.3) | 29108 (23990 to 35291) | 148511 (100831 to 164398) | 410.2 (211.8 to 547.3) | 27000 (22695 to 32368) | 135752 (123486 to 149082) | 402.8 (304.4 to 513) |
|  |  | All ages  (rate per 100,000) | 95.8 (84.4 to 109.3) | 337.2 (283.8 to 363) | 251.8 (174.6 to 311.3) | 101.7 (83.8 to 123.3) | 358 (243.1 to 396.3) | 252 (115.1 to 346.6) | 90.2 (75.8 to 108.2) | 317 (288.4 to 348.2) | 251.4 (182.6 to 328.4) |
|  |  | Age-standardized (rate per 100,000) | 228.4 (199.6 to 261.7) | 403 (334.1 to 433.9) | 76.4 (38.1 to 107.3) | 251.4 (203 to 304.1) | 426.6 (291.6 to 473.4) | 69.7 (3.5 to 124.9) | 204.8 (173.5 to 243.6) | 379.6 (345.6 to 417.2) | 85.3 (49.7 to 124.9) |
|  | **YLDs** | All ages  (number) | 82538 (56405 to 113853) | 432194 (298196 to 587454) | 423.6 (405.2 to 445.3) | 38791 (26470 to 53271) | 224178 (154996 to 306078) | 477.9 (454 to 504.8) | 43747 (29838 to 60496) | 208016 (143536 to 282452) | 375.5 (354.1 to 398.5) |
|  |  | All ages  (rate per 100,000) | 141 (96.4 to 194.5) | 512.7 (353.7 to 696.9) | 263.6 (250.8 to 278.7) | 135.6 (92.5 to 186.1) | 540.4 (373.7 to 737.9) | 298.7 (282.2 to 317.3) | 146.2 (99.7 to 202.2) | 485.8 (335.2 to 659.7) | 232.3 (217.3 to 248.4) |
|  |  | Age-standardized (rate per 100,000) | 288.1 (197.9 to 395.5) | 555.2 (380.8 to 758.7) | 92.7 (85.8 to 100.7) | 282.6 (194.2 to 387) | 576 (397.5 to 788.5) | 103.8 (95.4 to 113.5) | 293.3 (201.9 to 402.9) | 534.2 (366.3 to 726) | 82.1 (74.2 to 91.1) |
| **Iraq** | **Incidence** | All ages  (number) | 30355 (27798 to 33220) | 145024 (133030 to 156843) | 377.8 (343.1 to 414.6) | 15441 (14105 to 16941) | 73237 (66226 to 79847) | 374.3 (334.9 to 418.2) | 14915 (13530 to 16430) | 71786 (64518 to 78772) | 381.3 (335.8 to 427.2) |
|  |  | All ages  (rate per 100,000) | 172.5 (158 to 188.8) | 344.3 (315.8 to 372.4) | 99.6 (85.1 to 115) | 179.1 (163.6 to 196.6) | 356.4 (322.3 to 388.5) | 98.9 (82.4 to 117.3) | 166.1 (150.7 to 183) | 332.8 (299.1 to 365.2) | 100.3 (81.4 to 119.4) |
|  |  | Age-standardized (rate per 100,000) | 286.9 (262.8 to 312.7) | 424.9 (392.4 to 460.2) | 48.1 (37.1 to 59.8) | 292.2 (266.5 to 318.7) | 439.6 (399.9 to 480.4) | 50.5 (36.8 to 64.4) | 281.8 (255.1 to 309.7) | 410 (370.7 to 450) | 45.5 (32.1 to 59.7) |
|  | **Prevalence** | All ages  (number) | 499111 (449622 to 550396) | 2330391 (2127199 to 2547889) | 366.9 (332.5 to 402) | 261565 (235784 to 289534) | 1205352 (1082693 to 1319122) | 360.8 (320.3 to 406.3) | 237547 (212198 to 264001) | 1125039 (1007249 to 1251696) | 373.6 (327.6 to 418.7) |
|  |  | All ages  (rate per 100,000) | 2836.4 (2555.2 to 3127.9) | 5532.8 (5050.4 to 6049.2) | 95.1 (80.7 to 109.7) | 3034.7 (2735.5 to 3359.2) | 5865.4 (5268.5 to 6419) | 93.3 (76.3 to 112.4) | 2646.1 (2363.7 to 2940.7) | 5216 (4669.9 to 5803.2) | 97.1 (78 to 115.9) |
|  |  | Age-standardized (rate per 100,000) | 5595.5 (5041.5 to 6185.8) | 8564.6 (7852.9 to 9298.9) | 53.1 (41.1 to 65.7) | 5777.7 (5199.8 to 6414.7) | 8860.3 (7984.9 to 9709) | 53.4 (38.9 to 69.2) | 5421.2 (4864.2 to 6054.9) | 8259.4 (7399.9 to 9105.9) | 52.4 (37.6 to 67.6) |
|  | **Deaths** | All ages  (number) | 3584 (2884 to 4284) | 9038 (7191 to 10968) | 152.2 (91.8 to 227.2) | 1973 (1532 to 2430) | 4433 (3491 to 5598) | 124.7 (64.2 to 206.1) | 1611 (1260 to 2009) | 4605 (3564 to 5555) | 185.8 (109.6 to 285.8) |
|  |  | All ages  (rate per 100,000) | 20.4 (16.4 to 24.3) | 21.5 (17.1 to 26) | 5.3 (-19.9 to 36.7) | 22.9 (17.8 to 28.2) | 21.6 (17 to 27.2) | -5.8 (-31.1 to 28.4) | 17.9 (14 to 22.4) | 21.3 (16.5 to 25.8) | 18.9 (-12.8 to 60.6) |
|  |  | Age-standardized (rate per 100,000) | 49.2 (39.4 to 58.6) | 45.8 (37.1 to 54.4) | -7 (-28.6 to 19.8) | 52.2 (40.4 to 64.8) | 43.6 (34.5 to 54.2) | -16.6 (-37.6 to 12.9) | 45.9 (35.8 to 57.1) | 47.9 (38.4 to 56.4) | 4.5 (-21.3 to 40.2) |
|  | **DALYs** | All ages  (number) | 128267 (107542 to 151909) | 400206 (319445 to 489469) | 212 (154.1 to 272.6) | 69550 (56546 to 83939) | 200344 (159016 to 247371) | 188.1 (128.1 to 257.5) | 58717 (47631 to 70999) | 199862 (159894 to 243996) | 240.4 (175.2 to 320.9) |
|  |  | All ages  (rate per 100,000) | 728.9 (611.2 to 863.3) | 950.2 (758.4 to 1162.1) | 30.4 (6.1 to 55.7) | 806.9 (656 to 973.9) | 974.9 (773.8 to 1203.7) | 20.8 (-4.3 to 50) | 654.1 (530.6 to 790.9) | 926.6 (741.3 to 1131.2) | 41.7 (14.6 to 75.2) |
|  |  | Age-standardized (rate per 100,000) | 1544 (1292.5 to 1824.7) | 1625 (1314.8 to 1979.3) | 5.2 (-13.9 to 25.2) | 1632.9 (1322.4 to 1978.6) | 1598.6 (1285.3 to 1960.9) | -2.1 (-22.3 to 21) | 1454.1 (1177.3 to 1767) | 1652.2 (1339.4 to 1995.4) | 13.6 (-8 to 39.6) |
|  | **YLLs** | All ages  (number) | 89266 (72683 to 107462) | 218634 (168017 to 274485) | 144.9 (80.1 to 223.4) | 49003 (38288 to 60132) | 105228 (80691 to 136143) | 114.7 (51.7 to 203.9) | 40263 (31270 to 50774) | 113406 (85722 to 142020) | 181.7 (102.3 to 284.8) |
|  |  | All ages  (rate per 100,000) | 507.3 (413 to 610.7) | 519.1 (398.9 to 651.7) | 2.3 (-24.8 to 35.1) | 568.5 (444.2 to 697.6) | 512.1 (392.7 to 662.5) | -9.9 (-36.4 to 27.5) | 448.5 (348.3 to 565.6) | 525.8 (397.4 to 658.4) | 17.2 (-15.8 to 60.2) |
|  |  | Age-standardized (rate per 100,000) | 1089.9 (882.7 to 1305.5) | 928.7 (735 to 1133.2) | -14.8 (-35.9 to 11.9) | 1163.1 (903.8 to 1432.8) | 873.7 (682.6 to 1111) | -24.9 (-45.8 to 4.7) | 1014.9 (786.4 to 1275.5) | 984.8 (755.1 to 1200) | -3 (-29.1 to 30.9) |
|  | **YLDs** | All ages  (number) | 39001 (26067 to 54812) | 181572 (120406 to 254214) | 365.6 (328.8 to 401.7) | 20547 (13641 to 28775) | 95116 (62932 to 134775) | 362.9 (322.5 to 410.9) | 18454 (12423 to 25751) | 86456 (57180 to 120738) | 368.5 (320.6 to 414.1) |
|  |  | All ages  (rate per 100,000) | 221.6 (148.1 to 311.5) | 431.1 (285.9 to 603.6) | 94.5 (79.2 to 109.6) | 238.4 (158.3 to 333.8) | 462.8 (306.2 to 655.8) | 94.2 (77.2 to 114.3) | 205.6 (138.4 to 286.8) | 400.8 (265.1 to 559.8) | 95 (75 to 114) |
|  |  | Age-standardized (rate per 100,000) | 454.1 (304.5 to 632.1) | 696.3 (457.5 to 977.3) | 53.4 (40.9 to 66.1) | 469.8 (314 to 660) | 724.8 (474 to 1021.1) | 54.3 (38.8 to 70.5) | 439.1 (295.4 to 611.8) | 667.4 (441 to 933.5) | 52 (36.4 to 67.8) |
| **Jordan** | **Incidence** | All ages  (number) | 5950 (5503 to 6444) | 38421 (35067 to 41788) | 545.7 (501.5 to 591.7) | 2771 (2548 to 3009) | 15308 (13830 to 16960) | 452.4 (407 to 501.8) | 3179 (2897 to 3491) | 23113 (20759 to 25476) | 627 (564.7 to 696.7) |
|  |  | All ages  (rate per 100,000) | 157.7 (145.9 to 170.8) | 330.2 (301.4 to 359.1) | 109.4 (95 to 124.3) | 154.2 (141.8 to 167.4) | 282.3 (255.1 to 312.8) | 83.2 (68.1 to 99.5) | 160.9 (146.6 to 176.7) | 371.9 (334 to 409.9) | 131.1 (111.3 to 153.2) |
|  |  | Age-standardized (rate per 100,000) | 300.4 (277.9 to 323.3) | 395.6 (363.4 to 428.4) | 31.7 (22.9 to 41.3) | 305.5 (283.1 to 328.6) | 355.2 (322.2 to 390.4) | 16.3 (6.4 to 26.3) | 294 (268.9 to 322.8) | 427.6 (386.7 to 467.4) | 45.4 (32.7 to 59.3) |
|  | **Prevalence** | All ages  (number) | 81163 (74323 to 89072) | 592099 (534664 to 652247) | 629.5 (577.2 to 678.7) | 37191 (33745 to 40784) | 235732 (210891 to 264067) | 533.8 (482.5 to 595.7) | 43972 (39385 to 48870) | 356366 (317000 to 395903) | 710.4 (642.4 to 786.7) |
|  |  | All ages  (rate per 100,000) | 2151.1 (1969.8 to 2360.7) | 5088.2 (4594.6 to 5605.1) | 136.5 (119.6 to 152.5) | 2068.7 (1877.1 to 2268.6) | 4347.7 (3889.5 to 4870.2) | 110.2 (93.1 to 130.7) | 2226 (1993.8 to 2474) | 5734.3 (5100.8 to 6370.5) | 157.6 (136 to 181.8) |
|  |  | Age-standardized (rate per 100,000) | 5243.6 (4824.3 to 5714.5) | 7628.8 (6944.1 to 8318) | 45.5 (35 to 55.9) | 5063.5 (4624.4 to 5533.3) | 6631.4 (5961.5 to 7367.6) | 31 (20 to 43.8) | 5393.9 (4885.6 to 5971.7) | 8493.1 (7575.4 to 9405.4) | 57.5 (43.8 to 72.9) |
|  | **Deaths** | All ages  (number) | 666 (562 to 783) | 2000 (1694 to 2396) | 200.2 (136.6 to 281.6) | 413 (322 to 503) | 946 (754 to 1165) | 128.9 (71 to 211.2) | 253 (201 to 317) | 1054 (821 to 1333) | 316.5 (196.3 to 494.5) |
|  |  | All ages  (rate per 100,000) | 17.7 (14.9 to 20.7) | 17.2 (14.6 to 20.6) | -2.7 (-23.3 to 23.7) | 23 (17.9 to 28) | 17.4 (13.9 to 21.5) | -24.1 (-43.3 to 3.2) | 12.8 (10.2 to 16.1) | 17 (13.2 to 21.5) | 32.4 (-5.8 to 89) |
|  |  | Age-standardized (rate per 100,000) | 63.9 (53.8 to 74.9) | 40.2 (34.1 to 47.5) | -37.1 (-50.1 to -21) | 81.4 (64.1 to 99.2) | 42.3 (34.2 to 51.7) | -48 (-60.4 to -29.2) | 46.3 (36.7 to 57.6) | 38.7 (30.6 to 48.4) | -16.4 (-39.3 to 16.4) |
|  | **DALYs** | All ages  (number) | 21551 (18283 to 25268) | 86617 (70835 to 107107) | 301.9 (236.4 to 375.7) | 12150 (10082 to 14420) | 37391 (29580 to 45881) | 207.7 (148.1 to 284.1) | 9401 (7630 to 11367) | 49227 (38765 to 61875) | 423.6 (317.9 to 547.5) |
|  |  | All ages  (rate per 100,000) | 571.2 (484.6 to 669.7) | 744.3 (608.7 to 920.4) | 30.3 (9.1 to 54.3) | 675.8 (560.8 to 802.1) | 689.6 (545.6 to 846.2) | 2 (-17.7 to 27.3) | 475.9 (386.3 to 575.4) | 792.1 (623.8 to 995.6) | 66.4 (32.8 to 105.8) |
|  |  | Age-standardized (rate per 100,000) | 1624.7 (1380.1 to 1899.1) | 1292.9 (1069.4 to 1569.3) | -20.4 (-33.5 to -5.5) | 1916 (1572.9 to 2281.5) | 1228 (985.1 to 1475.6) | -35.9 (-48.6 to -18.9) | 1332.4 (1099 to 1603.3) | 1349.2 (1081.7 to 1674.9) | 1.3 (-19.1 to 26.6) |
|  | **YLLs** | All ages  (number) | 15433 (13049 to 18153) | 42788 (36063 to 51399) | 177.3 (116 to 256.9) | 9231 (7354 to 11264) | 19076 (15049 to 23941) | 106.6 (55 to 185.3) | 6202 (4876 to 7828) | 23713 (18285 to 30485) | 282.4 (172.4 to 451) |
|  |  | All ages  (rate per 100,000) | 409 (345.9 to 481.1) | 367.7 (309.9 to 441.7) | -10.1 (-30 to 15.7) | 513.5 (409.1 to 626.5) | 351.8 (277.5 to 441.5) | -31.5 (-48.6 to -5.4) | 314 (246.8 to 396.3) | 381.6 (294.2 to 490.5) | 21.5 (-13.4 to 75.1) |
|  |  | Age-standardized (rate per 100,000) | 1211.3 (1022.6 to 1424) | 705.7 (596.2 to 843.2) | -41.7 (-54.5 to -25.4) | 1503 (1179.9 to 1831.7) | 695.7 (555.7 to 858.6) | -53.7 (-65 to -36.7) | 920.4 (727.7 to 1155.9) | 714.3 (555.7 to 904.3) | -22.4 (-44.7 to 10.7) |
|  | **YLDs** | All ages  (number) | 6118 (4085 to 8495) | 43829 (29021 to 61029) | 616.4 (565.2 to 665.9) | 2919 (1936 to 4119) | 18315 (12156 to 25736) | 527.4 (474.8 to 590.3) | 3199 (2111 to 4461) | 25514 (16870 to 35852) | 697.6 (629.3 to 777.3) |
|  |  | All ages  (rate per 100,000) | 162.1 (108.3 to 225.1) | 376.6 (249.4 to 524.5) | 132.3 (115.7 to 148.3) | 162.4 (107.7 to 229.1) | 337.8 (224.2 to 474.6) | 108 (90.6 to 128.9) | 161.9 (106.9 to 225.8) | 410.5 (271.4 to 576.9) | 153.5 (131.8 to 178.8) |
|  |  | Age-standardized (rate per 100,000) | 413.3 (276.4 to 578.5) | 587.2 (387.1 to 817.2) | 42.1 (31.9 to 52.4) | 413.1 (275.5 to 585) | 532.2 (351 to 749.1) | 28.9 (17.8 to 41.7) | 412.1 (276.3 to 571.3) | 634.9 (420.6 to 887.7) | 54.1 (40.8 to 70.3) |
| **Kuwait** | **Incidence** | All ages  (number) | 4253 (3832 to 4698) | 24117 (21492 to 27092) | 467 (420.3 to 512.6) | 1456 (1290 to 1609) | 9549 (8401 to 10845) | 555.8 (490.4 to 623.6) | 2797 (2486 to 3115) | 14568 (12929 to 16474) | 420.8 (368.9 to 478.5) |
|  |  | All ages  (rate per 100,000) | 241.7 (217.8 to 267) | 544.8 (485.5 to 612) | 125.4 (106.8 to 143.5) | 193.8 (171.8 to 214.2) | 464.7 (408.8 to 527.7) | 139.7 (115.8 to 164.5) | 277.5 (246.6 to 309) | 614.3 (545.2 to 694.7) | 121.4 (99.3 to 146) |
|  |  | Age-standardized (rate per 100,000) | 346.9 (317.7 to 378.9) | 495.5 (451.9 to 542.7) | 42.8 (33.1 to 53.1) | 328.2 (297 to 358.2) | 449.8 (402.6 to 498.8) | 37 (25.6 to 49.6) | 356.4 (322.3 to 390.8) | 531.9 (481.9 to 585.5) | 49.2 (36.9 to 62.8) |
|  | **Prevalence** | All ages  (number) | 58101 (51753 to 64787) | 375781 (332354 to 428212) | 546.8 (499.2 to 593.3) | 19784 (17548 to 21891) | 143452 (125346 to 164264) | 625.1 (562.9 to 689.3) | 38317 (33925 to 43360) | 232330 (205903 to 263888) | 506.3 (453.1 to 565.8) |
|  |  | All ages  (rate per 100,000) | 3302.3 (2941.5 to 3682.3) | 8489.2 (7508.2 to 9673.7) | 157.1 (138.2 to 175.5) | 2633.6 (2335.9 to 2914.1) | 6980.3 (6099.2 to 7993) | 165 (142.3 to 188.5) | 3800.5 (3364.9 to 4300.7) | 9796.9 (8682.5 to 11127.6) | 157.8 (135.2 to 183.1) |
|  |  | Age-standardized (rate per 100,000) | 6685.2 (6107.3 to 7339.9) | 10250.1 (9240.2 to 11339.5) | 53.3 (42.1 to 63.9) | 6270.7 (5603.2 to 6879.1) | 9154.6 (8175.1 to 10231.6) | 46 (32.9 to 59.7) | 6941.5 (6271.1 to 7647.2) | 11041.4 (9941.8 to 12281.4) | 59.1 (46.3 to 73.3) |
|  | **Deaths** | All ages  (number) | 130 (117 to 145) | 354 (295 to 423) | 173.7 (133.1 to 222.3) | 63 (55 to 71) | 129 (101 to 161) | 103.7 (64.1 to 150.2) | 66 (59 to 77) | 225 (177 to 284) | 240.7 (171.1 to 319.4) |
|  |  | All ages  (rate per 100,000) | 7.4 (6.7 to 8.2) | 8 (6.7 to 9.6) | 8.8 (-7.3 to 28.1) | 8.4 (7.3 to 9.5) | 6.3 (4.9 to 7.8) | -25.5 (-40 to -8.5) | 6.6 (5.8 to 7.6) | 9.5 (7.4 to 12) | 44.9 (15.3 to 78.3) |
|  |  | Age-standardized (rate per 100,000) | 28.8 (25.4 to 32.2) | 18.5 (15.3 to 22.2) | -35.8 (-45.5 to -24.7) | 33.7 (29 to 38) | 16.7 (13 to 20.8) | -50.4 (-60.1 to -38.8) | 24.5 (21.3 to 29) | 19.6 (15.3 to 24.8) | -20.1 (-36.3 to -2.1) |
|  | **DALYs** | All ages  (number) | 7481 (5929 to 9359) | 35526 (25657 to 47276) | 374.9 (322.6 to 425.9) | 2922 (2377 to 3591) | 13543 (9569 to 18306) | 363.5 (298.5 to 425.9) | 4559 (3533 to 5817) | 21983 (16004 to 29726) | 382.1 (326.3 to 434.1) |
|  |  | All ages  (rate per 100,000) | 425.2 (337 to 531.9) | 802.6 (579.6 to 1068) | 88.7 (68 to 109) | 389 (316.4 to 478.1) | 659 (465.6 to 890.8) | 69.4 (45.7 to 92.3) | 452.2 (350.4 to 577) | 927 (674.9 to 1253.5) | 105 (81.2 to 127.1) |
|  |  | Age-standardized (rate per 100,000) | 1072.4 (877.3 to 1311) | 1136.3 (841.6 to 1480) | 6 (-5.9 to 17.2) | 1136.2 (942.7 to 1382.6) | 1020.5 (737.6 to 1336.3) | -10.2 (-23 to 1.7) | 1025.8 (820.8 to 1284.8) | 1215.7 (917.4 to 1595.5) | 18.5 (4.9 to 31.4) |
|  | **YLLs** | All ages  (number) | 3087 (2823 to 3423) | 7011 (5824 to 8459) | 127.1 (92.3 to 170.6) | 1398 (1227 to 1562) | 2532 (2029 to 3158) | 81.1 (44.4 to 123.8) | 1689 (1501 to 1940) | 4479 (3440 to 5706) | 165.1 (107.7 to 232.5) |
|  |  | All ages  (rate per 100,000) | 175.5 (160.5 to 194.6) | 158.4 (131.6 to 191.1) | -9.7 (-23.5 to 7.6) | 186.1 (163.3 to 207.9) | 123.2 (98.7 to 153.7) | -33.8 (-47.2 to -18.2) | 167.6 (148.9 to 192.4) | 188.9 (145.1 to 240.6) | 12.7 (-11.7 to 41.4) |
|  |  | Age-standardized (rate per 100,000) | 519.5 (470 to 580.3) | 301.3 (250.7 to 364.1) | -42 (-50.7 to -31) | 613.5 (534.6 to 689.8) | 266.5 (211.4 to 331.8) | -56.6 (-65.3 to -46.5) | 453.2 (399.4 to 530.7) | 323.9 (251.5 to 413.8) | -28.5 (-43.8 to -11.3) |
|  | **YLDs** | All ages  (number) | 4394 (2895 to 6229) | 28516 (18812 to 40408) | 548.9 (499.4 to 597.2) | 1524 (1015 to 2133) | 11012 (7160 to 15630) | 622.4 (555.7 to 692.7) | 2870 (1874 to 4078) | 17504 (11529 to 24850) | 509.9 (455.8 to 570.5) |
|  |  | All ages  (rate per 100,000) | 249.8 (164.5 to 354) | 644.2 (425 to 912.9) | 157.9 (138.2 to 177.1) | 202.9 (135.1 to 283.9) | 535.8 (348.4 to 760.6) | 164.1 (139.7 to 189.8) | 284.7 (185.9 to 404.5) | 738.1 (486.1 to 1047.9) | 159.3 (136.3 to 185) |
|  |  | Age-standardized (rate per 100,000) | 552.9 (364.4 to 773.1) | 834.9 (555.8 to 1174.9) | 51 (39.9 to 61.9) | 522.7 (346.2 to 739.1) | 754.1 (504.9 to 1072.2) | 44.3 (31.4 to 58.1) | 572.6 (376.9 to 810.8) | 891.8 (596.1 to 1251.4) | 55.7 (42.8 to 70.8) |
| **Lebanon** | **Incidence** | All ages  (number) | 6092 (5581 to 6712) | 20237 (18365 to 22297) | 232.2 (211.6 to 251.4) | 2917 (2655 to 3217) | 9919 (8969 to 10950) | 240 (216.9 to 265.1) | 3175 (2874 to 3536) | 10318 (9215 to 11457) | 225 (198.8 to 253) |
|  |  | All ages  (rate per 100,000) | 186 (170.4 to 204.9) | 390.9 (354.7 to 430.7) | 110.1 (97.1 to 122.3) | 180 (163.8 to 198.5) | 377.2 (341.1 to 416.4) | 109.6 (95.3 to 125) | 191.9 (173.7 to 213.7) | 405 (361.7 to 449.7) | 111 (94 to 129.2) |
|  |  | Age-standardized (rate per 100,000) | 231.3 (211.8 to 255.2) | 383.5 (347.7 to 424.1) | 65.8 (56.2 to 74.9) | 218.7 (199.3 to 240.5) | 350 (316.1 to 387.2) | 60 (49 to 71.8) | 243.2 (220.9 to 270.3) | 423.3 (377.5 to 472.9) | 74 (60.7 to 88.8) |
|  | **Prevalence** | All ages  (number) | 106905 (96886 to 118263) | 401584 (360406 to 448278) | 275.6 (252.7 to 298.4) | 51887 (46764 to 57672) | 200634 (179206 to 224073) | 286.7 (259.5 to 316.6) | 55019 (49328 to 61466) | 200950 (179078 to 225835) | 265.2 (236 to 296) |
|  |  | All ages  (rate per 100,000) | 3264.2 (2958.3 to 3611) | 7757 (6961.6 to 8658.9) | 137.6 (123.1 to 152) | 3201.6 (2885.5 to 3558.6) | 7630.3 (6815.4 to 8521.8) | 138.3 (121.6 to 156.7) | 3325.5 (2981.5 to 3715.2) | 7887.7 (7029.2 to 8864.5) | 137.2 (118.2 to 157.2) |
|  |  | Age-standardized (rate per 100,000) | 4447.1 (4035 to 4924.8) | 7653.3 (6871 to 8524.3) | 72.1 (61.2 to 82) | 4259.4 (3854.3 to 4726.7) | 7022.5 (6269.3 to 7837.4) | 64.9 (52.6 to 77.3) | 4638.3 (4172.2 to 5164.3) | 8414.4 (7497.8 to 9458.4) | 81.4 (66.9 to 96.8) |
|  | **Deaths** | All ages  (number) | 400 (345 to 481) | 821 (588 to 1052) | 105.2 (46.3 to 170.6) | 178 (147 to 216) | 328 (244 to 455) | 84 (33.4 to 163.6) | 222 (184 to 271) | 493 (305 to 689) | 122.4 (35.6 to 218.5) |
|  |  | All ages  (rate per 100,000) | 12.2 (10.5 to 14.7) | 15.9 (11.4 to 20.3) | 29.8 (-7.5 to 71.2) | 11 (9.1 to 13.3) | 12.5 (9.3 to 17.3) | 13.4 (-17.8 to 62.5) | 13.4 (11.1 to 16.4) | 19.3 (12 to 27.1) | 44.4 (-11.9 to 106.8) |
|  |  | Age-standardized (rate per 100,000) | 20.4 (17.5 to 24.4) | 15.9 (11.4 to 20.4) | -21.9 (-44.4 to 2.8) | 17.7 (14.5 to 21.5) | 11.6 (8.7 to 16.1) | -34.3 (-52.4 to -6.1) | 23.3 (19.4 to 28.4) | 21.2 (13.3 to 29.6) | -9 (-44.3 to 30.8) |
|  | **DALYs** | All ages  (number) | 18443 (14745 to 22808) | 51734 (38476 to 67616) | 180.5 (139.6 to 216.4) | 8697 (6870 to 10862) | 24535 (18342 to 32523) | 182.1 (142.6 to 222.8) | 9746 (7807 to 12268) | 27199 (19804 to 35830) | 179.1 (126.4 to 227.5) |
|  |  | All ages  (rate per 100,000) | 563.1 (450.2 to 696.4) | 999.3 (743.2 to 1306.1) | 77.5 (51.6 to 100.2) | 536.6 (423.9 to 670.2) | 933.1 (697.6 to 1236.9) | 73.9 (49.5 to 99) | 589.1 (471.9 to 741.5) | 1067.6 (777.3 to 1406.4) | 81.2 (47 to 112.6) |
|  |  | Age-standardized (rate per 100,000) | 801.8 (649.5 to 990.4) | 991.2 (737.8 to 1295) | 23.6 (5.2 to 39.6) | 740.8 (590.9 to 919.8) | 860.9 (643.7 to 1142.3) | 16.2 (-0.4 to 33.1) | 864.6 (699.8 to 1074.3) | 1149.7 (838.7 to 1510.5) | 33 (7.8 to 57.3) |
|  | **YLLs** | All ages  (number) | 8732 (7503 to 10545) | 15278 (11074 to 19633) | 75 (24.8 to 132.8) | 3976 (3241 to 4837) | 6173 (4584 to 8504) | 55.3 (13.3 to 119.6) | 4756 (3914 to 5902) | 9105 (5653 to 12845) | 91.4 (19.1 to 179.4) |
|  |  | All ages  (rate per 100,000) | 266.6 (229.1 to 322) | 295.1 (213.9 to 379.2) | 10.7 (-21.1 to 47.3) | 245.3 (200 to 298.5) | 234.8 (174.3 to 323.4) | -4.3 (-30.2 to 35.4) | 287.5 (236.5 to 356.8) | 357.4 (221.9 to 504.2) | 24.3 (-22.6 to 81.4) |
|  |  | Age-standardized (rate per 100,000) | 392.4 (338.8 to 470) | 293.4 (212.6 to 377.3) | -25.2 (-46.9 to -0.9) | 348 (285.6 to 422.2) | 217 (161 to 299.7) | -37.6 (-54.4 to -11.6) | 438.3 (364.1 to 537.7) | 386.3 (239.1 to 542.5) | -11.9 (-45.9 to 26.2) |
|  | **YLDs** | All ages  (number) | 9711 (6528 to 13526) | 36456 (23925 to 51161) | 275.4 (250.8 to 297.6) | 4721 (3169 to 6598) | 18362 (12089 to 26074) | 289 (257.7 to 321.6) | 4990 (3313 to 6977) | 18094 (11948 to 25573) | 262.6 (232.7 to 294.3) |
|  |  | All ages  (rate per 100,000) | 296.5 (199.3 to 413) | 704.2 (462.1 to 988.2) | 137.5 (121.9 to 151.5) | 291.3 (195.5 to 407.1) | 698.3 (459.8 to 991.6) | 139.7 (120.5 to 159.9) | 301.6 (200.3 to 421.7) | 710.2 (469 to 1003.8) | 135.5 (116.1 to 156.1) |
|  |  | Age-standardized (rate per 100,000) | 409.4 (276 to 567.9) | 697.9 (456.4 to 980.6) | 70.5 (58.8 to 80.7) | 392.8 (264.8 to 551) | 643.9 (424.2 to 913.8) | 63.9 (51 to 76.4) | 426.3 (283.1 to 593.4) | 763.4 (503.8 to 1079.4) | 79.1 (64.2 to 94.7) |
| **Libya** | **Incidence** | All ages  (number) | 6200 (5615 to 6844) | 33010 (29472 to 37016) | 432.5 (396.7 to 466.8) | 2652 (2385 to 2945) | 15512 (13765 to 17593) | 485 (436.7 to 536.9) | 3548 (3211 to 3944) | 17498 (15391 to 19917) | 393.2 (351.2 to 434.8) |
|  |  | All ages  (rate per 100,000) | 146.3 (132.5 to 161.5) | 490.1 (437.6 to 549.6) | 234.9 (212.4 to 256.5) | 131.2 (118 to 145.7) | 476.3 (422.7 to 540.2) | 263.2 (233.2 to 295.4) | 160.2 (145 to 178.1) | 503 (442.4 to 572.5) | 214 (187.3 to 240.6) |
|  |  | Age-standardized (rate per 100,000) | 244.7 (221.3 to 271.1) | 454.8 (409.7 to 506.5) | 85.8 (75.1 to 95.7) | 235 (210.4 to 260.3) | 443.9 (398.4 to 494.1) | 88.9 (75.5 to 103.7) | 252.2 (227.7 to 281.2) | 465.1 (416.3 to 520.1) | 84.4 (70.8 to 97.4) |
|  | **Prevalence** | All ages  (number) | 99974 (89656 to 110772) | 545926 (486543 to 612688) | 446.1 (412.3 to 476.4) | 43744 (38923 to 49021) | 257939 (227323 to 290205) | 489.7 (447.6 to 536.5) | 56230 (50155 to 62751) | 287987 (254624 to 325638) | 412.2 (370.6 to 452.4) |
|  |  | All ages  (rate per 100,000) | 2359.6 (2116.1 to 2614.5) | 8105.1 (7223.5 to 9096.3) | 243.5 (222.2 to 262.5) | 2163.7 (1925.3 to 2424.7) | 7920.3 (6980.2 to 8911.1) | 266 (240 to 295.1) | 2538.4 (2264.1 to 2832.8) | 8278.2 (7319.2 to 9360.5) | 226.1 (199.7 to 251.7) |
|  |  | Age-standardized (rate per 100,000) | 4770.4 (4282.1 to 5283.8) | 9292 (8328.3 to 10384.7) | 94.8 (83 to 105.6) | 4573.8 (4072.5 to 5122.4) | 9029.1 (8012 to 10112.9) | 97.4 (83.3 to 113.1) | 4942.6 (4433.3 to 5516) | 9543.8 (8488.5 to 10713.8) | 93.1 (78 to 107.8) |
|  | **Deaths** | All ages  (number) | 238 (174 to 300) | 834 (605 to 1106) | 250.4 (143.5 to 400) | 126 (94 to 178) | 444 (318 to 596) | 251.6 (142.1 to 406.1) | 112 (78 to 147) | 390 (249 to 571) | 249.1 (119.8 to 423.3) |
|  |  | All ages  (rate per 100,000) | 5.6 (4.1 to 7.1) | 12.4 (9 to 16.4) | 120.4 (53.1 to 214.5) | 6.2 (4.6 to 8.8) | 13.6 (9.8 to 18.3) | 118.3 (50.3 to 214.2) | 5 (3.5 to 6.7) | 11.2 (7.1 to 16.4) | 122.3 (40 to 233.2) |
|  |  | Age-standardized (rate per 100,000) | 14.2 (10.5 to 18) | 18.3 (13.3 to 24.1) | 28.9 (-10 to 82.3) | 15.7 (11.5 to 22.3) | 19.7 (14.2 to 26.3) | 26 (-13.3 to 81.6) | 12.9 (9.1 to 17) | 17 (10.8 to 24.7) | 31.3 (-17.7 to 96.9) |
|  | **DALYs** | All ages  (number) | 13403 (10317 to 16938) | 62338 (46425 to 81547) | 365.1 (305.6 to 437.7) | 6382 (4913 to 8122) | 30844 (22922 to 39910) | 383.3 (307.7 to 471) | 7021 (5307 to 9041) | 31495 (22521 to 42190) | 348.6 (280.5 to 422.4) |
|  |  | All ages  (rate per 100,000) | 316.3 (243.5 to 399.8) | 925.5 (689.3 to 1210.7) | 192.6 (155.2 to 238.2) | 315.7 (243 to 401.7) | 947.1 (703.8 to 1225.5) | 200 (153.1 to 254.5) | 317 (239.6 to 408.1) | 905.3 (647.4 to 1212.8) | 185.6 (142.3 to 232.6) |
|  |  | Age-standardized (rate per 100,000) | 686.8 (531.8 to 866.7) | 1138.8 (859.7 to 1475.2) | 65.8 (43.5 to 92.1) | 706.7 (544.9 to 899) | 1154 (864.2 to 1481.4) | 63.3 (37.5 to 92.4) | 670.2 (509.2 to 855.9) | 1124 (810.6 to 1491.8) | 67.7 (41.2 to 96) |
|  | **YLLs** | All ages  (number) | 5312 (3934 to 6659) | 19018 (13730 to 25491) | 258 (145.4 to 422.1) | 2802 (2110 to 3813) | 10119 (7250 to 13635) | 261.1 (147.1 to 429) | 2510 (1723 to 3368) | 8899 (5601 to 13108) | 254.5 (118.6 to 440.5) |
|  |  | All ages  (rate per 100,000) | 125.4 (92.8 to 157.2) | 282.4 (203.8 to 378.5) | 125.2 (54.4 to 228.4) | 138.6 (104.4 to 188.6) | 310.7 (222.6 to 418.7) | 124.2 (53.4 to 228.4) | 113.3 (77.8 to 152) | 255.8 (161 to 376.8) | 125.7 (39.2 to 244.1) |
|  |  | Age-standardized (rate per 100,000) | 286.5 (211.7 to 359.5) | 373.6 (271 to 494.8) | 30.4 (-10.1 to 88) | 321.7 (241.4 to 444.2) | 406 (289.9 to 545.6) | 26.2 (-13.7 to 83.2) | 255.9 (176.1 to 342.6) | 342.2 (217.1 to 505.5) | 33.7 (-17.2 to 102.3) |
|  | **YLDs** | All ages  (number) | 8091 (5381 to 11295) | 43320 (28399 to 61006) | 435.4 (400 to 466.2) | 3580 (2359 to 4991) | 20725 (13391 to 29309) | 478.9 (433.9 to 526.9) | 4511 (2969 to 6393) | 22595 (14884 to 31686) | 400.9 (361.7 to 440.8) |
|  |  | All ages  (rate per 100,000) | 191 (127 to 266.6) | 643.2 (421.6 to 905.7) | 236.8 (214.5 to 256.2) | 177.1 (116.7 to 246.9) | 636.4 (411.2 to 900) | 259.4 (231.4 to 289.2) | 203.6 (134 to 288.6) | 649.5 (427.8 to 910.8) | 219 (194 to 244.4) |
|  |  | Age-standardized (rate per 100,000) | 400.3 (266.4 to 561.8) | 765.1 (504.9 to 1074) | 91.2 (79.1 to 101.9) | 385 (254.2 to 538.7) | 748 (484.7 to 1055.6) | 94.3 (80.1 to 110.3) | 414.3 (274.2 to 583.7) | 781.8 (517.7 to 1098.8) | 88.7 (73.6 to 103) |
| **Morocco** | **Incidence** | All ages  (number) | 31322 (28198 to 34674) | 129305 (116581 to 143166) | 312.8 (291.6 to 337.5) | 15766 (14134 to 17523) | 64804 (57743 to 71901) | 311 (282.1 to 344.4) | 15556 (13942 to 17272) | 64501 (57907 to 71997) | 314.6 (283.6 to 346.1) |
|  |  | All ages  (rate per 100,000) | 123.8 (111.5 to 137.1) | 359.7 (324.3 to 398.2) | 190.5 (175.5 to 207.9) | 124.2 (111.4 to 138.1) | 362.6 (323.1 to 402.3) | 191.8 (171.3 to 215.5) | 123.4 (110.6 to 137) | 356.8 (320.3 to 398.3) | 189.1 (167.5 to 211.1) |
|  |  | Age-standardized (rate per 100,000) | 178.7 (161.4 to 198.1) | 345.1 (311.5 to 378.7) | 93.1 (83 to 103.3) | 177.2 (158.2 to 198.1) | 345.4 (308.9 to 380.8) | 94.9 (80.6 to 109.3) | 180.1 (162.2 to 201) | 344.6 (310.3 to 384.2) | 91.4 (78.4 to 104.6) |
|  | **Prevalence** | All ages  (number) | 515583 (459272 to 574640) | 2331322 (2085396 to 2596446) | 352.2 (327 to 377) | 260272 (231471 to 292254) | 1180625 (1054267 to 1327495) | 353.6 (320.7 to 392.3) | 255311 (225982 to 285012) | 1150697 (1019976 to 1290438) | 350.7 (318 to 382.6) |
|  |  | All ages  (rate per 100,000) | 2038.2 (1815.6 to 2271.6) | 6484.5 (5800.5 to 7221.9) | 218.2 (200.5 to 235.7) | 2051 (1824 to 2303) | 6605.1 (5898.2 to 7426.8) | 222 (198.7 to 249.5) | 2025.2 (1792.6 to 2260.8) | 6365.2 (5642.1 to 7138.2) | 214.3 (191.5 to 236.5) |
|  |  | Age-standardized (rate per 100,000) | 3419.6 (3061.9 to 3803.5) | 6918 (6211.8 to 7684) | 102.3 (91.7 to 113.5) | 3398 (3024.5 to 3807.5) | 6952.1 (6205.4 to 7793.8) | 104.6 (89.3 to 121.3) | 3440.4 (3069.9 to 3843.2) | 6883.7 (6137.4 to 7694.8) | 100.1 (86.2 to 113.7) |
|  | **Deaths** | All ages  (number) | 1562 (1254 to 2196) | 6196 (4687 to 7779) | 296.7 (201.2 to 396.5) | 913 (678 to 1473) | 3588 (2707 to 5032) | 292.9 (191.1 to 424) | 649 (503 to 819) | 2607 (1899 to 3267) | 302 (190.9 to 427.6) |
|  |  | All ages  (rate per 100,000) | 6.2 (5 to 8.7) | 17.2 (13 to 21.6) | 179.1 (111.9 to 249.4) | 7.2 (5.3 to 11.6) | 20.1 (15.1 to 28.2) | 179 (106.7 to 272) | 5.1 (4 to 6.5) | 14.4 (10.5 to 18.1) | 180.3 (102.9 to 267.9) |
|  |  | Age-standardized (rate per 100,000) | 13.1 (10.4 to 18.6) | 22.4 (17.2 to 28.3) | 70.8 (31.5 to 112.6) | 14.9 (11 to 24.8) | 25.2 (19.3 to 36) | 69.8 (27.9 to 125.1) | 11.3 (8.8 to 14.1) | 19.4 (14.3 to 24.1) | 72.3 (26.1 to 122.8) |
|  | **DALYs** | All ages  (number) | 77215 (61491 to 97915) | 325869 (252352 to 408280) | 322 (267.1 to 374.4) | 42101 (32973 to 57202) | 176716 (136021 to 226228) | 319.7 (246.7 to 393.5) | 35113 (27355 to 44025) | 149153 (111316 to 190483) | 324.8 (266.6 to 384.6) |
|  |  | All ages  (rate per 100,000) | 305.2 (243.1 to 387.1) | 906.4 (701.9 to 1135.6) | 196.9 (158.3 to 233.8) | 331.8 (259.8 to 450.8) | 988.7 (761 to 1265.7) | 198 (146.2 to 250.4) | 278.5 (217 to 349.2) | 825.1 (615.8 to 1053.7) | 196.2 (155.6 to 237.9) |
|  |  | Age-standardized (rate per 100,000) | 546.7 (436.7 to 695.7) | 1008.6 (786.9 to 1261.8) | 84.5 (60.9 to 106.9) | 587.1 (463.2 to 799.8) | 1080.4 (835.1 to 1373) | 84 (51.9 to 115.6) | 505.6 (393.3 to 628.5) | 936.1 (701.8 to 1175.7) | 85.1 (59.8 to 110.5) |
|  | **YLLs** | All ages  (number) | 35913 (29077 to 48618) | 138463 (103571 to 174003) | 285.5 (183.4 to 391.9) | 21210 (15712 to 32280) | 81374 (60855 to 110077) | 283.7 (172.6 to 430.4) | 14704 (11379 to 18581) | 57089 (40539 to 72533) | 288.3 (174.1 to 423) |
|  |  | All ages  (rate per 100,000) | 142 (114.9 to 192.2) | 385.1 (288.1 to 484) | 171.3 (99.4 to 246.1) | 167.1 (123.8 to 254.4) | 455.3 (340.5 to 615.8) | 172.4 (93.5 to 276.6) | 116.6 (90.3 to 147.4) | 315.8 (224.2 to 401.2) | 170.8 (91.1 to 264.7) |
|  |  | Age-standardized (rate per 100,000) | 264.1 (212.6 to 362.4) | 443.2 (333.4 to 555.2) | 67.8 (26.1 to 110.7) | 306.2 (227.5 to 482) | 510.9 (383.4 to 696.9) | 66.9 (20.4 to 127) | 221.3 (172.5 to 278.6) | 374.7 (271.9 to 470.9) | 69.3 (21.3 to 122.4) |
|  | **YLDs** | All ages  (number) | 41301 (27360 to 58725) | 187406 (122622 to 263914) | 353.8 (328.7 to 380.7) | 20892 (13930 to 29781) | 95341 (62198 to 133305) | 356.4 (321.7 to 396.8) | 20410 (13375 to 28920) | 92064 (60767 to 131102) | 351.1 (317.3 to 383.7) |
|  |  | All ages  (rate per 100,000) | 163.3 (108.2 to 232.1) | 521.3 (341.1 to 734.1) | 219.3 (201.6 to 238.2) | 164.6 (109.8 to 234.7) | 533.4 (348 to 745.8) | 224 (199.4 to 252.7) | 161.9 (106.1 to 229.4) | 509.3 (336.1 to 725.2) | 214.6 (191 to 237.3) |
|  |  | Age-standardized (rate per 100,000) | 282.7 (188.3 to 400.5) | 565.4 (372.4 to 796.4) | 100 (89.2 to 111.7) | 280.9 (186.5 to 399.7) | 569.5 (373.7 to 799.5) | 102.7 (87.6 to 119.3) | 284.3 (187 to 399.1) | 561.4 (369.9 to 794.8) | 97.5 (83.6 to 111.2) |
| **Oman** | **Incidence** | All ages  (number) | 2707 (2450 to 2991) | 14921 (13301 to 16609) | 451.3 (416.5 to 485.6) | 996 (905 to 1096) | 4517 (4071 to 5036) | 353.5 (322.4 to 387.7) | 1711 (1528 to 1907) | 10404 (9192 to 11684) | 508.2 (459.9 to 560.7) |
|  |  | All ages  (rate per 100,000) | 139.3 (126.1 to 153.9) | 325.5 (290.2 to 362.3) | 133.7 (118.9 to 148.2) | 123.1 (111.8 to 135.3) | 277.3 (249.9 to 309.1) | 125.3 (109.9 to 142.3) | 150.9 (134.8 to 168.2) | 352.1 (311.1 to 395.4) | 133.4 (114.8 to 153.5) |
|  |  | Age-standardized (rate per 100,000) | 242 (222.1 to 264.4) | 410.3 (374.4 to 447.8) | 69.6 (60.5 to 79) | 238.6 (218.3 to 261.3) | 370.1 (336.6 to 409.8) | 55.1 (44.9 to 66.3) | 244.2 (221.2 to 267.8) | 433.4 (393.5 to 474.6) | 77.5 (66.5 to 90.9) |
|  | **Prevalence** | All ages  (number) | 36478 (32624 to 40863) | 192772 (170019 to 219061) | 428.5 (397.9 to 460.5) | 14712 (13158 to 16500) | 64057 (57072 to 71890) | 335.4 (304.2 to 371.1) | 21767 (19157 to 24615) | 128714 (112560 to 147284) | 491.3 (443.6 to 540.6) |
|  |  | All ages  (rate per 100,000) | 1877.2 (1678.9 to 2102.9) | 4205.3 (3709 to 4778.8) | 124 (111 to 137.6) | 1817.3 (1625.3 to 2038.2) | 3931.7 (3502.9 to 4412.4) | 116.3 (100.9 to 134.1) | 1920.1 (1689.8 to 2171.3) | 4356.2 (3809.5 to 4984.7) | 126.9 (108.6 to 145.8) |
|  |  | Age-standardized (rate per 100,000) | 4248.8 (3825.3 to 4693.4) | 7423.3 (6666.4 to 8199.8) | 74.7 (64.5 to 84.7) | 4237.4 (3785.3 to 4715.8) | 6823.8 (6125.4 to 7667) | 61 (49.2 to 74.5) | 4303.8 (3831.3 to 4795.7) | 7906.7 (7072.4 to 8756) | 83.7 (69.5 to 98.3) |
|  | **Deaths** | All ages  (number) | 233 (179 to 301) | 642 (547 to 743) | 175.4 (105.2 to 271.8) | 123 (89 to 165) | 284 (236 to 337) | 130.2 (65.4 to 236.5) | 110 (81 to 143) | 358 (277 to 439) | 226.2 (114.5 to 381.4) |
|  |  | All ages  (rate per 100,000) | 12 (9.2 to 15.5) | 14 (11.9 to 16.2) | 16.7 (-13 to 57.6) | 15.2 (11 to 20.4) | 17.4 (14.5 to 20.7) | 14.4 (-17.8 to 67.2) | 9.7 (7.2 to 12.6) | 12.1 (9.4 to 14.8) | 25.2 (-17.7 to 84.7) |
|  |  | Age-standardized (rate per 100,000) | 44.7 (34.2 to 57.2) | 58.3 (50.4 to 66.5) | 30.4 (-1.9 to 74) | 48.5 (35.1 to 64.3) | 54.1 (44.5 to 66) | 11.6 (-18.7 to 65) | 41.8 (32.2 to 53.2) | 64.2 (51.5 to 76.7) | 53.5 (6 to 114.8) |
|  | **DALYs** | All ages  (number) | 8539 (6732 to 10396) | 29392 (23840 to 36087) | 244.2 (175.2 to 323) | 4094 (3123 to 5124) | 11506 (9387 to 13987) | 181.1 (118.1 to 264.1) | 4445 (3477 to 5504) | 17886 (14012 to 22362) | 302.4 (207.3 to 407.3) |
|  |  | All ages  (rate per 100,000) | 439.4 (346.4 to 535) | 641.2 (520.1 to 787.2) | 45.9 (16.7 to 79.3) | 505.7 (385.8 to 633) | 706.2 (576.1 to 858.5) | 39.7 (8.4 to 80.9) | 392.1 (306.7 to 485.5) | 605.3 (474.2 to 756.8) | 54.4 (17.9 to 94.7) |
|  |  | Age-standardized (rate per 100,000) | 1248.3 (992 to 1505.9) | 1618.5 (1363.6 to 1912.1) | 29.6 (4.1 to 58.3) | 1351.1 (1035.2 to 1685.8) | 1544 (1284.1 to 1845) | 14.3 (-11 to 49.2) | 1176.9 (929.2 to 1453.2) | 1713.3 (1403.5 to 2051.3) | 45.6 (11.1 to 83.9) |
|  | **YLLs** | All ages  (number) | 5718 (4286 to 7427) | 14838 (12190 to 17396) | 159.5 (87.8 to 255.7) | 2929 (2075 to 3927) | 6490 (5352 to 7773) | 121.6 (57.9 to 224.6) | 2790 (2037 to 3700) | 8348 (6206 to 10437) | 199.2 (90 to 353.7) |
|  |  | All ages  (rate per 100,000) | 294.3 (220.6 to 382.2) | 323.7 (265.9 to 379.5) | 10 (-20.4 to 50.8) | 361.8 (256.4 to 485.1) | 398.3 (328.5 to 477.1) | 10.1 (-21.5 to 61.3) | 246.1 (179.7 to 326.3) | 282.5 (210 to 353.2) | 14.8 (-27.1 to 74.1) |
|  |  | Age-standardized (rate per 100,000) | 898 (688.9 to 1161.4) | 1006.5 (863.5 to 1159.2) | 12.1 (-16.2 to 50.8) | 1001.9 (720.4 to 1340) | 979.1 (815.1 to 1168.9) | -2.3 (-30.4 to 43.8) | 820.9 (613.3 to 1064.7) | 1060.5 (835.5 to 1284.8) | 29.2 (-13.8 to 85) |
|  | **YLDs** | All ages  (number) | 2820 (1862 to 3994) | 14554 (9531 to 20489) | 416 (384.5 to 450.5) | 1165 (775 to 1665) | 5016 (3242 to 7052) | 330.5 (298.9 to 366.9) | 1655 (1099 to 2359) | 9538 (6285 to 13660) | 476.2 (428.4 to 530.3) |
|  |  | All ages  (rate per 100,000) | 145.1 (95.8 to 205.5) | 317.5 (207.9 to 447) | 118.8 (105.4 to 133.4) | 143.9 (95.8 to 205.7) | 307.9 (199 to 432.8) | 113.9 (98.2 to 132) | 146 (97 to 208.1) | 322.8 (212.7 to 462.3) | 121.1 (102.7 to 141.8) |
|  |  | Age-standardized (rate per 100,000) | 350.3 (232.2 to 494.4) | 612 (404.2 to 857.6) | 74.7 (64.4 to 85.5) | 349.2 (233 to 496.7) | 564.9 (368 to 788.5) | 61.8 (49.7 to 76) | 356 (235.8 to 504.3) | 652.8 (429.2 to 916) | 83.4 (69.3 to 99.9) |
| **Palestine** | **Incidence** | All ages  (number) | 2769 (2537 to 3024) | 15518 (14193 to 16744) | 460.5 (419.2 to 503.8) | 1436 (1312 to 1571) | 6937 (6286 to 7560) | 383.2 (344.5 to 429.9) | 1333 (1215 to 1466) | 8581 (7775 to 9327) | 543.7 (483.4 to 603) |
|  |  | All ages  (rate per 100,000) | 133.8 (122.6 to 146.1) | 313.1 (286.3 to 337.8) | 134.1 (116.8 to 152.2) | 139.8 (127.7 to 153) | 285.3 (258.5 to 310.9) | 104.1 (87.8 to 123.8) | 127.8 (116.5 to 140.6) | 339.8 (307.9 to 369.4) | 165.9 (141 to 190.4) |
|  |  | Age-standardized (rate per 100,000) | 258.2 (237.5 to 281.7) | 452.7 (414.5 to 485.4) | 75.3 (62 to 89) | 250.5 (229.6 to 273.7) | 415.9 (380.1 to 452.5) | 66 (52.5 to 82.6) | 267.6 (244 to 296.4) | 486.7 (443.1 to 525.5) | 81.9 (64.6 to 100) |
|  | **Prevalence** | All ages  (number) | 42912 (38612 to 47562) | 225249 (203113 to 245840) | 424.9 (386.3 to 465.3) | 22250 (19946 to 24749) | 103216 (92289 to 113749) | 363.9 (323.6 to 408.7) | 20661 (18500 to 23028) | 122034 (108729 to 134358) | 490.6 (432.5 to 547.5) |
|  |  | All ages  (rate per 100,000) | 2073 (1865.3 to 2297.7) | 4544.4 (4097.8 to 4959.8) | 119.2 (103.1 to 136.1) | 2166.5 (1942.2 to 2409.8) | 4245.1 (3795.8 to 4678.4) | 95.9 (78.9 to 114.9) | 1980.9 (1773.7 to 2207.8) | 4832.6 (4305.7 to 5320.7) | 144 (119.9 to 167.5) |
|  |  | Age-standardized (rate per 100,000) | 4569.7 (4130.8 to 5053) | 8319.6 (7549.9 to 9013) | 82.1 (67.8 to 97.1) | 4366.1 (3931.3 to 4847.3) | 7566.6 (6813.8 to 8323.9) | 73.3 (57.7 to 90.8) | 4819.1 (4316.2 to 5387.5) | 9104.7 (8189.9 to 9907.9) | 88.9 (70.2 to 108.1) |
|  | **Deaths** | All ages  (number) | 413 (327 to 511) | 1257 (1081 to 1443) | 204.8 (138 to 298.5) | 237 (186 to 298) | 658 (553 to 764) | 177.2 (114 to 265.9) | 175 (132 to 227) | 599 (510 to 698) | 242.2 (151.7 to 367.2) |
|  |  | All ages  (rate per 100,000) | 19.9 (15.8 to 24.7) | 25.4 (21.8 to 29.1) | 27.3 (-0.6 to 66.4) | 23.1 (18.1 to 29) | 27.1 (22.8 to 31.4) | 17.1 (-9.6 to 54.5) | 16.8 (12.7 to 21.8) | 23.7 (20.2 to 27.6) | 41.3 (4 to 93) |
|  |  | Age-standardized (rate per 100,000) | 53.9 (43 to 66.4) | 68.8 (59.5 to 78.7) | 27.7 (0.8 to 65.3) | 56.5 (44.4 to 70.9) | 67 (56.6 to 77.8) | 18.5 (-8.7 to 56.4) | 51 (38.7 to 65.5) | 71.5 (61 to 83) | 40.3 (5 to 88.4) |
|  | **DALYs** | All ages  (number) | 12033 (9685 to 14775) | 44332 (37713 to 52723) | 268.4 (203.3 to 346.9) | 6667 (5387 to 8180) | 21179 (17709 to 25360) | 217.7 (159.7 to 291.2) | 5366 (4200 to 6743) | 23154 (19382 to 27506) | 331.5 (242.2 to 435.9) |
|  |  | All ages  (rate per 100,000) | 581.3 (467.9 to 713.8) | 894.4 (760.9 to 1063.7) | 53.9 (26.7 to 86.6) | 649.2 (524.5 to 796.5) | 871.1 (728.4 to 1043) | 34.2 (9.7 to 65.3) | 514.4 (402.7 to 646.4) | 916.9 (767.5 to 1089.3) | 78.2 (41.4 to 121.4) |
|  |  | Age-standardized (rate per 100,000) | 1398.9 (1131.1 to 1700.6) | 1907.9 (1642.6 to 2233.1) | 36.4 (13.4 to 65.9) | 1422.6 (1150.7 to 1739.6) | 1790.1 (1515.6 to 2122.5) | 25.8 (3 to 55.2) | 1375.9 (1078.3 to 1729.6) | 2031.4 (1725.9 to 2395.2) | 47.6 (17.5 to 83.4) |
|  | **YLLs** | All ages  (number) | 8521 (6658 to 10708) | 26348 (22549 to 30430) | 209.2 (136.4 to 309.5) | 4824 (3744 to 6069) | 12798 (10807 to 14960) | 165.3 (100.9 to 255.3) | 3697 (2717 to 4845) | 13549 (11418 to 15884) | 266.5 (165.9 to 408) |
|  |  | All ages  (rate per 100,000) | 411.6 (321.6 to 517.3) | 531.6 (454.9 to 613.9) | 29.1 (-1.3 to 71) | 469.7 (364.6 to 590.9) | 526.4 (444.5 to 615.3) | 12.1 (-15.2 to 50.1) | 354.5 (260.5 to 464.5) | 536.6 (452.1 to 629) | 51.4 (9.8 to 109.8) |
|  |  | Age-standardized (rate per 100,000) | 1014.7 (799.2 to 1267.6) | 1217.3 (1049.1 to 1399.1) | 20 (-7.3 to 57) | 1052.9 (818.8 to 1319) | 1155.5 (974.8 to 1347.1) | 9.7 (-16.7 to 46.8) | 973.9 (724.5 to 1272) | 1281 (1088.6 to 1491.3) | 31.5 (-3.8 to 79.4) |
|  | **YLDs** | All ages  (number) | 3512 (2316 to 4904) | 17985 (12017 to 25082) | 412.1 (370.5 to 454.8) | 1844 (1228 to 2582) | 8380 (5565 to 11949) | 354.6 (312.6 to 398.7) | 1669 (1088 to 2377) | 9604 (6457 to 13315) | 475.6 (418.5 to 533.5) |
|  |  | All ages  (rate per 100,000) | 169.7 (111.9 to 236.9) | 362.8 (242.4 to 506) | 113.9 (96.5 to 131.7) | 179.5 (119.5 to 251.4) | 344.7 (228.9 to 491.5) | 92 (74.3 to 110.7) | 160 (104.3 to 227.9) | 380.3 (255.7 to 527.3) | 137.7 (114.2 to 161.7) |
|  |  | Age-standardized (rate per 100,000) | 384.2 (254.7 to 539.8) | 690.6 (455.8 to 971.4) | 79.7 (65.4 to 95.5) | 369.6 (246.1 to 517.3) | 634.5 (416.3 to 902) | 71.7 (56 to 89.3) | 402 (261.7 to 572.8) | 750.4 (500.9 to 1051.1) | 86.7 (67.6 to 106.4) |
| **Qatar** | **Incidence** | All ages  (number) | 1319 (1169 to 1489) | 23920 (21586 to 26587) | 1712.9 (1578.1 to 1876.2) | 340 (302 to 379) | 5299 (4641 to 5982) | 1459.1 (1307.1 to 1624.7) | 980 (854 to 1116) | 18620 (16855 to 20765) | 1800.9 (1639.7 to 2007) |
|  |  | All ages  (rate per 100,000) | 296.4 (262.6 to 334.6) | 835 (753.6 to 928.1) | 181.7 (160.8 to 207.1) | 230.1 (204.5 to 256.2) | 728.7 (638.2 to 822.6) | 216.8 (185.9 to 250.4) | 329.4 (287.3 to 375.2) | 871.2 (788.6 to 971.5) | 164.5 (142.1 to 193.2) |
|  |  | Age-standardized (rate per 100,000) | 493.1 (453.7 to 534.1) | 818 (773.9 to 868.7) | 65.9 (53.3 to 79.1) | 475.7 (435.2 to 517.7) | 821.7 (770.1 to 874.3) | 72.7 (57.4 to 87.1) | 497.6 (457 to 542) | 816.8 (771.4 to 873) | 64.1 (51.4 to 78.1) |
|  | **Prevalence** | All ages  (number) | 14781 (13050 to 16832) | 274291 (244493 to 309556) | 1755.7 (1622.7 to 1903.8) | 4105 (3636 to 4582) | 61371 (53652 to 70440) | 1395.1 (1258.2 to 1527) | 10676 (9357 to 12305) | 212920 (189232 to 240538) | 1894.3 (1728.7 to 2080.4) |
|  |  | All ages  (rate per 100,000) | 3320.6 (2931.8 to 3781.3) | 9575.4 (8535.1 to 10806.4) | 188.4 (167.7 to 211.4) | 2778.2 (2460.7 to 3101.3) | 8439.3 (7377.8 to 9686.5) | 203.8 (176 to 230.6) | 3590.1 (3146.4 to 4137.7) | 9961.9 (8853.6 to 11254.1) | 177.5 (154.4 to 203.4) |
|  |  | Age-standardized (rate per 100,000) | 8552.6 (7728.4 to 9442.2) | 16312.4 (15050 to 17723.2) | 90.7 (75.2 to 107.8) | 8308.7 (7432.4 to 9176.1) | 16294.9 (14866.9 to 17796.6) | 96.1 (76.2 to 116.4) | 8738.4 (7854.8 to 9738.4) | 16309.7 (15082.8 to 17843.8) | 86.6 (70.8 to 105.6) |
|  | **Deaths** | All ages  (number) | 62 (52 to 74) | 336 (253 to 438) | 440.5 (291.5 to 638) | 26 (21 to 32) | 109 (84 to 140) | 315 (209.2 to 446.2) | 36 (28 to 46) | 227 (163 to 302) | 532.5 (316.9 to 829) |
|  |  | All ages  (rate per 100,000) | 13.9 (11.7 to 16.6) | 11.7 (8.8 to 15.3) | -16 (-39.2 to 14.7) | 17.8 (14.1 to 21.9) | 15 (11.6 to 19.2) | -15.7 (-37.2 to 11) | 12 (9.5 to 15.4) | 10.6 (7.6 to 14.1) | -12 (-42 to 29.3) |
|  |  | Age-standardized (rate per 100,000) | 111.2 (94.4 to 130.9) | 122.1 (98.9 to 151.5) | 9.8 (-15.1 to 41.1) | 111.9 (88.7 to 138.7) | 173.2 (134.6 to 213.1) | 54.8 (18.9 to 100.1) | 112.7 (91.1 to 137.9) | 107.4 (83.8 to 136.3) | -4.7 (-33 to 33.7) |
|  | **DALYs** | All ages  (number) | 2580 (2096 to 3128) | 28694 (21143 to 37665) | 1012 (819.1 to 1201.5) | 886 (726 to 1071) | 7198 (5469 to 9403) | 712.8 (542 to 877.8) | 1695 (1342 to 2118) | 21496 (15697 to 28547) | 1168.4 (920.4 to 1411.9) |
|  |  | All ages  (rate per 100,000) | 579.7 (470.9 to 702.8) | 1001.7 (738.1 to 1314.8) | 72.8 (42.8 to 102.2) | 599.4 (491.6 to 724.7) | 989.9 (752 to 1293) | 65.1 (30.4 to 98.7) | 569.9 (451.4 to 712.4) | 1005.7 (734.4 to 1335.6) | 76.5 (42 to 110.4) |
|  |  | Age-standardized (rate per 100,000) | 2528.1 (2147.5 to 2948.9) | 2975.3 (2401.2 to 3673.1) | 17.7 (-2.4 to 40.8) | 2575.2 (2107.6 to 3102.4) | 3628.6 (2952.6 to 4381.6) | 40.9 (15.3 to 70) | 2545.5 (2071.5 to 3057.8) | 2769.8 (2202.2 to 3468.9) | 8.8 (-15.4 to 36.8) |
|  | **YLLs** | All ages  (number) | 1476 (1217 to 1775) | 8115 (6017 to 10829) | 449.8 (287.9 to 674.8) | 568 (451 to 716) | 2474 (1891 to 3208) | 335.4 (218.3 to 490.9) | 908 (709 to 1170) | 5641 (4016 to 7657) | 521.4 (306 to 835) |
|  |  | All ages  (rate per 100,000) | 331.6 (273.3 to 398.8) | 283.3 (210.1 to 378) | -14.6 (-39.7 to 20.4) | 384.5 (305.4 to 484.8) | 340.2 (260 to 441.2) | -11.5 (-35.3 to 20.1) | 305.3 (238.5 to 393.5) | 263.9 (187.9 to 358.3) | -13.5 (-43.5 to 30.1) |
|  |  | Age-standardized (rate per 100,000) | 1811.9 (1525.5 to 2135.4) | 1637.2 (1298.7 to 2052.7) | -9.6 (-31.6 to 19.4) | 1877.7 (1477.4 to 2314.7) | 2282.2 (1814.3 to 2834.7) | 21.5 (-7.9 to 58.3) | 1811.8 (1438.2 to 2244.7) | 1434 (1085.4 to 1851.4) | -20.9 (-46.3 to 13.3) |
|  | **YLDs** | All ages  (number) | 1104 (716 to 1581) | 20579 (13511 to 29498) | 1763.3 (1625.9 to 1918.5) | 318 (210 to 447) | 4725 (3043 to 6834) | 1387.9 (1238 to 1540.8) | 787 (511 to 1136) | 15855 (10412 to 22779) | 1914.8 (1737.3 to 2115.1) |
|  |  | All ages  (rate per 100,000) | 248.1 (160.9 to 355.2) | 718.4 (471.7 to 1029.8) | 189.5 (168.2 to 213.7) | 214.9 (142 to 302.4) | 649.7 (418.5 to 939.8) | 202.3 (171.8 to 233.4) | 264.6 (172 to 382) | 741.8 (487.1 to 1065.8) | 180.3 (155.6 to 208.2) |
|  |  | Age-standardized (rate per 100,000) | 716.2 (473.9 to 1001.3) | 1338.1 (886.5 to 1857.3) | 86.8 (71.7 to 103.8) | 697.5 (460.3 to 971.2) | 1346.3 (886.6 to 1889.1) | 93 (73.1 to 113) | 733.7 (481.9 to 1045.4) | 1335.7 (887.6 to 1873.4) | 82.1 (65.5 to 100.8) |
| **Saudi Arabia** | **Incidence** | All ages  (number) | 27124 (24772 to 29575) | 169200 (151544 to 189934) | 523.8 (480.8 to 571.8) | 9883 (8984 to 10887) | 63777 (57105 to 72452) | 545.3 (494.6 to 604.4) | 17241 (15685 to 18938) | 105424 (93329 to 119636) | 511.5 (459.2 to 572.3) |
|  |  | All ages  (rate per 100,000) | 169 (154.4 to 184.3) | 473.5 (424.1 to 531.6) | 180.1 (160.8 to 201.7) | 139.7 (127 to 153.8) | 428.7 (383.8 to 487) | 206.9 (182.8 to 235.1) | 192.2 (174.9 to 211.2) | 505.5 (447.5 to 573.7) | 163 (140.5 to 189.1) |
|  |  | Age-standardized (rate per 100,000) | 282.7 (260.4 to 306.4) | 462.1 (420.8 to 506.9) | 63.5 (53.8 to 73.5) | 264 (241.8 to 289.6) | 441.8 (399.5 to 491.1) | 67.4 (56 to 80) | 294.1 (270.2 to 320.4) | 475.6 (431 to 526.3) | 61.7 (50 to 74.7) |
|  | **Prevalence** | All ages  (number) | 394590 (358027 to 434591) | 2486355 (2216629 to 2808991) | 530.1 (488.8 to 570.7) | 146324 (131622 to 161977) | 938434 (830124 to 1068156) | 541.3 (488.7 to 595) | 248266 (223278 to 275508) | 1547921 (1370940 to 1765594) | 523.5 (473.1 to 581.1) |
|  |  | All ages  (rate per 100,000) | 2459.3 (2231.4 to 2708.6) | 6958.3 (6203.5 to 7861.3) | 182.9 (164.4 to 201.2) | 2067.7 (1859.9 to 2288.9) | 6307.3 (5579.4 to 7179.2) | 205 (180 to 230.6) | 2768.2 (2489.6 to 3072) | 7422.8 (6574.1 to 8466.7) | 168.1 (146.5 to 192.9) |
|  |  | Age-standardized (rate per 100,000) | 5380.7 (4914.3 to 5869.4) | 9453.1 (8563.1 to 10498.6) | 75.7 (65.4 to 87.1) | 5018.1 (4534.4 to 5539.9) | 9037 (8145.5 to 10160.6) | 80.1 (67.1 to 94.2) | 5638.3 (5129.7 to 6194.5) | 9728.5 (8777.6 to 10881.7) | 72.5 (59.9 to 87.2) |
|  | **Deaths** | All ages  (number) | 1268 (950 to 1683) | 2797 (2211 to 3439) | 120.6 (53.4 to 212.3) | 579 (429 to 756) | 1029 (794 to 1369) | 77.6 (20.1 to 163.8) | 688 (497 to 964) | 1767 (1379 to 2169) | 156.7 (64.6 to 280.7) |
|  |  | All ages  (rate per 100,000) | 7.9 (5.9 to 10.5) | 7.8 (6.2 to 9.6) | -0.9 (-31.1 to 40.2) | 8.2 (6.1 to 10.7) | 6.9 (5.3 to 9.2) | -15.5 (-42.9 to 25.5) | 7.7 (5.5 to 10.8) | 8.5 (6.6 to 10.4) | 10.4 (-29.2 to 63.7) |
|  |  | Age-standardized (rate per 100,000) | 25.2 (19.2 to 33.1) | 19.5 (16.1 to 23.7) | -22.5 (-43.8 to 7) | 26.2 (19 to 34) | 17.6 (13.8 to 23.2) | -32.6 (-53.6 to 0.7) | 24.7 (18.1 to 33.9) | 20.8 (16.8 to 24.7) | -15.7 (-43.6 to 22.9) |
|  | **DALYs** | All ages  (number) | 58772 (46154 to 72873) | 245548 (187706 to 316976) | 317.8 (233.6 to 408.2) | 24624 (19276 to 30485) | 92536 (70820 to 120557) | 275.8 (191.8 to 368.6) | 34148 (26194 to 43523) | 153012 (115644 to 199551) | 348.1 (245.6 to 448.6) |
|  |  | All ages  (rate per 100,000) | 366.3 (287.7 to 454.2) | 687.2 (525.3 to 887.1) | 87.6 (49.8 to 128.2) | 348 (272.4 to 430.8) | 621.9 (476 to 810.3) | 78.7 (38.8 to 122.9) | 380.8 (292.1 to 485.3) | 733.7 (554.6 to 956.9) | 92.7 (48.7 to 135.9) |
|  |  | Age-standardized (rate per 100,000) | 902.3 (710.1 to 1117) | 1064.5 (829.7 to 1348.6) | 18 (-5.1 to 42.3) | 914.1 (722.8 to 1127.9) | 994.6 (771.1 to 1275.6) | 8.8 (-14.6 to 36.3) | 899 (691 to 1144.8) | 1110.4 (854 to 1407) | 23.5 (-4.8 to 52.7) |
|  | **YLLs** | All ages  (number) | 31896 (23373 to 42935) | 79972 (61876 to 101000) | 150.7 (69.5 to 265) | 14650 (10827 to 19493) | 29767 (22702 to 39743) | 103.2 (35.8 to 207.7) | 17247 (12282 to 24481) | 50206 (37453 to 63402) | 191.1 (81.8 to 352.2) |
|  |  | All ages  (rate per 100,000) | 198.8 (145.7 to 267.6) | 223.8 (173.2 to 282.7) | 12.6 (-23.9 to 63.9) | 207 (153 to 275.5) | 200.1 (152.6 to 267.1) | -3.4 (-35.4 to 46.3) | 192.3 (136.9 to 273) | 240.8 (179.6 to 304) | 25.2 (-21.8 to 94.5) |
|  |  | Age-standardized (rate per 100,000) | 518.5 (385.9 to 692.7) | 395.5 (319.1 to 483.1) | -23.7 (-46.5 to 7.3) | 559.7 (412 to 744.7) | 358.6 (276.5 to 474) | -35.9 (-56.4 to -4.2) | 493.4 (355.2 to 694.6) | 419.6 (329.8 to 508.3) | -15 (-44.7 to 26.1) |
|  | **YLDs** | All ages  (number) | 26876 (18001 to 36974) | 165576 (108817 to 232949) | 516.1 (468 to 574.7) | 9975 (6665 to 13786) | 62770 (40954 to 89009) | 529.3 (466.6 to 598.5) | 16901 (11282 to 23394) | 102806 (66836 to 147648) | 508.3 (454.8 to 580.1) |
|  |  | All ages  (rate per 100,000) | 167.5 (112.2 to 230.4) | 463.4 (304.5 to 651.9) | 176.6 (155.1 to 203) | 141 (94.2 to 194.8) | 421.9 (275.3 to 598.2) | 199.3 (169.5 to 232.2) | 188.4 (125.8 to 260.8) | 493 (320.5 to 708) | 161.6 (138.6 to 192.5) |
|  |  | Age-standardized (rate per 100,000) | 383.8 (260 to 523.8) | 668.9 (450.7 to 939.7) | 74.3 (61.6 to 90) | 354.3 (239.9 to 487) | 636.1 (414.5 to 892) | 79.5 (62.8 to 98.3) | 405.5 (272.8 to 558) | 690.8 (460.9 to 979.5) | 70.3 (55.2 to 89.4) |
| **Sudan** | **Incidence** | All ages  (number) | 22706 (20519 to 25117) | 100586 (90376 to 112084) | 343 (322.5 to 364.5) | 11222 (10100 to 12522) | 48675 (43594 to 54463) | 333.7 (308 to 361.7) | 11484 (10284 to 12802) | 51910 (46361 to 58063) | 352 (324 to 385.4) |
|  |  | All ages  (rate per 100,000) | 112.4 (101.6 to 124.3) | 246.5 (221.5 to 274.7) | 119.3 (109.1 to 129.9) | 112.2 (101 to 125.2) | 241.8 (216.6 to 270.6) | 115.5 (102.7 to 129.4) | 112.6 (100.8 to 125.5) | 251 (224.2 to 280.8) | 122.9 (109.1 to 139.4) |
|  |  | Age-standardized (rate per 100,000) | 186.5 (168.7 to 206.6) | 359.6 (323.4 to 399.6) | 92.8 (84.1 to 101.6) | 186.4 (167.7 to 207) | 355.3 (319.2 to 396.5) | 90.6 (79.1 to 102.7) | 186.4 (166.5 to 208.1) | 364 (325.5 to 407.6) | 95.2 (82.5 to 109.7) |
|  | **Prevalence** | All ages  (number) | 372731 (332103 to 417992) | 1592344 (1420144 to 1790129) | 327.2 (307.7 to 349) | 180145 (160288 to 202656) | 747723 (664897 to 841779) | 315.1 (289.1 to 342.6) | 192586 (170231 to 217619) | 844621 (746471 to 952832) | 338.6 (310.6 to 371.7) |
|  |  | All ages  (rate per 100,000) | 1845.3 (1644.2 to 2069.4) | 3902 (3480 to 4386.7) | 111.5 (101.8 to 122.2) | 1801.6 (1603 to 2026.7) | 3715 (3303.5 to 4182.3) | 106.2 (93.3 to 119.9) | 1888.2 (1669 to 2133.6) | 4084 (3609.4 to 4607.3) | 116.3 (102.5 to 132.6) |
|  |  | Age-standardized (rate per 100,000) | 3621.7 (3234.6 to 4040) | 7272 (6509.8 to 8134.3) | 100.8 (91.6 to 110.8) | 3603.1 (3211.9 to 4040) | 7155.5 (6396.4 to 8022) | 98.6 (86.1 to 112) | 3636.5 (3219.1 to 4088.5) | 7371.9 (6548.5 to 8260.8) | 102.7 (89.8 to 117.8) |
|  | **Deaths** | All ages  (number) | 957 (693 to 1334) | 2549 (1723 to 3552) | 166.3 (92.9 to 263) | 520 (344 to 825) | 1235 (811 to 1831) | 137.5 (75.9 to 222) | 437 (329 to 618) | 1314 (794 to 2010) | 200.5 (89.9 to 348.6) |
|  |  | All ages  (rate per 100,000) | 4.7 (3.4 to 6.6) | 6.2 (4.2 to 8.7) | 31.8 (-4.5 to 79.7) | 5.2 (3.4 to 8.3) | 6.1 (4 to 9.1) | 18 (-12.6 to 60) | 4.3 (3.2 to 6.1) | 6.4 (3.8 to 9.7) | 48.2 (-6.4 to 121.2) |
|  |  | Age-standardized (rate per 100,000) | 11.5 (8.4 to 16.4) | 15.7 (10.6 to 21.7) | 36 (-0.3 to 81.3) | 13 (8.7 to 21.1) | 16.6 (11 to 24.4) | 27.5 (-5.5 to 72.3) | 10.1 (7.6 to 14.1) | 14.9 (9.1 to 22.5) | 46.8 (-5.3 to 116) |
|  | **DALYs** | All ages  (number) | 49524 (37355 to 64065) | 172228 (126964 to 221856) | 247.8 (198.3 to 299.4) | 24898 (18381 to 33494) | 80584 (59600 to 104097) | 223.7 (163.6 to 279.8) | 24626 (18484 to 31828) | 91645 (66039 to 121542) | 272.1 (209.8 to 338.9) |
|  |  | All ages  (rate per 100,000) | 245.2 (184.9 to 317.2) | 422 (311.1 to 543.7) | 72.1 (47.6 to 97.7) | 249 (183.8 to 335) | 400.4 (296.1 to 517.2) | 60.8 (30.9 to 88.7) | 241.4 (181.2 to 312.1) | 443.1 (319.3 to 587.7) | 83.5 (52.8 to 116.5) |
|  |  | Age-standardized (rate per 100,000) | 510.3 (386.5 to 656.2) | 855.6 (643.6 to 1090.2) | 67.7 (43.6 to 92.6) | 534.2 (396.5 to 718.6) | 852.8 (641.3 to 1101.7) | 59.6 (30.1 to 87.6) | 488.6 (372.2 to 627.5) | 857.9 (619.2 to 1124.8) | 75.6 (46.6 to 107.2) |
|  | **YLLs** | All ages  (number) | 22788 (16261 to 31584) | 59127 (38801 to 85062) | 159.5 (84.7 to 265.6) | 12520 (8114 to 19208) | 29055 (18294 to 43177) | 132.1 (64.9 to 223.2) | 10267 (7514 to 14588) | 30071 (17690 to 46549) | 192.9 (84.4 to 348.7) |
|  |  | All ages  (rate per 100,000) | 112.8 (80.5 to 156.4) | 144.9 (95.1 to 208.4) | 28.4 (-8.6 to 80.9) | 125.2 (81.1 to 192.1) | 144.4 (90.9 to 214.5) | 15.3 (-18.1 to 60.6) | 100.7 (73.7 to 143) | 145.4 (85.5 to 225.1) | 44.4 (-9.1 to 121.3) |
|  |  | Age-standardized (rate per 100,000) | 242.4 (173.6 to 334.7) | 317.9 (212.1 to 447.1) | 31.2 (-5.5 to 83.1) | 278.4 (182.9 to 430.4) | 338.9 (219.5 to 498.2) | 21.7 (-10.7 to 67.7) | 210 (155.7 to 296.4) | 300.5 (181.7 to 463.6) | 43.1 (-8.7 to 116.6) |
|  | **YLDs** | All ages  (number) | 26737 (17756 to 37261) | 113102 (74008 to 159039) | 323 (293.6 to 357.3) | 12378 (8265 to 17180) | 51528 (34207 to 72114) | 316.3 (283 to 353.6) | 14359 (9610 to 20096) | 61573 (40078 to 87002) | 328.8 (290.9 to 372) |
|  |  | All ages  (rate per 100,000) | 132.4 (87.9 to 184.5) | 277.2 (181.4 to 389.7) | 109.4 (94.8 to 126.3) | 123.8 (82.7 to 171.8) | 256 (170 to 358.3) | 106.8 (90.3 to 125.4) | 140.8 (94.2 to 197) | 297.7 (193.8 to 420.7) | 111.5 (92.8 to 132.8) |
|  |  | Age-standardized (rate per 100,000) | 267.9 (179.1 to 373.1) | 537.7 (358.2 to 753.7) | 100.7 (87.1 to 116.4) | 255.8 (171 to 356.1) | 513.9 (343.3 to 713.3) | 100.9 (84.7 to 118.7) | 278.7 (186.6 to 390.7) | 557.4 (370.3 to 783) | 100 (82.1 to 120.8) |
| **Syrian Arab Republic** | **Incidence** | All ages  (number) | 16251 (14798 to 17839) | 51642 (46247 to 56782) | 217.8 (196.5 to 241.6) | 8089 (7316 to 8938) | 26500 (23536 to 29423) | 227.6 (201 to 257.5) | 8161 (7395 to 9017) | 25142 (22214 to 27925) | 208.1 (181.2 to 238.5) |
|  |  | All ages  (rate per 100,000) | 126 (114.8 to 138.3) | 356.4 (319.1 to 391.8) | 182.8 (163.8 to 204) | 128.4 (116.1 to 141.9) | 356.5 (316.6 to 395.8) | 177.6 (155 to 203) | 123.7 (112.1 to 136.7) | 356.2 (314.7 to 395.7) | 187.9 (162.8 to 216.3) |
|  |  | Age-standardized (rate per 100,000) | 224.5 (204.8 to 246) | 345.7 (312.5 to 378.2) | 54 (45.5 to 63.7) | 231.4 (208.5 to 255.3) | 351.1 (315.6 to 386) | 51.7 (40.6 to 64) | 218.3 (198.2 to 241) | 341.2 (305.9 to 374.4) | 56.3 (43.7 to 70.6) |
|  | **Prevalence** | All ages  (number) | 260867 (236014 to 290479) | 906551 (806813 to 1009546) | 247.5 (224.4 to 271.3) | 127938 (114301 to 143068) | 450687 (397795 to 504961) | 252.3 (222.6 to 284.2) | 132929 (118839 to 148862) | 455864 (402452 to 509713) | 242.9 (215.1 to 274.6) |
|  |  | All ages  (rate per 100,000) | 2023 (1830.3 to 2252.7) | 6255.9 (5567.6 to 6966.6) | 209.2 (188.7 to 230.4) | 2031.1 (1814.6 to 2271.3) | 6063.1 (5351.6 to 6793.3) | 198.5 (173.3 to 225.6) | 2015.3 (1801.7 to 2256.9) | 6458.8 (5702.1 to 7221.8) | 220.5 (194.4 to 250.1) |
|  |  | Age-standardized (rate per 100,000) | 4341.8 (3942.2 to 4797.3) | 6832.5 (6133.8 to 7554.8) | 57.4 (47.8 to 67.7) | 4419.1 (3952.2 to 4904.8) | 6852.5 (6071.4 to 7632.5) | 55.1 (43.2 to 68.8) | 4271.1 (3826.6 to 4746.7) | 6824 (6058.8 to 7597.9) | 59.8 (47.3 to 74.3) |
|  | **Deaths** | All ages  (number) | 873 (683 to 1089) | 1599 (1216 to 2139) | 83.2 (33.8 to 162.8) | 503 (375 to 641) | 850 (645 to 1136) | 69.2 (21.5 to 151.4) | 370 (286 to 471) | 748 (548 to 997) | 102.2 (35.1 to 198.8) |
|  |  | All ages  (rate per 100,000) | 6.8 (5.3 to 8.4) | 11 (8.4 to 14.8) | 63 (19.1 to 133.9) | 8 (6 to 10.2) | 11.4 (8.7 to 15.3) | 43.4 (2.9 to 113) | 5.6 (4.3 to 7.1) | 10.6 (7.8 to 14.1) | 88.9 (26.3 to 179.2) |
|  |  | Age-standardized (rate per 100,000) | 18.7 (14.4 to 23.2) | 15.9 (12.3 to 20.8) | -14.9 (-36.6 to 20.4) | 23 (17.1 to 29.4) | 19.7 (15.2 to 26.4) | -14.5 (-36.6 to 26.8) | 14.8 (11.4 to 18.9) | 13.7 (10.2 to 18.1) | -7.6 (-36.7 to 34.6) |
|  | **DALYs** | All ages  (number) | 41771 (33397 to 51291) | 108820 (81226 to 142143) | 160.5 (117.7 to 203.9) | 22204 (17782 to 27454) | 55220 (41558 to 72787) | 148.7 (103.2 to 201.7) | 19567 (15410 to 24364) | 53601 (40041 to 69758) | 173.9 (125.2 to 226.5) |
|  |  | All ages  (rate per 100,000) | 323.9 (259 to 397.8) | 750.9 (560.5 to 980.9) | 131.8 (93.7 to 170.4) | 352.5 (282.3 to 435.9) | 742.9 (559.1 to 979.2) | 110.7 (72.2 to 155.7) | 296.6 (233.6 to 369.4) | 759.4 (567.3 to 988.4) | 156 (110.4 to 205.1) |
|  |  | Age-standardized (rate per 100,000) | 740.1 (594.8 to 903) | 853.2 (646.5 to 1103.2) | 15.3 (-3.1 to 34.8) | 824.3 (662.9 to 1013.6) | 898.2 (684.1 to 1169.5) | 9 (-10.4 to 32.1) | 663.9 (525.4 to 825.2) | 820.1 (615.5 to 1057.3) | 23.5 (2.5 to 46.6) |
|  | **YLLs** | All ages  (number) | 21193 (16587 to 26232) | 35718 (26861 to 48419) | 68.5 (20.2 to 144.1) | 12076 (9206 to 15503) | 18697 (13843 to 25616) | 54.8 (10 to 130.4) | 9117 (7057 to 11580) | 17020 (12260 to 23157) | 86.7 (23.4 to 178.7) |
|  |  | All ages  (rate per 100,000) | 164.3 (128.6 to 203.4) | 246.5 (185.4 to 334.1) | 50 (6.9 to 117.2) | 191.7 (146.2 to 246.1) | 251.5 (186.2 to 344.6) | 31.2 (-6.8 to 95.2) | 138.2 (107 to 175.6) | 241.2 (173.7 to 328.1) | 74.5 (15.3 to 160.5) |
|  |  | Age-standardized (rate per 100,000) | 383.3 (299.9 to 480) | 295.8 (224.7 to 395.5) | -22.8 (-44.3 to 10.4) | 460 (346.6 to 587.6) | 335.3 (254.8 to 450.5) | -27.1 (-47 to 6.9) | 314.2 (242.3 to 401.2) | 267.9 (196.5 to 359.2) | -14.7 (-42.8 to 26.6) |
|  | **YLDs** | All ages  (number) | 20578 (13624 to 28911) | 73103 (48153 to 102576) | 255.2 (231.2 to 280.4) | 10128 (6639 to 14432) | 36523 (23885 to 51729) | 260.6 (230.5 to 296.2) | 10450 (6743 to 14698) | 36580 (23757 to 51836) | 250.1 (219.5 to 284.6) |
|  |  | All ages  (rate per 100,000) | 159.6 (105.7 to 224.2) | 504.5 (332.3 to 707.8) | 216.1 (194.8 to 238.5) | 160.8 (105.4 to 229.1) | 491.3 (321.3 to 695.9) | 205.6 (180.1 to 235.8) | 158.4 (102.2 to 222.8) | 518.3 (336.6 to 734.4) | 227.1 (198.6 to 259.4) |
|  |  | Age-standardized (rate per 100,000) | 356.8 (238.1 to 502.3) | 557.4 (368.6 to 780.6) | 56.2 (46.8 to 67.3) | 364.3 (239.1 to 506.2) | 562.9 (372.9 to 801.2) | 54.5 (42.2 to 69.6) | 349.8 (227 to 489) | 552.2 (358.9 to 783.3) | 57.9 (45 to 73) |
| **Tunisia** | **Incidence** | All ages  (number) | 13747 (12477 to 15114) | 55022 (49389 to 60980) | 300.2 (280 to 322.1) | 5927 (5318 to 6592) | 24546 (21953 to 27381) | 314.1 (286.5 to 344.4) | 7820 (7075 to 8648) | 30475 (27184 to 33952) | 289.7 (261.9 to 319.3) |
|  |  | All ages  (rate per 100,000) | 162.9 (147.8 to 179.1) | 475.5 (426.8 to 527) | 191.9 (177.1 to 207.9) | 142.5 (127.8 to 158.4) | 422.6 (377.9 to 471.4) | 196.6 (176.8 to 218.3) | 182.8 (165.3 to 202.1) | 528.8 (471.7 to 589.2) | 189.4 (168.7 to 211.3) |
|  |  | Age-standardized (rate per 100,000) | 226.1 (205.3 to 249.6) | 408.6 (369.9 to 451.9) | 80.7 (72.5 to 89.5) | 197.3 (176.6 to 219.7) | 360.1 (322.9 to 400.7) | 82.5 (70.2 to 94.7) | 254.5 (230.2 to 282.1) | 458.4 (411.8 to 508.7) | 80.1 (67.6 to 93.2) |
|  | **Prevalence** | All ages  (number) | 234482 (210287 to 259720) | 1061155 (950830 to 1181080) | 352.6 (330.8 to 376) | 98798 (87486 to 110371) | 472890 (419962 to 527155) | 378.6 (346.4 to 412.6) | 135684 (121360 to 152053) | 588264 (522100 to 654829) | 333.6 (302.9 to 366.6) |
|  |  | All ages  (rate per 100,000) | 2778.5 (2491.8 to 3077.5) | 9170.3 (8216.9 to 10206.7) | 230.1 (214.2 to 247.2) | 2374.5 (2102.7 to 2652.7) | 8140.8 (7229.7 to 9075) | 242.8 (219.7 to 267.2) | 3171.2 (2836.4 to 3553.8) | 10208.1 (9060 to 11363.2) | 221.9 (199.1 to 246.5) |
|  |  | Age-standardized (rate per 100,000) | 4352.6 (3907.5 to 4816.6) | 8162.2 (7324.5 to 9059.8) | 87.5 (78.5 to 97.1) | 3758.3 (3334.8 to 4202.3) | 7132 (6318.8 to 7951.1) | 89.8 (76.7 to 102.8) | 4919.7 (4434.8 to 5506.8) | 9239.5 (8228.7 to 10271.1) | 87.8 (74.2 to 101.8) |
|  | **Deaths** | All ages  (number) | 459 (371 to 616) | 1722 (1237 to 2322) | 274.9 (172.6 to 415.2) | 238 (182 to 406) | 860 (615 to 1218) | 260.9 (156.5 to 419.6) | 221 (170 to 279) | 863 (592 to 1237) | 290 (169.3 to 461.8) |
|  |  | All ages  (rate per 100,000) | 5.4 (4.4 to 7.3) | 14.9 (10.7 to 20.1) | 173.4 (98.8 to 275.7) | 5.7 (4.4 to 9.7) | 14.8 (10.6 to 21) | 158.5 (83.7 to 272.2) | 5.2 (4 to 6.5) | 15 (10.3 to 21.5) | 189.5 (100 to 317.1) |
|  |  | Age-standardized (rate per 100,000) | 10.9 (8.7 to 15) | 14.8 (10.7 to 19.9) | 36.1 (-0.8 to 85.4) | 11.6 (8.9 to 20.1) | 14.1 (10.1 to 20.2) | 21.7 (-13.1 to 73.4) | 10.3 (8 to 12.8) | 15.6 (10.9 to 22.1) | 52.3 (5.8 to 117.6) |
|  | **DALYs** | All ages  (number) | 29009 (22160 to 37306) | 120897 (90701 to 157871) | 316.8 (270.3 to 364.8) | 13287 (10275 to 17638) | 55751 (41695 to 73569) | 319.6 (263.7 to 375.6) | 15723 (11740 to 20333) | 65146 (48071 to 85082) | 314.3 (267.6 to 371.2) |
|  |  | All ages  (rate per 100,000) | 343.7 (262.6 to 442) | 1044.8 (783.8 to 1364.3) | 203.9 (170 to 238.9) | 319.3 (247 to 423.9) | 959.8 (717.8 to 1266.5) | 200.6 (160.5 to 240.7) | 367.5 (274.4 to 475.2) | 1130.5 (834.2 to 1476.4) | 207.6 (172.9 to 249.9) |
|  |  | Age-standardized (rate per 100,000) | 564 (434.2 to 720.3) | 943.8 (713.8 to 1233.8) | 67.4 (48 to 86.7) | 533 (411.3 to 704) | 851.5 (635.9 to 1121.7) | 59.8 (37.7 to 82.8) | 593.8 (446.6 to 761.7) | 1040.9 (771.3 to 1355.6) | 75.3 (54.9 to 98.9) |
|  | **YLLs** | All ages  (number) | 9915 (8040 to 12889) | 34284 (24502 to 46780) | 245.8 (142.6 to 379.9) | 5206 (4047 to 8451) | 16758 (11916 to 23780) | 221.9 (121.6 to 367.9) | 4709 (3661 to 5891) | 17527 (11930 to 25609) | 272.2 (149.8 to 443.2) |
|  |  | All ages  (rate per 100,000) | 117.5 (95.3 to 152.7) | 296.3 (211.7 to 404.3) | 152.2 (77 to 250) | 125.1 (97.3 to 203.1) | 288.5 (205.1 to 409.4) | 130.6 (58.7 to 235.2) | 110.1 (85.6 to 137.7) | 304.1 (207 to 444.4) | 176.3 (85.4 to 303.3) |
|  |  | Age-standardized (rate per 100,000) | 202.3 (163.8 to 269.4) | 274.7 (196.9 to 370.4) | 35.8 (-3.8 to 87.5) | 218.8 (169.3 to 367.2) | 261.4 (186.7 to 375) | 19.5 (-16.9 to 73.7) | 187.3 (145.2 to 232.9) | 289.1 (198.1 to 418.2) | 54.4 (5.7 to 123.3) |
|  | **YLDs** | All ages  (number) | 19094 (12640 to 27012) | 86613 (56568 to 122195) | 353.6 (331 to 378.3) | 8081 (5385 to 11535) | 38994 (25269 to 55396) | 382.6 (349.4 to 418.2) | 11013 (7234 to 15573) | 47619 (31256 to 67295) | 332.4 (300.2 to 365.2) |
|  |  | All ages  (rate per 100,000) | 226.3 (149.8 to 320.1) | 748.5 (488.9 to 1056) | 230.8 (214.4 to 248.9) | 194.2 (129.4 to 277.2) | 671.3 (435 to 953.6) | 245.6 (221.9 to 271.2) | 257.4 (169.1 to 364) | 826.3 (542.4 to 1167.8) | 221 (197.1 to 245.4) |
|  |  | Age-standardized (rate per 100,000) | 361.7 (240.3 to 507.3) | 669.1 (438.8 to 939.6) | 85 (75.9 to 94.6) | 314.2 (209.7 to 446.5) | 590.1 (382.3 to 837.9) | 87.8 (74.9 to 101.3) | 406.5 (266.2 to 573.4) | 751.8 (493.7 to 1053.6) | 85 (70.9 to 98.8) |
| **Turkey** | **Incidence** | All ages  (number) | 88857 (82308 to 96372) | 258309 (234932 to 281495) | 190.7 (165.6 to 216.9) | 46543 (43178 to 50412) | 127990 (115658 to 141466) | 175 (147.6 to 204.8) | 42313 (38613 to 46429) | 130319 (116382 to 144414) | 208 (175.5 to 243.6) |
|  |  | All ages  (rate per 100,000) | 148.7 (137.7 to 161.2) | 317.5 (288.8 to 346) | 113.6 (95.1 to 132.8) | 158 (146.5 to 171.1) | 317.9 (287.3 to 351.4) | 101.3 (81.2 to 123.1) | 139.6 (127.4 to 153.2) | 317.1 (283.1 to 351.3) | 127.1 (103.1 to 153.4) |
|  |  | Age-standardized (rate per 100,000) | 202.5 (188.6 to 219.4) | 273.8 (248.8 to 298.6) | 35.2 (23.7 to 47.2) | 211.6 (197.4 to 228.8) | 269.1 (243.4 to 296.4) | 27.2 (15 to 41.1) | 192.7 (175.9 to 211.3) | 277.9 (249.3 to 307.9) | 44.2 (28.8 to 60.1) |
|  | **Prevalence** | All ages  (number) | 1396535 (1276620 to 1529149) | 4614950 (4151327 to 5055521) | 230.5 (202.4 to 258.7) | 742456 (679512 to 812093) | 2339542 (2091272 to 2593970) | 215.1 (183.8 to 247.9) | 654079 (585269 to 723127) | 2275408 (2028292 to 2527926) | 247.9 (210.4 to 288.4) |
|  |  | All ages  (rate per 100,000) | 2336.4 (2135.8 to 2558.3) | 5672.3 (5102.4 to 6213.8) | 142.8 (122.2 to 163.6) | 2519.9 (2306.3 to 2756.3) | 5811.6 (5194.9 to 6443.6) | 130.6 (107.7 to 154.7) | 2158 (1931 to 2385.8) | 5535.8 (4934.6 to 6150.2) | 156.5 (128.9 to 186.4) |
|  |  | Age-standardized (rate per 100,000) | 3582.8 (3284.4 to 3912.6) | 5082 (4584.6 to 5564.4) | 41.8 (29.5 to 54.5) | 3712.4 (3411.3 to 4047.7) | 4963.9 (4432.4 to 5497) | 33.7 (20.2 to 47.7) | 3443.5 (3091.4 to 3797.2) | 5204.3 (4648.1 to 5783.6) | 51.1 (34.7 to 68.8) |
|  | **Deaths** | All ages  (number) | 12931 (11086 to 14894) | 18831 (15145 to 22949) | 45.6 (12.6 to 89.9) | 7842 (6011 to 9298) | 10882 (8620 to 13506) | 38.8 (4.9 to 92.5) | 5089 (4034 to 6426) | 7949 (6301 to 9936) | 56.2 (11.4 to 119.4) |
|  |  | All ages  (rate per 100,000) | 21.6 (18.5 to 24.9) | 23.1 (18.6 to 28.2) | 7 (-17.3 to 39.5) | 26.6 (20.4 to 31.6) | 27 (21.4 to 33.5) | 1.6 (-23.2 to 40.9) | 16.8 (13.3 to 21.2) | 19.3 (15.3 to 24.2) | 15.2 (-17.8 to 61.8) |
|  |  | Age-standardized (rate per 100,000) | 40.3 (34.6 to 47) | 22.7 (18.3 to 27.6) | -43.7 (-56.3 to -26.7) | 46.2 (35.4 to 55.2) | 24 (19 to 29.9) | -47.9 (-60.9 to -28.2) | 33 (26.1 to 41.6) | 20.8 (16.5 to 25.9) | -37 (-55.5 to -12.5) |
|  | **DALYs** | All ages  (number) | 413748 (351292 to 484046) | 770690 (619599 to 956089) | 86.3 (55.1 to 120.9) | 234627 (189716 to 277537) | 407252 (326495 to 505329) | 73.6 (42.1 to 117.6) | 179122 (146628 to 220333) | 363438 (284840 to 453303) | 102.9 (57.3 to 152.9) |
|  |  | All ages  (rate per 100,000) | 692.2 (587.7 to 809.8) | 947.3 (761.6 to 1175.1) | 36.8 (14 to 62.3) | 796.3 (643.9 to 942) | 1011.6 (811 to 1255.3) | 27 (4 to 59.3) | 591 (483.8 to 727) | 884.2 (693 to 1102.8) | 49.6 (16 to 86.5) |
|  |  | Age-standardized (rate per 100,000) | 1141.6 (976.1 to 1333.6) | 869.9 (702.3 to 1073.5) | -23.8 (-36.5 to -9.4) | 1253.4 (1016.8 to 1487.1) | 873.2 (700.6 to 1082.4) | -30.3 (-43.2 to -13.1) | 1011.1 (831 to 1239.9) | 860.2 (678.3 to 1066.5) | -14.9 (-33.5 to 5.8) |
|  | **YLLs** | All ages  (number) | 288081 (246005 to 333461) | 350513 (278647 to 432470) | 21.7 (-7.6 to 62.2) | 165722 (125790 to 197206) | 187014 (150172 to 229799) | 12.8 (-15.5 to 60.4) | 122359 (96287 to 154198) | 163498 (128429 to 205998) | 33.6 (-7.5 to 90.8) |
|  |  | All ages  (rate per 100,000) | 482 (411.6 to 557.9) | 430.8 (342.5 to 531.6) | -10.6 (-32.1 to 19.1) | 562.5 (426.9 to 669.3) | 464.6 (373 to 570.8) | -17.4 (-38.1 to 17.4) | 403.7 (317.7 to 508.8) | 397.8 (312.5 to 501.2) | -1.5 (-31.8 to 40.7) |
|  |  | Age-standardized (rate per 100,000) | 810.8 (690.5 to 935.3) | 404.5 (322.6 to 497.2) | -50.1 (-61.8 to -34.1) | 902.4 (688.5 to 1072) | 405.6 (325 to 498.7) | -55.1 (-66.4 to -36.6) | 701.5 (554.6 to 885.1) | 396.9 (312 to 498.7) | -43.4 (-60.5 to -19.8) |
|  | **YLDs** | All ages  (number) | 125667 (82701 to 176298) | 420178 (274783 to 588274) | 234.4 (203.7 to 265.2) | 68904 (45730 to 96428) | 220238 (145816 to 307930) | 219.6 (186.4 to 254.9) | 56763 (37429 to 80458) | 199940 (129300 to 286143) | 252.2 (211.9 to 296.6) |
|  |  | All ages  (rate per 100,000) | 210.2 (138.4 to 294.9) | 516.4 (337.7 to 723.1) | 145.6 (123.1 to 168.3) | 233.9 (155.2 to 327.3) | 547.1 (362.2 to 764.9) | 133.9 (109.6 to 159.8) | 187.3 (123.5 to 265.5) | 486.4 (314.6 to 696.2) | 159.7 (130 to 192.4) |
|  |  | Age-standardized (rate per 100,000) | 330.8 (218.2 to 464.1) | 465.4 (304.9 to 649) | 40.7 (27.8 to 53.8) | 351 (233.5 to 488.5) | 467.5 (310.3 to 654.6) | 33.2 (19.2 to 48.1) | 309.5 (203.8 to 435.4) | 463.3 (299.6 to 659.5) | 49.7 (32.7 to 68) |
| **United Arab Emirates** | **Incidence** | All ages  (number) | 4008 (3561 to 4555) | 63860 (55884 to 73094) | 1493.3 (1365.1 to 1631.7) | 1093 (970 to 1223) | 13587 (12062 to 15462) | 1142.8 (1032.6 to 1261.2) | 2915 (2549 to 3336) | 50274 (43424 to 58304) | 1624.8 (1453.8 to 1799.6) |
|  |  | All ages  (rate per 100,000) | 214.1 (190.2 to 243.3) | 691 (604.7 to 790.9) | 222.7 (196.8 to 250.8) | 165.5 (146.8 to 185.1) | 537 (476.7 to 611.1) | 224.5 (195.8 to 255.5) | 240.6 (210.4 to 275.4) | 749.1 (647 to 868.7) | 211.3 (180.4 to 242.9) |
|  |  | Age-standardized (rate per 100,000) | 397.2 (364.7 to 431.8) | 589.9 (546 to 641.8) | 48.5 (40.5 to 57.7) | 419.1 (380.5 to 456.5) | 601.9 (556.1 to 653.7) | 43.6 (33.4 to 54.7) | 383.8 (352.5 to 421.1) | 583.4 (537 to 638.6) | 52 (41.9 to 63.1) |
|  | **Prevalence** | All ages  (number) | 43887 (38439 to 49682) | 719240 (625472 to 821821) | 1538.8 (1432.8 to 1652.1) | 13211 (11584 to 15002) | 160978 (141434 to 184459) | 1118.5 (1021.4 to 1225.1) | 30677 (26733 to 34924) | 558262 (480768 to 643700) | 1719.8 (1573.1 to 1872.2) |
|  |  | All ages  (rate per 100,000) | 2344.3 (2053.3 to 2653.8) | 7782.5 (6767.9 to 8892.5) | 232 (210.5 to 254.9) | 1999.5 (1753.3 to 2270.6) | 6362.5 (5590 to 7290.6) | 218.2 (192.8 to 246) | 2532.4 (2206.8 to 2883) | 8317.9 (7163.2 to 9590.9) | 228.5 (202 to 256) |
|  |  | Age-standardized (rate per 100,000) | 6855.8 (6171.6 to 7585.2) | 11098.2 (10089 to 12249.4) | 61.9 (52.2 to 72.8) | 7156.6 (6328.6 to 7956.8) | 11175.7 (10171.8 to 12325.5) | 56.2 (43.7 to 70.1) | 6650.4 (5979 to 7414) | 11028.1 (9980.5 to 12236.8) | 65.8 (53.4 to 78.8) |
|  | **Deaths** | All ages  (number) | 175 (137 to 217) | 1232 (869 to 1662) | 603.8 (338.1 to 941.5) | 69 (50 to 91) | 246 (182 to 329) | 254 (126.9 to 454.4) | 106 (79 to 140) | 986 (660 to 1380) | 833.5 (409.4 to 1382.4) |
|  |  | All ages  (rate per 100,000) | 9.4 (7.3 to 11.6) | 13.3 (9.4 to 18) | 42.6 (-11.3 to 111) | 10.5 (7.6 to 13.8) | 9.7 (7.2 to 13) | -7.5 (-40.7 to 44.8) | 8.7 (6.6 to 11.6) | 14.7 (9.8 to 20.6) | 68.5 (-8.1 to 167.6) |
|  |  | Age-standardized (rate per 100,000) | 75.3 (58.3 to 92.7) | 55.2 (41.4 to 70.7) | -26.7 (-51.7 to 3.3) | 85.7 (58.3 to 114.4) | 51.2 (38 to 67.5) | -40.2 (-60.2 to -10.2) | 66.9 (50.6 to 86.4) | 57.1 (40.4 to 75.3) | -14.7 (-50.4 to 27.5) |
|  | **DALYs** | All ages  (number) | 7697 (6225 to 9332) | 87940 (66705 to 113103) | 1042.5 (791.7 to 1327.6) | 2616 (2054 to 3244) | 18389 (13988 to 24105) | 603 (440.3 to 801.6) | 5081 (3987 to 6415) | 69551 (51959 to 89709) | 1268.8 (907.8 to 1652.2) |
|  |  | All ages  (rate per 100,000) | 411.2 (332.5 to 498.5) | 951.6 (721.8 to 1223.8) | 131.4 (80.6 to 189.2) | 395.9 (310.9 to 490.9) | 726.8 (552.9 to 952.7) | 83.6 (41.1 to 135.4) | 419.5 (329.2 to 529.6) | 1036.3 (774.2 to 1336.6) | 147 (81.9 to 216.3) |
|  |  | Age-standardized (rate per 100,000) | 1884.2 (1558.7 to 2246.7) | 1865.2 (1468 to 2332.4) | -1 (-23.8 to 23.8) | 2032.8 (1571.4 to 2527.7) | 1741.3 (1374.7 to 2211) | -14.3 (-35.2 to 12.1) | 1765.2 (1406.6 to 2187.7) | 1909.7 (1484.3 to 2404) | 8.2 (-22.2 to 41.5) |
|  | **YLLs** | All ages  (number) | 4610 (3612 to 5784) | 37614 (25997 to 51771) | 715.8 (391.9 to 1144.7) | 1658 (1214 to 2169) | 6888 (4998 to 9209) | 315.3 (164.1 to 565.4) | 2952 (2166 to 3950) | 30726 (20141 to 43518) | 940.9 (470.8 to 1587) |
|  |  | All ages  (rate per 100,000) | 246.3 (192.9 to 309) | 407 (281.3 to 560.2) | 65.3 (-0.3 to 152.1) | 251 (183.8 to 328.3) | 272.2 (197.5 to 364) | 8.5 (-31 to 73.8) | 243.7 (178.8 to 326.1) | 457.8 (300.1 to 648.4) | 87.9 (3 to 204.5) |
|  |  | Age-standardized (rate per 100,000) | 1347.5 (1058.2 to 1645.9) | 1011.5 (745.8 to 1316.9) | -24.9 (-51.1 to 7.6) | 1473.5 (1047.5 to 1934.7) | 880.7 (657.8 to 1156.8) | -40.2 (-60.8 to -9.6) | 1242.5 (946.6 to 1614) | 1060.5 (741.4 to 1429.3) | -14.6 (-50.2 to 30.2) |
|  | **YLDs** | All ages  (number) | 3087 (2051 to 4370) | 50326 (32210 to 71895) | 1530.4 (1417.2 to 1649.3) | 957 (630 to 1353) | 11501 (7544 to 16525) | 1101.2 (1000.1 to 1220.1) | 2129 (1405 to 3041) | 38825 (24946 to 55595) | 1723.3 (1572.1 to 1882.1) |
|  |  | All ages  (rate per 100,000) | 164.9 (109.5 to 233.5) | 544.6 (348.5 to 777.9) | 230.3 (207.3 to 254.3) | 144.9 (95.3 to 204.7) | 454.6 (298.2 to 653.2) | 213.7 (187.3 to 244.7) | 175.8 (116 to 251) | 578.5 (371.7 to 828.3) | 229.1 (201.8 to 257.7) |
|  |  | Age-standardized (rate per 100,000) | 536.7 (357.8 to 749) | 853.7 (570.4 to 1189.5) | 59.1 (49 to 70.3) | 559.3 (371.1 to 791.7) | 860.6 (570.1 to 1207.8) | 53.9 (41.6 to 67.6) | 522.7 (349.3 to 727.1) | 849.2 (565.6 to 1188.9) | 62.5 (50.4 to 75.8) |
| **Yemen** | **Incidence** | All ages  (number) | 10045 (9007 to 11156) | 48534 (43651 to 54069) | 383.2 (360.1 to 408.4) | 4959 (4435 to 5526) | 24808 (22213 to 27686) | 400.3 (369.3 to 435) | 5086 (4509 to 5680) | 23725 (21097 to 26588) | 366.5 (334.3 to 403.3) |
|  |  | All ages  (rate per 100,000) | 73.2 (65.6 to 81.3) | 154.1 (138.6 to 171.6) | 110.6 (100.5 to 121.6) | 73.6 (65.9 to 82.1) | 159.3 (142.6 to 177.8) | 116.3 (103 to 131.4) | 72.7 (64.5 to 81.2) | 148.9 (132.4 to 166.9) | 104.8 (90.7 to 121) |
|  |  | Age-standardized (rate per 100,000) | 145.9 (131.5 to 161.8) | 241.7 (217.4 to 268.6) | 65.7 (57.3 to 75.3) | 145 (129.2 to 160.5) | 246.7 (221.5 to 274.5) | 70.1 (59 to 82.5) | 146 (130.8 to 163.4) | 236.6 (210.4 to 266.7) | 62.1 (50.8 to 75.1) |
|  | **Prevalence** | All ages  (number) | 153005 (135510 to 172394) | 739598 (652478 to 833372) | 383.4 (358.6 to 409.9) | 76819 (67551 to 86636) | 378557 (333929 to 427794) | 392.8 (360.6 to 429.8) | 76186 (67096 to 86230) | 361041 (315270 to 408858) | 373.9 (340.5 to 412.9) |
|  |  | All ages  (rate per 100,000) | 1114.5 (987 to 1255.7) | 2347.7 (2071.2 to 2645.4) | 110.7 (99.9 to 122.2) | 1140.6 (1003 to 1286.4) | 2430.6 (2144.1 to 2746.8) | 113.1 (99.2 to 129.1) | 1089.3 (959.3 to 1232.9) | 2266.6 (1979.3 to 2566.8) | 108.1 (93.4 to 125.2) |
|  |  | Age-standardized (rate per 100,000) | 2720.2 (2418.6 to 3042) | 4686.9 (4150.6 to 5245.5) | 72.3 (63.6 to 82) | 2690.7 (2378.5 to 3028.3) | 4760.7 (4223.4 to 5376.9) | 76.9 (64.9 to 90.2) | 2754.7 (2432.8 to 3103.3) | 4611.5 (4068.9 to 5223.1) | 67.4 (55.6 to 81.2) |
|  | **Deaths** | All ages  (number) | 485 (333 to 720) | 1620 (1138 to 2302) | 234.1 (146.9 to 353.2) | 289 (181 to 491) | 970 (619 to 1454) | 236 (137.8 to 370.2) | 196 (135 to 288) | 650 (449 to 920) | 231.3 (135.7 to 375.4) |
|  |  | All ages  (rate per 100,000) | 3.5 (2.4 to 5.2) | 5.1 (3.6 to 7.3) | 45.6 (7.6 to 97.5) | 4.3 (2.7 to 7.3) | 6.2 (4 to 9.3) | 45.3 (2.8 to 103.3) | 2.8 (1.9 to 4.1) | 4.1 (2.8 to 5.8) | 45.5 (3.5 to 108.8) |
|  |  | Age-standardized (rate per 100,000) | 11.7 (8.1 to 17.9) | 14.2 (10.2 to 19.8) | 21.6 (-9.7 to 62.6) | 12.8 (8.2 to 21.6) | 16.5 (10.8 to 24.4) | 29.1 (-7.3 to 77.8) | 10.3 (7.4 to 14.5) | 11.7 (8.3 to 16.2) | 13.9 (-16.9 to 57.2) |
|  | **DALYs** | All ages  (number) | 24099 (18086 to 32145) | 96676 (72068 to 127175) | 301.2 (236.3 to 368.4) | 13052 (9460 to 18135) | 53220 (38265 to 71501) | 307.8 (228.6 to 386.5) | 11047 (8146 to 15180) | 43456 (31684 to 57313) | 293.4 (224.5 to 365.5) |
|  |  | All ages  (rate per 100,000) | 175.5 (131.7 to 234.1) | 306.9 (228.8 to 403.7) | 74.8 (46.5 to 104.1) | 193.8 (140.5 to 269.3) | 341.7 (245.7 to 459.1) | 76.3 (42.1 to 110.4) | 157.9 (116.5 to 217) | 272.8 (198.9 to 359.8) | 72.7 (42.5 to 104.4) |
|  |  | Age-standardized (rate per 100,000) | 467.3 (354.5 to 626.4) | 676.5 (512.3 to 878.7) | 44.8 (21.8 to 68.9) | 494.8 (357.2 to 695.5) | 736.4 (535.4 to 978.6) | 48.8 (19.4 to 77.3) | 439.1 (330.6 to 584.1) | 614.9 (453.8 to 807.5) | 40 (17 to 64.7) |
|  | **YLLs** | All ages  (number) | 12116 (8028 to 17952) | 39206 (26702 to 57107) | 223.6 (136.3 to 348.3) | 6982 (4252 to 11460) | 23570 (14515 to 35631) | 237.6 (130.1 to 386.4) | 5134 (3439 to 7715) | 15635 (10578 to 22941) | 204.6 (113.9 to 344.6) |
|  |  | All ages  (rate per 100,000) | 88.2 (58.5 to 130.8) | 124.5 (84.8 to 181.3) | 41 (3 to 95.4) | 103.7 (63.1 to 170.2) | 151.3 (93.2 to 228.8) | 46 (-0.5 to 110.3) | 73.4 (49.2 to 110.3) | 98.2 (66.4 to 144) | 33.7 (-6.1 to 95.2) |
|  |  | Age-standardized (rate per 100,000) | 244.5 (166.1 to 362.4) | 293.6 (204.5 to 420.5) | 20.1 (-12.2 to 63.3) | 273.8 (170.5 to 455) | 346.3 (216.7 to 522.9) | 26.5 (-10.5 to 79) | 213.6 (149.2 to 313.3) | 239.4 (165.5 to 342.6) | 12.1 (-19.6 to 60.6) |
|  | **YLDs** | All ages  (number) | 11983 (7936 to 17032) | 57470 (37432 to 80652) | 379.6 (354.4 to 408.5) | 6070 (3960 to 8602) | 29650 (19042 to 41898) | 388.5 (353.7 to 425.9) | 5913 (3866 to 8514) | 27820 (18471 to 39524) | 370.5 (334.3 to 414.2) |
|  |  | All ages  (rate per 100,000) | 87.3 (57.8 to 124.1) | 182.4 (118.8 to 256) | 109 (98 to 121.6) | 90.1 (58.8 to 127.7) | 190.4 (122.3 to 269) | 111.2 (96.2 to 127.4) | 84.5 (55.3 to 121.7) | 174.7 (116 to 248.1) | 106.6 (90.7 to 125.8) |
|  |  | Age-standardized (rate per 100,000) | 222.8 (148.3 to 316.6) | 382.9 (253.7 to 536.5) | 71.9 (62.9 to 82.3) | 221 (145.2 to 310.6) | 390.1 (254 to 541.7) | 76.5 (63.9 to 90.4) | 225.5 (148.9 to 324.2) | 375.5 (248.6 to 536.8) | 66.5 (53.7 to 81.3) |

Data in parentheses are 95% uncertainty interval (UI); DALYs= Disability-Adjusted Life Years; YLLs= Years of Life Lost; YLDs= Years Lived with Disability
