# Supplementary material for: Burden of type 2 diabetes mellitus and its risk factors in North Africa and the Middle East, 1990–2019: findings from the Global Burden of Disease study 2019
Source: BMC Public Health. 2024 Jan 5;24:98. doi: 10.1186/s12889-023-16540-8 (PMC10768242; doi:10.1186/s12889-023-16540-8)
Supplement: Supplementary file 3 — Additional file 3: Supplementary Table 3. Burden and ranking of region countries based on the age-standardized YLLs and YLDs rates in 1990 and 2019 with percent change. [file 12889_2023_16540_MOESM3_ESM.docx]

**YLLs**

| **Country** | **Both** | | | | | **Female** | | | | | **Male** | | | | |
| --- | --- | --- | --- | --- | --- | --- | --- | --- | --- | --- | --- | --- | --- | --- | --- |
|  | **1990** | | **2019** | | **% Change (1990 to 2019)** | **1990** |  | **2019** | | **% Change (1990 to 2019)** | **1990** | | **2019** | | **% Change (1990 to 2019)** |
|  | **ASR (per 100,000)** | **Ranking** | **ASR (per 100,000)** | **Ranking** |  | **ASR (per 100,000)** | **Ranking** | **ASR (per 100,000)** | **Ranking** |  | **ASR (per 100,000)** | **Ranking** | **ASR (per 100,000)** | **Ranking** |  |
| **Afghanistan** | 638.04 (406.48 to 955.73) | 9 | 887.52 (512.15 to 1308.87) | 7  **↑** | 39.1 (-1.5 to 86.4) | 888.54 (495.38 to 1430.26) | 9 | 1286.82 (651.09 to 2001.95) | 3  **↑** | 44.8 (-1.7 to 99.4) | 398.77 (273.1 to 580.71) | 13 | 449.91 (306.59 to 641.51) | 9  **↑** | 12.8 (-20.1 to 51.7) |
| **Algeria** | 305.1 (220.83 to 420.42) | 15 | 318.92 (244.76 to 411.25) | 15  ***** | 4.5 (-22.2 to 42.4) | 358.09 (235.73 to 535) | 14 | 387.72 (288.61 to 545.47) | 14  ***** | 8.3 (-21.8 to 55.8) | 256.56 (186.88 to 354.94) | 15 | 259.4 (187.61 to 336.73) | 20  **↓** | 1.1 (-30.7 to 44.2) |
| **Bahrain** | 1421.32 (1202.05 to 1671.73) | 2 | 2032.29 (1632.39 to 2519.81) | 1  **↑** | 43 (8.4 to 81.3) | 1350.58 (1109.69 to 1707.97) | 4 | 2052.82 (1594.18 to 2487.84) | 2  **↑** | 52 (-5.2 to 99.1) | 1490.35 (1210.58 to 1842.77) | 2 | 2015.32 (1586.05 to 2577.98) | 1  **↑** | 35.2 (3 to 78.4) |
| **Egypt** | 504.65 (467.02 to 546.41) | 12 | 729.89 (547.35 to 956.12) | 8  **↑** | 44.6 (8.3 to 89.7) | 565.85 (511.37 to 630.46) | 11 | 827.25 (609.68 to 1159.63) | 8  **↑** | 46.2 (8.2 to 107.8) | 443.19 (384.97 to 493.46) | 11 | 668.86 (483.96 to 897.78) | 8  **↑** | 50.9 (10.2 to 104.6) |
| **Iran (Islamic Republic of)** | 228.4 (199.64 to 261.66) | 20 | 402.95 (334.12 to 433.94) | 12  **↑** | 76.4 (38.1 to 107.3) | 251.39 (203.01 to 304.11) | 20 | 426.59 (291.59 to 473.44) | 11  **↑** | 69.7 (3.5 to 124.9) | 204.84 (173.46 to 243.57) | 20 | 379.64 (345.64 to 417.22) | 13  **↑** | 85.3 (49.7 to 124.9) |
| **Iraq** | 1089.95 (882.66 to 1305.52) | 5 | 928.67 (734.96 to 1133.23) | 6  **↓** | -14.8 (-35.9 to 11.9) | 1163.06 (903.83 to 1432.79) | 5 | 873.74 (682.57 to 1111.03) | 7  **↓** | -24.9 (-45.8 to 4.7) | 1014.95 (786.43 to 1275.47) | 4 | 984.81 (755.14 to 1200.04) | 6  **↓** | -3 (-29.1 to 30.9) |
| **Jordan** | 1211.35 (1022.55 to 1424.05) | 4 | 705.68 (596.19 to 843.17) | 9  **↓** | -41.7 (-54.5 to -25.4) | 1502.95 (1179.86 to 1831.67) | 2 | 695.74 (555.68 to 858.62) | 9  **↓** | -53.7 (-65 to -36.7) | 920.36 (727.69 to 1155.92) | 6 | 714.26 (555.72 to 904.34) | 7  **↓** | -22.4 (-44.7 to 10.7) |
| **Kuwait** | 519.48 (470.04 to 580.29) | 10 | 301.33 (250.67 to 364.1) | 17  **↓** | -42 (-50.7 to -31) | 613.48 (534.58 to 689.76) | 10 | 266.47 (211.4 to 331.77) | 19  **↓** | -56.6 (-65.3 to -46.5) | 453.15 (399.42 to 530.73) | 10 | 323.9 (251.5 to 413.82) | 16  **↓** | -28.5 (-43.8 to -11.3) |
| **Lebanon** | 392.37 (338.85 to 470.02) | 13 | 293.38 (212.64 to 377.29) | 20  **↓** | -25.2 (-46.9 to -0.9) | 347.97 (285.65 to 422.2) | 15 | 217.01 (161 to 299.66) | 21  **↓** | -37.6 (-54.4 to -11.6) | 438.3 (364.14 to 537.73) | 12 | 386.32 (239.08 to 542.5) | 12  ***** | -11.9 (-45.9 to 26.2) |
| **Libya** | 286.5 (211.73 to 359.48) | 16 | 373.64 (271 to 494.77) | 14  **↑** | 30.4 (-10.1 to 88) | 321.71 (241.41 to 444.22) | 16 | 405.98 (289.89 to 545.65) | 12  **↑** | 26.2 (-13.7 to 83.2) | 255.93 (176.13 to 342.57) | 16 | 342.18 (217.09 to 505.55) | 15  **↑** | 33.7 (-17.2 to 102.3) |
| **Morocco** | 264.1 (212.6 to 362.39) | 17 | 443.21 (333.4 to 555.19) | 10  **↑** | 67.8 (26.1 to 110.7) | 306.16 (227.48 to 481.96) | 17 | 510.89 (383.36 to 696.94) | 10  **↑** | 66.9 (20.4 to 127) | 221.34 (172.47 to 278.63) | 17 | 374.67 (271.95 to 470.91) | 14  **↑** | 69.3 (21.3 to 122.4) |
| **Oman** | 897.99 (688.85 to 1161.43) | 7 | 1006.49 (863.54 to 1159.22) | 5  **↑** | 12.1 (-16.2 to 50.8) | 1001.93 (720.37 to 1339.99) | 7 | 979.09 (815.11 to 1168.87) | 5  **↑** | -2.3 (-30.4 to 43.8) | 820.87 (613.27 to 1064.72) | 7 | 1060.5 (835.46 to 1284.76) | 5  **↑** | 29.2 (-13.8 to 85) |
| **Palestine** | 1014.72 (799.23 to 1267.61) | 6 | 1217.33 (1049.13 to 1399.08) | 3  **↑** | 20 (-7.3 to 57) | 1052.93 (818.79 to 1318.95) | 6 | 1155.53 (974.75 to 1347.12) | 4  **↑** | 9.7 (-16.7 to 46.8) | 973.88 (724.46 to 1272) | 5 | 1280.99 (1088.55 to 1491.34) | 3  **↑** | 31.5 (-3.8 to 79.4) |
| **Qatar** | 1811.9 (1525.53 to 2135.44) | 1 | 1637.23 (1298.72 to 2052.75) | 2  **↓** | -9.6 (-31.6 to 19.4) | 1877.72 (1477.4 to 2314.73) | 1 | 2282.23 (1814.27 to 2834.75) | 1  ***** | 21.5 (-7.9 to 58.3) | 1811.81 (1438.2 to 2244.65) | 1 | 1434.02 (1085.37 to 1851.36) | 2  **↓** | -20.9 (-46.3 to 13.3) |
| **Saudi Arabia** | 518.53 (385.87 to 692.75) | 11 | 395.52 (319.13 to 483.13) | 13  **↓** | -23.7 (-46.5 to 7.3) | 559.74 (412.01 to 744.69) | 12 | 358.58 (276.49 to 473.97) | 15  **↓** | -35.9 (-56.4 to -4.2) | 493.43 (355.17 to 694.61) | 9 | 419.56 (329.78 to 508.28) | 10  **↓** | -15 (-44.7 to 26.1) |
| **Sudan** | 242.38 (173.61 to 334.7) | 19 | 317.95 (212.13 to 447.14) | 16  **↑** | 31.2 (-5.5 to 83.1) | 278.42 (182.95 to 430.4) | 18 | 338.93 (219.5 to 498.21) | 17  **↑** | 21.7 (-10.7 to 67.7) | 209.95 (155.75 to 296.39) | 19 | 300.49 (181.73 to 463.61) | 17  **↑** | 43.1 (-8.7 to 116.6) |
| **Syrian Arab Republic** | 383.33 (299.93 to 479.95) | 14 | 295.79 (224.65 to 395.54) | 18  **↓** | -22.8 (-44.3 to 10.4) | 460 (346.61 to 587.62) | 13 | 335.27 (254.8 to 450.47) | 18  **↓** | -27.1 (-47 to 6.9) | 314.16 (242.27 to 401.15) | 14 | 267.93 (196.46 to 359.16) | 19  **↓** | -14.7 (-42.8 to 26.6) |
| **Tunisia** | 202.32 (163.78 to 269.36) | 21 | 274.72 (196.92 to 370.42) | 21  ***** | 35.8 (-3.8 to 87.5) | 218.82 (169.32 to 367.18) | 21 | 261.41 (186.74 to 374.99) | 20  **↑** | 19.5 (-16.9 to 73.7) | 187.26 (145.16 to 232.87) | 21 | 289.11 (198.13 to 418.19) | 18  **↑** | 54.4 (5.7 to 123.3) |
| **Turkey** | 810.77 (690.49 to 935.27) | 8 | 404.53 (322.6 to 497.21) | 11  **↓** | -50.1 (-61.8 to -34.1) | 902.45 (688.53 to 1071.98) | 8 | 405.63 (325.04 to 498.74) | 13  **↓** | -55.1 (-66.4 to -36.6) | 701.51 (554.64 to 885.05) | 8 | 396.86 (311.96 to 498.71) | 11  **↓** | -43.4 (-60.5 to -19.8) |
| **United Arab Emirates** | 1347.51 (1058.22 to 1645.92) | 3 | 1011.52 (745.83 to 1316.88) | 4  **↓** | -24.9 (-51.1 to 7.6) | 1473.53 (1047.54 to 1934.66) | 3 | 880.65 (657.75 to 1156.83) | 6  **↓** | -40.2 (-60.8 to -9.6) | 1242.49 (946.61 to 1614.02) | 3 | 1060.55 (741.45 to 1429.26) | 4  **↓** | -14.6 (-50.2 to 30.2) |
| **Yemen** | 244.54 (166.11 to 362.36) | 18 | 293.61 (204.52 to 420.54) | 19  **↓** | 20.1 (-12.2 to 63.3) | 273.82 (170.5 to 455.05) | 19 | 346.28 (216.68 to 522.93) | 16  **↑** | 26.5 (-10.5 to 79) | 213.57 (149.16 to 313.28) | 18 | 239.43 (165.51 to 342.61) | 21  **↓** | 12.1 (-19.6 to 60.6) |

ASR: Age-standardized rate; Data in parentheses are 95% Uncertainty Intervals (95% UIs).

Change in the ranking of countries (range from 1 (the highest estimate) to 21 (the lowest estimate)) in 2019 vs 1990 were classified by three groups: **Upward ↑**

**Monotone ***

**Downward ↓**

**YLDs**

| **Country** | **Both** | | | | | **Female** | | | | | **Male** | | | | |
| --- | --- | --- | --- | --- | --- | --- | --- | --- | --- | --- | --- | --- | --- | --- | --- |
|  | **1990** | | **2019** | | **% Change (1990 to 2019)** | **1990** | | **2019** | | **% Change (1990 to 2019)** | **1990** | | **2019** | | **% Change (1990 to 2019)** |
|  | **ASR (per 100,000)** | **Ranking** | **ASR (per 100,000)** | **Ranking** |  | **ASR (per 100,000)** | **Ranking** | **ASR (per 100,000)** | **Ranking** |  | **ASR (per 100,000)** | **Ranking** | **ASR (per 100,000)** | **Ranking** |  |
| **Afghanistan** | 359.03 (237.82 to 510.33) | 12 | 680.38 (457.42 to 959.04) | 9  **↑** | 89.5 (80.7 to 98.9) | 391.7 (256.57 to 557.14) | 8 | 755.41 (498.06 to 1053.59) | 4  **↑** | 92.9 (81.5 to 105.4) | 323.74 (212.15 to 460.63) | 15 | 603.45 (399.78 to 845.55) | 14  **↑** | 86.4 (73.9 to 99.4) |
| **Algeria** | 336.25 (224.77 to 476.26) | 15 | 632.1 (420.01 to 888.76) | 12  **↑** | 88 (77.1 to 99.8) | 342.3 (228.42 to 484.9) | 15 | 658.29 (437.11 to 930.07) | 8  **↑** | 92.3 (77 to 108.8) | 330.12 (219.18 to 468.38) | 14 | 606.64 (399.54 to 846.54) | 13  **↑** | 83.8 (71 to 99.1) |
| **Bahrain** | 646.24 (427.99 to 900.18) | 2 | 1200.22 (798.69 to 1666.01) | 2  ***** | 85.7 (70.9 to 100.9) | 601.63 (396.66 to 850.03) | 2 | 1148 (751.39 to 1594.66) | 2  ***** | 90.8 (75 to 107.4) | 685.2 (455.1 to 966.48) | 2 | 1231.28 (817.81 to 1719.68) | 2  ***** | 79.7 (61.8 to 98.3) |
| **Egypt** | 213.59 (142.32 to 296.85) | 21 | 494.79 (325.79 to 691.95) | 19  **↑** | 131.7 (114.9 to 149.5) | 251.42 (165.47 to 350.77) | 20 | 516.69 (339.62 to 727.05) | 18  **↑** | 105.5 (87.6 to 125.1) | 175.42 (115.99 to 247.74) | 21 | 477.66 (312.29 to 667.19) | 19  **↑** | 172.3 (150.2 to 198.2) |
| **Iran (Islamic Republic of)** | 288.11 (197.9 to 395.55) | 17 | 555.16 (380.82 to 758.69) | 17  ***** | 92.7 (85.8 to 100.7) | 282.6 (194.2 to 387.04) | 17 | 576.02 (397.52 to 788.53) | 13  **↑** | 103.8 (95.4 to 113.5) | 293.28 (201.91 to 402.94) | 17 | 534.15 (366.27 to 725.96) | 18  **↓** | 82.1 (74.2 to 91.1) |
| **Iraq** | 454.07 (304.49 to 632.07) | 5 | 696.33 (457.52 to 977.25) | 7  **↓** | 53.4 (40.9 to 66.1) | 469.81 (313.98 to 659.96) | 5 | 724.83 (473.98 to 1021.1) | 7  **↓** | 54.3 (38.8 to 70.5) | 439.14 (295.43 to 611.76) | 5 | 667.37 (440.97 to 933.48) | 10  **↓** | 52 (36.4 to 67.8) |
| **Jordan** | 413.33 (276.39 to 578.54) | 6 | 587.2 (387.05 to 817.21) | 14  **↓** | 42.1 (31.9 to 52.4) | 413.06 (275.51 to 584.95) | 6 | 532.24 (351 to 749.14) | 17  **↓** | 28.9 (17.8 to 41.7) | 412.07 (276.29 to 571.26) | 8 | 634.91 (420.61 to 887.67) | 12  **↓** | 54.1 (40.8 to 70.3) |
| **Kuwait** | 552.94 (364.38 to 773.11) | 3 | 834.95 (555.81 to 1174.95) | 4  **↓** | 51 (39.9 to 61.9) | 522.7 (346.19 to 739.09) | 4 | 754.06 (504.9 to 1072.25) | 5  **↓** | 44.3 (31.4 to 58.1) | 572.61 (376.92 to 810.81) | 3 | 891.78 (596.1 to 1251.37) | 3  ***** | 55.7 (42.8 to 70.8) |
| **Lebanon** | 409.4 (275.96 to 567.92) | 7 | 697.85 (456.44 to 980.58) | 6  **↑** | 70.5 (58.8 to 80.7) | 392.79 (264.79 to 550.97) | 7 | 643.85 (424.18 to 913.78) | 9  **↓** | 63.9 (51 to 76.4) | 426.3 (283.11 to 593.36) | 6 | 763.43 (503.84 to 1079.45) | 6  ***** | 79.1 (64.2 to 94.7) |
| **Libya** | 400.26 (266.35 to 561.78) | 8 | 765.15 (504.89 to 1073.99) | 5  **↑** | 91.2 (79.1 to 101.9) | 385.03 (254.24 to 538.66) | 9 | 748.03 (484.65 to 1055.58) | 6  **↑** | 94.3 (80.1 to 110.3) | 414.28 (274.17 to 583.68) | 7 | 781.81 (517.69 to 1098.8) | 5  **↑** | 88.7 (73.6 to 103) |
| **Morocco** | 282.65 (188.32 to 400.47) | 18 | 565.41 (372.41 to 796.4) | 15  **↑** | 100 (89.2 to 111.7) | 280.93 (186.47 to 399.7) | 18 | 569.48 (373.68 to 799.45) | 14  **↑** | 102.7 (87.6 to 119.3) | 284.27 (187.04 to 399.08) | 18 | 561.38 (369.87 to 794.85) | 15  **↑** | 97.5 (83.6 to 111.2) |
| **Oman** | 350.35 (232.15 to 494.35) | 14 | 611.97 (404.2 to 857.55) | 13  **↑** | 74.7 (64.4 to 85.5) | 349.2 (232.96 to 496.71) | 14 | 564.9 (368 to 788.54) | 15  **↓** | 61.8 (49.7 to 76) | 356.03 (235.82 to 504.27) | 12 | 652.83 (429.23 to 916.03) | 11  **↑** | 83.4 (69.3 to 99.9) |
| **Palestine** | 384.2 (254.7 to 539.77) | 9 | 690.6 (455.84 to 971.37) | 8  **↑** | 79.7 (65.4 to 95.5) | 369.62 (246.07 to 517.31) | 10 | 634.55 (416.28 to 902) | 11  **↓** | 71.7 (56 to 89.3) | 402.04 (261.69 to 572.77) | 11 | 750.41 (500.89 to 1051.13) | 8  **↑** | 86.7 (67.6 to 106.4) |
| **Qatar** | 716.24 (473.91 to 1001.32) | 1 | 1338.09 (886.53 to 1857.3) | 1  ***** | 86.8 (71.7 to 103.8) | 697.48 (460.32 to 971.24) | 1 | 1346.35 (886.63 to 1889.05) | 1  ***** | 93 (73.1 to 113) | 733.67 (481.92 to 1045.42) | 1 | 1335.74 (887.6 to 1873.41) | 1  ***** | 82.1 (65.5 to 100.8) |
| **Saudi Arabia** | 383.79 (259.98 to 523.76) | 10 | 668.95 (450.73 to 939.72) | 11  **↓** | 74.3 (61.6 to 90) | 354.33 (239.87 to 486.98) | 12 | 636.05 (414.48 to 891.98) | 10  **↑** | 79.5 (62.8 to 98.3) | 405.52 (272.83 to 557.99) | 10 | 690.81 (460.86 to 979.51) | 9  **↑** | 70.3 (55.2 to 89.4) |
| **Sudan** | 267.9 (179.14 to 373.08) | 19 | 537.67 (358.15 to 753.71) | 18  **↑** | 100.7 (87.1 to 116.4) | 255.8 (171.02 to 356.11) | 19 | 513.86 (343.29 to 713.25) | 19  ***** | 100.9 (84.7 to 118.7) | 278.67 (186.56 to 390.75) | 19 | 557.37 (370.34 to 783) | 16  **↑** | 100 (82.1 to 120.8) |
| **Syrian Arab Republic** | 356.8 (238.13 to 502.31) | 13 | 557.36 (368.58 to 780.6) | 16  **↓** | 56.2 (46.8 to 67.3) | 364.28 (239.08 to 506.17) | 11 | 562.9 (372.89 to 801.2) | 16  **↓** | 54.5 (42.2 to 69.6) | 349.76 (226.99 to 488.96) | 13 | 552.22 (358.9 to 783.33) | 17  **↓** | 57.9 (45 to 73) |
| **Tunisia** | 361.66 (240.3 to 507.26) | 11 | 669.12 (438.79 to 939.6) | 10  **↑** | 85 (75.9 to 94.6) | 314.18 (209.73 to 446.52) | 16 | 590.11 (382.3 to 837.88) | 12  **↑** | 87.8 (74.9 to 101.3) | 406.5 (266.16 to 573.4) | 9 | 751.83 (493.68 to 1053.64) | 7  **↑** | 85 (70.9 to 98.8) |
| **Turkey** | 330.83 (218.21 to 464.09) | 16 | 465.41 (304.85 to 649.04) | 20  **↓** | 40.7 (27.8 to 53.8) | 350.99 (233.49 to 488.5) | 13 | 467.54 (310.26 to 654.59) | 20  **↓** | 33.2 (19.2 to 48.1) | 309.54 (203.79 to 435.43) | 16 | 463.33 (299.55 to 659.53) | 20  **↓** | 49.7 (32.7 to 68) |
| **United Arab Emirates** | 536.7 (357.82 to 749) | 4 | 853.67 (570.37 to 1189.46) | 3  **↑** | 59.1 (49 to 70.3) | 559.29 (371.13 to 791.7) | 3 | 860.64 (570.12 to 1207.79) | 3  ***** | 53.9 (41.6 to 67.6) | 522.68 (349.27 to 727.07) | 4 | 849.2 (565.62 to 1188.94) | 4  ***** | 62.5 (50.4 to 75.8) |
| **Yemen** | 222.76 (148.25 to 316.57) | 20 | 382.89 (253.69 to 536.55) | 21  **↓** | 71.9 (62.9 to 82.3) | 220.98 (145.19 to 310.56) | 21 | 390.12 (253.96 to 541.67) | 21  ***** | 76.5 (63.9 to 90.4) | 225.54 (148.92 to 324.19) | 20 | 375.52 (248.65 to 536.82) | 21  **↓** | 66.5 (53.7 to 81.3) |

ASR: Age-standardized rate; Data in parentheses are 95% Uncertainty Intervals (95% UIs).

Change in the ranking of countries (range from 1 (the highest estimate) to 21 (the lowest estimate)) in 2019 vs 1990 were classified by three groups: **Upward ↑**

**Monotone ***

**Downward ↓**
