# Supplementary material for: Burden of type 2 diabetes mellitus and its risk factors in North Africa and the Middle East, 1990–2019: findings from the Global Burden of Disease study 2019
Source: BMC Public Health. 2024 Jan 5;24:98. doi: 10.1186/s12889-023-16540-8 (PMC10768242; doi:10.1186/s12889-023-16540-8)
Supplement: Supplementary file 4 — Additional file 4: Supplementary Table 4. Annual percent change of the age-standardized rates in the region and its countries by the measures and sex. [file 12889_2023_16540_MOESM4_ESM.docx]

| **Province** | **Measure** | **APC (1990 to 2019)** | | |
| --- | --- | --- | --- | --- |
|  |  |  |  |  |
|  |  | **Both** | **Female** | **Male** |
| **North Africa and Middle East** | **Incidence** | 2.32 (2.21 to 2.43) | 2.22 (2.11 to 2.32) | 2.42 (2.30 to 2.55) |
|  | **Prevalence** | 2.51 (2.37 to 2.64) | 2.44 (2.31 to 2.57) | 2.58 (2.44 to 2.72) |
|  | **Deaths** | 0.28 (0.13 to 0.43) | 0.13 (-0.04 to 0.30) | 0.51 (0.38 to 0.64) |
|  | **DALYs** | 1.21 (1.08 to 1.34) | 1.02 (0.89 to 1.16) | 1.43 (1.30 to 1.55) |
|  | **YLLs** | 0.13 (0.02 to 0.24) | -0.08 (-0.20 to 0.03) | 0.39 (0.28 to 0.50) |
|  | **YLDs** | 2.50 (2.35 to 2.66) | 2.46 (2.30 to 2.62) | 2.55 (2.40 to 2.70) |
| **Afghanistan** | **Incidence** | 2.17 (2.13 to 2.21) | 2.24 (2.19 to 2.28) | 2.13 (2.09 to 2.17) |
|  | **Prevalence** | 2.34 (2.29 to 2.39) | 2.41 (2.34 to 2.47) | 2.27 (2.23 to 2.30) |
|  | **Deaths** | 1.63 (1.48 to 1.78) | 1.79 (1.62 to 1.96) | 0.78 (0.66 to 0.91) |
|  | **DALYs** | 1.83 (1.74 to 1.93) | 1.91 (1.79 to 2.02) | 1.48 (1.42 to 1.54) |
|  | **YLLs** | 1.51 (1.37 to 1.65) | 1.65 (1.49 to 1.81) | 0.69 (0.59 to 0.79) |
|  | **YLDs** | 2.31 (2.26 to 2.36) | 2.39 (2.33 to 2.45) | 2.22 (2.18 to 2.26) |
| **Algeria** | **Incidence** | 2.20 (2.12 to 2.27) | 2.29 (2.23 to 2.35) | 2.10 (1.99 to 2.21) |
|  | **Prevalence** | 2.32 (2.25 to 2.38) | 2.42 (2.38 to 2.46) | 2.22 (2.12 to 2.32) |
|  | **Deaths** | 0.72 (0.43 to 1.00) | 1.11 (0.78 to 1.45) | 0.35 (0.05 to 0.66) |
|  | **DALYs** | 1.61 (1.45 to 1.78) | 1.71 (1.55 to 1.87) | 1.54 (1.35 to 1.73) |
|  | **YLLs** | 0.57 (0.29 to 0.85) | 0.81 (0.52 to 1.10) | 0.36 (0.04 to 0.68) |
|  | **YLDs** | 2.30 (2.22 to 2.37) | 2.40 (2.35 to 2.45) | 2.20 (2.09 to 2.30) |
| **Bahrain** | **Incidence** | 1.87 (1.65 to 2.10) | 2.05 (1.84 to 2.25) | 1.73 (1.49 to 1.97) |
|  | **Prevalence** | 2.18 (1.97 to 2.40) | 2.32 (2.10 to 2.54) | 2.03 (1.80 to 2.27) |
|  | **Deaths** | 2.04 (1.52 to 2.57) | 2.31 (1.80 to 2.82) | 1.80 (1.24 to 2.36) |
|  | **DALYs** | 1.63 (1.27 to 1.99) | 1.81 (1.46 to 2.16) | 1.47 (1.10 to 1.84) |
|  | **YLLs** | 1.39 (0.91 to 1.87) | 1.60 (1.14 to 2.06) | 1.23 (0.72 to 1.73) |
|  | **YLDs** | 2.10 (1.97 to 2.23) | 2.25 (2.13 to 2.38) | 1.94 (1.78 to 2.10) |
| **Egypt** | **Incidence** | 2.74 (2.62 to 2.86) | 2.32 (2.21 to 2.43) | 3.33 (3.17 to 3.49) |
|  | **Prevalence** | 3.21 (3.05 to 3.37) | 2.80 (2.66 to 2.94) | 3.79 (3.59 to 3.99) |
|  | **Deaths** | 1.63 (1.50 to 1.76) | 2.07 (1.90 to 2.24) | 1.53 (1.40 to 1.66) |
|  | **DALYs** | 2.16 (2.05 to 2.28) | 2.13 (1.99 to 2.26) | 2.38 (2.26 to 2.51) |
|  | **YLLs** | 1.64 (1.51 to 1.77) | 1.80 (1.64 to 1.97) | 1.67 (1.55 to 1.79) |
|  | **YLDs** | 3.14 (2.99 to 3.29) | 2.73 (2.60 to 2.87) | 3.73 (3.53 to 3.92) |
| **Iran (Islamic Republic of)** | **Incidence** | 2.50 (2.38 to 2.62) | 2.70 (2.57 to 2.82) | 2.32 (2.18 to 2.45) |
|  | **Prevalence** | 2.54 (2.40 to 2.68) | 2.67 (2.51 to 2.83) | 2.41 (2.27 to 2.55) |
|  | **Deaths** | 2.31 (2.05 to 2.57) | 2.21 (1.99 to 2.42) | 2.52 (2.21 to 2.83) |
|  | **DALYs** | 2.49 (2.36 to 2.63) | 2.47 (2.37 to 2.57) | 2.52 (2.35 to 2.69) |
|  | **YLLs** | 2.38 (2.10 to 2.66) | 2.16 (1.92 to 2.39) | 2.65 (2.32 to 2.98) |
|  | **YLDs** | 2.58 (2.41 to 2.75) | 2.73 (2.53 to 2.93) | 2.43 (2.27 to 2.59) |
| **Iraq** | **Incidence** | 1.34 (1.29 to 1.39) | 1.40 (1.33 to 1.48) | 1.26 (1.22 to 1.29) |
|  | **Prevalence** | 1.45 (1.37 to 1.54) | 1.48 (1.37 to 1.59) | 1.41 (1.36 to 1.47) |
|  | **Deaths** | -0.39 (-0.47 to -0.31) | -0.73 (-0.81 to -0.66) | -0.02 (-0.16 to 0.12) |
|  | **DALYs** | 0.03 (-0.03 to 0.10) | -0.21 (-0.31 to -0.10) | 0.29 (0.23 to 0.35) |
|  | **YLLs** | -0.78 (-0.87 to -0.68) | -1.20 (-1.35 to -1.06) | -0.34 (-0.44 to -0.23) |
|  | **YLDs** | 1.45 (1.37 to 1.54) | 1.50 (1.39 to 1.60) | 1.40 (1.34 to 1.46) |
| **Jordan** | **Incidence** | 0.72 (0.54 to 0.90) | 0.18 (-0.10 to 0.46) | 1.17 (1.06 to 1.27) |
|  | **Prevalence** | 1.04 (0.86 to 1.23) | 0.56 (0.30 to 0.82) | 1.43 (1.28 to 1.58) |
|  | **Deaths** | -1.98 (-2.40 to -1.55) | -2.85 (-3.56 to -2.14) | -0.67 (-0.82 to -0.52) |
|  | **DALYs** | -1.13 (-1.47 to -0.80) | -2.07 (-2.61 to -1.52) | -0.07 (-0.21 to 0.08) |
|  | **YLLs** | -2.31 (-2.79 to -1.82) | -3.33 (-4.08 to -2.58) | -0.99 (-1.19 to -0.80) |
|  | **YLDs** | 1.05 (0.89 to 1.20) | 0.67 (0.46 to 0.88) | 1.38 (1.26 to 1.50) |
| **Kuwait** | **Incidence** | 0.99 (0.75 to 1.22) | 0.83 (0.60 to 1.05) | 1.14 (0.92 to 1.37) |
|  | **Prevalence** | 1.14 (0.86 to 1.41) | 0.99 (0.71 to 1.27) | 1.25 (0.98 to 1.51) |
|  | **Deaths** | -1.79 (-2.49 to -1.09) | -2.31 (-2.89 to -1.72) | -1.33 (-2.18 to -0.48) |
|  | **DALYs** | -0.09 (-0.52 to 0.35) | -0.49 (-0.89 to -0.10) | 0.18 (-0.29 to 0.65) |
|  | **YLLs** | -2.21 (-2.99 to -1.43) | -2.81 (-3.48 to -2.14) | -1.78 (-2.67 to -0.88) |
|  | **YLDs** | 1.11 (0.84 to 1.38) | 0.96 (0.69 to 1.24) | 1.21 (0.94 to 1.47) |
| **Lebanon** | **Incidence** | 1.77 (1.72 to 1.82) | 1.61 (1.57 to 1.64) | 1.99 (1.92 to 2.07) |
|  | **Prevalence** | 1.91 (1.86 to 1.96) | 1.71 (1.68 to 1.74) | 2.14 (2.06 to 2.22) |
|  | **Deaths** | -0.63 (-0.87 to -0.40) | -1.58 (-1.71 to -1.46) | 0.17 (-0.22 to 0.55) |
|  | **DALYs** | 0.89 (0.75 to 1.03) | 0.51 (0.44 to 0.58) | 1.29 (1.07 to 1.51) |
|  | **YLLs** | -0.78 (-1.06 to -0.50) | -1.79 (-1.96 to -1.61) | 0.10 (-0.34 to 0.54) |
|  | **YLDs** | 1.92 (1.82 to 2.02) | 1.77 (1.65 to 1.88) | 2.12 (2.02 to 2.21) |
| **Libya** | **Incidence** | 2.22 (2.12 to 2.32) | 2.27 (2.20 to 2.35) | 2.19 (2.06 to 2.33) |
|  | **Prevalence** | 2.37 (2.25 to 2.50) | 2.41 (2.32 to 2.50) | 2.36 (2.20 to 2.52) |
|  | **Deaths** | 1.36 (1.12 to 1.60) | 1.42 (1.11 to 1.73) | 1.26 (1.07 to 1.46) |
|  | **DALYs** | 1.96 (1.85 to 2.07) | 1.97 (1.84 to 2.09) | 1.94 (1.82 to 2.06) |
|  | **YLLs** | 1.35 (1.13 to 1.57) | 1.37 (1.07 to 1.67) | 1.27 (1.10 to 1.45) |
|  | **YLDs** | 2.31 (2.18 to 2.43) | 2.35 (2.27 to 2.44) | 2.27 (2.12 to 2.42) |
| **Morocco** | **Incidence** | 2.41 (2.37 to 2.46) | 2.47 (2.41 to 2.53) | 2.36 (2.32 to 2.39) |
|  | **Prevalence** | 2.57 (2.52 to 2.61) | 2.63 (2.56 to 2.70) | 2.50 (2.47 to 2.54) |
|  | **Deaths** | 2.17 (2.00 to 2.33) | 2.38 (2.11 to 2.64) | 1.89 (1.51 to 2.27) |
|  | **DALYs** | 2.34 (2.24 to 2.43) | 2.44 (2.28 to 2.60) | 2.23 (2.11 to 2.35) |
|  | **YLLs** | 2.12 (1.96 to 2.28) | 2.28 (2.02 to 2.53) | 1.89 (1.60 to 2.18) |
|  | **YLDs** | 2.53 (2.48 to 2.58) | 2.60 (2.53 to 2.66) | 2.46 (2.42 to 2.50) |
| **Oman** | **Incidence** | 1.75 (1.65 to 1.85) | 1.41 (1.33 to 1.49) | 1.93 (1.82 to 2.03) |
|  | **Prevalence** | 1.70 (1.49 to 1.91) | 1.39 (1.17 to 1.60) | 1.89 (1.68 to 2.10) |
|  | **Deaths** | 1.31 (1.12 to 1.50) | 0.58 (0.40 to 0.76) | 2.02 (1.79 to 2.24) |
|  | **DALYs** | 1.05 (0.86 to 1.24) | 0.50 (0.33 to 0.67) | 1.54 (1.33 to 1.75) |
|  | **YLLs** | 0.74 (0.43 to 1.05) | 0.10 (-0.18 to 0.39) | 1.35 (1.02 to 1.69) |
|  | **YLDs** | 1.71 (1.52 to 1.91) | 1.41 (1.22 to 1.60) | 1.90 (1.71 to 2.09) |
| **Palestine** | **Incidence** | 2.08 (1.98 to 2.17) | 1.98 (1.85 to 2.11) | 2.11 (2.03 to 2.20) |
|  | **Prevalence** | 2.19 (2.09 to 2.29) | 2.15 (2.01 to 2.28) | 2.19 (2.10 to 2.27) |
|  | **Deaths** | 0.82 (0.39 to 1.25) | 0.59 (0.12 to 1.06) | 1.10 (0.72 to 1.48) |
|  | **DALYs** | 1.06 (0.78 to 1.35) | 0.85 (0.53 to 1.17) | 1.26 (1.00 to 1.51) |
|  | **YLLs** | 0.54 (0.14 to 0.94) | 0.27 (-0.16 to 0.71) | 0.80 (0.43 to 1.16) |
|  | **YLDs** | 2.16 (2.07 to 2.25) | 2.13 (2.00 to 2.25) | 2.16 (2.09 to 2.24) |
| **Qatar** | **Incidence** | 1.74 (1.43 to 2.05) | 1.87 (1.51 to 2.22) | 1.69 (1.40 to 1.99) |
|  | **Prevalence** | 2.28 (1.93 to 2.63) | 2.37 (1.98 to 2.76) | 2.21 (1.88 to 2.55) |
|  | **Deaths** | 0.75 (0.22 to 1.29) | 2.39 (1.64 to 3.15) | 0.12 (-0.39 to 0.63) |
|  | **DALYs** | 0.80 (0.31 to 1.29) | 1.69 (1.04 to 2.35) | 0.42 (0.02 to 0.82) |
|  | **YLLs** | 0.02 (-0.60 to 0.65) | 1.41 (0.61 to 2.21) | -0.62 (-1.15 to -0.08) |
|  | **YLDs** | 2.18 (1.84 to 2.53) | 2.29 (1.91 to 2.68) | 2.10 (1.78 to 2.43) |
| **Saudi Arabia** | **Incidence** | 1.74 (1.68 to 1.79) | 1.83 (1.70 to 1.95) | 1.69 (1.65 to 1.72) |
|  | **Prevalence** | 2.04 (1.97 to 2.10) | 2.15 (2.03 to 2.27) | 1.95 (1.88 to 2.03) |
|  | **Deaths** | -1.61 (-1.97 to -1.26) | -2.13 (-2.51 to -1.75) | -1.29 (-1.63 to -0.95) |
|  | **DALYs** | 0.19 (0.02 to 0.35) | -0.14 (-0.34 to 0.05) | 0.38 (0.22 to 0.53) |
|  | **YLLs** | -1.63 (-1.99 to -1.28) | -2.27 (-2.66 to -1.88) | -1.22 (-1.56 to -0.88) |
|  | **YLDs** | 1.96 (1.91 to 2.02) | 2.09 (1.97 to 2.22) | 1.86 (1.79 to 1.92) |
| **Sudan** | **Incidence** | 2.29 (2.25 to 2.32) | 2.26 (2.20 to 2.31) | 2.32 (2.28 to 2.35) |
|  | **Prevalence** | 2.48 (2.41 to 2.54) | 2.45 (2.37 to 2.54) | 2.50 (2.45 to 2.55) |
|  | **Deaths** | 1.36 (1.17 to 1.56) | 1.21 (1.03 to 1.39) | 1.56 (1.31 to 1.80) |
|  | **DALYs** | 2.00 (1.91 to 2.10) | 1.88 (1.76 to 2.00) | 2.12 (2.02 to 2.22) |
|  | **YLLs** | 1.25 (1.06 to 1.44) | 1.06 (0.88 to 1.24) | 1.48 (1.24 to 1.72) |
|  | **YLDs** | 2.54 (2.47 to 2.61) | 2.56 (2.46 to 2.66) | 2.51 (2.47 to 2.56) |
| **Syrian Arab Republic** | **Incidence** | 1.36 (1.30 to 1.41) | 1.27 (1.17 to 1.37) | 1.44 (1.41 to 1.48) |
|  | **Prevalence** | 1.41 (1.36 to 1.47) | 1.32 (1.23 to 1.42) | 1.50 (1.45 to 1.54) |
|  | **Deaths** | -1.27 (-1.62 to -0.92) | -1.28 (-1.66 to -0.91) | -0.90 (-1.21 to -0.58) |
|  | **DALYs** | 0.07 (-0.12 to 0.26) | -0.19 (-0.40 to 0.02) | 0.38 (0.21 to 0.55) |
|  | **YLLs** | -1.64 (-2.03 to -1.25) | -1.88 (-2.28 to -1.47) | -1.23 (-1.59 to -0.86) |
|  | **YLDs** | 1.40 (1.34 to 1.45) | 1.32 (1.22 to 1.42) | 1.47 (1.43 to 1.51) |
| **Tunisia** | **Incidence** | 2.22 (2.06 to 2.38) | 2.06 (2.04 to 2.08) | 2.35 (2.08 to 2.63) |
|  | **Prevalence** | 2.30 (2.18 to 2.42) | 2.13 (2.09 to 2.16) | 2.47 (2.24 to 2.70) |
|  | **Deaths** | 1.26 (1.16 to 1.37) | 0.90 (0.79 to 1.01) | 1.65 (1.53 to 1.77) |
|  | **DALYs** | 1.93 (1.82 to 2.04) | 1.65 (1.61 to 1.69) | 2.19 (2.01 to 2.38) |
|  | **YLLs** | 1.24 (1.14 to 1.34) | 0.84 (0.73 to 0.95) | 1.64 (1.55 to 1.74) |
|  | **YLDs** | 2.26 (2.13 to 2.40) | 2.10 (2.07 to 2.14) | 2.43 (2.18 to 2.67) |
| **Turkey** | **Incidence** | 2.27 (1.77 to 2.77) | 2.01 (1.51 to 2.53) | 2.55 (2.06 to 3.04) |
|  | **Prevalence** | 2.84 (2.20 to 3.49) | 2.67 (2.00 to 3.36) | 3.04 (2.43 to 3.65) |
|  | **Deaths** | -1.65 (-1.94 to -1.37) | -1.97 (-2.35 to -1.59) | -1.23 (-1.45 to -1.01) |
|  | **DALYs** | -0.25 (-0.64 to 0.15) | -0.55 (-1.00 to -0.10) | 0.14 (-0.20 to 0.47) |
|  | **YLLs** | -2.26 (-2.47 to -2.04) | -2.65 (-2.93 to -2.37) | -1.79 (-1.96 to -1.61) |
|  | **YLDs** | 2.85 (2.20 to 3.51) | 2.76 (2.06 to 3.46) | 2.98 (2.37 to 3.59) |
| **United Arab Emirates** | **Incidence** | 1.39 (1.16 to 1.62) | 1.31 (0.97 to 1.65) | 1.46 (1.26 to 1.66) |
|  | **Prevalence** | 1.71 (1.46 to 1.96) | 1.62 (1.28 to 1.96) | 1.79 (1.59 to 1.99) |
|  | **Deaths** | -1.02 (-1.75 to -0.28) | -1.23 (-2.62 to 0.17) | -0.81 (-1.21 to -0.41) |
|  | **DALYs** | 0.01 (-0.47 to 0.49) | -0.16 (-1.07 to 0.76) | 0.12 (-0.14 to 0.39) |
|  | **YLLs** | -0.93 (-1.60 to -0.26) | -1.21 (-2.51 to 0.10) | -0.80 (-1.16 to -0.43) |
|  | **YLDs** | 1.69 (1.48 to 1.89) | 1.61 (1.33 to 1.89) | 1.75 (1.58 to 1.92) |
| **Yemen** | **Incidence** | 1.83 (1.73 to 1.93) | 1.95 (1.81 to 2.10) | 1.73 (1.67 to 1.79) |
|  | **Prevalence** | 1.86 (1.80 to 1.91) | 1.97 (1.94 to 2.00) | 1.74 (1.62 to 1.85) |
|  | **Deaths** | 0.93 (0.78 to 1.08) | 1.16 (1.01 to 1.31) | 0.68 (0.53 to 0.83) |
|  | **DALYs** | 1.40 (1.32 to 1.49) | 1.53 (1.44 to 1.61) | 1.26 (1.16 to 1.35) |
|  | **YLLs** | 0.87 (0.71 to 1.02) | 1.07 (0.91 to 1.22) | 0.60 (0.46 to 0.75) |
|  | **YLDs** | 1.88 (1.85 to 1.91) | 1.99 (1.94 to 2.04) | 1.75 (1.68 to 1.82) |

Data in parentheses are 95% Confidence Intervals (95% CIs)

APC=Average Annual Percent Change; DALYs=Disability-Adjusted Life Years; YLLs=Years of Life Lost; YLDs=Years Lived with Disability
