# Supplementary material for: Burden of type 2 diabetes mellitus and its risk factors in North Africa and the Middle East, 1990–2019: findings from the Global Burden of Disease study 2019
Source: BMC Public Health. 2024 Jan 5;24:98. doi: 10.1186/s12889-023-16540-8 (PMC10768242; doi:10.1186/s12889-023-16540-8)
Supplement: Supplementary file 5 — Additional file 5: Supplementary Table 5. Socio-demographic index of the region countries during 1990 to 2019. [file 12889_2023_16540_MOESM5_ESM.pdf]

Country name	Year	SDI	SDI quintiles
Afghanistan	1990	0.187	1
Afghanistan	1991	0.191	1
Afghanistan	1992	0.195	1
Afghanistan	1993	0.196	1
Afghanistan	1994	0.194	1
Afghanistan	1995	0.194	1
Afghanistan	1996	0.193	1
Afghanistan	1997	0.192	1
Afghanistan	1998	0.19	1
Afghanistan	1999	0.189	1
Afghanistan	2000	0.188	1
Afghanistan	2001	0.188	1
Afghanistan	2002	0.194	1
Afghanistan	2003	0.202	1
Afghanistan	2004	0.209	1
Afghanistan	2005	0.216	1
Afghanistan	2006	0.224	1
Afghanistan	2007	0.234	1
Afghanistan	2008	0.243	1
Afghanistan	2009	0.253	1
Afghanistan	2010	0.264	1
Afghanistan	2011	0.274	1
Afghanistan	2012	0.285	1
Afghanistan	2013	0.295	1
Afghanistan	2014	0.304	1
Afghanistan	2015	0.313	1
Afghanistan	2016	0.321	1
Afghanistan	2017	0.329	1
Afghanistan	2018	0.337	1
Afghanistan	2019	0.343	1

Country name	Year	SDI	SDI quintiles
Algeria	1990	0.436	3
Algeria	1991	0.446	3
Algeria	1992	0.456	3
Algeria	1993	0.465	3
Algeria	1994	0.474	3
Algeria	1995	0.483	3
Algeria	1996	0.492	3
Algeria	1997	0.5	3
Algeria	1998	0.509	3
Algeria	1999	0.518	3
Algeria	2000	0.526	3
Algeria	2001	0.534	3
Algeria	2002	0.542	3
Algeria	2003	0.55	2
Algeria	2004	0.558	2
Algeria	2005	0.566	2
Algeria	2006	0.573	2
Algeria	2007	0.58	2
Algeria	2008	0.587	2
Algeria	2009	0.593	2
Algeria	2010	0.599	2
Algeria	2011	0.605	2
Algeria	2012	0.611	2
Algeria	2013	0.617	2
Algeria	2014	0.623	2
Algeria	2015	0.628	2
Algeria	2016	0.634	2
Algeria	2017	0.64	2
Algeria	2018	0.646	2
Algeria	2019	0.652	2

Country name	Year	SDI	SDI quintiles
Bahrain	1990	0.553	5
Bahrain	1991	0.56	5
Bahrain	1992	0.566	5
Bahrain	1993	0.573	5
Bahrain	1994	0.58	5
Bahrain	1995	0.587	5
Bahrain	1996	0.595	5
Bahrain	1997	0.602	5
Bahrain	1998	0.61	5
Bahrain	1999	0.618	5
Bahrain	2000	0.626	5
Bahrain	2001	0.634	5
Bahrain	2002	0.643	5
Bahrain	2003	0.653	5
Bahrain	2004	0.662	5
Bahrain	2005	0.672	5
Bahrain	2006	0.68	5
Bahrain	2007	0.688	4
Bahrain	2008	0.697	4
Bahrain	2009	0.705	4
Bahrain	2010	0.711	4
Bahrain	2011	0.715	4
Bahrain	2012	0.72	4
Bahrain	2013	0.726	4
Bahrain	2014	0.731	4
Bahrain	2015	0.735	4
Bahrain	2016	0.739	4
Bahrain	2017	0.743	4
Bahrain	2018	0.747	4
Bahrain	2019	0.751	4

Country name	Year	SDI	SDI quintiles
Egypt	1990	0.403	2
Egypt	1991	0.415	2
Egypt	1992	0.426	2
Egypt	1993	0.437	2
Egypt	1994	0.448	2
Egypt	1995	0.459	2
Egypt	1996	0.469	2
Egypt	1997	0.478	2
Egypt	1998	0.487	2
Egypt	1999	0.496	2
Egypt	2000	0.504	2
Egypt	2001	0.512	2
Egypt	2002	0.52	2
Egypt	2003	0.527	2
Egypt	2004	0.535	2
Egypt	2005	0.542	2
Egypt	2006	0.549	2
Egypt	2007	0.557	2
Egypt	2008	0.565	2
Egypt	2009	0.573	2
Egypt	2010	0.582	2
Egypt	2011	0.591	2
Egypt	2012	0.6	2
Egypt	2013	0.609	2
Egypt	2014	0.617	2
Egypt	2015	0.626	2
Egypt	2016	0.635	2
Egypt	2017	0.643	2
Egypt	2018	0.651	2
Egypt	2019	0.658	2

Country name	Year	SDI	SDI quintiles
Iran (Islamic Republic of)	1990	0.404	2
Iran (Islamic Republic of)	1991	0.419	2
Iran (Islamic Republic of)	1992	0.433	2
Iran (Islamic Republic of)	1993	0.446	2
Iran (Islamic Republic of)	1994	0.457	2
Iran (Islamic Republic of)	1995	0.468	2
Iran (Islamic Republic of)	1996	0.477	2
Iran (Islamic Republic of)	1997	0.486	2
Iran (Islamic Republic of)	1998	0.496	2
Iran (Islamic Republic of)	1999	0.506	2
Iran (Islamic Republic of)	2000	0.517	2
Iran (Islamic Republic of)	2001	0.529	2
Iran (Islamic Republic of)	2002	0.541	2
Iran (Islamic Republic of)	2003	0.552	3
Iran (Islamic Republic of)	2004	0.562	3
Iran (Islamic Republic of)	2005	0.571	3
Iran (Islamic Republic of)	2006	0.581	3
Iran (Islamic Republic of)	2007	0.593	3
Iran (Islamic Republic of)	2008	0.603	3
Iran (Islamic Republic of)	2009	0.613	3
Iran (Islamic Republic of)	2010	0.622	3
Iran (Islamic Republic of)	2011	0.63	3
Iran (Islamic Republic of)	2012	0.635	3
Iran (Islamic Republic of)	2013	0.64	3
Iran (Islamic Republic of)	2014	0.645	3
Iran (Islamic Republic of)	2015	0.649	3
Iran (Islamic Republic of)	2016	0.654	3
Iran (Islamic Republic of)	2017	0.659	3
Iran (Islamic Republic of)	2018	0.665	3
Iran (Islamic Republic of)	2019	0.67	2

Country name	Year	SDI	SDI quintiles
Iraq	1990	0.392	2
Iraq	1991	0.397	2
Iraq	1992	0.402	2
Iraq	1993	0.407	2
Iraq	1994	0.412	2
Iraq	1995	0.417	2
Iraq	1996	0.422	2
Iraq	1997	0.429	2
Iraq	1998	0.439	2
Iraq	1999	0.451	2
Iraq	2000	0.462	2
Iraq	2001	0.475	2
Iraq	2002	0.485	2
Iraq	2003	0.493	2
Iraq	2004	0.504	2
Iraq	2005	0.514	2
Iraq	2006	0.525	2
Iraq	2007	0.535	2
Iraq	2008	0.547	2
Iraq	2009	0.558	2
Iraq	2010	0.57	2
Iraq	2011	0.583	2
Iraq	2012	0.597	2
Iraq	2013	0.61	2
Iraq	2014	0.622	2
Iraq	2015	0.632	2
Iraq	2016	0.644	2
Iraq	2017	0.654	2
Iraq	2018	0.663	2
Iraq	2019	0.671	3

Country name	Year	SDI	SDI quintiles
Jordan	1990	0.52	4
Jordan	1991	0.529	4
Jordan	1992	0.537	4
Jordan	1993	0.546	4
Jordan	1994	0.554	4
Jordan	1995	0.562	4
Jordan	1996	0.57	4
Jordan	1997	0.577	4
Jordan	1998	0.585	4
Jordan	1999	0.592	4
Jordan	2000	0.6	4
Jordan	2001	0.607	4
Jordan	2002	0.614	4
Jordan	2003	0.621	4
Jordan	2004	0.63	4
Jordan	2005	0.639	4
Jordan	2006	0.648	4
Jordan	2007	0.656	4
Jordan	2008	0.664	4
Jordan	2009	0.673	4
Jordan	2010	0.681	4
Jordan	2011	0.688	3
Jordan	2012	0.695	3
Jordan	2013	0.702	3
Jordan	2014	0.707	3
Jordan	2015	0.713	4
Jordan	2016	0.718	4
Jordan	2017	0.723	4
Jordan	2018	0.727	4
Jordan	2019	0.731	4

Country name	Year	SDI	SDI quintiles
Kuwait	1990	0.655	5
Kuwait	1991	0.659	5
Kuwait	1992	0.662	5
Kuwait	1993	0.667	5
Kuwait	1994	0.673	5
Kuwait	1995	0.68	5
Kuwait	1996	0.689	5
Kuwait	1997	0.699	5
Kuwait	1998	0.709	5
Kuwait	1999	0.717	5
Kuwait	2000	0.724	5
Kuwait	2001	0.729	5
Kuwait	2002	0.735	5
Kuwait	2003	0.742	5
Kuwait	2004	0.75	5
Kuwait	2005	0.76	5
Kuwait	2006	0.769	5
Kuwait	2007	0.777	5
Kuwait	2008	0.785	5
Kuwait	2009	0.793	5
Kuwait	2010	0.801	5
Kuwait	2011	0.808	5
Kuwait	2012	0.815	5
Kuwait	2013	0.822	5
Kuwait	2014	0.828	5
Kuwait	2015	0.834	5
Kuwait	2016	0.839	5
Kuwait	2017	0.844	5
Kuwait	2018	0.848	5
Kuwait	2019	0.851	5

Country name	Year	SDI	SDI quintiles
Lebanon	1990	0.462	4
Lebanon	1991	0.47	4
Lebanon	1992	0.477	4
Lebanon	1993	0.485	3
Lebanon	1994	0.493	3
Lebanon	1995	0.502	3
Lebanon	1996	0.511	3
Lebanon	1997	0.52	3
Lebanon	1998	0.53	3
Lebanon	1999	0.54	3
Lebanon	2000	0.548	3
Lebanon	2001	0.557	3
Lebanon	2002	0.565	3
Lebanon	2003	0.574	3
Lebanon	2004	0.582	3
Lebanon	2005	0.591	3
Lebanon	2006	0.6	3
Lebanon	2007	0.609	3
Lebanon	2008	0.618	3
Lebanon	2009	0.628	3
Lebanon	2010	0.639	3
Lebanon	2011	0.649	3
Lebanon	2012	0.66	3
Lebanon	2013	0.67	3
Lebanon	2014	0.677	3
Lebanon	2015	0.685	3
Lebanon	2016	0.691	3
Lebanon	2017	0.698	3
Lebanon	2018	0.704	3
Lebanon	2019	0.708	3

Country name	Year	SDI	SDI quintiles
Libya	1990	0.405	3
Libya	1991	0.422	3
Libya	1992	0.438	3
Libya	1993	0.455	3
Libya	1994	0.472	3
Libya	1995	0.489	3
Libya	1996	0.506	3
Libya	1997	0.522	3
Libya	1998	0.538	3
Libya	1999	0.552	3
Libya	2000	0.566	3
Libya	2001	0.58	3
Libya	2002	0.593	3
Libya	2003	0.606	3
Libya	2004	0.619	3
Libya	2005	0.632	4
Libya	2006	0.645	4
Libya	2007	0.658	4
Libya	2008	0.67	4
Libya	2009	0.681	4
Libya	2010	0.691	4
Libya	2011	0.695	4
Libya	2012	0.703	4
Libya	2013	0.707	4
Libya	2014	0.707	3
Libya	2015	0.707	3
Libya	2016	0.705	3
Libya	2017	0.705	3
Libya	2018	0.707	3
Libya	2019	0.709	3

Country name	Year	SDI	SDI quintiles
Morocco	1990	0.347	1
Morocco	1991	0.354	1
Morocco	1992	0.361	1
Morocco	1993	0.367	1
Morocco	1994	0.374	1
Morocco	1995	0.38	1
Morocco	1996	0.386	1
Morocco	1997	0.392	1
Morocco	1998	0.398	1
Morocco	1999	0.403	1
Morocco	2000	0.409	1
Morocco	2001	0.414	1
Morocco	2002	0.42	1
Morocco	2003	0.426	1
Morocco	2004	0.432	1
Morocco	2005	0.439	1
Morocco	2006	0.445	1
Morocco	2007	0.452	1
Morocco	2008	0.46	1
Morocco	2009	0.467	1
Morocco	2010	0.475	1
Morocco	2011	0.483	1
Morocco	2012	0.491	1
Morocco	2013	0.499	1
Morocco	2014	0.508	1
Morocco	2015	0.516	1
Morocco	2016	0.524	1
Morocco	2017	0.533	1
Morocco	2018	0.541	1
Morocco	2019	0.548	1

Country name	Year	SDI	SDI quintiles
Oman	1990	0.441	3
Oman	1991	0.455	3
Oman	1992	0.469	3
Oman	1993	0.485	3
Oman	1994	0.5	4
Oman	1995	0.514	4
Oman	1996	0.528	4
Oman	1997	0.543	4
Oman	1998	0.558	4
Oman	1999	0.573	4
Oman	2000	0.588	4
Oman	2001	0.601	4
Oman	2002	0.615	4
Oman	2003	0.628	4
Oman	2004	0.641	4
Oman	2005	0.653	4
Oman	2006	0.664	4
Oman	2007	0.678	4
Oman	2008	0.692	4
Oman	2009	0.704	4
Oman	2010	0.715	4
Oman	2011	0.726	4
Oman	2012	0.737	4
Oman	2013	0.747	4
Oman	2014	0.754	4
Oman	2015	0.76	4
Oman	2016	0.767	4
Oman	2017	0.773	4
Oman	2018	0.778	4
Oman	2019	0.783	4

Country name	Year	SDI	SDI quintiles
Palestine	1990	0.314	1
Palestine	1991	0.32	1
Palestine	1992	0.329	1
Palestine	1993	0.337	1
Palestine	1994	0.347	1
Palestine	1995	0.356	1
Palestine	1996	0.365	1
Palestine	1997	0.375	1
Palestine	1998	0.386	1
Palestine	1999	0.397	1
Palestine	2000	0.407	1
Palestine	2001	0.415	1
Palestine	2002	0.422	1
Palestine	2003	0.43	1
Palestine	2004	0.439	1
Palestine	2005	0.449	1
Palestine	2006	0.458	1
Palestine	2007	0.467	1
Palestine	2008	0.476	1
Palestine	2009	0.486	1
Palestine	2010	0.497	1
Palestine	2011	0.509	1
Palestine	2012	0.521	1
Palestine	2013	0.533	1
Palestine	2014	0.543	1
Palestine	2015	0.553	1
Palestine	2016	0.564	1
Palestine	2017	0.573	1
Palestine	2018	0.582	1
Palestine	2019	0.588	1

Country name	Year	SDI	SDI quintiles
Qatar	1990	0.585	5
Qatar	1991	0.598	5
Qatar	1992	0.61	5
Qatar	1993	0.621	5
Qatar	1994	0.632	5
Qatar	1995	0.643	5
Qatar	1996	0.654	5
Qatar	1997	0.666	5
Qatar	1998	0.676	5
Qatar	1999	0.685	5
Qatar	2000	0.694	5
Qatar	2001	0.703	5
Qatar	2002	0.711	5
Qatar	2003	0.719	5
Qatar	2004	0.727	5
Qatar	2005	0.735	5
Qatar	2006	0.743	5
Qatar	2007	0.75	5
Qatar	2008	0.757	5
Qatar	2009	0.764	5
Qatar	2010	0.772	5
Qatar	2011	0.779	5
Qatar	2012	0.786	5
Qatar	2013	0.793	5
Qatar	2014	0.799	5
Qatar	2015	0.806	5
Qatar	2016	0.812	5
Qatar	2017	0.818	5
Qatar	2018	0.825	5
Qatar	2019	0.83	5

Country name	Year	SDI	SDI quintiles
Saudi Arabia	1990	0.48	4
Saudi Arabia	1991	0.491	4
Saudi Arabia	1992	0.504	4
Saudi Arabia	1993	0.516	4
Saudi Arabia	1994	0.529	4
Saudi Arabia	1995	0.541	4
Saudi Arabia	1996	0.554	4
Saudi Arabia	1997	0.566	4
Saudi Arabia	1998	0.578	4
Saudi Arabia	1999	0.59	4
Saudi Arabia	2000	0.602	4
Saudi Arabia	2001	0.614	4
Saudi Arabia	2002	0.625	4
Saudi Arabia	2003	0.637	4
Saudi Arabia	2004	0.65	4
Saudi Arabia	2005	0.664	4
Saudi Arabia	2006	0.677	4
Saudi Arabia	2007	0.69	5
Saudi Arabia	2008	0.703	5
Saudi Arabia	2009	0.715	5
Saudi Arabia	2010	0.726	5
Saudi Arabia	2011	0.738	5
Saudi Arabia	2012	0.75	5
Saudi Arabia	2013	0.76	5
Saudi Arabia	2014	0.769	5
Saudi Arabia	2015	0.778	5
Saudi Arabia	2016	0.786	5
Saudi Arabia	2017	0.793	5
Saudi Arabia	2018	0.8	5
Saudi Arabia	2019	0.805	5

Country name	Year	SDI	SDI quintiles
Sudan	1990	0.227	1
Sudan	1991	0.234	1
Sudan	1992	0.241	1
Sudan	1993	0.248	1
Sudan	1994	0.256	1
Sudan	1995	0.263	1
Sudan	1996	0.271	1
Sudan	1997	0.28	1
Sudan	1998	0.289	1
Sudan	1999	0.298	1
Sudan	2000	0.308	1
Sudan	2001	0.318	1
Sudan	2002	0.327	1
Sudan	2003	0.337	1
Sudan	2004	0.348	1
Sudan	2005	0.358	1
Sudan	2006	0.37	1
Sudan	2007	0.381	1
Sudan	2008	0.393	1
Sudan	2009	0.404	1
Sudan	2010	0.416	1
Sudan	2011	0.428	1
Sudan	2012	0.44	1
Sudan	2013	0.451	1
Sudan	2014	0.462	1
Sudan	2015	0.474	1
Sudan	2016	0.485	1
Sudan	2017	0.497	1
Sudan	2018	0.507	1
Sudan	2019	0.515	1

Country name	Year	SDI	SDI quintiles
Syrian Arab Republic	1990	0.367	2
Syrian Arab Republic	1991	0.376	2
Syrian Arab Republic	1992	0.387	2
Syrian Arab Republic	1993	0.398	2
Syrian Arab Republic	1994	0.409	2
Syrian Arab Republic	1995	0.421	2
Syrian Arab Republic	1996	0.432	2
Syrian Arab Republic	1997	0.443	2
Syrian Arab Republic	1998	0.454	2
Syrian Arab Republic	1999	0.465	2
Syrian Arab Republic	2000	0.476	2
Syrian Arab Republic	2001	0.486	2
Syrian Arab Republic	2002	0.497	2
Syrian Arab Republic	2003	0.508	2
Syrian Arab Republic	2004	0.521	2
Syrian Arab Republic	2005	0.535	2
Syrian Arab Republic	2006	0.548	2
Syrian Arab Republic	2007	0.56	2
Syrian Arab Republic	2008	0.572	2
Syrian Arab Republic	2009	0.583	2
Syrian Arab Republic	2010	0.594	2
Syrian Arab Republic	2011	0.601	2
Syrian Arab Republic	2012	0.605	2
Syrian Arab Republic	2013	0.606	2
Syrian Arab Republic	2014	0.607	2
Syrian Arab Republic	2015	0.608	2
Syrian Arab Republic	2016	0.61	2
Syrian Arab Republic	2017	0.613	2
Syrian Arab Republic	2018	0.616	2
Syrian Arab Republic	2019	0.619	2

Country name	Year	SDI	SDI quintiles
Tunisia	1990	0.434	3
Tunisia	1991	0.444	3
Tunisia	1992	0.455	3
Tunisia	1993	0.466	3
Tunisia	1994	0.476	3
Tunisia	1995	0.487	3
Tunisia	1996	0.498	3
Tunisia	1997	0.508	3
Tunisia	1998	0.518	3
Tunisia	1999	0.528	3
Tunisia	2000	0.538	3
Tunisia	2001	0.548	3
Tunisia	2002	0.556	3
Tunisia	2003	0.565	3
Tunisia	2004	0.574	3
Tunisia	2005	0.582	3
Tunisia	2006	0.591	3
Tunisia	2007	0.599	3
Tunisia	2008	0.607	3
Tunisia	2009	0.614	3
Tunisia	2010	0.622	3
Tunisia	2011	0.628	3
Tunisia	2012	0.634	3
Tunisia	2013	0.64	3
Tunisia	2014	0.646	3
Tunisia	2015	0.651	3
Tunisia	2016	0.657	3
Tunisia	2017	0.662	3
Tunisia	2018	0.667	3
Tunisia	2019	0.672	3

Country name	Year	SDI	SDI quintiles
Turkey	1990	0.473	4
Turkey	1991	0.483	4
Turkey	1992	0.493	4
Turkey	1993	0.504	4
Turkey	1994	0.513	4
Turkey	1995	0.523	4
Turkey	1996	0.534	4
Turkey	1997	0.545	4
Turkey	1998	0.556	4
Turkey	1999	0.566	4
Turkey	2000	0.577	4
Turkey	2001	0.587	4
Turkey	2002	0.597	4
Turkey	2003	0.607	4
Turkey	2004	0.619	3
Turkey	2005	0.63	3
Turkey	2006	0.641	3
Turkey	2007	0.652	3
Turkey	2008	0.663	3
Turkey	2009	0.671	3
Turkey	2010	0.68	3
Turkey	2011	0.689	4
Turkey	2012	0.698	4
Turkey	2013	0.707	4
Turkey	2014	0.715	4
Turkey	2015	0.723	4
Turkey	2016	0.729	4
Turkey	2017	0.736	4
Turkey	2018	0.743	4
Turkey	2019	0.748	4

Country name	Year	SDI	SDI quintiles
United Arab Emirates	1990	0.621	5
United Arab Emirates	1991	0.637	5
United Arab Emirates	1992	0.653	5
United Arab Emirates	1993	0.669	5
United Arab Emirates	1994	0.685	5
United Arab Emirates	1995	0.7	5
United Arab Emirates	1996	0.714	5
United Arab Emirates	1997	0.727	5
United Arab Emirates	1998	0.74	5
United Arab Emirates	1999	0.751	5
United Arab Emirates	2000	0.762	5
United Arab Emirates	2001	0.773	5
United Arab Emirates	2002	0.783	5
United Arab Emirates	2003	0.792	5
United Arab Emirates	2004	0.801	5
United Arab Emirates	2005	0.81	5
United Arab Emirates	2006	0.819	5
United Arab Emirates	2007	0.829	5
United Arab Emirates	2008	0.838	5
United Arab Emirates	2009	0.846	5
United Arab Emirates	2010	0.853	5
United Arab Emirates	2011	0.859	5
United Arab Emirates	2012	0.863	5
United Arab Emirates	2013	0.868	5
United Arab Emirates	2014	0.87	5
United Arab Emirates	2015	0.872	5
United Arab Emirates	2016	0.874	5
United Arab Emirates	2017	0.876	5
United Arab Emirates	2018	0.879	5
United Arab Emirates	2019	0.88	5

Country name	Year	SDI	SDI quintiles
Yemen	1990	0.176	1
Yemen	1991	0.183	1
Yemen	1992	0.191	1
Yemen	1993	0.198	1
Yemen	1994	0.207	1
Yemen	1995	0.215	1
Yemen	1996	0.224	1
Yemen	1997	0.234	1
Yemen	1998	0.243	1
Yemen	1999	0.253	1
Yemen	2000	0.263	1
Yemen	2001	0.273	1
Yemen	2002	0.283	1
Yemen	2003	0.293	1
Yemen	2004	0.303	1
Yemen	2005	0.314	1
Yemen	2006	0.325	1
Yemen	2007	0.335	1
Yemen	2008	0.346	1
Yemen	2009	0.356	1
Yemen	2010	0.366	1
Yemen	2011	0.375	1
Yemen	2012	0.384	1
Yemen	2013	0.393	1
Yemen	2014	0.402	1
Yemen	2015	0.407	1
Yemen	2016	0.41	1
Yemen	2017	0.412	1
Yemen	2018	0.413	1
Yemen	2019	0.412	1
