# Supplementary figures and images for: Burden of type 2 diabetes mellitus and its risk factors in North Africa and the Middle East, 1990–2019: findings from the Global Burden of Disease study 2019
Source: BMC Public Health. 2024 Jan 5;24:98. doi: 10.1186/s12889-023-16540-8 (PMC10768242; doi:10.1186/s12889-023-16540-8)

1990

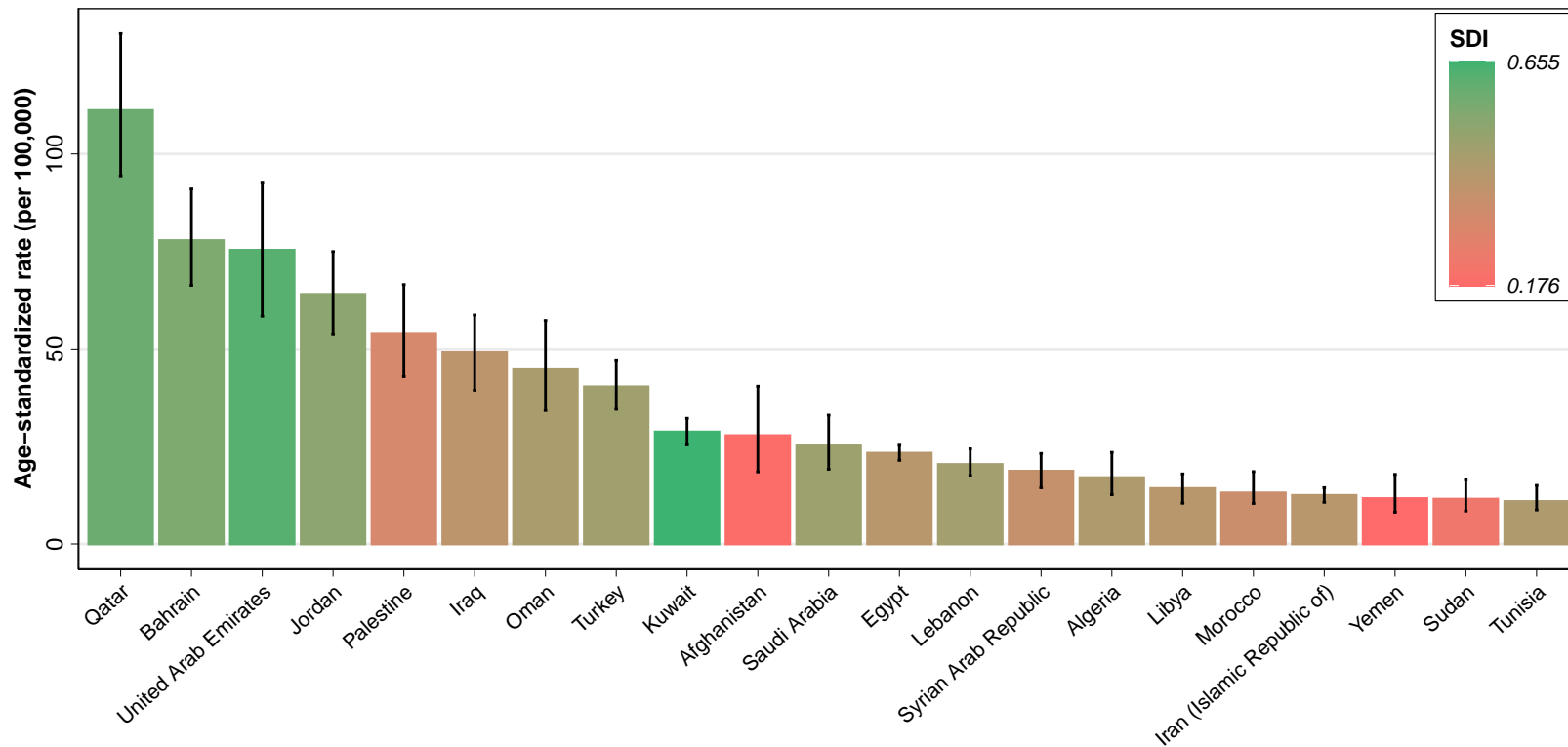

2000

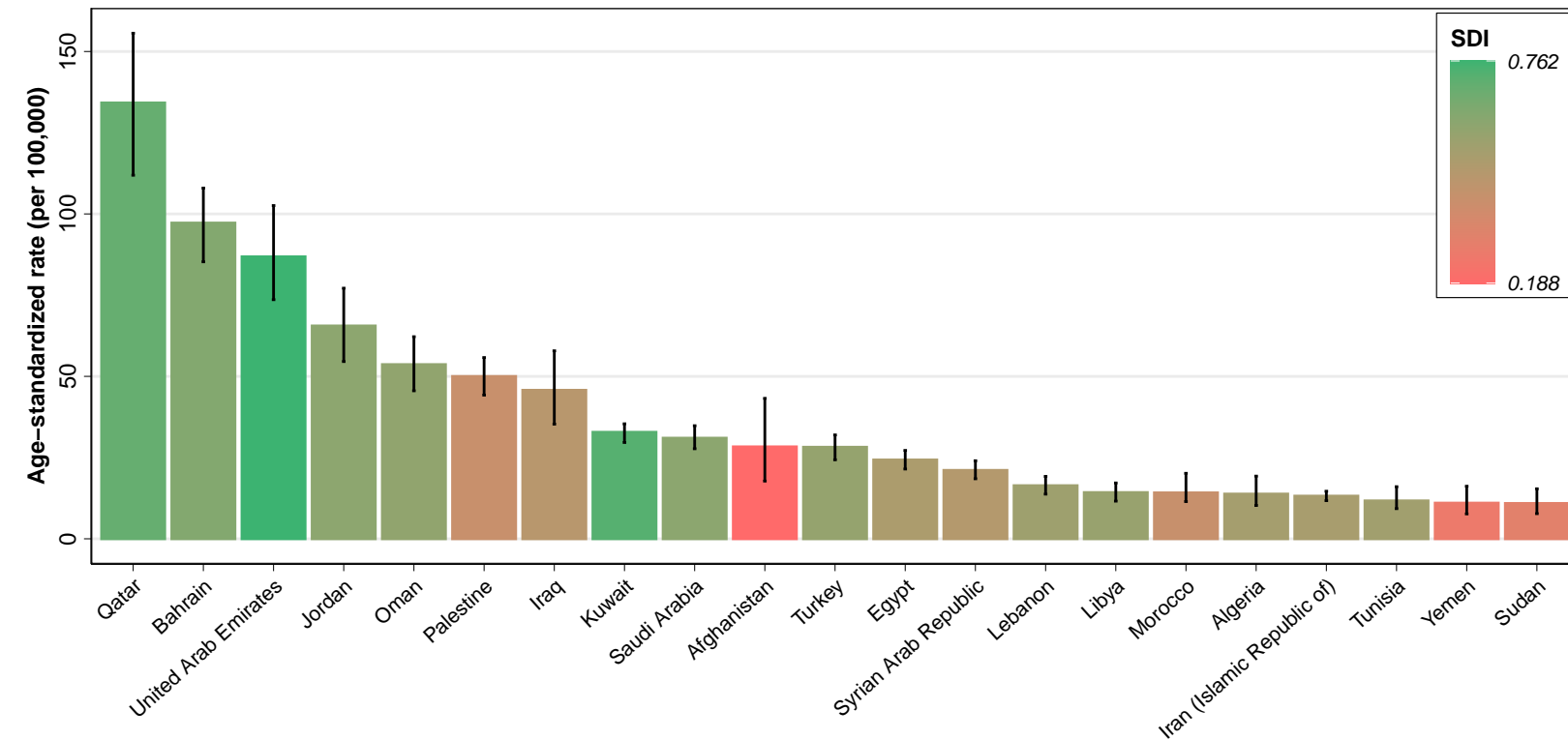

2010

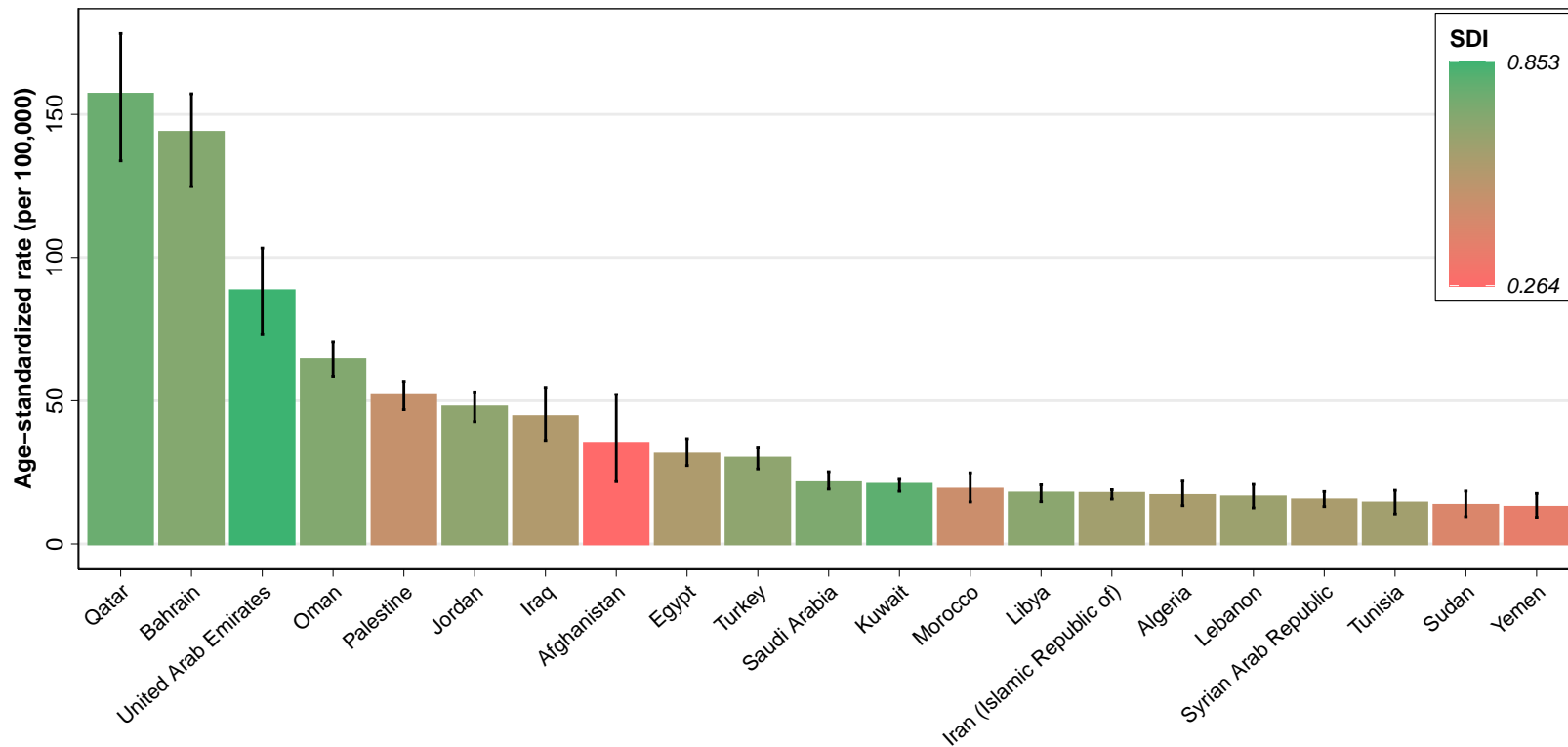

2019

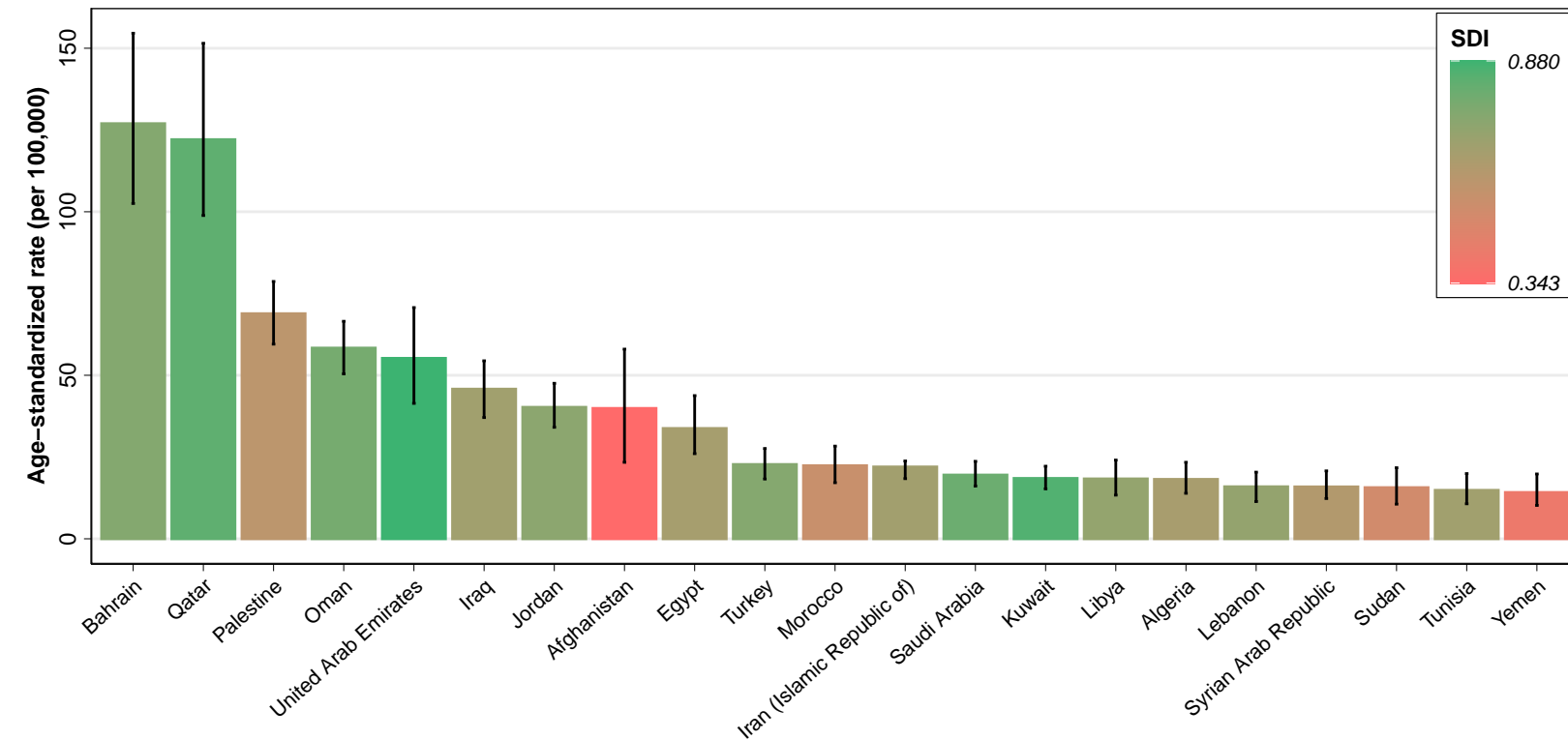

Supplement: Supplementary file 6 — Additional file 6: Supplementary Figure 1. Ordering of the region countries based on the age-standardized mortality rate and colored based on the socio-demographic index in 1990, 2000, 2010, and 2019. [file 12889_2023_16540_MOESM6_ESM.pdf]

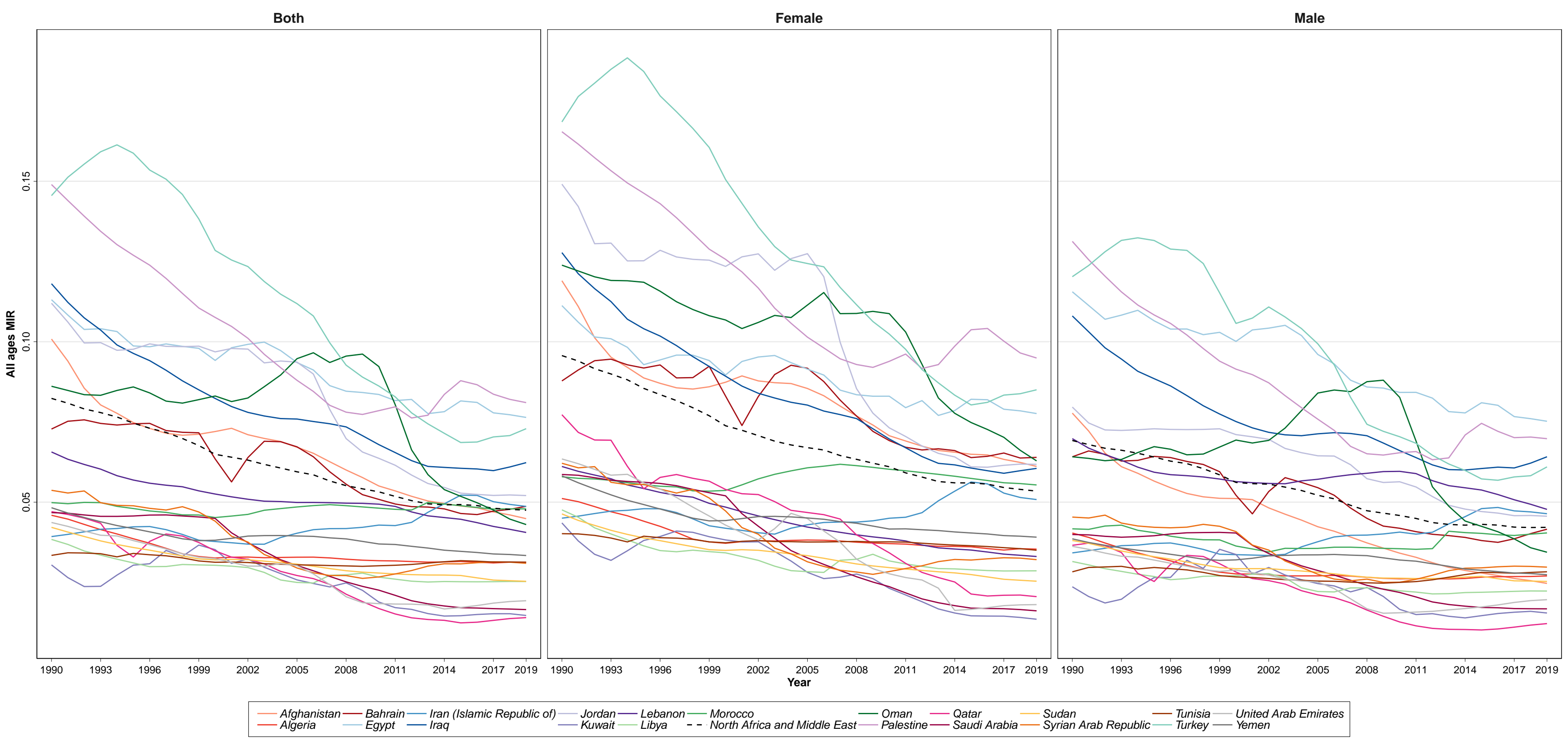

Supplement: Supplementary file 7 — Additional file 7: Supplementary Figure 2. Time trend of mortality-to-incidence ratio in the region and its countries by sex, 1990 to 2019. [file 12889_2023_16540_MOESM7_ESM.pdf]
