# Supplementary material for: Burden of type 2 diabetes mellitus and its risk factors in North Africa and the Middle East, 1990–2019: findings from the Global Burden of Disease study 2019
Source: BMC Public Health. 2024 Jan 5;24:98. doi: 10.1186/s12889-023-16540-8 (PMC10768242; doi:10.1186/s12889-023-16540-8)
Supplement: Supplementary file 8 — Additional file 8: Supplementary Figure 3. Geographical distribution of attributed burden to T2DM risk factors by measures in 1990 and 2019. [file 12889_2023_16540_MOESM8_ESM.pdf]

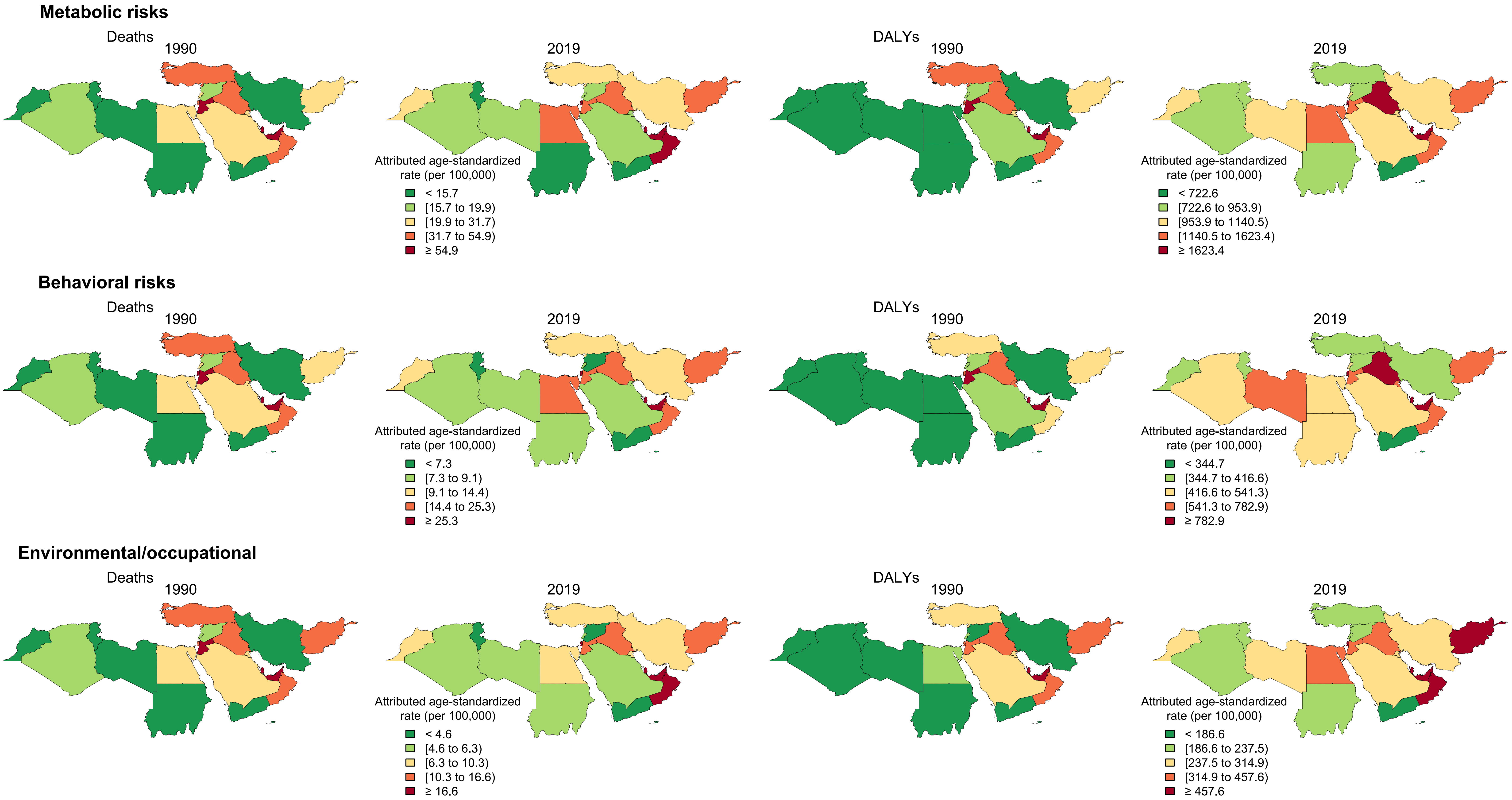
Metabolic risks
Deaths
1990
2019
DALYs
1990
2019
Attributed age-standardized rate (per 100,000)
< 15.7
[15.7 to 19.9)
[19.9 to 31.7)
[31.7 to 54.9)
≥ 54.9
Attributed age-standardized rate (per 100,000)
< 722.6
[722.6 to 953.9)
[953.9 to 1140.5)
[1140.5 to 1623.4)
≥ 1623.4
Behavioral risks
Deaths
1990
2019
DALYs
1990
2019
Attributed age-standardized rate (per 100,000)
< 7.3
[7.3 to 9.1)
[9.1 to 14.4)
[14.4 to 25.3)
≥ 25.3
Attributed age-standardized rate (per 100,000)
< 344.7
[344.7 to 416.6)
[416.6 to 541.3)
[541.3 to 782.9)
≥ 782.9
Environmental/occupational
Deaths
1990
2019
DALYs
1990
2019
Attributed age-standardized rate (per 100,000)
< 4.6
[4.6 to 6.3)
[6.3 to 10.3)
[10.3 to 16.6)
≥ 16.6
Attributed age-standardized rate (per 100,000)
< 186.6
[186.6 to 237.5)
[237.5 to 314.9)
[314.9 to 457.6)
≥ 457.6
